# Supplementary material for: Drug induced pancreatitis: A systematic review of case reports to determine potential drug associations
Source: PLoS One. 2020 Apr 17;15(4):e0231883. doi: 10.1371/journal.pone.0231883 (PMC7164626; doi:10.1371/journal.pone.0231883)
Supplement: S1 Text — (DOCX) [file pone.0231883.s001.docx]

S1 TEXT: Literature search strategy

Ovid Multifile

Database: Embase Classic+Embase, Ovid MEDLINE(R) Epub Ahead of Print, In-Process & Other Non-Indexed Citations, Ovid MEDLINE(R) Daily and Ovid MEDLINE(R) <1946 to Present>

Search Strategy:

--------------------------------------------------------------------------------

1 Pancreatitis/ci (3325)

2 Pancreatitis, Acute Necrotizing/ci (262)

3 (pancreati* adj5 ((drug adj2 induc*) or (drugs adj2 induc*) or (chemical* adj2 induc*) or (medication? adj2 induc*) or (pharmaceutic* adj2 induc*) or (prescri* adj2 nduc*))).tw,kw. (804)

4 (pancreati* adj5 ((drug adj2 assoc*) or (drugs adj2 assoc*) or (chemical* adj2 assoc*) or (medication? adj2 assoc*) or (pharmaceutic* adj2 assoc*) or (prescri* adj2 assoc*))).tw,kw. (123)

5 (pancreati* adj5 ((drug adj2 caus*) or (drugs adj2 caus*) or (chemical* adj2 caus*) or (medication? adj2 caus*) or (pharmaceutic* adj2 caus*) or (prescri* adj2 caus*))).tw,kw. (106)

6 (pancreati* adj5 ((drug adj2 relat*) or (drugs adj2 relat*) or (chemical* adj2 relat*) or (medication? adj2 relat*) or (pharmaceutic* adj2 relat*) or (prescri* adj2 relat*))).tw,kw. (94)

7 (pancreati* adj5 ((drug adj2 initiat*) or (drugs adj2 initiat*) or (chemical* adj2 initiat*) or (medication? adj2 initiat*) or (pharmaceutic* adj2 initiat*) or (prescri* adj2 initiat*))).tw,kw. (6)

8 ((pancreati* adj5 (caus* or etiolog* or induc* or initiat*)) and (chemical or chemicals or chemically or drug or drugs or medication? or pharmaceutic* or prescri*)).tw,kw. (4684)

9 or/1-8 (7960)

10 exp Pancreatitis/ (141381)

11 po.fs. (552295)

12 exp Pharmacologic Actions/ae, to (764674)

13 exp Organic Chemicals/ae, to (1102505)

14 exp Inorganic Chemicals/ae, to (211061)

15 exp Drug Therapy/ae (69970)

16 "Drug-Related Side Effects and Adverse Reactions"/ (210261)

17 ((adverse effect? or adverse event? or adverse reaction? or adverse incident? or side effect?) adj5 (chemical or chemicals or chemically or drug or drugs or medication? or pharmaceutic* or prescri*)).tw,kw. (103389)

18 ((adverse drug? or adverse chemical* or adverse medication* or adverse pharmaceutic* or adverse prescri*) adj5 (effect? or event? or incident* or reaction?)).tw,kw. (42411)

19 ((injurious effect? or injurious event? or injurious reaction? or injurious incident?) adj5 (chemical or chemicals or chemically or drug or drugs or medication? or pharmaceutic* or prescri*)).tw,kw. (125)

20 ((undesirable effect? or undesirable event? or undesirable reaction? or undesirable incident?) adj5 (chemical or chemicals or chemically or drug or drugs or medication? or pharmaceutic* or prescri*)).tw,kw. (481)

21 ((unintended effect? or unintended event? or unintended reaction? or unintended incident?) adj5 (chemical or chemicals or chemically or drug or drugs or medication? or pharmaceutic* or prescri*)).tw,kw. (78)

22 (ADE or ADEs or ADR or ADRs).tw,kw. (27541)

23 ((chemical or chemicals or chemically or drug or drugs or medication? or pharmaceutic* or prescri*) adj2 (safe or safety or unsafe or toxic*)).tw,kw. (73985)

24 (safety or safe or unsafe).ti. (283236)

25 or/11-24 (2690489)

26 10 and 25 (14315)

27 9 or 26 [DRUG-INDUCED PANCREATITIS] (19775)

28 case reports/ (1827795)

29 ((case or cases) adj1 (report or reports or series or study or studies)).tw,kw. (989817)

30 ((case or cases) adj1 (history or histories)).tw,kw. (37359)

31 or/28-30 (2616089)

32 27 and 31 [CASE STUDIES - DRUG-INDUCED PANCREATITIS] (2105)

33 exp Animals/ not (exp Animals/ and Humans/) (16172081)

34 32 not 33 (1660)

35 34 use ppez [MEDLINE RECORDS] (1346)

36 pancreatitis/ (98629)

37 acute hemorrhagic pancreatitis/ (4135)

38 acute pancreatitis/ (26885)

39 hemorrhagic pancreatitis/ (544)

40 or/36-39 (120376)

41 drug induced disease/ (38346)

42 40 and 41 (504)

43 (pancreati* adj5 ((drug adj2 induc*) or (drugs adj2 induc*) or (chemical* adj2 induc*) or (medication? adj2 induc*) or (pharmaceutic* adj2 induc*) or (prescri* adj2 induc*))).tw,kw. (806)

44 (pancreati* adj5 ((drug adj2 assoc*) or (drugs adj2 assoc*) or (chemical* adj2 assoc*) or (medication? adj2 assoc*) or (pharmaceutic* adj2 assoc*) or (prescri* adj2 assoc*))).tw,kw. (123)

45 (pancreati* adj5 ((drug adj2 caus*) or (drugs adj2 caus*) or (chemical* adj2 caus*) or (medication? adj2 caus*) or (pharmaceutic* adj2 caus*) or (prescri* adj2 caus*))).tw,kw. (106)

46 (pancreati* adj5 ((drug adj2 relat*) or (drugs adj2 relat*) or (chemical* adj2 relat*) or (medication? adj2 relat*) or (pharmaceutic* adj2 relat*) or (prescri* adj2 relat*))).tw,kw. (94)

47 (pancreati* adj5 ((drug adj2 initiat*) or (drugs adj2 initiat*) or (chemical* adj2 initiat*) or (medication? adj2 initiat*) or (pharmaceutic* adj2 initiat*) or (prescri* adj2 initiat*))).tw,kw. (6)

48 ((pancreati* adj5 (caus* or etiolog* or induc* or initiat*)) and (chemical or chemicals or chemically or drug or drugs or medication? or pharmaceutic* or prescri*)).tw,kw. (4684)

49 or/42-48 (5179)

50 pancreatitis/si (6329)

51 acute hemorrhagic pancreatitis/si (110)

52 acute pancreatitis/si (1657)

53 hemorrhagic pancreatitis/si (68)

54 or/50-53 (7939)

55 exp drug therapy/ae [Adverse Drug Reaction] (69970)

56 adverse drug reaction/ (296227)

57 ((adverse effect? or adverse event? or adverse reaction? or adverse incident? or side effect?) adj5 (chemical or chemicals or chemically or drug or drugs or medication? or pharmaceutic* or prescri*)).tw,kw. (103389)

58 ((adverse drug? or adverse chemical* or adverse medication* or adverse pharmaceutic* or adverse prescri*) adj5 (effect? or event? or incident* or reaction?)).tw,kw. (42411)

59 ((injurious effect? or injurious event? or injurious reaction? or injurious incident?) adj5 (chemical or chemicals or chemically or drug or drugs or medication? or pharmaceutic* or prescri*)).tw,kw. (125)

60 ((undesirable effect? or undesirable event? or undesirable reaction? or undesirable incident?) adj5 (chemical or chemicals or chemically or drug or drugs or medication? or pharmaceutic* or prescri*)).tw,kw. (481)

61 ((unintended effect? or unintended event? or unintended reaction? or unintended incident?) adj5 (chemical or chemicals or chemically or drug or drugs or medication? or pharmaceutic* or prescri*)).tw,kw. (78)

62 (ADE or ADEs or ADR or ADRs).tw,kw. (27541)

63 drug safety/ (296578)

64 ((chemical or chemicals or chemically or drug or drugs or medication? or pharmaceutic* or prescri*) adj2 (safe or safety or unsafe or toxic*)).tw,kw. (73985)

65 (safety or safe or unsafe).ti. (283236)

66 or/55-65 (1028325)

67 40 and 66 (6109)

68 54 or 67 (10708)

69 49 or 68 [DRUG-INDUCED PANCREATITIS] (14946)

70 case report/ (4060654)

71 ((case or cases) adj1 (report or reports or series or study or studies)).tw,kw. (989817)

72 ((case or cases) adj1 (history or histories)).tw,kw. (37359)

73 or/70-72 (4438349)

74 69 and 73 [CASE REPORTS - DRUG-INDUCED PANCREATITIS] (2242)

75 exp animal experimentation/ or exp models animal/ or exp animal experiment/ or nonhuman/ or exp vertebrate/ (44577783)

76 exp human/ or exp human experimentation/ or exp human experiment/ (34999937)

77 75 not 76 (9579534)

78 74 not 77 [ANIMAL-ONLY REMOVED] (2236)

79 78 use emczd [EMBASE RECORDS] (1801)

80 35 or 79 [BOTH DATABASES] (3147)

81 remove duplicates from 80 [TOTAL UNIQUE RECORDS] (2326)

82 81 use ppez [MEDLINE UNIQUE RECORDS] (1313)

83 81 use emczd [EMBASE RECORDS] (1013)

***************************
