# Supplementary material for: Drug induced pancreatitis: A systematic review of case reports to determine potential drug associations
Source: PLoS One. 2020 Apr 17;15(4):e0231883. doi: 10.1371/journal.pone.0231883 (PMC7164626; doi:10.1371/journal.pone.0231883)
Supplement: S3 Text — (DOCX) [file pone.0231883.s003.docx]

S3 TEXT: List of included studies

Abdul-Ghaffar, N. U., and M. R. el-Sonbaty. “Pancreatitis and Rhabdomyolysis Associated with Lovastatin-Gemfibrozil Therapy.” *Journal of Clinical Gastroenterology* 21, no. 4 (1995): 340–41.

Abdullah, A. M., R. B. Scott, and S. R. Martin. “Acute Pancreatitis Secondary to 5-Aminosalicylic Acid in a Child with Ulcerative Colitis.” *Journal of Pediatric Gastroenterology and Nutrition* 17, no. 4 (1993): 441–44.

Abou Chacra, L., M. Ghosn, E. Ghayad, and K. Honein. “A Case of Pancreatitis Associated with All-Trans-Retinoic Acid Therapy in Acute Promyelocytic Leukemia.” *The Hematology Journal : The Official Journal of the European Haematology Association* 2, no. 6 (2001): 406–7.

Abraham, Albin, Pooja Raghavan, Rajshree Patel, Dhyan Rajan, Jaspreet Singh, and Paul Mustacchia. “Acute Pancreatitis Induced by Methimazole Therapy.” *Case Reports in Gastroenterology* 6, no. 2 (2012): 223–31.

Acharya, Gyanendra Kumar, Angel Guido Hita, Sai-Ching J. Yeung, and Sai-Ching J. Yeung. “Diabetic Ketoacidosis and Acute Pancreatitis: Serious Adverse Effects of Everolimus.” *Annals of Emergency Medicine* 69, no. 5 (2017): 666–67. <https://doi.org/10.1016/j.annemergmed.2017.01.002>.

Achecar, Justo L., Fernandez M. Rivero, Reinoso J. Cobo, and Olmos L. Ruiz Del Arbol. “Doxycycline Induced-Acute Pancreatitis.” *Medicina Clinica* 134, no. 15 (2010): 705–6.

Adachi, E., K. Okazaki, Y. Matsushima, H. Seno, K. Uchida, H. Nakase, C. Kawanami, T. Nakamura, and T. Chiba. “Acute Pancreatitis Secondary to 5-Aminosalicylic Acid Therapy in a Patient with Ulcerative Colitis.” *International Journal of Pancreatology : Official Journal of the International Association of Pancreatology* 25, no. 3 (1999): 217–21.

Adam, Jean Philippe, Philippe Gauthier, and Nathalie Letarte. “Safe Administration of Docetaxel after Weekly Paclitaxel-Induced Acute Pancreatitis.” *Journal of Oncology Pharmacy Practice : Official Publication of the International Society of Oncology Pharmacy Practitioners*, 2016. <http://ovidsp.ovid.com/ovidweb.cgi?T=JS&PAGE=reference&D=medp&NEWS=N&AN=27466225>.

Agarwal, M., H. Lunt, and R. Scott. “Hormone Replacement Therapy, Diabetes and Pancreatitis Secondary to Hypertriglyceridaemia.” *The New Zealand Medical Journal* 110, no. 1055 (1997): 426.

Agito, Katrina, and Andrea Manni. “Acute Pancreatitis Induced by Methimazole in a Patient With Subclinical Hyperthyroidism.” *Journal of Investigative Medicine High Impact Case Reports* 3, no. 2 (2015): 2324709615592229.

Ahmad, Imran, Edward Ruby, Harris Usman, Mateen Hotiana, Mehnaz Hussain, and Fahd Rahman. “Ezetimibe-Induced Acute Pancreatitis.” *Southern Medical Journal* 100, no. 4 (2007): 409–10.

Aissaoui, M., N. Mounedji, P. Mathelier-Fusade, and F. Leynadier. “[Pancreatitis Caused by Azathioprine: Immuno-Allergy?].” *Presse Medicale (Paris, France : 1983)* 25, no. 34 (1996): 1650.

Alagozlu, Hakan, Mehmet Cindoruk, and Selahattin Unal. “Tamoxifen-Induced Severe Hypertriglyceridaemia and Acute Pancreatitis.” *Clinical Drug Investigation* 26, no. 5 (2006): 297–302.

Alberti-Flor, J. J. “Pancreatitis Associated with Ondansetron [2].” *Journal of the National Cancer Institute* 87, no. 9 (1995): 689–90.

Alexander, S., and D. Dowling. “Azathioprine Pancreatitis in Inflammatory Bowel Disease and Successful Subsequent Treatment with Mercaptopurine.” *Internal Medicine Journal* 35, no. 9 (2005): 570–71.

Ali, M. F., and K. Y. Loh. “Sodium Valproate Induced Necrotising Pancreatitis: A Case Report.” *Malaysian Family Physician : The Official Journal of the Academy of Family Physicians of Malaysia* 8, no. 3 (2013): 28–30.

Allaouchiche, B., F. Duflo, L. Cotte, L. Mathon, and D. Chassard. “Acute Pancreatitis with Severe Lactic Acidosis in an HIV-Infected Patient on Didanosine Therapy.” *The Journal of Antimicrobial Chemotherapy* 44, no. 1 (1999): 137–38.

Almeida, Daniel M., M. Raphaelle Jean, Anastasiya Chystsiakova, Ellen Monahan, Stephanie B. Oliveira, and Iona M. Monteiro. “Levetiracetam-Associated Acute Pancreatitis in an Adolescent with Autism: A Case Report.” *Pancreas* 42, no. 1 (2013): 177–78.

Alsubaie, S., and M. H. Almalki. “Metformin Induced Acute Pancreatitis.” *Dermato-Endocrinology* 5, no. 2 (2013): 317–18.

Altman, A. J., P. Dinndorf, and J. J. Quinn. “Acute Pancreatitis in Association with Cytosine Arabinoside Therapy.” *Cancer* 49, no. 7 (1982): 1384–86.

Al-Zayani, J. “Acute Pancreatitis Associated with the Use of 5-Aminosaliclic Acid and Sulfasalazine in a Patient with Ulcerative Colitis.” *Journal of the Bahrain Medical Society* 9, no. 1 (1997): 55–59.

Amar, S., K. J. Wu, and W. W. Tan. “Sorafenib-Induced Pancreatitis [4].” *Mayo Clinic Proceedings* 82, no. 4 (2007): 521.

Amaravadi, R. K., B. C. Jacobson, D. H. Solomon, and M. A. Fischer. “Acute Pancreatitis Associated with Rofecoxib [18].” *American Journal of Gastroenterology* 97, no. 4 (2002): 1077–78.

Amery, A., J. Vandenbroucke, J. L. Desbuquoit, and Groote J. De. “Pancreatitis during Clonidine Treatment.” *T.GASTRO-ENT.* 16, no. 3 (1973): 179–85.

Anagnostopoulos, G. K., P. Kostopoulos, S. Tsiakos, G. Margantinis, and D. Arvanitidis. “Fulminant Pancreatitis Associated with Ramipril Therapy.” *Pancreas* 27, no. 3 (2003): 278–79.

Anagnostopoulos, G. K., S. Tsiakos, G. Margantinis, P. Kostopoulos, and D. Arvanitidis. “Acute Pancreatitis Due to Pravastatin Therapy.” *Journal of the Pancreas* 4, no. 3 (2003): 129–32.

Anand, H., G. Parthasarathi, and M. Ramesh. “Stavudine-Induced Pancreatitis Followed by Lopinavir-Ritonavir-Induced Pancreatitis.” *Journal of Postgraduate Medicine* 54, no. 2 (2008): 153–55.

Anderson, J. R., G. W. Johnston, and T. L. Kennedy. “Drug-Associated Recurrent Pancreatitis.” *Digestive Surgery* 2, no. 1 (1985): 24–26.

Anderson, P. E., G. G. J. Ellis, and S. M. Austin. “Case Report: Metolazone-Associated Hypercalcemia and Acute Pancreatitis.” *The American Journal of the Medical Sciences* 302, no. 4 (1991): 235–37.

Anton Aranda, E., and E. Altuna Basurto. “[Acute Pancreatitis and Erythromycin].” *Medicina Clinica* 96, no. 16 (1991): 638.

Antonow, D. R. “Acute Pancreatitis Associated with Trimethoprim-Sulfamethoxazole.” *Annals of Internal Medicine* 104, no. 3 (1986): 363–65.

Arai, Yoshinori, Seiji Arihiro, Daisuke Ide, Isao Odagi, Munenori Itagaki, Nobuhiko Komoike, Yutaka Nakao, et al. “Acute Pancreatitis Due to PH-Dependent Mesalazine That Occurred in the Course of Ulcerative Colitis.” *Case Reports in Gastroenterology* 5, no. 3 (2011): 610–16.

Arbel, Y., D. Weinstein, R. Yogev, and A. Halevy. “Acute Pancreatitis Following Clomiphene Citrate Treatment: Case Report and Review of the Literature.” *International Journal of Surgery* 6, no. 6 (2008): 483–84.

Arellano, Lucia, Anna Altaba, Conrad Santamaria, and Juan Antonio Garcia-Vicente. “[Acute Pancreatitis in a Patient Treated with Losartan].” *Atencion Primaria* 46, no. 6 (2014): 316–17.

Arnold, F., P. J. Doyle, and G. Bell. “Acute Pancreatitis in a Patient Treated with Cimetidine.” *Lancet (London, England)* 1, no. 8060 (1978): 382–83.

Artero, Ana, Marine Bourguet, Rosario I. Lorente, and Jose T. Real. “[Acute Pancreatitis in a Patient Treated with Liraglutide].” *Medicina Clinica* 141, no. 8 (2013): 368–69.

Asma, O. K., G. Dalila, K. Asma, B. Norsaf, La E. He, T. Dorra, and K. Jamel. “Acute Pancreatitis Secondary to Long-Term 5-Aminosalicylic Acid Therapy in a Patient with Ulcerative Colitis: A Case-Report.” *Tunisie Medicale* 92, no. 6 (2014): 423.

Atam, V., J. Singh, K. Agrawal, A. Dinkar, and I. Atam. “A Case Report of Valproate-Induced Acute Pancreatitis.” *JMS - Journal of Medical Society* 31, no. 1 (2017): 48–49.

Aubry, A., C. Alandry, and C. Lemiere. “[Acute Pancreatitis during Treatment with Salazosulfapyridine].” *Presse Medicale (Paris, France : 1983)* 18, no. 2 (1989): 80.

Avraam, C., K. Siomos, M. C. Armenaka, and M. L. Sion. “Clarithromycin Associated Acute Pancreatitis.” *Annals of Gastroenterology* 20, no. 1 (2007): 35–37.

Aygencel, Gulbin, Betul Akbuga, and Ayfer Keles. “Acute Pancreatitis Following Naproxen Intake.” *European Journal of Emergency Medicine : Official Journal of the European Society for Emergency Medicine* 13, no. 6 (2006): 372.

Ayoola, E. A., N. S. Dahmash, D. Ajarim, and S. M. Al-Mugairin. “Delayed Multiple Toxic Reactions Possibly Related to Valproate Therapy in a Saudi Patient [4].” *Annals of Saudi Medicine* 14, no. 2 (1994): 163–64.

Ayoub, Walaa A., Ashok A. Kumar, Hossam S. Naguib, and Harris C. Taylor. “Exenatide-Induced Acute Pancreatitis.” *Endocrine Practice : Official Journal of the American College of Endocrinology and the American Association of Clinical Endocrinologists* 16, no. 1 (2010): 80–83.

Ayyildiz, T., T. Aydin, A. T. Eminler, C. Yildirim, K. Irak, M. Kiyici, S. Gurel, S. G. Nak, M. Gulten, and E. Dolar. “Sorafenib-Induced Pancreatitis.” *Journal of Experimental and Clinical Medicine (Turkey)* 32, no. 3 (2015): 133–35.

Azar, Nabil J., and Patsy Aune. “Acute Pancreatitis and Elevated Liver Transaminases after Rapid Titration of Oral Levetiracetam.” *Journal of Clinical Neuroscience : Official Journal of the Neurosurgical Society of Australasia* 21, no. 6 (2014): 1053–54.

Baciewicz, A. M., T. J. King, and D. R. Sokos. “Acute Pancreatitis Associated with Celecoxib [12].” *Annals of Internal Medicine* 132, no. 8 (2000): 680.

Baffoni, L., V. Durante, and M. Grossi. “Acute Pancreatitis Induced by Telmisartan Overdose [3].” *Annals of Pharmacotherapy* 38, no. 6 (2004): 1088.

Bahamonde Carrasco, A., A. Moran Blanco, and J. L. Olcoz Goni. “[Acute Pancreatitis Caused by Valproic Acid: Apropos a Case].” *Gastroenterologia y Hepatologia* 19, no. 5 (1996): 253–54.

Balasch, J., S. Martinez-Roman, J. Carreras, and J. A. Vanrell. “Acute Pancreatitis Associated with Danazol Treatment for Endometriosis.” *Human Reproduction (Oxford, England)* 9, no. 6 (1994): 1163–65.

Bank, L., and J. P. Wright. “6-Mercaptopurine-Related Pancreatitis in 2 Patients with Inflammatory Bowel Disease.” *Digestive Diseases and Sciences* 29, no. 4 (1984): 357–59.

Bank, S., and I. N. Marks. “Case Reports. Hyperlipaemic Pancreatitis and the Pill.” *Postgraduate Medical Journal* 46, no. 539 (1960): 576–78.

Bär, Sarah, Fritz Daudel, and Thomas Zueger. “Ominous Triad Triggered by High-Dose Glucocorticosteroid Therapy.” *BMJ Case Reports* 2017 (June 18, 2017). <https://doi.org/10.1136/bcr-2017-220328>.

Bartels, R. H., J. A. van der Spek, and H. R. Oosten. “Acute Pancreatitis Due to Sulfamethoxazole-Trimethoprim.” *Southern Medical Journal* 85, no. 10 (1992): 1006–7.

Barthet, M., P. Brunet, J. C. Bernard, B. Dussol, F. Rodor, J. Jouglard, Y. Berland, and J. Sahel. “[Acute Pancreatitis during Treatment with Meglumine Antimoniate (Glucantime)].” *Gastroenterologie Clinique et Biologique* 18, no. 1 (1994): 90–92.

Basturk, Ahmet, Aygen Yilmaz, Meryem Keceli, and Reha Artan. “Infliximab Treatment in a Paediatric Patient with Ulcerative Colitis, Who Developed Acute Pancreatitis Due to Azathioprine during Follow-Up.” *Gastroenterology Review* 3 (2017): 235–37. <https://doi.org/10.5114/pg.2017.70479>.

Batalden, P. B., B. J. Van Dyne, and J. Cloyd. “Pancreatitis Associated with Valproic Acid Therapy.” *Pediatrics* 64, no. 4 (1979): 520–22.

Battaglia, M., P. Ditonno, S. Palazzo, C. Bettocchi, L. Garofalo, and F. P. Selvaggi. “Lethal Somatostatin Analog-Induced Acute Necrotizing Pancreatitis in a Patient with Hormone-Refractory Prostate Cancer.” *Scandinavian Journal of Urology and Nephrology* 40, no. 5 (2006): 423–25.

Bauters, T., V. Mondelaers, H. Robays, H. De Wilde, Y. Benoit, and B. De Moerloose. “Methemoglobinemia and Hemolytic Anemia after Rasburicase Administration in a Child with Leukemia.” *International Journal of Clinical Pharmacy* 35, no. 3 (2013): 303–5.

Bayard, J. M. F., O. S. Descamps, S. Evrard, J. M. Dumonceau, L. Servais, Z. Zingir, S. Adams, et al. “Case Report: Acute Pancreatitis Induced by Clozapine.” *Acta Gastro-Enterologica Belgica* 68, no. 1 (2005): 92–94.

Baysal, B., Y. Kayar, A. Ozmen, M. ElShobaky, N. Mahdi, A. T. Ince, A. Danaliotlu, and H. Senturk. “Olanzapine-Induced Acute Pancreatitis.” *Turkish Journal of Gastroenterology* 26, no. 3 (2015): 289–90.

Beaufort, Carine de, Peter Beck, Roland Seligmann, Linda de Meirleir, and Jean de Schepper. “Acute Pancreatitis after Growth Hormone Treatment: Disease or Treatment Linked?” *European Journal of Pediatrics* 165, no. 9 (2006): 652–53.

Bekassy, A., T. Wiebe, and P. Hochbergs. “Erwinase-Induced Pancreatitis.” *Lancet (London, England)* 340, no. 8834–8835 (1992): 1552–53.

Belaiche, G., G. Ley, and J. L. Slama. “[Acute Pancreatitis Associated with Atorvastatine Therapy].” *Gastroenterologie Clinique et Biologique* 24, no. 4 (2000): 471–72.

Belli, Hasan, Yasar Sertbas, and Yilmaz Bayik. “Olanzapine-Induced Diabetes Due to Pancreatitis.” *Indian Journal of Gastroenterology : Official Journal of the Indian Society of Gastroenterology* 24, no. 6 (2005): 273.

Ben, Mokhtar H., H. Thabet, I. Zaghdoudi, and M. Amamou. “Metformin Associated Acute Pancreatitis.” *Veterinary and Human Toxicology* 44, no. 1 (2002): 47–48.

Ben-Ami, H., S. Pollack, P. Nagachandran, I. Lashevsky, D. Yarnitsky, and Y. Edoute. “Reversible Pancreatitis, Hepatitis, and Peripheral Polyneuropathy Associated with Parenteral Gold Therapy.” *The Journal of Rheumatology* 26, no. 9 (1999): 2049–50.

Benavente Fernandez, A., O. Barakat Shrem, I. Ibanez Godoy, M. J. Fernandez Perez, and M. A. Bolivar Raya. “[Carbamacepine - Induced Pancreatitis].” *Anales de Medicina Interna (Madrid, Spain : 1984)* 21, no. 4 (2004): 199–200.

Berent, I., J. Carabeth, M. M. Cordero, R. Cordero, B. Sugerman, and D. Robinson. “Pancreatitis Associated with Risperidone Treatment? [3].” *American Journal of Psychiatry* 154, no. 1 (1997): 130–31.

Berger, T. M., W. J. Cook, A. S. O’Marcaigh, and D. Zimmerman. “Acute Pancreatitis in a 12-Year-Old Girl after an Erythromycin Overdose.” *Pediatrics* 90, no. 4 (1992): 624–26.

Bernas Albeniz, A., D. A. Aveiga Valencia, L. Etxeberria Zabala, J. Zaldibar-Gerrikagoitia Bilbao, and L. Aguilera Celorrio. “Acute Pancreatitis in ICU Secondary to Treatment with Tigecycline.” *Revista Espanola de Anestesiologia y Reanimacion* 64, no. 1 (2017): 46–49.

Berrak, S. G., C. Canpolat, P. Berik, and G. Kiyan. “Pancreatic Pseudocyst Following Acute Pancreatitis Induced by L-Asparaginase Treatment.” *International Journal of Pediatric Hematology/Oncology* 7, no. 5–6 (2001): 413–16.

Bertolone, S. J., M. M. Fuenfer, D. B. Groff, and C. C. Patel. “Delayed Pancreatic Pseudocyst Formations. Long-Term Complication of L-Asparaginase Treatment.” *Cancer* 50, no. 12 (1982): 2964–66.

Besseau, M., J. C. Delchier, M. Blazquez, and J. C. Soule. “Acute Pancreatitis Induced by Mesalazine (Pentasa) [7].” *Gastroenterologie Clinique et Biologique* 15, no. 2 (1991): 174–75.

Betrosian, A. P., M. Balla, M. Papanikolaou, G. Kofinas, and G. Georgiadis. “Post-Operative Pancreatitis after Propofol Administration [3].” *Acta Anaesthesiologica Scandinavica* 45, no. 8 (2001): 1052.

Bilar, Juliana Miguel, Roberto Jose Carvalho-Filho, Carolina Frade Magalhaes Girardin Pimentel Mota, Patricia da Silva Fucuta, and Maria Lucia Cardoso Gomes Ferraz. “Acute Pancreatitis Associated with Boceprevir: A Case Report.” *The Brazilian Journal of Infectious Diseases : An Official Publication of the Brazilian Society of Infectious Diseases* 18, no. 4 (2014): 454–56.

Binek, J., A. Hany, and M. Heer. “Valproic-Acid-Induced Pancreatitis. Case Report and Review of the Literature.” *Journal of Clinical Gastroenterology* 13, no. 6 (1991): 690–93.

Birchfield, G. R., J. H. Ward, B. G. Redman, L. Flaherty, and W. E. Samlowski. “Acute Pancreatitis Associated with High-Dose Interleukin-2 Immunotherapy for Malignant Melanoma.” *The Western Journal of Medicine* 152, no. 6 (1990): 714–16.

Birck, R., V. Keim, F. Fiedler, F. J. van der Woude, and P. Rohmeiss. “Pancreatitis after Losartan.” *Lancet (London, England)* 351, no. 9110 (1998): 1178.

Bird, H., and V. Brim. “Propofol and Postoperative Pancreatitis.” *Anaesthesia* 55, no. 5 (2000): 506–7.

Blain, H., V. Baty, A. Blain, P. Trechot, and C. Jeandel. “Acute Pancreatitis Induced by Valpromide: A First Case Report.” *European Journal of Internal Medicine* 10, no. 2 (1999): 117–19.

Blake, William E. D., and Meron E. Pitcher. “Estrogen-Related Pancreatitis in the Setting of Normal Plasma Lipids: Case Report.” *Menopause (New York, N.Y.)* 10, no. 1 (2003): 99–101.

Bories, J. M., P. Bauret, D. Larrey, and H. Michel. “[Acute Pancreatitis and Dexfenfluramine (Isomeride): Apropos of a Case].” *Gastroenterologie Clinique et Biologique* 16, no. 10 (1992): 817–18.

Boruchowicz, Arnaud, Patrice Gallon, David Foissey, Philippe Gower, Claudine Gamblin, Philippe Cuingnet, Vincent Maunoury, Antoine Cortot, and Jean Frederic Colombel. “[Acute Pancreatitis Associated with Corticosteroid Treatment in Crohn’s Disease].” *Gastroenterologie Clinique et Biologique* 27, no. 5 (2003): 560–61.

Bosch, J. A., M. Valdes, J. Oristrell, C. Pigrau, and J. Ordi. “Oxyphenbutazone-Induced Sialadenitis, Intrahepatic Cholestasis and Pancreatitis.” *Acta Gastro-Enterologica Belgica* 48, no. 5 (1985): 529–30.

Bosch, X. “Losartan-Induced Acute Pancreatitis [8].” *Annals of Internal Medicine* 127, no. 11 (1997): 1043–44.

Bosch, X., and O. Bernadich. “Acute Pancreatitis during Treatment with Amiodarone.” *Lancet (London, England)* 350, no. 9087 (1997): 1300.

Bourezane, Hayet, Bruno Kastler, and Jean Pierre Kantelip. “Late and Severe Acute Necrotizing Pancreatitis in a Patient with Liraglutide.” *Therapie* 67, no. 6 (2012): 539–43.

Bouvet, E., E. Casalino, M. H. Prevost, and F. Vachon. “Fatal Case of 2’,3’-Dideoxyinosine-Associated Pancreatitis.” *Lancet (London, England)* 336, no. 8729 (1990): 1515.

Bowers, Riley D., Sara M. Valanejad, and Ashley A. Holombo. “Mirtazapine-Induced Pancreatitis-A Case Report.” *Journal of Pharmacy Practice*, January 1, 2018, 897190018760645. <https://doi.org/10.1177/0897190018760645>.

Boyle, M. P. “Minocycline-Induced Pancreatitis in Cystic Fibrosis.” *Chest* 119, no. 4 (2001): 1283–85.

Briongos-Figuero, L. S., P. Bachiller-Luque, F. Pons-Renedo, and J. M. Eiros-Bouza. “Isoniazid-Induced Acute Pancreatitis [2].” *Enfermedades Infecciosas y Microbiologia Clinica* 25, no. 3 (2007): 217–18.

Bunin, N., W. H. Meyer, M. Christensen, and C. B. Pratt. “Pancreatitis Following Cisplatin: A Case Report.” *Cancer Treatment Reports* 69, no. 2 (1985): 236–37.

Bustamante, Sergio E., and Elumalai Appachi. “Acute Pancreatitis after Anesthesia with Propofol in a Child with Glycogen Storage Disease Type IA.” *Paediatric Anaesthesia* 16, no. 6 (2006): 680–83.

Buszek, S. M., P. Roy-Chaudhury, and G. Yadlapalli. “Olanzapine-Induced Hypertriglyceridemia Resulting in Necrotizing Pancreatitis.” *ACG Case Reports Journal* 3, no. 4 (2016): no.

Buzan, R. D., D. Firestone, M. Thomas, and S. L. Dubovsky. “Valproate-Associated Pancreatitis and Cholecystitis in Six Mentally Retarded Adults.” *The Journal of Clinical Psychiatry* 56, no. 11 (1995): 529–32.

Cadranel, J. F., P. Grippon, F. Lunel, N. Victor, and P. Opolon. “[Ingestion of Tiaprofenic Acid (Surgam) Associated with an Outbreak of Acute Pancreatitis].” *Gastroenterologie Clinique et Biologique* 11, no. 1 (1987): 99–100.

Caldarola, V., J. M. Hassett, A. H. Hall, A. B. Bronstein, K. W. Kulig, and B. H. Rumack. “Hemorrhagic Pancreatitis Associated with Acetaminophen Overdose.” *The American Journal of Gastroenterology* 81, no. 7 (1986): 579–82.

Call, T., W. B. Malarkey, and F. B. Thomas. “Acute Pancreatitis Secondary to Furosemide with Associated Hyperlipidemia.” *The American Journal of Digestive Diseases* 22, no. 9 (1977): 835–38.

Calmus, Y., M. Biour, and F. Bodin. “Indalpine-Induced Hepatitis and Pancreatitis.” *Gastroenterologie Clinique et Biologique* 9, no. 3 (1985): 266–68.

Can, Burak, Mursel Sali, Adnan Batman, Hasan Yilmaz, Ugur Korkmaz, Altay Celebi, Omer Senturk, and Sadettin Hulagu. “Valsartan-Induced Acute Pancreatitis.” *Internal Medicine (Tokyo, Japan)* 53, no. 7 (2014): 703–5.

Canovas, B., D. A. de Luis, P. Beato, and P. Zurita. “[Acute Lithiasic Pancreatitis in Patients Treated with Somatostatin Analogs].” *Revista Clinica Espanola* 200, no. 3 (2000): 182–83.

Cappell, M. S., and K. M. Das. “Rapid Development of Pancreatitis Following Reuse of 6-Mercaptopurine.” *Journal of Clinical Gastroenterology* 11, no. 6 (1989): 679–81.

Carnovale, A., P. Esposito, P. Bassano, L. Russo, and G. Uomo. “Enalapril-Induced Acute Recurrent Pancreatitis.” *Digestive and Liver Disease* 35, no. 1 (2003): 55–57.

Castiella, A., P. Lopez, L. Bujanda, and J. I. Arenas. “Possible Association of Acute Pancreatitis with Naproxen.” *Journal of Clinical Gastroenterology* 21, no. 3 (1995): 258.

Castro, J. L., L. R. Rabago, R. Vello, M. D. Perez, M. Redondo, C. Blesa, and B. Diaz. “[Rifampicin as a Cause of Acute Pancreatitis].” *Revista Espanola de Enfermedades Digestivas : Organo Oficial de La Sociedad Espanola de Patologia Digestiva* 92, no. 12 (2000): 822–23.

Castro, M. R., T. T. Nguyen, and T. O’Brien. “Clomiphene-Induced Severe Hypertriglyceridemia and Pancreatitis.” *Mayo Clinic Proceedings* 74, no. 11 (1999): 1125–28.

Cavanaugh, Zachary, and Edgar R. Naut. “Acetaminophen-Induced Pancreatic Pseudocyst: First Case Report.” *Connecticut Medicine* 78, no. 1 (2014): 37–39.

Cecchi, Enrica, Paolo Forte, Elisabetta Cini, Grazia Banchelli, Chiara Ferlito, and Alessandro Mugelli. “Pancreatitis Induced by Pegylated Interferon Alfa-2b in a Patient Affected by Chronic Hepatitis C.” *Emergency Medicine Australasia : EMA* 16, no. 5–6 (2004): 473–75.

Celifarco, A., C. Warschauer, and R. Burakoff. “Metronidazole-Induced Pancreatitis.” *The American Journal of Gastroenterology* 84, no. 8 (1989): 958–60.

Cerulli, T. R. “Clozapine-Associated Pancreatitis.” *Harvard Review of Psychiatry* 7, no. 1 (1999): 61–63.

Chambon, J. P., B. Dupriez, P. Danjou, M. Provost, F. Bauters, A. Wurtz, and P. Quandalle. “[Acute Necrotic Pancreatitis Secondary to Asparaginase. Role of Drug Combinations--Early Diagnosis and Treatment. Apropos of 2 Cases].” *Journal de Chirurgie* 130, no. 2 (1993): 74–78.

Chams, Sana, Skye El Sayegh, Mulham Hamdon, Sarwan Kumar, and Vesna Tegeltija. “Amoxicillin/Clavulanic Acid-Induced Pancreatitis: Case Report.” *BMC Gastroenterology* 18, no. 1 (August 2, 2018): 122. <https://doi.org/10.1186/s12876-018-0851-6>.

Chan, H. Y., C. M. Ng, S. C. Tiu, A. O. K. Chan, and C. C. Shek. “Hypertriglyceridaemia-Induced Pancreatitis: A Contributory Role of Capecitabine?” *Hong Kong Medical Journal* 18, no. 6 (2012): 526–29.

Chan, K. L., H. S. Chan, S. F. Lui, and K. N. Lai. “Recurrent Acute Pancreatitis Induced by Isoniazid.” *Tubercle and Lung Disease : The Official Journal of the International Union against Tuberculosis and Lung Disease* 75, no. 5 (1994): 383–85.

Chang, T. G., N. Y. Chiu, and W. Y. Hsu. “Acute Pancreatitis Associated with Quetiapine Use in Schizophrenia.” *Journal of Clinical Psychopharmacology* 34, no. 3 (2014): 382–83.

Chao, Chia Ter, and Jia Yu Chao. “Case Report: Furosemide and Pancreatitis: Importance of Dose and Latency Period before Reaction.” *Canadian Family Physician Medecin de Famille Canadien* 59, no. 1 (2013): 43–45.

Charan, V. D., N. Desai, A. P. Singh, and V. P. Choudhry. “Diabetes Mellitus and Pancreatitis as a Complication of L-Asparaginase Therapy.” *Indian Pediatrics* 30, no. 6 (1993): 809–10.

Chen, C. H., M. Y. Lu, K. H. Lin, D. T. Lin, S. F. Peng, and S. T. Jou. “Ureteral Obstruction Caused by L-Asparaginase Induced Pancreatitis in a Child with Acute Lymphoblastic Leukemia.” *Journal of the Formosan Medical Association* 103, no. 5 (2004): 380–84.

Chen, Julie L., Noam Spinowitz, and Manoj Karwa. “Hypertriglyceridemia, Acute Pancreatitis, and Diabetic Ketoacidosis Possibly Associated with Mirtazapine Therapy: A Case Report.” *Pharmacotherapy* 23, no. 7 (2003): 940–44.

Chen, Yen Yuan, Ching Yu Chen, and Kai Kuen Leung. “Acute Pancreatitis and Amiodarone: A Case Report.” *World Journal of Gastroenterology* 13, no. 6 (2007): 975–77.

Chengappa, K. N. R., M. Pelucio, R. W. Baker, and D. Cole. “Recurrent Pancreatitis on Clozapine Re-Challenge.” *Journal of Psychopharmacology* 9, no. 4 (1995): 381–82.

Chetaille, E., R. Delcenserie, T. Yzet, G. Decocq, M. Biour, and M. Andrejak. “[Minocycline Involvement in Two Cases of Acute Pancreatitis].” *Gastroenterologie Clinique et Biologique* 22, no. 5 (1998): 555–56.

Cheung, O., K. Chopra, T. Yu, M. A. Nalesnik, S. Amin, and A. O. Shakil. “Gatifloxacin-Induced Hepatotoxicity and Acute Pancreatitis [9].” *Annals of Internal Medicine* 140, no. 1 (2004): 73–74.

Cheung, Y. F., C. W. Lee, C. F. Chan, K. L. Chan, Y. L. Lau, and C. Y. Yeung. “Somatostatin Therapy in L-Asparaginase-Induced Pancreatitis.” *Medical and Pediatric Oncology* 22, no. 6 (1994): 421–24.

Chhaparia, Anuj, Muhammad B. Hammami, Ashley Vareedayah, and Katie Schroeder. “Eluxadoline-Associated Pancreatitis in a Post-Cholecystectomy Patient: A Case Report.” *Delaware Medical Journal* 89, no. 3 (March 2017): 90–92.

Chintanaboina, J., and D. Gopavaram. “Recurrent Acute Pancreatitis Probably Induced by Rosuvastatin Therapy: A Case Report.” *Case Reports in Medicine* 2012 (2012): no.

Chis, Bogdan Augustin, and Daniela Fodor. “Acute Pancreatitis during GLP-1 Receptor Agonist Treatment. A Case Report.” *Clujul Medical (1957)* 91, no. 1 (2018): 117–19. <https://doi.org/10.15386/cjmed-804>.

Chng, Chiaw Ling, Peng Chin Kek, and Daphne Hsu Chin Khoo. “Carbimazole-Induced Acute Pancreatitis and Cholestatic Hepatitis.” *Endocrine Practice : Official Journal of the American College of Endocrinology and the American Association of Clinical Endocrinologists* 17, no. 6 (2011): 960–61.

Choi, Jong Wook, June Sung Lee, Woo Hyun Paik, Tae Jun Song, Jung Wook Kim, Won Ki Bae, Kyung Ah Kim, and Jung Gon Kim. “Acute Pancreatitis Associated with Pegylated Interferon-Alpha-2a Therapy in Chronic Hepatitis C.” *Clinical and Molecular Hepatology* 22, no. 1 (2016): 168–71.

Chow, K. M., C. C. Szeto, C. B. Leung, and P. K. T. Li. “Recurrent Acute Pancreatitis after Isoniazid.” *The Netherlands Journal of Medicine* 62, no. 5 (2004): 172–74.

Christophe, J. L. “Pancreatitis Induced by Nitrofurantoin.” *Gut* 35, no. 5 (1994): 712–13.

Chung, L. W., S. P. Yeh, C. Y. Hsieh, Y. M. Liao, H. H. Huang, C. Y. Lin, and C. F. Chiu. “Life-Threatening Acute Pancreatitis Due to Thalidomide Therapy for Chronic Graft-versus-Host Disease [3].” *Annals of Hematology* 87, no. 5 (2008): 421–23.

Cina, S. J., and S. E. Conradi. “Acute Pancreatitis in a Prisoner with AIDS. Bugs or Drugs?” *The American Journal of Forensic Medicine and Pathology* 15, no. 1 (1994): 28–31.

Collet, T., C. Even, A. Peytier, M. A. Piquet, T. Dao, and J. C. Verwaerde. “[Acute Pancreatitis and Propylthiouracil].” *Gastroenterologie Clinique et Biologique* 19, no. 11 (1995): 952.

Colomina Aviles, J., L. Quintana Tomas, M. Arenas Gracia, and E. Llorca Martinez. “[Pancreatitis Associated with Cotrimoxazole in an HIV-Positive Patient].” *Anales de Medicina Interna (Madrid, Spain : 1984)* 14, no. 9 (1997): 487–88.

Cooper, M. A., and A. Groll. “A Case of Chronic Pancreatic Insufficiency Due to Valproic Acid in a Child.” *Canadian Journal of Gastroenterology* 15, no. 2 (2001): 127–30.

Cordeiro, Q., Jr., and H. Elkis. “Pancreatitis and Cholestatic Hepatitis Induced by Risperidone.” *Journal of Clinical Psychopharmacology* 21, no. 5 (2001): 529–30.

Corey, W. A., B. N. Doebbeling, K. J. DeJong, and B. E. Britigan. “Metronidazole-Induced Acute Pancreatitis.” *Reviews of Infectious Diseases* 13, no. 6 (1991): 1213–15.

Cortes, E., E. Ribera, E. Cucurull, J. de Otero, I. Ocana, and A. Pahissa. “[Acute Pancreatitis Due to Antimonials in Patients with Visceral Leishmaniasis and HIV Infection].” *Medicina Clinica* 104, no. 15 (1995): 578–80.

Cortes, J., E. Arroyo, S. Reus, E. Climent, and J. Portilla. “Acute Pancreatitis Due to Cotrimoxazole and HIV Infection: A Case Report.” *Farmacia Hospitalaria* 23, no. 5 (1999): 325–26.

Coschieri, M., J. F. Dor, G. Andrieux, J. P. Alessandra, and P. Dulbecco. “[Acute Pancreatitis and Diethylstilbestrol (Distilben)].” *Gastroenterologie Clinique et Biologique* 18, no. 11 (1994): 1040–41.

Couderc, M., P. Blanc, J. M. Rouillon, P. Bauret, D. Larrey, and H. Michel. “[A New Case of Simvastatin-Induced Acute Pancreatitis].” *Gastroenterologie Clinique et Biologique* 15, no. 12 (1991): 986–87.

Coward, R. A. “Paracetamol-Induced Acute Pancreatitis.” *British Medical Journal* 1, no. 6068 (1977): 1086.

Croizet, O., D. Louvel, J. P. Teuliere, L. Buscail, J. Escourrou, and J. Frexinos. “[Acute Pancreatitis Induced by Valproic Acid].” *Gastroenterologie Clinique et Biologique* 18, no. 10 (1994): 910–11.

Csomor, J., I. Murínová, K. Broulíková, O. Kučerka, P. Sedloň, J. Jarošek, P. Urbánek, and M. Zavoral. “Propofol-Induced Acute Pancreatitis.” *Journal of Clinical Pharmacy and Therapeutics* 42, no. 4 (August 2017): 495–98. <https://doi.org/10.1111/jcpt.12524>.

Dabaghi, S. “ACE Inhibitors and Pancreatitis.” *Annals of Internal Medicine* 115, no. 4 (1991): 330–31.

Daniel, F., P. Seksik, W. Cacheux, R. Jian, and P. Marteau. “Tolerance of 4-Aminosalicylic Acid Enemas in Patients with Inflammatory Bowel Disease and 5-Aminosalicylic-Induced Acute Pancreatitis.” *Inflammatory Bowel Diseases* 10, no. 3 (2004): 258–60.

Das, Anup K., and Qaiser Jawed. “Drug-Induced Acute Pancreatitis: A Rare Manifestation of an Incomplete ‘Dapsone Syndrome.’” *Indian Journal of Pharmacology* 46, no. 4 (2014): 455–57.

Das, Saibal, Abhrajit Ganguly, Abhinaba Ghosh, Somnath Mondal, Jayanta Kumar Dey, and Indranil Saha. “Oral Pantoprazole-Induced Acute Pancreatitis in an 11-Year-Old Child.” *Therapeutic Drug Monitoring* 34, no. 3 (2012): 242–44.

Das, Saibal, Somnath Mondal, Jayanta Kumar Dey, Sanjib Bandyopadhyay, Indranil Saha, and Santanu Kumar Tripathi. “A Case of Montelukast Induced Hypercholesterolemia, Severe Hypertriglyceridemia and Pancreatitis.” *Journal of Young Pharmacists : JYP* 5, no. 2 (2013): 64–66.

Davido, B., J. Shourick, S. Makhloufi, A. Dinh, and J. Salomon. “True Incidence of Tigecycline-Induced Pancreatitis: How Many Cases Are We Missing?” *Journal of Antimicrobial Chemotherapy* 71, no. 10 (2016): 2994–95.

De Gennes, J. L., F. Dairou, and B. Surbled-Delas. “[Demonstration of the Role of Cholelithiasis in the Onset of Acute Pancreatitis Occurring during Clofibrate (or Its Analog) Treatment of Atherogenic Hyperlipemia].” *Annales de Medecine Interne* 129, no. 6–7 (1978): 435–39.

De Lalla, F., G. Pellizzer, L. Gradoni, M. Vespignani, M. Franzetti, and C. Stecca. “Acute Pancreatitis Associated with the Administration of Meglumine Antimonate for the Treatment of Visceral Leishmaniasis [7].” *Clinical Infectious Diseases* 16, no. 5 (1993): 730–31.

De Mesa, C., M. A. Dajoyag-Mejia, I. Regina, and R. O. Darouiche. “Tigecycline-Induced Acute Pancreatitis with Rechallenge: A Case Report.” *Journal of Pharmacy Technology* 29, no. 1 (2013): 3–8.

Debongnie, J. C., and X. Dekoninck. “Sulfasalazine, 5-ASA and Acute Pancreatitis in Crohn’s Disease [2].” *Journal of Clinical Gastroenterology* 19, no. 4 (1994): 348–49.

Decocq, G., V. Gras-Champel, C. Vrolant-Mille, R. Delcenserie, L. Sauve, H. Masson, and M. Andrejak. “[Acute Pancreatitis Induced by Drugs Derived from 5-Aminosalicylic Acid: Case Report and Review of the Literature].” *Therapie* 54, no. 1 (1999): 41–48.

Delgado Fontaneda, E., F. Garcia Campos, L. Ruiz Rebollo, B. Ibarra Pena, and M. Moreto Canela. “[Acute Pancreatitis Caused by Salazopyrine. An Unusual Association].” *Revista Espanola de Enfermedades Digestivas : Organo Oficial de La Sociedad Espanola de Patologia Digestiva* 79, no. 6 (1991): 439–40.

Denker, P. S., and P. E. Dimarco. “Exenatide (Exendin-4)-Induced Pancreatitis: A Case Report [13].” *Diabetes Care* 29, no. 2 (2006): 471.

Deprez, P., C. Descamps, and R. Fiasse. “Pancreatitis Induced by 5-Aminosalicylic Acid.” *Lancet (London, England)* 2, no. 8660 (1989): 445–46.

Deshpande, P. R., K. Khera, G. Thunga, M. Hande, S. T. G. Gouda, and A. N. Nagappa. “Atorvastatin-Induced Acute Pancreatitis.” *Journal of Pharmacology and Pharmacotherapeutics* 2, no. 1 (2011): 40–42.

Devars du Mayne, J. F., E. Bouchacourt, and J. P. Hardouin. “[Acute Pancreatitis Cholestatic Hepatitis Induced by Estroprogestatives].” *La Nouvelle Presse Medicale* 9, no. 46 (1980): 3550–51.

Di Martino, V., J. Ezenfis, Y. Benhamou, B. Bernard, P. Opolon, F. Bricaire, and T. Poynard. “Severe Acute Pancreatitis Related to the Use of Nelfinavir in HIV Infection: Report of a Case with Positive Rechallenge.” *AIDS (London, England)* 13, no. 11 (1999): 1421–23.

Diaz, Javier, Milagros Davalos, Rossana Roman, Carla Bustios, and Eduardo Zumaeta. “[Hepatotoxicity and Pancreatitis Associated with Gold Salts: Case Report].” *Revista de Gastroenterologia Del Peru : Organo Oficial de La Sociedad de Gastroenterologia Del Peru* 24, no. 4 (2004): 353–56.

Domingo, P., S. Ferrer, L. Kolle, C. Munoz, and P. Rodriguez. “Acute Pancreatitis Associated with Sodium Stibogluconate Treatment in a Patient with Human Immunodeficiency Virus [5].” *Archives of Internal Medicine* 156, no. 9 (1996): 1029.

Donovan, K. L., A. D. White, D. A. Cooke, and D. J. Fisher. “Pancreatitis and Palindromic Arthropathy with Effusions Associated with Sodium Stibogluconate Treatment in a Renal Transplant Recipient.” *The Journal of Infection* 21, no. 1 (1990): 107–10.

Doucette, D. E., J. P. Grenier, and P. S. Robertson. “Olanzapine-Induced Acute Pancreatitis.” *The Annals of Pharmacotherapy* 34, no. 10 (2000): 1128–31.

Drabo, Y. J., A. Niakara, and H. Ouedraogo. “[Acute Pancreatitis Secondary to Administration or Norfloxacin].” *Annales Francaises d’anesthesie et de Reanimation* 21, no. 1 (2002): 68–69.

Drory, V. E., I. Sidi, and A. D. Korczyn. “Riluzole-Induced Pancreatitis.” *Neurology* 52, no. 4 (1999): 892–93.

Du Ville, L., S. Debeuckelaere, H. Reynaert, and G. Devis. “Pancreatitis Associated with Naproxen.” *The American Journal of Gastroenterology* 88, no. 3 (1993): 464.

Duboeuf, T., A. De Widerspach-Thor, B. Scotto, and Y. Bacq. “Acute Glimepiride-Induced Pancreatitis.” *Gastroenterologie Clinique et Biologique* 28, no. 4 (2004): 409–10.

Echarri, A., F. Borda, F. J. Jimenez, A. Arin, I. Martin-Granizo, and R. Aznarez. “[Acute Pancreatitis Caused by Azathioprine in Patient with Crohn Disease].” *Revista Espanola de Enfermedades Digestivas : Organo Oficial de La Sociedad Espanola de Patologia Digestiva* 88, no. 9 (1996): 645–46.

Echinard, E., M. Dupon, M. Malou, J. M. Ragnaud, J. Y. Lacut, and H. Albin. “[Acute Fatal Pancreatitis Following Treatment with Pentamidine].” *Therapie* 41, no. 6 (1986): 520.

Eddoukani, I., S. Oubaha, Z. Samlani, and K. Krati. “Pancréatite aiguë secondaire à la 6-mercaptopurine : à propos d’un cas.” *Journal Africain d’Hépato-Gastroentérologie* 10, no. 4 (December 2016): 220–22. <https://doi.org/10.1007/s12157-016-0679-z>.

Einollahi, Behzad, and Fardin Dolatimehr. “Acute Pancreatitis Induced by Mycophenolate Mofetil in a Kidney Transplant Patient.” *Journal of Nephropharmacology* 4, no. 2 (2015): 72–74.

Eisemann, A. D., N. J. Becker, P. B. J. Miner, and J. Fleming. “Pancreatitis and Gold Treatment of Rheumatoid Arthritis.” *Annals of Internal Medicine* 111, no. 10 (1989): 860–61.

Eland, I. A., E. P. van Puijenbroek, M. J. Sturkenboom, J. H. Wilson, and B. H. Stricker. “Drug-Associated Acute Pancreatitis: Twenty-One Years of Spontaneous Reporting in The Netherlands.” *The American Journal of Gastroenterology* 94, no. 9 (1999): 2417–22.

Eland, I. A., M. C. Rasch, M. J. Sturkenboom, F. C. Bekkering, J. T. Brouwer, J. Delwaide, J. Belaiche, G. Houbiers, and B. H. Stricker. “Acute Pancreatitis Attributed to the Use of Interferon Alfa-2b.” *Gastroenterology* 119, no. 1 (2000): 230–33.

Elisaf, M. S., K. Nakou, G. Liamis, and N. A. Pavlidis. “Tamoxifen-Induced Severe Hypertriglyceridemia and Pancreatitis.” *Annals of Oncology : Official Journal of the European Society for Medical Oncology* 11, no. 8 (2000): 1067–69.

Elmore, M. F., and J. D. Rogge. “Tetracycline-Induced Pancreatitis.” *Gastroenterology* 81, no. 6 (1981): 1134–36.

Engel, Tal, Dan Justo, Michal Amitai, Yulia Volchek, and Haim Mayan. “Nilotinib-Associated Acute Pancreatitis.” *The Annals of Pharmacotherapy* 47, no. 1 (2013): e3.

Erdkamp, F., M. Houben, E. Ackerman, W. Breed, and J. van Spreeuwel. “Pancreatitis Induced by Mesalamine.” *The Netherlands Journal of Medicine* 41, no. 1–2 (1992): 71–73.

Erenoglu, Cengiz, Ahmet Haldun Uluutku, Cihan Top, Mehmet Levhi Akin, and Tuncay Celenk. “Do MRI Agents Cause or Worsen Acute Pancreatitis?” *Ulusal Travma ve Acil Cerrahi Dergisi = Turkish Journal of Trauma & Emergency Surgery : TJTES* 13, no. 1 (2007): 78–79.

Evans, R. J., R. N. Miranda, J. Jordan, and F. J. Krolikowski. “Fatal Acute Pancreatitis Caused by Valproic Acid.” *The American Journal of Forensic Medicine and Pathology* 16, no. 1 (1995): 62–65.

Faintuch, J., C. B. Mott, and M. C. Machado. “Pancreatitis and Pancreatic Necrosis during Sulfasalazine Therapy.” *International Surgery* 70, no. 3 (1985): 271–72.

Falko, J. M., and F. B. Thomas. “Letter: Acute Pancreatitis Due to Procainamide-Induced Lupus Erythematosus.” *Annals of Internal Medicine* 83, no. 6 (1975): 832–33.

Famularo, G., C. Bizzarri, and G. C. Nicotra. “Acute Pancreatitis Caused by Ketorolac Tromethamine [3].” *Journal of Clinical Gastroenterology* 34, no. 3 (2002): 283–84.

Famularo, G., Simone C. De, G. Minisola, and G. C. Nicotra. “Cross-Reaction Allergic Pancreatitis with Ketoprofen and Flurbiprofen [1].” *Pancreas* 35, no. 2 (2007): 187–88.

Famularo, G., L. Gasbarrone, and G. Minisola. “Pancreatitis during Treatment with Liraglutide.” *Journal of the Pancreas* 13, no. 5 (2012): 540–41.

Famularo, G., G. Minisola, G. C. Nicotra, and Simone C. De. “Acute Pancreatitis Associated with Irbesartan Therapy [3].” *Pancreas* 31, no. 3 (2005): 294–95.

Famularo, G., S. Polchi, and C. De Simone. “Acute Cholecystitis and Pancreatitis in a Patient with Biliary Sludge Associated with the Use of Ceftriaxone: A Rare but Potentially Severe Complication.” *Annali Italiani Di Medicina Interna : Organo Ufficiale Della Societa Italiana Di Medicina Interna* 14, no. 3 (1999): 202–4.

Famularo, Giuseppe, Giovanni Minisola, Giulio Cesare Nicotra, and Claudio De Simone. “Acute Pancreatitis Caused by Amiodarone.” *European Journal of Emergency Medicine : Official Journal of the European Society for Emergency Medicine* 11, no. 5 (2004): 305–6.

———. “Idiosyncratic Pancreatitis Associated with Perindopril.” *JOP : Journal of the Pancreas* 6, no. 6 (2005): 605–7.

Fang, C. C., H. P. Wang, and J. T. Lin. “Erythromycin-Induced Acute Pancreatitis.” *Journal of Toxicology.Clinical Toxicology* 34, no. 1 (1996): 93–95.

Fathallah, Neila, Michele Zamy, Raoudha Slim, Olivier Fain, Houssem Hmouda, Kamel Bouraoui, Chaker Ben Salem, and Michel Biour. “Acute Pancreatitis in the Course of Meprobamate Poisoning.” *JOP : Journal of the Pancreas* 12, no. 4 (2011): 404–6.

Fecik, S. E., S. C. Stoner, J. Raphael, and C. Lindsey. “Recurrent Acute Pancreatitis Associated with Valproic Acid Use for Mood Stabilization [9].” *Journal of Clinical Psychopharmacology* 19, no. 5 (1999): 483–84.

Felig, D. M., and M. Topazian. “Corticosteroid-Induced Pancreatitis.” *Annals of Internal Medicine* 124, no. 11 (1996): 1016.

Feola, David J., and Alice C. Thornton. “Metronidazole-Induced Pancreatitis in a Patient with Recurrent Vaginal Trichomoniasis.” *Pharmacotherapy* 22, no. 11 (2002): 1508–10.

Fernandes, Roland. “Acute Pancreatitis Following Paracetamol Overdose.” *BMJ Case Reports* 2009 (2009). <http://ovidsp.ovid.com/ovidweb.cgi?T=JS&PAGE=reference&D=prem&NEWS=N&AN=22096469>.

Fernandez, J., M. Sala, J. Panes, F. Feu, S. Navarro, and J. Teres. “Acute Pancreatitis after Long-Term 5-Aminosalicylic Acid Therapy.” *The American Journal of Gastroenterology* 92, no. 12 (1997): 2302–3.

Fernandez Lison, L. C., P. Perez Puente, M. T. Martin Cillero, and M. R. Garrido Ameigeiras. “[Acute Secondary Pancreatitis to Azathioprine versus Autoimmune Pancreatitis in a Patient with Ulcerative Colitis].” *Farmacia Hospitalaria : Organo Oficial de Expresion Cientifica de La Sociedad Espanola de Farmacia Hospitalaria* 34, no. 6 (2010): 310–12.

Fiorentini, M. T., M. Fracchia, G. Galatola, A. Barlotta, and M. de la Pierre. “Acute Pancreatitis during Oral 5-Aminosalicylic Acid Therapy.” *Digestive Diseases and Sciences* 35, no. 9 (1990): 1180–82.

Flaig, T., A. Douros, E. Bronder, A. Klimpel, R. Kreutz, and E. Garbe. “Tocilizumab-Induced Pancreatitis: Case Report and Review of Data from the FDA Adverse Event Reporting System.” *Journal of Clinical Pharmacy and Therapeutics* 41, no. 6 (2016): 718–21.

Flamenbaum, M., A. Abergel, N. Marcato, M. Zenut, J. L. Kemeny, and P. Cassan. “[Regressive Fulminant Hepatitis, Acute Pancreatitis and Renal Insufficiency after Taking Ketoprofen].” *Gastroenterologie Clinique et Biologique* 22, no. 11 (1998): 975–76.

Floris-Moore, M. A., M. I. Amodio-Groton, and M. T. Catalano. “Adverse Reactions to Trimethoprim/Sulfamethoxazole in AIDS.” *Annals of Pharmacotherapy* 37, no. 12 (2003): 1810–13.

Flynn, W. J., P. G. Freeman, and L. G. Wickboldt. “Pancreatitis Associated with Isotretinoin-Induced Hypertriglyceridemia.” *Annals of Internal Medicine* 107, no. 1 (1987): 63.

Foisy, M. M., K. L. Slayter, R. G. Hewitt, and G. D. Morse. “Pancreatitis during Intravenous Pentamidine Therapy in an AIDS Patient with Prior Exposure to Didanosine.” *The Annals of Pharmacotherapy* 28, no. 9 (1994): 1025–28.

Forte, A., L. Gallinaro, G. Montesano, R. Turano, A. Bertagni, and G. Illuminati. “A Possible Case of Carbamazepine Induced Pancreatitis.” *Rivista Europea per Le Scienze Mediche e Farmacologiche = European Review for Medical and Pharmacological Sciences = Revue Europeenne Pour Les Sciences Medicales et Pharmacologiques* 18, no. 5–6 (1996): 187–89.

Francobandiera, G., G. Rondalli, and P. Telattin. “Acute Pancreatitis Associated with Clothiapine Use.” *Human Psychopharmacology* 14, no. 3 (1999): 211–12.

Franga, D. L., and J. A. Harris. “Polyethylene Glycol-Induced Pancreatitis.” *Gastrointestinal Endoscopy* 52, no. 6 (2000): 789–91.

Fredenrich, A., C. Sosset, J. L. Bernard, J. L. Sadoul, and P. Freychet. “Acute Pancreatitis after Short-Term Octreotide [6].” *Lancet (London, England)* 338, no. 8758 (1991): 52–53.

Fuchs, J. E. J., M. R. Keith, and A. N. Galanos. “Probable Metolazone-Induced Pancreatitis.” *DICP : The Annals of Pharmacotherapy* 23, no. 9 (1989): 711.

Funayama, Y., K. Fukushima, C. Shibata, K. Koyama, K. Miura, K. Takahashi, A. Hashimoto, et al. “Acute Pancreatitis Complicating Ulcerative Colitis under Administration of Corticosteroid in Surgical Cases [3].” *Journal of Gastroenterology* 39, no. 6 (2004): 592–94.

Gabriel, Joseph Gabriel, Sukhdeep Bhogal, and Aaysha Kapila. “Minocycline-Associated Pancreatitis.” *American Journal of Therapeutics* 25, no. 5 (October 2018): e556–57. <https://doi.org/10.1097/MJT.0000000000000635>.

Galindo, C., J. Buenestado, J. M. Rene Espinet, and M. C. Pinol. “Acute Pancreatitis and Liver Injury Associated with Amoxicillin-Clavulanic Therapy.” *Revista Espanola de Enfermedades Digestivas* 87, no. 8 (1995): 597–600.

Gallego-Gutierrez, Silvia, Victor Manuel Navas-Lopez, Michal Kolorz, Ladislava Bartosova, Katerina Lukac, Silvia Luque-Perez, Leticia Nunez-Caro, et al. “Successful Mercaptopurine Usage despite Azathioprine-Induced Pancreatitis in Paediatric Crohn’s Disease.” *Journal of Crohn’s & Colitis* 9, no. 8 (2015): 676–79.

Gallego-Rojo, F. J., J. L. Gonzalez-Calvin, J. Guilarte, F. J. Casado-Caballero, and V. Bellot. “Perindopril-Induced Acute Pancreatitis.” *Digestive Diseases and Sciences* 42, no. 8 (1997): 1789–91.

Gang, N., P. Langevitz, and A. Livneh. “Relapsing Acute Pancreatitis Induced by Re-Exposure to the Cholesterol Lowering Agent Bezafibrate.” *The American Journal of Gastroenterology* 94, no. 12 (1999): 3626–28.

Garau, P., S. R. Orenstein, D. A. Neigut, and S. A. Kocoshis. “Pancreatitis Associated with Olsalazine and Sulfasalazine in Children with Ulcerative Colitis.” *Journal of Pediatric Gastroenterology and Nutrition* 18, no. 4 (1994): 481–85.

Garcia Aguilera, Xavier, Carlos Teruel Sanchez-Vegazo, Laura Crespo Perez, and Victor Moreira Vicente. “[Orlistat-Induced Acute Pancreatitis].” *Medicina Clinica* 130, no. 14 (2008): 557.

Garg, Rajan, Sandeep Agarwala, and Veereshwar Bhatnagar. “Acute Pancreatitis Induced by Ifosfamide Therapy.” *Journal of Pediatric Surgery* 45, no. 10 (2010): 2071–73.

Garrington, T., D. Bensard, J. D. Ingram, and C. C. Silliman. “Successful Management with Octreotide of a Child with L-Asparaginase Induced Hemorrhagic Pancreatitis.” *Medical and Pediatric Oncology* 30, no. 2 (1998): 106–9.

Gasser, Jr., A. J. Magill, C. N. Oster, E. D. Franke, M. Grogl, and J. D. Berman. “Pancreatitis Induced by Pentavalent Antimonial Agents during Treatment of Leishmaniasis.” *Clinical Infectious Diseases* 18, no. 1 (1994): 83–90.

Gatto, E. M. “Clozapine and Pancreatitis.” *Clinical Neuropharmacology* 21, no. 3 (1998): 203.

Gerson, R., A. Serrano, A. Villalobos, G. L. Sternbach, and J. Varon. “Acute Pancreatitis Secondary to Ifosfamide.” *The Journal of Emergency Medicine* 15, no. 5 (1997): 645–47.

Gill, C. J., A. E. Jennings, J. B. Newton, and D. E. Schwartz. “Fatal Acute Pancreatitis in a Patient Chronically Treated with Candesartan.” *Journal of Pharmacy Technology* 21, no. 2 (2005): 79–82.

Gilmore, I. T., and E. Tourvas. “Paracetamol-Induced Acute Pancreatitis.” *British Medical Journal* 1, no. 6063 (1977): 753–54.

Gilson, Melanie, Laurence Moachon, Luc Jeanne, Valerie Dumaine, Luc Eyrolle, Philippe Morand, Mona Ben m’Rad, and Dominique Salmon. “Acute Pancreatitis Related to Tigecycline: Case Report and Review of the Literature.” *Scandinavian Journal of Infectious Diseases* 40, no. 8 (2008): 681–83.

Girgis, Christian M., and Bernard L. Champion. “Vildagliptin-Induced Acute Pancreatitis.” *Endocrine Practice : Official Journal of the American College of Endocrinology and the American Association of Clinical Endocrinologists* 17, no. 3 (2011): e48–50.

Godino, J., R. C. Butani, P. W. K. Wong, and F. T. Murphy. “Acute Drug-Induced Pancreatitis Associated with Celecoxib [5].” *Journal of Clinical Rheumatology* 5, no. 5 (1999): 305–7.

Goffin, E., Y. Horsmans, Y. Pirson, C. Cornu, A. Geubel, and C. van Ypersele De Strihou. “Acute Necrotico-Hemorrhagic Pancreatitis after Famciclovir Prescription.” *Transplantation* 59, no. 8 (1995): 1218–19.

Goldberg, B. H., and J. M. Bergstein. “Acute Respiratory Distress in a Child after Steroid-Induced Pancreatitis.” *Pediatrics* 61, no. 2 (1978): 317–18.

Goldman, Kim E., Melanie K. Marshall, Edward Alessandrini, and Mark L. Bernstein. “Complications of Alpha-Interferon Therapy for Aggressive Central Giant Cell Lesion of the Maxilla.” *Oral Surgery, Oral Medicine, Oral Pathology, Oral Radiology, and Endodontics* 100, no. 3 (2005): 285–91.

Gonzalez Carro, P., F. Ribes, and M. J. Garcia. “Acute Pancreatitis Associated with Erythromycin [2].” *Revista Espanola de Enfermedades Digestivas* 87, no. 10 (1995): 757–58.

Gonzalez Carro, Pedro, Francisco Perez Roldan, Maria Luisa Legaz Huidobro, Manuel Moraleda de Acuna, and Juan Carlos Nieto Garcia. “Acute Pancreatitis and Modified-Release Clarithromycin.” *The Annals of Pharmacotherapy* 38, no. 3 (2004): 508–9.

Gonzalez Ramallo, V. J., A. Muino Miguez, and F. J. Torres Segovia. “Necrotizing Pancreatitis and Enalapril.” *The European Journal of Medicine* 1, no. 2 (1992): 123.

Gottschling, Sven, Reinhard Larsen, Sascha Meyer, Norbert Graf, and Harald Reinhard. “Acute Pancreatitis Induced by Short-Term Propofol Administration.” *Paediatric Anaesthesia* 15, no. 11 (2005): 1006–8.

Goyal, S. B., and R. S. Goyal. “Ketorolac Tromethamine-Induced Acute Pacreatitis [1].” *Archives of Internal Medicine* 158, no. 4 (1998): 411.

Gradon, J. D., R. H. Schulman, E. K. Chapnick, and D. V. Sepkowitz. “Octreotide-Induced Acute Pancreatitis in a Patient with Acquired Immunodeficiency Syndrome.” *Southern Medical Journal* 84, no. 11 (1991): 1410–11.

Granados, L. M., F. Ballester, F. Suarez, J. Sanchez-Pena, and E. Navas. “[Acute Postoperative Pancreatitis, Is It Associated with the Use of Ondansetron?].” *Revista Espanola de Anestesiologia y Reanimacion* 44, no. 2 (1997): 87.

Grauso-Eby, Nancy L., Olga Goldfarb, Lori B. Feldman-Winter, and Gary N. McAbee. “Acute Pancreatitis in Children from Valproic Acid: Case Series and Review.” *Pediatric Neurology* 28, no. 2 (February 2003): 145–48.

Greenstein, R., C. Nogeire, T. Ohnuma, and A. Greenstein. “Management of Asparaginase Induced Hemorrhagic Pancreatitis Complicated by Pseudocyst.” *Cancer* 43, no. 2 (1979): 718–22.

Guerra, M. “Toxicity of Indomethacin. Report of a Case of Acute Pancreatitis.” *JAMA* 200, no. 6 (1967): 552–53.

Guerrero Igea, F. J., J. A. Lepe Jimenez, A. Garrido Serrano, and S. Palomo Gil. “[Acute Pancreatitis Caused by Erythromycin].” *Anales de Medicina Interna (Madrid, Spain : 1984)* 18, no. 7 (2001): 400.

Guevara-Campos, Jose, Lucia Gonzalez-Guevara, Ixora Vacaro-Bolivar, and Juan Manuel Rojas. “Acute Pancreatitis Associated to the Use of Valproic Acid.” *Arquivos de Neuro-Psiquiatria* 67, no. 2B (2009): 513–15.

Guillaume, P., E. Grandjean, and P. J. Male. “Azathioprine-Associated Acute Pancreatitis in the Course of Chronic Active Hepatitis.” *Digestive Diseases and Sciences* 29, no. 1 (1984): 78–79.

Gumaste, V. V. “Erythromycin-Induced Pancreatitis.” *The American Journal of Medicine* 86, no. 6 Pt 1 (1989): 725.

Gupta, Ankur, Uday C. Ghoshal, Samir Mohindra, and Vivek A. Saraswat. “Acute Necrotizing Pancreatitis Following Olanzapine Therapy.” *Tropical Gastroenterology : Official Journal of the Digestive Diseases Foundation* 35, no. 2 (2014): 132–34.

Hainaut, P., M. Schapira, P. Mugabo, G. Cerulus, and E. Coche. “Cimetidine-Induced Acute Pancreatitis.” *Revue de Medecine Interne* 8, no. 5 (1987): 516–18.

Halim, M. A., O. Alfurayh, M. E. Kalin, S. Dammas, A. Al-Eisa, and G. Damanhouri. “Successful Treatment of Visceral Leishmaniasis with Allopurinol plus Ketoconazole in a Renal Transplant Recipient after the Occurrence of Pancreatitis Due to Stibogluconate.” *Clinical Infectious Diseases* 16, no. 3 (1993): 397–99.

Hamed, I., R. D. Lindeman, and A. W. Czerwinski. “Case Report: Acute Pancreatitis Following Corticosteroid and Azathioprine Therapy.” *The American Journal of the Medical Sciences* 276, no. 2 (1978): 211–19.

Hanft, Alan, and James Bourgeois. “Risperidone and Pancreatitis.” *Journal of the American Academy of Child and Adolescent Psychiatry* 43, no. 12 (2004): 1458–59.

Hanline, M. H. J. “Acute Pancreatitis Caused by Ampicillin.” *Southern Medical Journal* 80, no. 8 (1987): 1069.

Hart, C. C. “Aerosolized Pentamidine and Pancreatitis.” *Annals of Internal Medicine* 111, no. 8 (1989): 691.

Hastier, P., M. J. Buckley, E. P. Peten, N. Demuth, R. Dumas, J. F. Demarquay, F. X. Caroli-Bosc, and J. P. Delmont. “A New Source of Drug-Induced Acute Pancreatitis: Codeine.” *The American Journal of Gastroenterology* 95, no. 11 (2000): 3295–98.

Hawksworth, C. R. “Acute Pancreatitis Associated with Infusion of Erythromycin Lactobionate.” *BMJ (Clinical Research Ed.)* 298, no. 6667 (1989): 190.

Hegazi, M. O., F. Saleh, and J. E. John. “Is It Tinidazole-Induced Pancreatitis?” *Journal of Clinical Pharmacy and Therapeutics* 40, no. 5 (2015): 607–8.

Heluwaert, Frederic, Joanna Pofelski, Emmanuel Germain, and Xavier Roblin. “[Piroxicam and Acute Pancreatitis].” *Gastroenterologie Clinique et Biologique* 30, no. 4 (2006): 635–36.

Herer, B., T. Chinet, S. Labrune, M. A. Collignon, J. Chretien, and G. Huchon. “Pancreatitis Associated with Pentamidine by Aerosol.” *BMJ (Clinical Research Ed.)* 298, no. 6673 (1989): 605.

Herrmann, R., R. G. Shaw, and D. J. Fone. “Ranitidine-Associated Recurrent Acute Pancreatitis.” *Australian and New Zealand Journal of Medicine* 20, no. 3 (1990): 243–44.

Herskowitz, L. J., S. Olansky, and P. G. Lang. “Acute Pancreatitis Associated with Long-Term Azathioprine Therapy. Occurrence in a Patient with Systemic Lupus Erythematosus.” *Archives of Dermatology* 115, no. 2 (1979): 179.

Hochain, P., C. Guedon, and R. Colin. “Acute Pancreatitis as a Complication of Crohn’s Disease Treated by Oral Mesalazine [6].” *Gastroenterologie Clinique et Biologique* 15, no. 2 (1991): 173–74.

Holla, S., B. Ommurugan, D. Amita, K. L. Bairy, K. Saravu, and J. Madireddi. “A Rare Case of Cotrimoxazole Induced Acute Pancreatitis, Acute Kidney Injury and Crystalluria (APAKIC).” *Research Journal of Pharmaceutical, Biological and Chemical Sciences* 7, no. 1 (2016): 1099–1102.

Houben, Michiel L., Ingeborg Wilting, Hans Stroink, and Pieter J. van Dijken. “Pancreatitis, Complicated by a Pancreatic Pseudocyst Associated with the Use of Valproic Acid.” *European Journal of Paediatric Neurology : EJPN : Official Journal of the European Paediatric Neurology Society* 9, no. 2 (2005): 77–80.

Hoyte, F. C. L., R. W. Weber, and R. K. Katial. “Pancreatitis as a Novel Complication of Aspirin Therapy in Patients with Aspirin-Exacerbated Respiratory Disease.” *Journal of Allergy and Clinical Immunology* 129, no. 6 (2012): 1684–86.

Huang, Yu Jhen, Hsien Yuan Lane, Chun Hui Liao, and Chih Chia Huang. “Recurrent Pancreatitis without Eosinophilia on Clozapine Rechallenge.” *Progress in Neuro-Psychopharmacology & Biological Psychiatry* 33, no. 8 (2009): 1561–62.

Hung, M. C., G. Y. Hung, P. C. Lin, C. M. Tiu, and Y. C. Tien. “Acute Pancreatitis Associated with Ifosfamide.” *Journal of the Chinese Medical Association* 70, no. 4 (2007): 176–79.

Hung, Whitney Y., Laura Kogelman, Gretchen Volpe, Mark Iafrati, and Lisa Davidson. “Tigecycline-Induced Acute Pancreatitis: Case Report and Literature Review.” *International Journal of Antimicrobial Agents* 34, no. 5 (2009): 486–89.

Hussain, Abrar, and John Burke. “Mirtazapine Associated with Recurrent Pancreatitis - a Case Report.” *Journal of Psychopharmacology (Oxford, England)* 22, no. 3 (2008): 336–37.

Ianiro, G., G. Cammarota, A. Milani, M. Mettimano, and A. Gasbarrini. “Moderately Severe Acute Pancreatitis Associated with Riluzole.” *Journal of Clinical Gastroenterology* 48, no. 6 (2014): 563.

Igarashi, Hisato, Tetsuhide Ito, Masahiro Yoshinaga, Takamasa Oono, Hiroyuki Sakai, and Ryoichi Takayanagi. “Acetaminophen-Induced Acute Pancreatitis. A Case Report.” *JOP : Journal of the Pancreas* 10, no. 5 (2009): 550–53.

Ikeuchi, K., Y. Okuma, and T. Tabata. “Immune-Related Pancreatitis Secondary to Nivolumab in a Patient with Recurrent Lung Adenocarcinoma: A Case Report.” *Lung Cancer* 99 (2016): 148–50.

Iliopoulou, A., G. Giannakopoulos, H. Pagoy, T. Christos, and S. Theodore. “Acute Pancreatitis Due to Captopril Treatment.” *Digestive Diseases and Sciences* 46, no. 9 (2001): 1882–83.

Im, Moon Sun, Hyo Suk Ahn, Hyun Jai Cho, Ki Bong Kim, and Hae Young Lee. “Diabetic Ketoacidosis Associated with Acute Pancreatitis in a Heart Transplant Recipient Treated with Tacrolimus.” *Experimental and Clinical Transplantation : Official Journal of the Middle East Society for Organ Transplantation* 11, no. 1 (2013): 72–74.

Inayat, Faisal, Hafeez Ul Hassan Virk, Daniel J. Yoon, and Iqra Riaz. “Drug-Induced Pancreatitis: A Rare Manifestation of Doxycycline Administration.” *North American Journal of Medical Sciences* 8, no. 2 (2016): 117–20.

Inoue, Hidekazu, Katsuya Shiraki, Hiroshi Okano, Masatoshi Deguchi, Takenari Yamanaka, Takahisa Sakai, Shigeru Ohmori, Hitoshi Yoshimura, and Takeshi Nakano. “Acute Pancreatitis in Patients with Ulcerative Colitis.” *Digestive Diseases and Sciences* 50, no. 6 (2005): 1064–67.

Isenberg, J. N. “Pancreatitis, Amylase Clearance, and Azathioprine.” *The Journal of Pediatrics* 93, no. 6 (1978): 1043–44.

Izraeli, S., P. C. Adamson, S. M. Blaney, and F. M. Balis. “Acute Pancreatitis after Ifosfamide Therapy.” *Cancer* 74, no. 5 (1994): 1627–28.

Izzedine, H., V. Launay-Vacher, T. Storme, and G. Deray. “Acute Pancreatitis Induced by Isoniazid.” *The American Journal of Gastroenterology* 96, no. 11 (2001): 3208–9.

Jain, N., M. Savani, M. Agarwal, and C. W. Sands. “Albiglutide-Induced Pancreatitis.” *Therapeutic Advances in Drug Safety* 7, no. 6 (2016): 236–38.

Jamshidi, Mohammad, Robert J. Obermeyer, Satish Govindaraj, Armand Garcia, and Abdul Ghani. “Acute Pancreatitis Secondary to Isotretinoin-Induced Hyperlipidemia.” *The Journal of the Oklahoma State Medical Association* 95, no. 2 (2002): 79–80.

Javaloyas, M., and T. Casasin. “Acute Pancreatitis by Carbamazepine in an AIDS Patient [1].” *Medicina Clinica* 110, no. 11 (1998): 437.

Jawaid, Q., M. E. Presti, B. A. Neuschwander-Tetri, and F. R. Burton. “Case Report: Acute Pancreatitis after Single-Dose Exposure to Propofol: A Case Report and Review of Literature.” *Digestive Diseases and Sciences* 47, no. 3 (2002): 614–18.

Jeandidier, N., M. Klewansky, and M. Pinget. “Captopril-Induced Acute Pancreatitis.” *Diabetes Care* 18, no. 3 (1995): 410–11.

Jetha, Mary M., and Loretta Fiorillo. “Xanthomata and Diabetes in an Adolescent with Familial Dysbetalipoproteinemia 9 Yr after Valproate-Induced Pancreatitis.” *Pediatric Diabetes* 13, no. 5 (2012): 444–47.

Jha, Shivkumar H., Jyothi A. Reddy, and Jatin K. Dave. “Dapsone-Induced Acute Pancreatitis.” *The Annals of Pharmacotherapy* 37, no. 10 (2003): 1438–40.

Jiang, Rong, Li Xu, Yun Huang, Chen Fang, Heming Guo, Sicheng Li, Jianwu Wu, and Ziyan Du. “Anti-PD-1 Drug (Nivolumab) May Induce Acute and Life-Threatening Pancreatitis in Lung Cancer Patient: A Case Report.” *Pancreas* 47, no. 8 (2018): e53–54. <https://doi.org/10.1097/MPA.0000000000001107>.

Johnson, Jeremy L., and Ilana B. Loomis. “A Case of Simvastatin-Associated Pancreatitis and Review of Statin-Associated Pancreatitis.” *Pharmacotherapy* 26, no. 3 (2006): 414–22.

Juang, Paul, Robert L. Page, and Ronald Zolty. “Probable Loop Diuretic-Induced Pancreatitis in a Sulfonamide-Allergic Patient.” *The Annals of Pharmacotherapy* 40, no. 1 (2006): 128–34.

Jubert, P., R. Fernandez, and A. Ruiz. “Clozapine-Related Pancreatitis.” *Annals of Internal Medicine* 121, no. 9 (1994): 722–23.

Julve Pardo, R., M. Garcia-Escrig, J. Catala Barcelo, J. H. del Val, and J. Fernandez Ponsati. “[Acute Pancreatitis as an Effect of IV Methylprednisolone in the Treatment of Optical Neuritis].” *Neurologia (Barcelona, Spain)* 13, no. 7 (1998): 372–73.

Jung, Jung Hwa, Jong Ryeal Hahm, Jaehoon Jung, Soo Kyoung Kim, Sungsu Kim, Kyong Young Kim, Bo Ra Kim, Hong Jun Kim, Yi Yeong Jeong, and Sun Joo Kim. “Acute Pancreatitis Induced by Methimazole Treatment in a 51-Year-Old Korean Man: A Case Report.” *Journal of Korean Medical Science* 29, no. 8 (2014): 1170–73.

Jung, Minyoung, Jihyun Kim, Ji Young Lee, Minji Kim, Seung-Hyun Kim, and Kangmo Ahn. “Trimethoprim-Sulfamethoxazole Induces Acute Pancreatitis Associated with Drug-Specific Cytotoxic T Lymphocytes.” *The Journal of Allergy and Clinical Immunology. In Practice* 7, no. 1 (January 2019): 336–38. <https://doi.org/10.1016/j.jaip.2018.06.009>.

Kanbay, M., M. Korkmaz, U. Yilmaz, G. Gur, and S. Boyacioglu. “Acute Pancreatitis Due to Ramipril Therapy.” *Postgraduate Medical Journal* 80, no. 948 (2004): 617–18.

Kanbay, M., H. Selcuk, U. Yilmaz, and S. Boyacioglu. “Recurrent Acute Pancreatitis Probably Secondary to Lisinopril.” *Southern Medical Journal* 99, no. 12 (2006): 1388–89.

Kataria, Pritam Suresh Chandra, Pradip Piraji Kendre, Apurva Ashok Patel, Murtaza Zoher Bohra, and Nahush Tahiliani. “Tamoxifen Induced Pancreatitis: An Unusual Complication of Commonly Used Drug.” *Journal of Clinical and Diagnostic Research: JCDR* 11, no. 8 (August 2017): XD05–6. <https://doi.org/10.7860/JCDR/2017/27440.10467>.

Kattah Martinez, Laura Ximena, Lisseth Fernanda Marín Carrillo, and Leonardo Rojas Melo. “Sorafenib-Induced Acute Pancreatitis in a Patient with Differentiated Thyroid Cancer.” *European Thyroid Journal* 7, no. 3 (June 2018): 145–48. <https://doi.org/10.1159/000488316>.

Kawabe, K., and S. I. Ueno. “A Case of Acute Pancreatitis Associated with Risperidone Treatment.” *Clinical Psychopharmacology and Neuroscience* 12, no. 1 (2014): 67–68.

Kawakami, Hiroshi, Yoshimasa Kubota, Tesshin Ban, Nobuhiro Shibata, and Ayumu Hosokawa. “Lenvatinib-Induced Acute Pancreatitis Associated With a Pancreatic Pseudocyst and Splenic Pseudoaneurysms.” *Pancreas* 47, no. 6 (2018): e34–35. <https://doi.org/10.1097/MPA.0000000000001061>.

Kayemba Kay’s Kabangu, S., M. Bovier Lapierre, and E. Jalaguier. “[Acute Pancreatitis and Valproic Acid].” *Pediatrie* 46, no. 12 (1991): 839–43.

Keefe, M., and F. Munro. “Acute Pancreatitis: A Fatal Complication of Treatment of Bullous Pemphigoid with Systemic Corticosteroids.” *Dermatologica* 179, no. 2 (1989): 73–75.

Khan, I. H., and N. Edward. “Pancreatitis Associated with Diclofenac.” *Postgraduate Medical Journal* 69, no. 812 (1993): 486–87.

Khanna, S., and A. Kumar. “Acute Pancreatitis Due to Hydrocortisone in a Patient with Ulcerative Colitis [2].” *Journal of Gastroenterology and Hepatology (Australia)* 18, no. 9 (2003): 1110–11.

Khetpal, Neelam, Lokesh Yadav, Sameen Khalid, and Ranjeet Kumar. “Eluxadoline-Induced Recurrent Pancreatitis in a Young Female without a Gallbladder: A Case Report and Literature Review.” *Cureus* 10, no. 12 (December 18, 2018): e3747. <https://doi.org/10.7759/cureus.3747>.

Kikuchi, Itsuka, Nobuyuki Miyata, Yukihiro Yoshimura, Kazunori Miyamoto, and Natsuo Tachikawa. “Methimazole-Induced Acute Pancreatitis: A Case Report.” *Clinical Journal of Gastroenterology* 12, no. 3 (June 2019): 239–42. <https://doi.org/10.1007/s12328-018-0926-5>.

Kim, Young Ae, Sol Lee, Ji Woong Jung, Yu Jin Kwon, Gyeong Bok Lee, Dong Gue Shin, Sang Su Park, Jin Yun, Yong Seog Jang, and Dong Hui Cho. “Severe Acute Pancreatitis Due to Tamoxifen-Induced Hypertriglyceridemia with Diabetes Mellitus.” *Chinese Journal of Cancer Research = Chung-Kuo Yen Cheng Yen Chiu* 26, no. 3 (2014): 341–44.

Kitamura, Yosuke, Hidehiko Yoshii, Koshiro Nishimoto, Yusuke Shinchi, Shigeki Tokonabe, Masaya Takao, and Yuichiro Daido. “A Case of Pancreatic Side Effects Resulting from Sorafenib and Axitinib Treatment of Stage IV Renal Cell Carcinoma.” *The Keio Journal of Medicine* 64, no. 4 (2015): 62–64.

Klein, S. M., and M. A. Khan. “Hepatitis, Toxic Epidermal Necrolysis and Pancreatitis in Association with Sulindac Therapy.” *The Journal of Rheumatology* 10, no. 3 (1983): 512–13.

Knackstedt, C., R. Winograd, A. Koch, F. Abuzahra, C. Trautwein, and H. E. Wasmuth. “Acute Necrotic Pancreatitis Induced by Severe Hypercalcaemia Due to Tacalcitol Ointment.” *The British Journal of Dermatology* 156, no. 3 (2007): 576–77.

Knezevich, Emily, Theresa Crnic, Scott Kershaw, and Andjela Drincic. “Liraglutide-Associated Acute Pancreatitis.” *American Journal of Health-System Pharmacy : AJHP : Official Journal of the American Society of Health-System Pharmacists* 69, no. 5 (2012): 386–89.

Kobayashi, Y., T. Kanemitu, A. Kamoto, M. Satoh, N. Mori, K. Sekii, T. Yoshioka, H. Itatani, and T. Fujimoto. “Painless Acute Pancreatitis Associated with Sorafenib Treatment: A Case Report.” *Medical Oncology* 28, no. 2 (2011): 463–65.

Kocak, Mehmet Zahid, Gulali Aktas, Edip Erkus, Tuba T. Duman, Burcin M. Atak, Deniz Sahin, and Haluk Savli. “A Case of Sitagliptin-Induced Mild Acute Pancreatitis.” *Journal of the College of Physicians and Surgeons--Pakistan: JCPSP* 28, no. 4 (April 2018): 334. <https://doi.org/10.29271/jcpsp.2018.04.334>.

Koniver, G. A., and J. E. Scott. “Pancreatitis with Pseudocyst: A Complication of L-Asparaqinase Therapy for Leukemia.” *Delaware Medical Journal* 50, no. 6 (1978): 330–32.

Koufakis, T., I. Gabranis, K. Ntais, D. Karangelis, S. Batalla, N. Paschala, A. Margaritis, K. Makrigiannis, K. Karamitsos, and K. Karanikas. “Acute Pancreatitis Due to Clarithromycin Therapy: A Rare Adverse Effect of a Common Drug.” *European Journal of Internal Medicine* 24 (2013): e77.

Kovacic, Sanja, Sinisa Roginic, Johann Nemrava, Ksenija Gospocic, Maida Seferovic Saric, and Kresimir Luetic. “Acute Pancreatitis in Two Patients with Parkinson’s Disease.” Edited by Udo Schumacher. *Cogent Medicine* 4, no. 1 (March 31, 2017). <https://doi.org/10.1080/2331205X.2017.1312802>.

Kumar, A. N., D. E. Schwartz, and K. G. Lim. “Propofol-Induced Pancreatitis: Recurrence of Pancreatitis after Rechallenge.” *Chest* 115, no. 4 (1999): 1198–99.

Kumar, D. M., S. Sundar, and S. Vasanthan. “A Case of Paclitaxel-Induced Pancreatitis [1].” *Clinical Oncology* 15, no. 1 (2003): 35.

Kumar, S., V. J. Schnadig, and M. G. MacGregor. “Fatal Acute Pancreatitis Associated with Pentamidine Therapy.” *The American Journal of Gastroenterology* 84, no. 4 (1989): 451–53.

Kuyucu, N., C. Kara, A. Bakirtac, and T. Tezic. “Successful Treatment of Visceral Leishmaniasis with Allopurinol plus Ketoconazole in an Infant Who Developed Pancreatitis Caused by Meglumine Antimoniate.” *The Pediatric Infectious Disease Journal* 20, no. 4 (2001): 455–57.

Laczek, J. T., M. Shrestha, N. D. Kortan, and J. M. Lake. “Carbamazepine-Induced Pancreatitis with Positive Rechallenge.” *Journal of Clinical Gastroenterology* 44, no. 2 (2010): 153–54.

Lai, S. W., Y. C. Wang, C. H. Wang, and T. Y. Huang. “Acute Pancreatitis and Erythema Nodosum Associated with Azathioprine.” *QJM : Monthly Journal of the Association of Physicians* 105, no. 4 (2012): 363–64.

Lambrianides, A. L., and R. D. Rosin. “Acute Pancreatitis Complicating Excessive Intake of Phenolphthalein.” *Postgraduate Medical Journal* 60, no. 705 (1984): 491–92.

Lankisch, P. G., and H. M. Werner. “Mirtazapine: Another Drug Responsible for Drug-Induced Acute Pancreatitis? A Letter of Warning.” *Pancreas* 26, no. 2 (2003): 211.

Laugel, V., B. Escande, N. Entz-Werle, F. Mazingue, A. Ferster, Y. Bertrand, F. Missud, and P. Lutz. “[Severe Acute Pancreatitis in Children Receiving Asparaginase: Multicenter Retrospective Study].” *Archives de Pediatrie : Organe Officiel de La Societe Francaise de Pediatrie* 12, no. 1 (2005): 34–41.

Leblanc, A., B. Leclercq, G. Nitenberg, P. Lasser, D. Couanet, O. Hartmann, and J. Lemerle. “[Acute hemorrhagic pancreatitis caused by asparaginase. A case in a child with a favorable course].” *Presse Medicale (Paris, France: 1983)* 12, no. 21 (May 14, 1983): 1351–53.

Ledder, Oren D., Daniel A. Lemberg, Chee Y. Ooi, and Andrew S. Day. “Are Thiopurines Always Contraindicated after Thiopurine-Induced Pancreatitis in Inflammatory Bowel Disease?” *Journal of Pediatric Gastroenterology and Nutrition* 57, no. 5 (2013): 583–86.

Lee, Chien Feng, Meng Shun Sun, and Yen Kuang Tai. “Saxagliptin-Induced Recurrent Acute Pancreatitis.” *Internal Medicine (Tokyo, Japan)* 53, no. 12 (2014): 1351–54.

Lee, Hwee Min, Antoine F. Villa, Sophie Caudrelier, and Robert Garnier. “Can Loperamide Cause Acute Pancreatitis?” *Pancreas* 40, no. 5 (2011): 780–81.

Lee, W. C., M. J. Wu, C. H. Cheng, C. H. Chen, M. C. Wen, H. C. Chen, and K. H. Shu. “Acute Pancreatitis Following Antilymphocyte Globulin Therapy in a Renal Transplant Recipient.” *Clinical Nephrology* 65, no. 2 (2006): 144–46.

Lee, Yang Deok, and Soo Teik Lee. “Acute Pancreatitis and Acute Renal Failure Complicating Doxylamine Succinate Intoxication.” *Veterinary and Human Toxicology* 44, no. 3 (2002): 165–66.

Leisure, G. S., J. O’Flaherty, L. Green, and D. R. Jones. “Propofol and Postoperative Pancreatitis.” *Anesthesiology* 84, no. 1 (1996): 224–27.

Lerche, A., M. Vyberg, and E. Kirkegaard. “Acute Cholangitis and Pancreatitis Associated with Sulindac (Clinoril).” *Histopathology* 11, no. 6 (1987): 647–53.

Levin, T. L., W. E. Berdon, H. B. Tang, and J. O. Haller. “Dideoxyinosine-Induced Pancreatitis in Human Immunodeficiency Virus-Infected Children.” *Pediatric Radiology* 27, no. 2 (1997): 189–91.

Levine, R. A., and R. F. McGuire. “Corticosteroid-Induced Pancreatitis: A Case Report Demonstrating Recurrence with Rechallenge.” *The American Journal of Gastroenterology* 83, no. 10 (1988): 1161–64.

Li, Mingqing, and Sandy Srinivas. “Acute Pancreatitis Associated with Sorafenib.” *Southern Medical Journal* 100, no. 9 (2007): 909–11.

Lilly, E. L. “Pancreatitis after Administration of Sulindac.” *JAMA* 246, no. 23 (1981): 2680.

Lin, Jinwen, Rending Wang, and Jianghua Chen. “Tigecycline-Induced Acute Pancreatitis in a Renal Transplant Patient: A Case Report and Literature Review.” *BMC Infectious Diseases* 18, no. 1 (02 2018): 201. <https://doi.org/10.1186/s12879-018-3103-z>.

Lin, Y. H., C. L. Perng, H. J. Lin, and F. Y. Chang. “Acute Pancreatitis Possibly Related to Finasteride.” *Journal of Clinical Gastroenterology* 32, no. 3 (2001): 276.

Liou, L. S., Y. J. Hung, C. H. Hsieh, and F. C. Hsiao. “Aggravation of Hypertriglyceridemia and Acute Pancreatitis in a Bipolar Patient Treated with Quetiapine.” *Yonsei Medical Journal* 55, no. 3 (2014): 831–33.

Lipshitz, Jay, Jonathan Kruh, Philip Cheung, and Manouchkathe Cassagnol. “Tigecycline-Induced Pancreatitis.” *Journal of Clinical Gastroenterology* 43, no. 1 (2009): 93.

Liu, Po Heng, Bor Jen Lee, Chen Yu Wang, and Dong Zong Hung. “Acute Pancreatitis after Severe Theophylline Overdose.” *Clinical Toxicology (Philadelphia, Pa.)* 46, no. 10 (2008): 1103.

Liviu, L., L. Yair, and S. Yehuda. “Pancreatitis Induced by Clarithromycin.” *Annals of Internal Medicine* 125, no. 8 (1996): 701.

Lons, T., and M. Chousterman. “[Simvastatin: A New Drug Responsible for Acute Pancreatitis?].” *Gastroenterologie Clinique et Biologique* 15, no. 1 (1991): 93–94.

Lopes Mondejar, P., M. P. Soto, F. Vences, and A. M. Hidalgo. “Acute Pancreatitis after Pregabalin Administration.” *Endocrinologia y Nutricion* 54, no. 6 (2007): 340.

Lopez Centeno, Beatriz, Monserrat Perez Encinas, and Sira Sanz Marquez. “ACUTE SECONDARY PANCREATITIS TO AZATHIOPRINE IN A PATIENT WITH SYSTEMIC LUPUS ERYTHEMATOSUS” 15, no. 5 (2013): 3.

Lott, J. A., L. W. Bond, R. C. Bobo, H. J. McClung, and R. D. Murray. “Valproic Acid-Associated Pancreatitis: Report of Three Cases and a Brief Review.” *Clinical Chemistry* 36, no. 2 (1990): 395–97.

Loulergue, P., and O. Mir. “Metronidazole-Induced Pancreatitis during HIV Infection.” *AIDS (London, England)* 22, no. 4 (2008): 545.

Lu, C. P., H. P. Wu, L. M. Chuang, B. J. Lin, C. Y. Chuang, and T. Y. Tai. “Pentamidine-Induced Hyperglycemia and Ketosis in Acquired Immunodeficiency Syndrome.” *Pancreas* 11, no. 3 (October 1995): 315–16.

Luo, H., H. Bhatt, S. Mohamad, E. Uhrik, S. Sen, T. Mathew, and A. Yousif. “Acute Pancreatitis: Possible Association of Dimethyl Fumarate for the Treatment of Relapsing-Remitting Multiple Sclerosis.” *Journal of Neurology* 262, no. 3 (2015): 779–80.

Madsen, J. S., and I. A. Jacobsen. “Angiotensin Converting Enzyme Inhibitor Therapy and Acute Pancreatitis.” *Blood Pressure* 4, no. 6 (1995): 369–71.

Magill, P., P. F. Ridgway, K. C. Conlon, and P. Neary. “A Case of Probable Ibuprofen-Induced Acute Pancreatitis.” *Journal of the Pancreas* 7, no. 3 (2006): 311–14.

Mahdi, Asmaa S., Mariya Molai, Juhi Chandwani, Huda Al Khalili, Hashim Ibrahim, Nenad Pandak, Faryal Khamis, and Eskild Petersen. “Late Onset Acute Pancreatitis in P. Falciparum Malaria - An Adverse Reaction to Intravenous Artesunate?” *IDCases* 12 (2018): 124–26. <https://doi.org/10.1016/j.idcr.2018.04.010>.

Mahto, Subodh Kumar, Pulin Kumar Gupta, Rajesh Satyapal Taneja, and Akanksha Singh. “Zidovudine-Induced Lactic Acidosis with Acute Pancreatitis and Myopathy: Lethal and Rare Complications.” *Indian Journal of Pharmacology* 50, no. 4 (August 2018): 212–14. <https://doi.org/10.4103/ijp.IJP_285_18>.

Maliekal, J., and C. F. Drake. “Acute Pancreatitis Associated with the Use of Lisinopril.” *The Annals of Pharmacotherapy* 27, no. 12 (1993): 1465–66.

Malozowski, S., W. Hung, D. C. Scott, and B. V. Stadel. “Acute Pancreatitis Associated with Growth Hormone Therapy for Short Stature [6].” *New England Journal of Medicine* 332, no. 6 (1995): 401–2.

Mann, S., and A. Thillainayagam. “Is Ciprofloxacin a New Cause of Acute Pancreatitis? [1].” *Journal of Clinical Gastroenterology* 31, no. 4 (2000): 336.

Maranan, M. C., S. I. Gerber, and G. G. Miller. “Gallstone Pancreatitis Caused by Ceftriaxone.” *The Pediatric Infectious Disease Journal* 17, no. 7 (1998): 662–63.

Marazuela, Monica, Graciano Sanchez de Paco, Isabel Jimenez, Rafael Carraro, Jesus Fernandez-Herrera, Jose Maria Pajares, and Antonio Gomez-Pan. “Acute Pancreatitis, Hepatic Cholestasis, and Erythema Nodosum Induced by Carbimazole Treatment for Graves’ Disease.” *Endocrine Journal* 49, no. 3 (2002): 315–18.

Mari, B., E. Brullet, R. Campo, E. Bustamante, and J. Bombardo. “Acute Pancreatitis by 5-Aminosalicillic Acid [2].” *Gastroenterologia y Hepatologia* 22, no. 1 (1999): 28–29.

Marinella, M. A., and J. E. Billi. “Lisinopril Therapy Associated with Acute Pancreatitis.” *The Western Journal of Medicine* 163, no. 1 (1995): 77–78.

Maringhini, A., A. Termini, R. Patti, M. Ciambra, P. Biffarella, and L. Pagliaro. “Enalapril-Associated Acute Pancreatitis: Recurrence after Rechallenge.” *The American Journal of Gastroenterology* 92, no. 1 (1997): 166–67.

Markov, M., K. Patel, A. Raeesy, A. Bant, D. H. Van Thiel, and A. Nadir. “Liver and Pancreatic Injury Induced by Antituberculous Therapy.” *Digestive Diseases and Sciences* 52, no. 11 (2007): 3275–81.

Marot, J. C., S. Jonckheere, H. Munyentwali, L. Belkhir, B. Vandercam, and J. C. Yombi. “Tigecycline-Induced Acute Pancreatitis: About Two Cases and Review of the Literature.” *Acta Clinica Belgica* 67, no. 3 (2012): 229–32.

Maroy, B. “[Benign Acute Pancreatitis Probably Due to Taking Ketoprofen].” *Therapie* 53, no. 6 (1998): 602–3.

Martin, A. “Acute Pancreatitis Associated with Clozapine Use [12].” *American Journal of Psychiatry* 149, no. 5 (1992): 714.

Martin, T., A. Taupignon, E. Graf, and D. Perrin. “[Pancreatitis and Hepatitis in a Patient Treated with Enalapril Maleate. A Case Report].” *Therapie* 44, no. 6 (1989): 449–50.

Martinez-Granados, Francisco, Jose Noe Navarro, Jose Luis Estrada, Maria Teresa Martinez-Lazcano, Felix Lluis-Casajuana, and Juan Pablo Ordovas-Baines. “Ertapenem-Induced Acute Pancreatitis in a Surgical Elderly Patient.” *Pharmacy World & Science : PWS* 30, no. 3 (2008): 278–80.

Maxson, C. J., S. M. Greenfield, and J. L. Turner. “Acute Pancreatitis as a Common Complication of 2’,3’-Dideoxyinosine Therapy in the Acquired Immunodeficiency Syndrome.” *The American Journal of Gastroenterology* 87, no. 6 (1992): 708–13.

McBride, C. E., R. T. Yavorski, F. M. Moses, M. E. Robson, Jr. Solimando, and J. C. Byrd. “Acute Pancreatitis Associated with Continuous Infusion Cytarabine Therapy.” *Cancer* 77, no. 12 (1996): 2588–91.

McBride, M. O., M. Linney, R. N. Davidson, and J. N. Weber. “Pancreatic Necrosis Following Treatment of Leishmaniasis with Sodium Stibogluconate.” *Clinical Infectious Diseases : An Official Publication of the Infectious Diseases Society of America* 21, no. 3 (1995): 710.

McCarter, T. L., and Y. K. Chen. “Marked Hyperlipidemia and Pancreatitis Associated with Isotretinoin Therapy.” *The American Journal of Gastroenterology* 87, no. 12 (1992): 1855–58.

McCarthy, A. E., J. S. Keystone, and K. C. Kain. “Pancreatitis Occurring during Therapy with Stibogluconate: Two Case Reports [23].” *Clinical Infectious Diseases* 17, no. 5 (1993): 952–53.

McCormick, P. A., D. O’Donoghue, and N. Brennan. “Diphenoxylate and Pancreatitis.” *Lancet (London, England)* 1, no. 8431 (1985): 752.

McFadden, M., A. Gordon, G. Leong, D. Ward, and J. G. Scott. “Pancreatitis Associated with Metformin Used for Management of Clozapine-Related Weight Gain.” *Australian and New Zealand Journal of Psychiatry* 50, no. 7 (2016): 701–2.

McLean, R., S. Martin, and P. R. Lam-Po-Tang. “Fatal Case of L-Asparaginase Induced Pancreatitis.” *Lancet (London, England)* 2, no. 8312 (1982): 1401–2.

McMahon, M. A., G. Kearns, J. McCaffrey, and L. Grogan. “Association between Paclitaxel and Necrotic Pancreatitis.” *Irish Medical Journal* 99, no. 9 (2006): no.

Meczker, Ágnes, Alexandra Mikó, and Péter Hegyi. “5-ASA Induces Mild Acute Pancreatitis. Case Report and Review of the Literature.” *Journal of Gastrointestinal and Liver Diseases: JGLD* 27, no. 2 (2018): 189–94. <https://doi.org/10.15403/jgld.2014.1121.272.asa>.

Memis, Dilek, Esin Akalin, and Tuba Yucel. “Indomethacin-Induced Pancreatitis: A Case Report.” *JOP : Journal of the Pancreas* 6, no. 4 (2005): 344–47.

Memon, A. N. “Pancreatitis and Sulindac.” *Annals of Internal Medicine* 97, no. 1 (1982): 139.

Mendoza, J. L., J. R. Larrubia, R. Lana, D. Espinos, and M. Diaz-Rubio. “[Acute Pancreatitis Induced by Isoniazid, a Casual Association].” *Anales de Medicina Interna (Madrid, Spain : 1984)* 15, no. 11 (1998): 588–90.

Mennecier, Didier, Franck Ceppa, Leila Sinayoko, Damien Corberand, Florence Harnois, Catherine Thiolet, and Olivier Farret. “[Acute Pancreatitis after Treatment by Celecoxib].” *Gastroenterologie Clinique et Biologique* 31, no. 8-9 Pt 1 (2007): 668–69.

Midgard, Rune, Kare Ertresvag, Erik Trondsen, and Olav Spigset. “Life-Threatening Acute Pancreatitis Associated with Interferon Beta-1a Treatment in Multiple Sclerosis.” *Neurology* 65, no. 1 (2005): 170–71.

Miller, L. G., and G. Tan. “Drug-Induced Pancreatitis (Lisinopril).” *The Journal of the American Board of Family Practice* 12, no. 2 (1999): 150–53.

Mirete, G., M. Masia, F. Gutierrez, A. Mora, C. Escolano, and A. Maestre. “Acute Pancreatitis as a Complication of Ritonavir Therapy in a Patient with AIDS.” *European Journal of Clinical Microbiology and Infectious Diseases* 17, no. 11 (1998): 810–11.

Mofenson, H. C., T. R. Caraccio, H. Nawaz, and G. Steckler. “Acetaminophen Induced Pancreatitis.” *Journal of Toxicology.Clinical Toxicology* 29, no. 2 (1991): 223–30.

Molina Infante, J., A. B. Prieto Bermejo, Gallardo B. Perez, and Bermejo M. Fernandez. “Toxic Metformin-Associated Acute Pancreatitis without Kidney Failure.” *Medicina Clinica* 131, no. 13 (2008): 519.

Moreiras Plaza, M., G. Rodriguez Goyanes, L. Cuina, and R. Alonso. “On the Toxicity of Valproic-Acid.” *Clinical Nephrology* 51, no. 3 (1999): 187–89.

Moreno Escobosa, M. C., J. Amat Lopez, S. Cruz Granados, and M. C. Moya Quesada. “Pancreatitis Due to Codeine.” *Allergologia et Immunopathologia* 33, no. 3 (2005): 175–77.

Moreno Sanchez-Canete, A., J. I. Bernardino de la Serna, J. Garcia Puig, and A. Gil Aguado. “[Acute Pancreatitis Associated with ACE Inhibitors].” *Atencion Primaria* 22, no. 4 (1998): 260–61.

Mori, Shunsuke, and Kenji Ebihara. “A Sudden Onset of Diabetic Ketoacidosis and Acute Pancreatitis after Introduction of Mizoribine Therapy in a Patient with Rheumatoid Arthritis.” *Modern Rheumatology* 18, no. 6 (2008): 634–38.

Morse, D., N. Kumar, and G. Aisenberg. “Gadolinium-Induced Acute Pancreatitis.” *Consultant* 58, no. 9 (2018): 257–58.

Moslim, Maitham A., Thomas C. Sodeman, and Ali T. Nawras. “A Case of Suggested Ibuprofen-Induced Acute Pancreatitis.” *American Journal of Therapeutics* 23, no. 6 (2016): e1918–21.

Mouallem, M., T. Sirotin, and Z. Farfel. “Nitrofurantoin-Induced Pancreatitis.” *Israel Medical Association Journal* 5, no. 10 (2003): 754–55.

Moy, Brian T., and Nikhil Kapila. “Probable Doxycycline-Induced Acute Pancreatitis.” *American Journal of Health-System Pharmacy : AJHP : Official Journal of the American Society of Health-System Pharmacists* 73, no. 5 (2016): 286–91.

Muchnick, J. S., and J. L. Mehta. “Angiotensin-Converting Enzyme Inhibitor-Induced Pancreatitis.” *Clinical Cardiology* 22, no. 1 (1999): 50–51.

Muluneh, Benyam, Larry W. Buie, and Frances Collichio. “Vemurafenib-Associated Pancreatitis: Case Report.” *Pharmacotherapy* 33, no. 4 (2013): e43–44.

Muniraj, Thiruvengadam, and Harry R. Aslanian. “Hypertriglyceridemia Independent Propofol-Induced Pancreatitis.” *JOP : Journal of the Pancreas* 13, no. 4 (2012): 451–53.

Murakawa, M., T. Okamura, T. Shibuya, M. Harada, T. Otsuka, and Y. Niho. “Use of a Synthetic Protease Inhibitor for the Treatment of L-Asparaginase-Induced Acute Pancreatitis Complicated by Disseminated Intravascular Coagulation.” *Annals of Hematology* 64, no. 5 (1992): 249–52.

Murphey, S. A., and A. S. Josephs. “Acute Pancreatitis Associated with Pentamidine Therapy.” *Archives of Internal Medicine* 141, no. 1 (1981): 56–58.

Murphy, M. J., I. W. Lyon, J. W. Taylor, and G. Mitts. “Valproic Acid Associated Pancreatitis in an Adult.” *Lancet (London, England)* 1, no. 8210 (1981): 41–42.

Murtaza, Ghulam, Anadil Faqah, Nicholas Konowitz, Hannah Lu, Aneesh Kuruvilla, and Sujeen Adhikari. “Acute Pancreatitis Related to a Chemotherapy Drug.” *World Journal of Oncology* 8, no. 1 (February 2017): 18–19. <https://doi.org/10.14740/wjon1006e>.

Muzaffar, Mahvish, Jingquan Jia, Darla Liles, Musharraf Naveed, and Anita Kumari. “Acute Pancreatitis Associated With Ado-Trastuzumab Emtansine.” *American Journal of Therapeutics* 23, no. 2 (2016): e572–74.

Nadir, A., F. Nadir, T. Hassanein, A. Gurakar, H. I. Wright, and D. H. Van Thiel. “Acute Relapsing Pancreatitis Induced with Ursodeoxycholic Acid Therapy.” *The Journal of the Oklahoma State Medical Association* 88, no. 7 (July 1995): 295–98.

Nakagawa, Nozomu, Nobuaki Ochi, Hiromichi Yamane, Yoshihiro Honda, Yasunari Nagasaki, Noriyo Urata, Hidekazu Nakanishi, Hirofumi Kawamoto, and Nagio Takigawa. “Ceftriaxone-Associated Pancreatitis Captured on Serial Computed Tomography Scans.” *Radiology Case Reports* 13, no. 1 (February 2018): 43–46. <https://doi.org/10.1016/j.radcr.2017.10.022>.

Nakata, Hirosuke, Seita Sugitani, Shuhei Yamaji, Satoko Otsu, Yoshihito Higashi, Yumiko Ohtomo, and Gen Inoue. “Pancreatitis with Pancreatic Tail Swelling Associated with Incretin-Based Therapies Detected Radiologically in Two Cases of Diabetic Patients with End-Stage Renal Disease.” *Internal Medicine (Tokyo, Japan)* 51, no. 21 (2012): 3045–49.

Nango, Daisuke, Hiroki Nakashima, Yukifumi Hirose, Masaaki Shiina, and Hirotoshi Echizen. “Causal Relationship between Acute Pancreatitis and Methylprednisolone Pulse Therapy for Fulminant Autoimmune Hepatitis: A Case Report and Review of Literature.” *Journal of Pharmaceutical Health Care and Sciences* 4 (2018): 14. <https://doi.org/10.1186/s40780-018-0111-5>.

Navarro-Mingorance, A., A. J. Castellanos-Alcarria, S. Ibanez-Mico, A. Cervantes-Pardo, and P. Sanchez-Pedreno. “Dapsone-Induced Isolated Acute Pancreatitis in a Child with Linear IgA Dermatitis.” *Indian Journal of Pediatrics* 81, no. 7 (2014): 735–36.

Nelis, G. F. “Nitrofurantoin-Induced Pancreatitis: Report of a Case.” *Gastroenterology* 84, no. 5 Pt 1 (1983): 1032–34.

Ng, J. Y., A. P. Disney, T. E. Jones, and G. Purdie. “Acute Pancreatitis and Sodium Valproate.” *The Medical Journal of Australia* 2, no. 8 (1982): 362.

Nicolau, D. P., D. E. Mengedoht, and J. J. Kline. “Tetracycline-Induced Pancreatitis.” *The American Journal of Gastroenterology* 86, no. 11 (1991): 1669–71.

Nieto, Y., P. Russ, G. Everson, S. I. Bearman, P. J. Cagnoni, R. B. Jones, and E. J. Shpall. “Acute Pancreatitis during Immunosuppression with Tacrolimus Following an Allogeneic Umbilical Cord Blood Transplantation.” *Bone Marrow Transplantation* 26, no. 1 (2000): 109–11.

Nigwekar, Sagar U., and Kevin J. Casey. “Metronidazole-Induced Pancreatitis. A Case Report and Review of Literature.” *JOP : Journal of the Pancreas* 5, no. 6 (2004): 516–19.

Nind, G., and W. Selby. “Acute Pancreatitis: A Rare Complication of Celecoxib [3].” *Internal Medicine Journal* 32, no. 12 (2002): 624–25.

Nishawala, M. A., M. Callaghan, J. J. Malatack, B. Moughan, P. J. Ambrosini, B. Price, and J. Elia. “Pancreatitis Associated with Serotonin-Dopamine Antagonists.” *Journal of Child and Adolescent Psychopharmacology* 7, no. 3 (1997): 211–13.

Nishioka, S. de A., and L. Q. Guedes. “Possible Lovastatin-Induced Fatal Necrotizing Pancreatitis.” *Journal of Pharmacy Technology* 19, no. 5 (2003): 283–86.

Nogueira Soriano, J. M., Diaz G. Pelaez, Montesinos A. Abad, and Carretero J. Esteban. “Acute Pancreatitis Produced by 6-Mercaptopurine in a Patient with Crohn Disease.” *Gastroenterologia y Hepatologia* 14, no. 1 (1991): 44–45.

Nott, D. M., and B. A. de Sousa. “Suspected Cimetidine-Induced Acute Pancreatitis.” *The British Journal of Clinical Practice* 43, no. 7 (1989): 264–65.

Nwogbe, Benedict, Julia Ferie, Hannah Smith, Indunil Gunawardena, and Ketan Dhatariya. “Significant Lamotrigine Overdose Associated with Acute Pancreatitis.” *Journal of the Royal Society of Medicine* 102, no. 3 (2009): 118–19.

Nykamp, Diane, and Emily J. Kraus. “Antacid-Induced Acute Pancreatitis.” *The Consultant Pharmacist : The Journal of the American Society of Consultant Pharmacists* 28, no. 4 (2013): 247–51.

Oflazoglu, U., U. Varol, A. Alacacioglu, T. Salman, N. Demir, H. S. Semiz, A. Karaoglu, and I. Oztop. “Case Report of a Renal Cell Carcinoma Patient with Acute Pancreatitis under Both Sunitinib and Axitinib Treatment.” *Journal of Oncological Science* 2, no. 2–3 (2016): 63–65.

Olson, E. L., and Y. E. Whang. “Hypertriglyceridemia and Pancreatitis Associated with Estramustine Phosphate.” *American Journal of Clinical Oncology: Cancer Clinical Trials* 25, no. 4 (2002): 342–43.

O’Neil, M. G., S. E. Selub, and L. J. Hak. “Pancreatitis during Pentamidine Therapy in Patients with AIDS.” *Clinical Pharmacy* 10, no. 1 (1991): 56–59.

Otusbo, S., T. Huruzono, H. Kobae, S. Yoshimi, and K. Miyata. “Pancreatitis with Normal Serum Amylase Associated with Sodium Valproate: A Case Report.” *Brain & Development* 17, no. 3 (1995): 219–21.

Ozaydin, Eda, Handan Yukselgungor, and Gulsen Kose. “Acute Hemorrhagic Pancreatitis Due to the Use of Valproic Acid in a Child.” *European Journal of Paediatric Neurology : EJPN : Official Journal of the European Paediatric Neurology Society* 12, no. 2 (2008): 141–43.

Ozdogan, O., V. Tahan, A. Cincin, N. Imeryuz, and N. Tozun. “Acute Pancreatitis Associated with the Use of Peginterferon [5].” *Pancreas* 34, no. 4 (2007): 485–87.

Pais, J. R., C. Cazorla, E. Novo, and A. Viana. “Massive Haemorrhage from Rupture of a Pancreatic Pseudocyst after Pentamidine-Associated Pancreatitis.” *The European Journal of Medicine* 1, no. 4 (1992): 251–53.

Paloyan, D., B. Levin, and D. Simonowitz. “Azathioprine-Associated Acute Pancreatitis.” *The American Journal of Digestive Diseases* 22, no. 9 (1977): 839–40.

Pandey, K., D. Singh, C. S. Lal, V. N. R. Das, and P. Das. “Fatal Acute Pancreatitis in a Patient with Visceral Leishmaniasis during Miltefosine Treatment.” *Journal of Postgraduate Medicine* 59, no. 4 (2013): 306–8.

Park, Tae Young, Hyoung Chul Oh, and Jae Hyuk Do. “A Case of Recurrent Pancreatitis Induced by Trimethoprim-Sulfamethoxazole Re-Exposure.” *Gut and Liver* 4, no. 2 (2010): 250–52.

Parker, P. H., G. L. Helinek, F. K. Ghishan, and H. L. Greene. “Recurrent Pancreatitis Induced by Valproic Acid. A Case Report and Review of the Literature.” *Gastroenterology* 80, no. 4 (1981): 826–28.

Pascual Velasco, F., L. Goicoechea Ibarra, and G. Bichara Antanios. “[Acute Pancreatitis Induced by Erythromycin: A New Case].” *Medicina Clinica* 97, no. 12 (1991): 473–74.

Passier, J. L. M., E. P. van Puijenbroek, G. J. P. M. Jonkers, and A. C. van Grootheest. “Pancreatitis Associated with the Use of Itraconazole.” *The Netherlands Journal of Medicine* 68, no. 6 (2010): 285–89.

Patel, Kinner M., Erik Pikas, and Tanya George. “Drug-Induced Necrotizing Pancreatitis With a Focus on Canagliflozin.” *American Journal of Therapeutics* 24, no. 4 (August 2017): e496. <https://doi.org/10.1097/MJT.0000000000000561>.

Patrick, Kaitlin A., Jenna T. Jarriel, and Michelle A. Hieger. “Pancreatic Pseudocyst Due to Acute Valproic Acid Overdose.” *American Journal of Therapeutics* 25, no. 5 (October 2018): e584–85. <https://doi.org/10.1097/MJT.0000000000000684>.

Paul, A. C., S. P. Oommen, S. Angami, and P. D. Moses. “Acute Pancreatitis in a Child with Idiopathic Ulcerative Colitis on Long-Term 5-Aminosalicylic Acid Therapy.” *Indian Journal of Gastroenterology : Official Journal of the Indian Society of Gastroenterology* 19, no. 4 (2000): 195–96.

Pauwels, A., M. Eliaszewicz, D. Larrey, F. Lacassin, J. M. Poirier, M. C. Meyohas, and J. Frottier. “Pentamidine-Induced Acute Pancreatitis in a Patient with AIDS.” *Journal of Clinical Gastroenterology* 12, no. 4 (1990): 457–59.

Pecquenard, L., M. Damay, C. Naveau, V. Lemarchand, E. Dufay, and T. Berod. “[Delayed Acute Pancreatitis after Treatment with L-Asparaginase. A Case Report].” *Therapie* 45, no. 5 (1990): 453–54.

Pedregal, M., J. L. Larraona, A. Tristancho, T. Lopez, and A. Maraver. “Acute Pancreatitis during Naltrexone Treatment [1].” *Gastroenterologia y Hepatologia* 15, no. 7 (1992): 433–34.

Pedrol, E., J. A. Martos, V. Plaza, R. Celis, and J. M. Montserrat. “[Acute Pancreatitis Caused by Demeclocycline].” *Revista Clinica Espanola* 184, no. 7 (1989): 392–93.

Peron, Julien, Safia Khenifer, Valerie Potier, Thierry Vitry, Florian Pasquet, Robin Rassat, and Michel Pavic. “Axitinib-Induced Acute Pancreatitis: A Case Report.” *Anti-Cancer Drugs* 25, no. 4 (2014): 478–79.

Pezzilli, R., R. Ceciliato, R. Corinaldesi, and B. Barakat. “Acute Pancreatitis Due to Simvastatin Therapy: Increased Severity after Rechallenge.” *Digestive and Liver Disease* 36, no. 9 (2004): 639–40.

Philip, A., P. Sivaprakasam, T. G. Sagar, and P. Ganesan. “Voriconazole-Induced Pancreatitis in a Patient of Acute Myeloid Leukemia and Invasive Aspergillosis.” *Journal of Pediatric Hematology/Oncology* 34, no. 5 (2012): 406.

Picardo, Sherman, Kenji So, Kannan Venugopal, and Marcus Chin. “Vedolizumab-Induced Acute Pancreatitis: The First Reported Clinical Case.” *BMJ Case Reports* 2018 (January 5, 2018). <https://doi.org/10.1136/bcr-2017-222554>.

Plotnick, B. H., I. Cohen, T. Tsang, and T. Cullinane. “Metronidazole-Induced Pancreatitis.” *Annals of Internal Medicine* 103, no. 6 I (1985): 891–92.

Poldermans, D., and M. van Blankenstein. “Pancreatitis Induced by Disodium Azodisalicylate.” *The American Journal of Gastroenterology* 83, no. 5 (1988): 578–80.

Prajapati, S., S. Shah, C. Desai, M. Desai, and R. K. Dikshit. “Atorvastatin-Induced Pancreatitis.” *Indian Journal of Pharmacology* 42, no. 5 (2010): 324–25.

Prigigine, Th, B. Futeral, and M. Kraytman. “Acute Hemorrhagic Pancreatitis Associated with Chlorthalidone Therapy.” *Acta Clinica Belgica* 33, no. 4 (1978): 272.

Priya, G., H. Bhagat, M. P. Pandia, A. Chaturvedi, A. Seth, and R. Goswami. “Can Propofol Precipitate Pancreatitis in Patients with Cushing’s Syndrome?” *Acta Anaesthesiologica Scandinavica* 49, no. 9 (2005): 1381–83.

Prot-Labarthe, S., R. Youdaren, M. Benkerrou, R. Basmaci, and M. Lorrot. “Pediatric Acute Pancreatitis Related to Tigecycline.” *Pediatric Infectious Disease Journal* 29, no. 9 (2010): 890–91.

Quan, Wei, Qing Shao, Hui Zhang, Fei-Hu Liu, and Xiao-Hong Zhang. “Acute Pancreatitis Associated with Valproate Treatment.” *Chinese Medical Journal* 131, no. 15 (August 5, 2018): 1889–90. <https://doi.org/10.4103/0366-6999.237390>.

Rabassa, A. A., G. Trey, U. Shukla, T. Samo, and B. S. Anand. “Isoniazid-Induced Acute Pancreatitis.” *Annals of Internal Medicine* 121, no. 6 (1994): 433–34.

Radke, M., G. Bartolomaeus, M. Muller, and I. Richter. “Acute Pancreatitis in Crohn’s Disease Due to 5-ASA Therapy.” *Journal of Pediatric Gastroenterology and Nutrition* 16, no. 3 (1993): 337–39.

Raiss, Hanan, Lamiae El Amarti, Jean Dominique Tigaud, Mohamed Layachi, Amandine Bruyas, Saber Boutayeb, and Hassan Errihani. “Probable Paclitaxel-Induced Pancreatitis: Uncommon Case Report and Literature Review.” *Journal of Gastrointestinal Oncology* 8, no. 6 (December 2017): E80–83. <https://doi.org/10.21037/jgo.2017.08.06>.

Raja, M., and A. Azzoni. “A Case of Clozapine-Associated Pancreatitis.” *Open Neuropsychopharmacology Journal* 4, no. 1 (2011): 5–7.

Ramdani, M., A. M. Schmitt, J. Liautard, O. Duhamel, P. Legroux, J. Gislon, E. A. Pariente, D. Agay, and D. Faure. “[Simvastatin-Induced Acute Pancreatitis: Two Cases].” *Gastroenterologie Clinique et Biologique* 15, no. 12 (1991): 986.

Ratkovic, M., N. Basic-Jukic, and D. Radunovic. “Possible Sirolimus-Induced Acute Pancreatitis in a Renal Transplant Recipient.” *Therapeutic Apheresis and Dialysis* 20, no. 2 (2016): 208–9.

Riemenschneider, T. A., J. F. Wilson, and R. L. Vernier. “Glucocorticoid-Induced Pancreatitis in Children.” *Pediatrics* 41, no. 2 (1968): 428–37.

Rion, R. J. “Recurrent Pancreatitis after Treatment with Hydrochlorothiazide.” *Journal of the American Board of Family Practice* 7, no. 1 (1994): 74–76.

Rizos, Emmanouil, Kalliopi Tournikioti, Evangelos Alevyzakis, Melpomeni Peppa, Konstantinos Papazaxos, Georgios Zorbas, Ioannis Michopoulos, Ioannis Liappas, Charalampos Papageorgiou, and Athanasios Douzenis. “Acute Necrotizing Pancreatitis Following Olanzapine Treatment and 759C/T Polymorphism of HTR2C Gene: A Case Report.” *In Vivo (Athens, Greece)* 29, no. 5 (2015): 529–31.

Roberge, R. J., T. G. Martin, M. Hodgman, and J. G. Benitez. “Acute Chemical Pancreatitis Associated with a Tricyclic Antidepressant (Clomipramine) Overdose.” *Journal of Toxicology.Clinical Toxicology* 32, no. 4 (1994): 425–29.

Roblin, X., Y. Abinader, and A. Baziz. “[Acute Pancreatitis Induced by Gliclazide].” *Gastroenterologie Clinique et Biologique* 16, no. 1 (1992): 96.

Roblin, X., F. Becot, J. M. Jacquot, A. Nairi, J. Abinader, and D. Monnet. “[Azathioprine-Induced Acute Pancreatitis].” *Annales de Gastroenterologie et d’Hepatologie* 26, no. 5 (1990): 233.

Rodier, J. M., E. Pujade-Lauraine, L. Batel-Copel, J. H. Alexandre, and A. Bernadou. “Granisetron-Induced Acute Pancreatitis [1].” *Journal of Cancer Research and Clinical Oncology* 122, no. 2 (1996): 132–33.

Rodrigo, L., M. Moreno, S. Calleja, V. Mateos, R. J. Andrade, and M. I. Lucena. “Riluzole-Induced Acute Pancreatitis [5].” *American Journal of Gastroenterology* 96, no. 7 (2001): 2268–69.

Roguedas, A. M., J. Lonceint, B. Sassolas, L. de Saint Martin, and G. Guillet. “[Acute Pancreatitis after High-Dose Interferon Therapy in a Patient with Melanoma].” *Presse Medicale (Paris, France : 1983)* 30, no. 22 (2001): 1105.

Romero Castro, R., M. Jimenez Saenz, F. J. Pellicer Bautista, S. Dominguez Palomo, and J. M. Herrerias Gutierrez. “[Acute Pancreatitis Due to 5-Aminosalicylic Acid].” *Revista Espanola de Enfermedades Digestivas : Organo Oficial de La Sociedad Espanola de Patologia Digestiva* 79, no. 3 (1991): 219–21.

Romero Ganuza, F. J. “Pancreatitis Associated with Metronidazole.” *Gastroenterologia y Hepatologia* 31, no. 4 (2008): 264–65.

Rose, E., G. de Miscault, M. Thome, and N. Boussard. “[Acute pancreatitis caused by sodium valproate. Review of the literature apropos of a case in a child].” *Pediatrie* 46, no. 12 (1991): 831–37.

Rosenfeld, G. A., A. Chang, M. Poulin, P. Kwan, and E. Yoshida. “Cholestatic Jaundice, Acute Kidney Injury and Acute Pancreatitis Secondary to the Recreational Use of Methandrostenolone: A Case Report.” *Journal of Medical Case Reports* 5 (2011): no.

Rossor, Alexander Martin, Nicola Leech, and R. Dermot Neely. “Olanzapine-Induced Chylomicronemia Presenting as Acute Pancreatitis.” *Journal of Clinical Psychopharmacology* 27, no. 4 (2007): 395–96.

Roush, M. K., R. A. McNutt, and T. F. Gray. “The Adverse Effect Dilemma: Quest for Accessible Information.” *Annals of Internal Medicine* 114, no. 4 (1991): 298–99.

Rubin, R. “Sulfasalazine-Induced Fulminant Hepatic Failure and Necrotizing Pancreatitis.” *The American Journal of Gastroenterology* 89, no. 5 (1994): 789–91.

Sabre, Alexander, Morgan Mary Guthrie, and Reza Maleknia. “Acute Necrotising Pancreatitis Derived from Low-Dose Corticosteroid Use: An Important Reminder of Clinical Management.” *BMJ Case Reports* 2015 (2015). <http://ovidsp.ovid.com/ovidweb.cgi?T=JS&PAGE=reference&D=medl&NEWS=N&AN=26150628>.

Sachedina, B., F. Saibil, L. B. Cohen, and J. Whittey. “Acute Pancreatitis Due to 5-Aminosalicylate.” *Annals of Internal Medicine* 110, no. 6 (1989): 490–92.

Sadoff, J., S. Hwang, D. Rosenfeld, L. Ettinger, and N. Spigland. “Surgical Pancreatic Complications Induced by L-Asparaginase.” *Journal of Pediatric Surgery* 32, no. 6 (1997): 860–63.

Sadoul, J. L., D. Benchimol, A. Thyss, and P. Freychet. “Acute Pancreatitis Following Octreotide Withdrawal.” *American Journal of Medicine* 90, no. 6 (1991): 763–64.

Saez-Royuela, F., E. Pacho, D. Hernandez, and G. Marin. “Acute Pancreatitis Caused by Salazopyrine.” *Gastroenterologia y Hepatologia* 11, no. 8 (1988): 434–35.

Safer, L., Karnighi A. El, S. Hochlaf, F. Bdioui, F. Halloul, and H. Saffar. “Acute Pancreatitis in a Patient Receiving Prednisone Therapy [2].” *Semaine Des Hopitaux* 71, no. 3–4 (1995): 123–24.

Sakhri, J., C. B. Salem, H. Harbi, N. Fathallah, and R. Ltaief. “Severe Acute Pancreatitis Due to Tamoxifen-Induced Hypertriglyceridemia with Positive Rechallenge.” *Journal of the Pancreas* 11, no. 4 (2010): 382–84.

Salah, A., O. Lortholary, F. Lhote, P. Cohen, and L. Guillevin. “[Acute Pancreatitis Induced by Pentamidine Isethionate in Aerosols].” *Presse Medicale (Paris, France : 1983)* 23, no. 1 (1994): 49.

Saleem, Ali Faisal, Saba Arbab, and Farah Qamar Naz. “Isoniazid Induced Acute Pancreatitis in a Young Girl.” *Journal of the College of Physicians and Surgeons--Pakistan : JCPSP* 25, no. 4 (2015): 299–300.

Salmeron, S., P. Petitpretz, C. Katlama, P. Herve, F. Brivet, G. Simonneau, P. Duroux, and B. Regnier. “Pentamidine and Pancreatitis.” *Annals of Internal Medicine* 105, no. 1 (1986): 140–41.

Samanta, S., K. Banik, and A. K. Baronia. “Emphysematous Pancreatitis Predisposed by Olanzapine.” *Indian Journal of Anaesthesia* 58, no. 3 (2014): 323–26.

Sanchez, A. J., and D. J. Boken. “Isoniazid-Associated Pancreatitis.” *Infections in Medicine* 21, no. 12 (2004): 622–23.

Sanford, K. A., J. E. Mayle, H. A. Dean, and D. S. Greenbaum. “Metronidazole-Associated Pancreatitis.” *Annals of Internal Medicine* 109, no. 9 (1988): 756–57.

Santos, B. L. dos, R. M. F. Fernandes, and F. F. Neves. “Valproic Acid-Induced Pancreatitis in an Adult.” *Arquivos de Neuro-Psiquiatria* 68, no. 1 (2010): 135–36.

Santos, J., A. Rivero, and M. Marquez. “[Acute Pancreatitis with a Fatal Evolution Due to Antimonials in Patients with Visceral Leishmaniasis and HIV Infection].” *Anales de Medicina Interna (Madrid, Spain : 1984)* 17, no. 10 (2000): 562–63.

Sasaki, M., S. Tonoda, Y. Aoki, and M. Katsumi. “Pancreatitis Due to Valproic Acid.” *Lancet (London, England)* 1, no. 8179 (1980): 1196.

Sato, K., E. Yamada, Y. Uehara, H. Takagi, and M. Mori. “Possible Role for Human Leukocyte Antigen Haplotype in Rofecoxib-Associated Acute Pancreatitis and Cholestatic Hepatitis.” *Clinical Pharmacology and Therapeutics* 80, no. 5 (2006): 554–55.

Sato, Ken, Minoru Hayashi, Mitsuyoshi Utsugi, Takahiro Ishizuka, Hitoshi Takagi, and Masatomo Mori. “Acute Pancreatitis in a Patient Treated with Micafungin.” *Clinical Therapeutics* 29, no. 7 (2007): 1468–73.

Sauleda, J., J. G. Gea, M. C. Aguar, X. Aran, M. Pasto, and J. M. Broquetas. “Probable Pentamidine-Induced Acute Pancreatitis.” *Annals of Pharmacotherapy* 28, no. 1 (1994): 52–53.

Schouwenberg, B. J. J. W., and J. Deinum. “Acute Pancreatitis after a Course of Clarithromycin.” *Netherlands Journal of Medicine* 61, no. 7 (2003): 266–67.

Schuler, D., R. Koós, T. Révész, I. Virág, and I. Gálfi. “L-Asparaginase Therapy and Its Complications in Acute Lymphoid Leukaemia and Generalized Lymphosarcoma.” *Haematologia* 10, no. 2 (1976): 205–11.

Sebastian Domingo, J. J., M. A. Simon Marco, and R. Uribarrena Echebarria. “Hepatic and Pancreatic Injury Associated with Amineptine Therapy.” *Journal of Clinical Gastroenterology* 18, no. 2 (1994): 168–69.

Seo, Ji Ho, Da Young Lee, Chang Woo Hong, In Hee Lee, Ki Sung Ahn, and Gun Woo Kang. “Severe Lactic Acidosis and Acute Pancreatitis Associated with Cimetidine in a Patient with Type 2 Diabetes Mellitus Taking Metformin.” *Internal Medicine (Tokyo, Japan)* 52, no. 19 (2013): 2245–48.

Sepulveda Vildosola, A. C., E. Lopez Aguilar, P. Yanez Lopez, R. Ramirez Colorado, B. Escobar Padilla, and A. Madrazo De la Garza. “[Sodium Diphenylhydantoin as a Probable Cause of Pancreatitis].” *Revista de Gastroenterologia de Mexico* 64, no. 4 (1999): 186–89.

Sequeira Lopes da Silva, Jose Tiago, Olga Gonzalez Casas, Veronica Bejarano Moguel, Maria Lobo Pascua, Antonio Lopez-Santamaria Redondo, and Remigio Cordero Torres. “Lanreotide Autogel-Induced Acute Pancreatitis in a Patient with Acromegaly.” *Gastroenterologia y Hepatologia* 36, no. 1 (2013): 21–25.

Sevastru, Stefan, Mai Wakatsuki, Jonathan Fennell, and Michael P. W. Grocott. “Plasma Exchange in the Management of a Case of Hypertriglyceridaemic Pancreatitis Triggered by Venlafaxine.” *BMJ Case Reports* 2012 (2012). <http://ovidsp.ovid.com/ovidweb.cgi?T=JS&PAGE=reference&D=med7&NEWS=N&AN=22892234>.

Sevencan, Nurhayat Ozkan, Aysegul Ertinmaz Ozkan, and Burcak Kayhan. “Linagliptin-Related Pancreatitis in a Diabetic Patient with Biliary Calculus: A Case Report.” *Medicine* 97, no. 50 (December 2018): e13284. <https://doi.org/10.1097/MD.0000000000013284>.

Sevenet, F., C. Sevenet, D. Capron, and P. Descombes. “Acute Pancreatitis Associated with Interferon Alpha Therapy [2].” *Gastroenterologie Clinique et Biologique* 23, no. 11 (1999): 1256.

Sevin, A., A. Chen, and B. Atkinson. “Tyrosine Kinase Inhibitor Induced Pancreatitis.” *Journal of Oncology Pharmacy Practice* 19, no. 3 (2013): 257–60.

Shafqet, M. A., T. V. Brown, and R. Sharma. “Normal Lipase Drug-Induced Pancreatitis: A Novel Finding.” *American Journal of Emergency Medicine* 33, no. 3 (2015): 476.

Shindano, A., L. Marot, and A. P. Geubel. “Nifuroxazide-Induced Acute Pancreatitis: A New Side-Effect for an Old Drug?” *Acta Gastro-Enterologica Belgica* 70, no. 1 (2007): 32–33.

Shirota, Tomoki, Toshihiko Ikegami, Satoshi Sugiyama, Kouji Kubota, Akira Shimizu, Yasunari Ohno, Atsuyoshi Mita, et al. “Successful Living Donor Liver Transplantation for Acute Liver Failure after Acetylsalicylic Acid Overdose.” *Clinical Journal of Gastroenterology* 8, no. 2 (2015): 97–102.

Siefkin, A. D. “Sulindac and Pancreatitis.” *Annals of Internal Medicine* 93, no. 6 (1980): 932–33.

Siemers, R. F., W. R. Friedenberg, and R. G. Norfleet. “High-Dose Cytosine Arabinoside-Associated Pancreatitis.” *Cancer* 56, no. 8 (1985): 1940–42.

Sinclair, D. Barry, Marjorie Berg, and Rene Breault. “Valproic Acid-Induced Pancreatitis in Childhood Epilepsy: Case Series and Review.” *Journal of Child Neurology* 19, no. 7 (2004): 498–502.

Singh, G., S. M. el-Gadi, and R. A. Sparks. “Pancreatitis Associated with Aerosolised Pentamidine.” *Genitourinary Medicine* 71, no. 2 (1995): 130–31.

Singh, H. K., M. S. Prasad, A. K. Kandasamy, and K. Dharanipragada. “Tamoxifen-Induced Hypertriglyceridemia Causing Acute Pancreatitis.” *Journal of Pharmacology and Pharmacotherapeutics* 7, no. 1 (2016): 38–40.

Siwach, V., V. Bansal, A. Kumar, U. Rao Ch, A. Sharma, and M. Minz. “Post-Renal Transplant Azathioprine-Induced Pancreatitis.” *Nephrology, Dialysis, Transplantation : Official Publication of the European Dialysis and Transplant Association - European Renal Association* 14, no. 10 (1999): 2495–98.

Slim, R., C. B. Salem, M. Zamy, N. Fathallah, J. J. Raynaud, K. Bouraoui, and M. Biour. “Secnidazole-Induced Acute Pancreatitis: A New Side-Effect for an Old Drug?” *Journal of the Pancreas* 11, no. 1 (2010): 85–86.

Sotomatsu, M., M. Shimoda, C. Ogawa, and A. Morikawa. “Acute Pancreatitis Associated with Interferon-Alpha Therapy for Chronic Myelogenous Leukemia.” *American Journal of Hematology* 48, no. 3 (1995): 211–12.

Souweine, B., J. Fialip, M. Ruivard, O. Aumaitre, J. Lavarenne, and P. Philippe. “Acute Pancreatitis Associated with Roxithromycin Therapy [3].” *DICP, Annals of Pharmacotherapy* 25, no. 10 (1991): 1137.

Soylu, Ali Riza, Gulbin Dokmeci, Ahmet Tezel, Bilge Cakir, Hasan Umit, Nesibe Karahan, and Humeyra Amuca. “Lamivudine-Induced Acute Pancreatitis in a Patient with Decompensated Hbv-Related Chronic Liver Disease.” *Journal of Clinical Gastroenterology* 38, no. 2 (2004): 134.

Srivali, N., C. Thongprayoon, W. Cheungpasitporn, and P. Ungprasert. “Acute Pancreatitis in the Use of Canagliflozin: A Rare Side-Effect of the Novel Therapy for Type 2 Diabetes Mellitus.” *Journal of Basic and Clinical Pharmacy* 6, no. 3 (2015): 101–2.

Standridge, J. B. “Fulminant Pancreatitis Associated with Lisinopril Therapy.” *Southern Medical Journal* 87, no. 2 (1994): 179–81.

Steiner, Raphael E., Robert Z. Orlowski, and Hans C. Lee. “Acute Pancreatitis Associated with Ixazomib in a Multiple Myeloma Patient.” *Acta Haematologica* 139, no. 1 (2018): 67–70. <https://doi.org/10.1159/000484655>.

Stenvinkel, P., and A. Alvestrand. “Loop Diuretic-Induced Pancreatitis with Rechallenge in a Patient with Malignant Hypertension and Renal Insufficiency.” *Acta Medica Scandinavica* 224, no. 1 (1988): 89–91.

Stephenson, I., M. J. Wiselka, and M. J. Qualie. “Acute Pancreatitis Induced by Isoniazid in the Treatment of Tuberculosis.” *The American Journal of Gastroenterology* 96, no. 7 (2001): 2271–72.

Storer, Andrew. “A 54-Year-Old Woman with a Rare Case of Drug-Induced Pancreatitis.” *Advanced Emergency Nursing Journal* 33, no. 1 (2011): 23–28.

Straumann, A., M. Bauer, W. J. Pichler, and M. Pirovino. “Acute Pancreatitis Due to Pyritinol: An Immune-Mediated Phenomenon.” *Gastroenterology* 115, no. 2 (1998): 452–54.

Subramaniam, Somasundaram, Jason A. Zell, and Pamela L. Kunz. “Everolimus Causing Severe Hypertriglyceridemia and Acute Pancreatitis.” *Journal of the National Comprehensive Cancer Network : JNCCN* 11, no. 1 (2013): 5–9.

Sue, Mariko, Aya Yoshihara, Koji Kuboki, Naoki Hiroi, and Gen Yoshino. “A Case of Severe Acute Necrotizing Pancreatitis after Administration of Sitagliptin.” *Clinical Medicine Insights.Case Reports* 6 (2013): 23–27.

Sugerman, H. J. “Sulindac-Induced Acute Pancreatitis Mimicking Gallstone Pancreatitis.” *The American Surgeon* 55, no. 9 (1989): 536–38.

Sura, M. E., K. A. Heinrich, and M. Suseno. “Metronidazole-Associated Pancreatitis.” *Annals of Pharmacotherapy* 34, no. 10 (2000): 1152–55.

Surinach, J. M., J. Alegre, T. Fernandez de Sevilla, and M. Queralt. “[Acute Pancreatitis from Erythromycin].” *Revista Clinica Espanola* 192, no. 9 (1993): 458.

Taguchi, M., M. Yokota, H. Koyano, Y. Endo, and Y. Ozawa. “Acute Pancreatitis and Parotitis Induced by Methimazole in a Patient with Graves’ Disease.” *Clinical Endocrinology* 51, no. 5 (1999): 667–70.

Tahan, V., G. Tahan, F. Dane, S. Uraz, and M. Yardim. “Acute Pancreatitis Attributed to the Use of Pegylated Interferon in a Patient with Chronic Hepatitis C [3].” *Journal of Gastrointestinal and Liver Diseases* 16, no. 2 (2007): 224–25.

Taira, N., H. Nishi, M. Mano, N. Waki, Y. Tsugita, S. Takashima, K. Fukuda, and S. Komatsubara. “Pancreatitis Induced by Valproic Acid: Report of a Case.” *Surgery Today* 31, no. 11 (2001): 1027–31.

Talamo, Giampaolo, Jeffrey Sivik, Manoj K. Pandey, and Muhammad A. Mir. “Bortezomib-Induced Acute Pancreatitis: Case Report and Review of the Literature.” *Journal of Oncology Pharmacy Practice : Official Publication of the International Society of Oncology Pharmacy Practitioners* 22, no. 2 (2016): 332–34.

Talwar, D. “Valproate-Associated Acute Pancreatitis in a Child with Neuronal Ceroid Lipofuscinosis.” *Journal of Child Neurology* 9, no. 1 (1994): 36–37.

Tan, C. L., S. P. Chiang, and K. P. Wee. “Acute Haemorrhagic Pancreatitis Following L Asparaginase Therapy in Acute Lymphoblastic Leukaemia: A Case Report.” *Singapore Medical Journal* 15, no. 4 (1974): 278–82.

Tan, W. W., E. K. Chapnick, E. I. Abter, S. Haddad, E. H. Zimbalist, and L. I. Lutwick. “Paromomycin-Associated Pancreatitis in HIV-Related Cryptosporidiosis.” *The Annals of Pharmacotherapy* 29, no. 1 (1995): 22–24.

Tannir, N. M., M. Talpaz, H. Ghazal, S. Proothi, and H. M. Kantarjian. “Acute Pancreatitis Associated with Interferon Alpha Therapy for Chronic Myelogenous Leukemia.” *Leukemia and Lymphoma* 39, no. 5–6 (2000): 647–50.

Targarona, E. M., E. Munoz, J. Puig, and C. Marco. “[Acute Pancreatitis Secondary to the Administration of 6-Mercaptopurine].” *Medicina Clinica* 95, no. 3 (1990): 116–17.

Teillet, L., S. Chaussade, B. Mory, H. Roche, D. Couturier, and J. Guerre. “[Drug-Induced Acute Pancreatitis Following Intravenous Erythomycin Antibiotherapy].” *Gastroenterologie Clinique et Biologique* 15, no. 3 (1991): 265–66.

Tenenbein, Marshall Stephen, and Milton Tenenbein. “Acute Pancreatitis Due to Erythromycin Overdose.” *Pediatric Emergency Care* 21, no. 10 (2005): 675–76.

Teng, H. W., L. Y. Bai, T. C. Chao, W. S. Wang, and P. M. Chen. “Acute Pancreatitis during All-Trans-Retinoic Acid Treatment for Acute Promyelocytic Leukemia in a Patient without Overt Hypertriglyceridemia.” *Japanese Journal of Clinical Oncology* 35, no. 2 (2005): 94–96.

Terzi, C., and S. Sokmen. “Acute Pancreatitis Induced by Magnetic-Resonance-Imaging Contrast Agent.” *Lancet (London, England)* 354, no. 9192 (1999): 1789–90.

Ting, T. W., and J. H. Lee. “Acute Pancreatitis after Propofol Infusion in a Teenage Patient.” *Anaesthesia and Intensive Care* 40, no. 3 (2012): 561–62.

Tobias, J. D., C. Capers, P. Sims, and G. W. Holcomb. “Necrotizing Pancreatitis after 10 Years of Therapy with Valproic Acid.” *Clinical Pediatrics* 34, no. 8 (1995): 446–48.

Tobias, Philip E., Christy A. Varughese, Amy P. Hanson, and Payal K. Gurnani. “A Case of Linezolid Induced Toxicity.” *Journal of Pharmacy Practice*, January 1, 2018, 897190018782787. <https://doi.org/10.1177/0897190018782787>.

Top, P. C., W. J. E. Tissing, J. W. Kuiper, R. Pieters, and C. H. J. van Eijck. “L-Asparaginase-Induced Severe Necrotizing Pancreatitis Successfully Treated with Percutaneous Drainage.” *Pediatric Blood and Cancer* 44, no. 1 (2005): 95–97.

Torosis, J., and R. Vender. “Tetracycline-Induced Pancreatitis.” *Journal of Clinical Gastroenterology* 9, no. 5 (1987): 580–81.

Torrus, D., B. Massa, V. Boix, J. Portilla, and M. Perez-Mateo. “Meglumine Antimoniate-Induced Pancreatitis.” *The American Journal of Gastroenterology* 91, no. 4 (1996): 820–21.

Toubanakis, Christos, Ekaterini Batziou, Nikolaos Sipsas, George Galanopoulos, Michael Tzivras, and Athanasios Archimandritis. “Acute Pancreatitis after Long-Term Therapy with Mesalazine, and Hyperamylasaemia Associated with Azathioprine in a Patient with Ulcerative Colitis.” *European Journal of Gastroenterology & Hepatology* 15, no. 8 (2003): 933–34.

Tragnone, A., G. Bazzocchi, G. Aversa, M. G. Pecorelli, G. Elmi, S. Venerato, and G. A. Lanfranchi. “Acute Pancreatitis after Azathioprine Treatment for Ulcerative Colitis.” *The Italian Journal of Gastroenterology* 28, no. 2 (1996): 102–4.

Tran, K., E. Froguel, R. Jian, M. Lemann, and R. Modigliani. “Acute Pancreatitis Induced by Mesalazine.” *Journal of Clinical Gastroenterology* 13, no. 6 (1991): 715–16.

Tripathy, N. R., S. Basha, R. Jain, S. Shetty, and A. Ramachandran. “Exenatide and Acute Pancreatitis.” *The Journal of the Association of Physicians of India* 56 (2008): 987–88.

Tsesmeli, N. E., K. E. Giannoulis, C. G. Savopoulos, E. E. Vretou, I. A. Ekonomou, and E. K. Giannoulis. “Acute Pancreatitis as a Possible Consequence of Metronidazole during a Relapse of Ulcerative Colitis.” *European Journal of Gastroenterology and Hepatology* 19, no. 9 (2007): 805–6.

Tsigrelis, Constantine, and C. S. Pitchumoni. “Pravastatin: A Potential Cause for Acute Pancreatitis.” *World Journal of Gastroenterology* 12, no. 43 (2006): 7055–57.

Tuon, F. F., C. M. de Fatima Guastini, and M. I. Castro Boulos. “Acute Pancreatitis Associated with Lamivudine Therapy for Chronic B Hepatitis.” *Brazilian Journal of Infectious Diseases* 12, no. 4 (2008): 263.

Twohig, Patrick, and Jaclyn Rivington. “Sorafenib-Induced Acute Pancreatitis: Case Report and Review of the Literature.” *Journal of Gastrointestinal Cancer* 50, no. 1 (March 2019): 137–42. <https://doi.org/10.1007/s12029-017-9980-3>.

Tysk, C., A. Y. Al-Eryani, and A. A. Shawabkeh. “Acute Pancreatitis Induced by Fluvastatin Therapy.” *Journal of Clinical Gastroenterology* 35, no. 5 (2002): 406–8.

Urru, S. A. M., E. Mariotti, P. Carta, S. Massidda, M. Marcias, R. Murru, P. Sanna, and E. Angelucci. “Acute Pancreatitis Following Brentuximab Vedotin Therapy for Refractory Hodgkin Lymphoma: A Case Report.” *Drugs in R and D* 14, no. 1 (2014): 9–11.

Val Antonana, Adolfo del, Michel Ble Caso, Maria D. Higon Ballester, and Juan A. Ortuno Cortes. “Lacosamide-Induced Acute Pancreatitis with Positive Rechallenge Test.” *Journal of Clinical Gastroenterology* 48, no. 7 (2014): 651.

Valencia, M. E., F. Laguna, and J. Gonzalez Lahoz. “[Nephrotic Syndrome and Acute Pancreatitis Related to Glucantime Administration].” *Anales de Medicina Interna (Madrid, Spain : 1984)* 17, no. 1 (2000): 54.

Vallianou, N., E. Geladari, K. Trigkidis, A. Skoula, and E. Kokkinakis. “Ramipril-Induced Acute Pancreatitis a Case Report and Literature Review.” *Archives of Hellenic Medicine* 34, no. 6 (2017): 821–23.

Van der Heide, H., M. A. Ten Haaft, and B. H. Stricker. “Pancreatitis Caused by Methyldopa.” *British Medical Journal (Clinical Research Ed.)* 282, no. 6280 (1981): 1930–31.

VanWalraven, A. A., M. Edels, and S. Fong. “Pancreatitis Caused by Mefenamic Acid.” *Canadian Medical Association Journal* 126, no. 8 (1982): 894.

Velicia, M. R., J. M. Gonzalez, P. Fernandez, B. Remacha, M. A. Martin, G. Sanchez, M. L. Goyeneche, and A. Caro-Paton. “[Acute Azathioprine-Induced Pancreatitis in a Female Patient with Crohn’s Disease].” *Gastroenterologia y Hepatologia* 22, no. 4 (1999): 186–87.

Venkatesh, P. G. K., and U. Navaneethan. “Azathioprine Induced Pancreatitis in a Patient with Co-Existing Autoimmune Pancreatitis and Hepatitis.” *Journal of the Pancreas* 12, no. 3 (2011): 250–54.

Ventura, C., R. Urich, S. Skinner, R. Bina, K. Y. Chuang, D. H. Van Thiel, and A. Nadir. “First Report of Telaprevir-Induced Pancreatitis.” *Digestive Diseases and Sciences* 58, no. 3 (2013): 887–88.

Veri, Kadi, Oivi Uibo, Inga Talvik, and Tiina Talvik. “Valproic Acid-Induced Pancreatitis in a 15-Year-Old Boy with Juvenile Myoclonic Epilepsy.” *Medicina (Kaunas, Lithuania)* 49, no. 11 (2013): 487–89.

Verma, Rajanshu. “Naltrexone-Associated Acute Pancreatitis.” *The Primary Care Companion for CNS Disorders* 18, no. 6 (November 24, 2016). <https://doi.org/10.4088/PCC.16l01953>.

Versleijen, M. W. J., A. H. J. Naber, N. P. Riksen, G. J. Wanten, and F. M. J. Debruyne. “Recurrent Pancreatitis after Trimethoprim-Sulfamethoxazole Rechallenge.” *The Netherlands Journal of Medicine* 63, no. 7 (2005): 275–77.

Vidal, J., E. Sacanella, E. Munoz, J. M. Miro, and S. Navarro. “Acute Pancreatitis Related to Octreotide in a Patient with Acquired Immunodeficiency Syndrome.” *Pancreas* 9, no. 3 (1994): 395–97.

Vignon, Rodolph K., Hassan Seddik, Fedoua Rouibaa, Hassane En-Nouali, Nawal Kabbaj, and Ahmed Benkirane. “Acute Pancreatitis during Pegylated Interferon Therapy in a Patient with Chronic Hepatitis B.” *Journal of Gastrointestinal and Liver Diseases : JGLD* 18, no. 4 (2009): 512.

Villamil, A., R. A. Hammer, and F. H. Rodriguez. “Edematous Pancreatitis Associated with Intravenous Pentamidine.” *Southern Medical Journal* 84, no. 6 (1991): 796–98.

Waage, Christian, Hans Carlsson, and Erik Waage Nielsen. “Olanzapine-Induced Pancreatitis: A Case Report.” *JOP : Journal of the Pancreas* 5, no. 5 (2004): 388–91.

Wang, He Hua, James Tsui, Xiao Yan Wang, Shou Sheng Liu, and Juan Li. “Bortezomib-Induced Acute Pancreatitis in a Patient with Multiple Myeloma.” *Leukemia & Lymphoma* 55, no. 6 (2014): 1404–5.

Weber, A., F. Carbonnel, N. Simon, B. Kantelip, A. Coaquette, G. Mantion, J. P. Miguet, and Martino V. Di. “Severe Acute Pancreatitis Related to the Use of Adefovir in a Liver Transplant Recipient.” *Gastroenterologie Clinique et Biologique* 32, no. 3 (2008): 247–49.

Wehmeier, Peter M., Philip Heiser, and Helmut Remschmidt. “Pancreatitis Followed by Pericardial Effusion in an Adolescent Treated with Clozapine.” *Journal of Clinical Psychopharmacology* 23, no. 1 (2003): 102–3.

Wilkinson, M. L., R. O’Driscoll, and T. J. Kiernan. “Cimetidine and Pancreatitis.” *Lancet (London, England)* 1, no. 8220 Pt 1 (1981): 610–11.

Williams, L. H., R. P. Reynolds, and J. L. Emery. “Pancreatitis during Sodium Valproate Treatment.” *Archives of Disease in Childhood* 58, no. 7 (1983): 543–44.

Wong, P. W., T. A. Dillard, and K. Kroenke. “Multiple Organ Toxicity from Addition of Erythromycin to Long-Term Lovastatin Therapy.” *Southern Medical Journal* 91, no. 2 (1998): 202–5.

Wood, G., N. Wetzig, P. Hogan, and M. Whitby. “Survival from Pentamidine Induced Pancreatitis and Diabetes Mellitus.” *Australian and New Zealand Journal of Medicine* 21, no. 3 (1991): 341–42.

Wu, F., L. Qu, Y. Tan, Y. Zhang, and C. Hu. “L-Asparaginase-Induced Severe Acute Pancreatitis in an Adult with Extranodal Natural Killer/T-Cell Lymphoma, Nasal Type: A Case Report and Review of the Literature.” *Oncology Letters* 7, no. 4 (2014): 1305–7.

Wu, S. M., and J. W. Wolf. “Pheniformin and Pancreatitis.” *Annals of Internal Medicine* 88, no. 1 (1978): 128.

Wurm, S., F. Schreiber, and W. Spindelboeck. “Mefenamic Acid: A Possible Cause of Drug-Induced Acute Pancreatitis.” *Pancreatology : Official Journal of the International Association of Pancreatology (IAP) ...[et Al.]* 15, no. 5 (2015): 570–72.

Wyllie, E., R. Wyllie, R. P. Cruse, G. Erenberg, and A. D. Rothner. “Pancreatitis Associated with Valproic Acid Therapy.” *American Journal of Diseases of Children (1960)* 138, no. 10 (1984): 912–14.

Yahiaoui, N., M. Roche, N. Aissaoui-Hoffmann, B. A. Keita, and M. Mallaret. “Intravenous Methylprednisolone Induced Acute Pancreatitis.” *European Journal of Clinical Pharmacology* 73, no. 5 (May 2017): 645–46. <https://doi.org/10.1007/s00228-017-2207-5>.

Yamada, Toshiki, Yasuhito Nannya, Masahito Shimizu, Mitsuru Seishima, and Hisashi Tsurumi. “Symptomatic Acute Pancreatitis Induced by Nilotinib: A Report of Two Cases.” *Internal Medicine (Tokyo, Japan)* 55, no. 23 (2016): 3495–97.

Yaman, Ayhan, Tanl Kendirli, Caglar Odek, Omer Bektas, Zarife Kuloglu, Meltem Kologlu, Erdal Ince, and Gulhis Deda. “Valproic Acid-Induced Acute Pancreatitis and Multiorgan Failure in a Child.” *Pediatric Emergency Care* 29, no. 5 (2013): 659–61.

Yang, M., H. Qu, and H. C. Deng. “Acute Pancreatitis Induced by Methimazole in a Patient with Graves’ Disease.” *Thyroid : Official Journal of the American Thyroid Association* 22, no. 1 (2012): 94–96.

Yang, Scott H., and Marguerite J. McNeely. “Rhabdomyolysis, Pancreatitis, and Hyperglycemia with Ziprasidone.” *The American Journal of Psychiatry* 159, no. 8 (2002): 1435.

Yazdani, K., M. Lippmann, and I. Gala. “Fatal Pancreatitis Associated with Valproic Acid: Review of the Literature.” *Medicine* 81, no. 4 (2002): 305–10.

Yen, J.M., L.F. Tan, and Y.L. Sze. “Sitagliptin Related Pancreatitis in the Elderly: A Rare but Serious Complication.” *European Geriatric Medicine* 8, no. 5–6 (November 2017): 377–78. <https://doi.org/10.1016/j.eurger.2017.07.006>.

Yi, P. H., D. R. Veltre, J. S. Kuttab, V. Rangan, and L. Norton. “Acute Groove Pancreatitis Due to Isoniazid.” *The Netherlands Journal of Medicine* 71, no. 2 (2013): 104.

Yoshizawa, Y., S. Ogasa, S. Izaki, and K. Kitamura. “Corticosteroid-Induced Pancreatitis in Patients with Autoimmune Bullous Disease: Case Report and Prospective Study.” *Dermatology (Basel, Switzerland)* 198, no. 3 (1999): 304–6.

Youssef, S. S., S. B. Iskandar, J. Scruggs, and T. M. Roy. “Acute Pancreatitis Associated with Omeprazole.” *International Journal of Clinical Pharmacology and Therapeutics* 43, no. 12 (2005): 558–61.

Yu, C. H., K. H. Lin, D. T. Lin, R. L. Chen, Y. C. Horng, and M. H. Chang. “L-Asparaginase-Related Pancreatic Pseudocyst: Report of a Case.” *Journal of the Formosan Medical Association = Taiwan Yi Zhi* 93, no. 5 (1994): 441–44.

Yutsudo, Y., S. Imoto, R. Ozuru, K. Kajimoto, H. Itoi, T. Koizumi, R. Nishimura, and T. Nakagawa. “Acute Pancreatitis after All-Trans Retinoic Acid Therapy [1].” *Annals of Hematology* 74, no. 6 (1997): 295–96.

Zimmermann, A. E., B. G. Katona, J. S. Jodhka, and R. B. Williams. “Ceftriaxone-Induced Acute Pancreatitis.” *The Annals of Pharmacotherapy* 27, no. 1 (1993): 36–37.

Zinberg, J., R. Chernaik, E. Coman, R. Rosenblatt, and L. J. Brandt. “Reversible Symptomatic Biliary Obstruction Associated with Ceftriaxone Pseudolithiasis.” *American Journal of Gastroenterology* 86, no. 9 (1991): 1251–54.

Zuger, A., B. Z. Wolf, W. el-Sadr, M. S. Simberkoff, and J. J. Rahal. “Pentamidine-Associated Fatal Acute Pancreatitis.” *JAMA* 256, no. 17 (1986): 2383–85.

Zygmunt, D. J., H. J. Williams, and S. R. Bienz. “Acute Pancreatitis Associated with Long-Term Sulindac Therapy.” *The Western Journal of Medicine* 144, no. 4 (1986): 461–62.
