# Supplementary material for: Drug induced pancreatitis: A systematic review of case reports to determine potential drug associations
Source: PLoS One. 2020 Apr 17;15(4):e0231883. doi: 10.1371/journal.pone.0231883 (PMC7164626; doi:10.1371/journal.pone.0231883)
Supplement: S4 Text — (DOCX) [file pone.0231883.s004.docx]

S4 TEXT: List of excluded full text reports

Abstract only (n = 145)

Ahmad, S. R. and Swann, J. Reporting rates of hemorrhagic/necrotizing pancreatitis (HNP) in association with selected newer antidiabetics. Pharmacoepidemiology and Drug Safety (PDS) 2009. 18 (S1) S79-S80.

Akhtar, N., Kovach, A., Fairley, K., Ahuja, N., and Komar, M. A multifactorial and simultaneous presentation of acute pancreatitis and acute hepatitis. American Journal of Gastroenterology 2014. 109:S366-S367.

Ali, M. F., Bari, M. A., Ahmed, H., Coppola, T., Morim, A., Corapi, M., and Grendell, J. H. Valproic acid-induced necrotic pancreatitis with superior mesenteric vein thrombosis: Case report and literature review. American Journal of Gastroenterology 2015. 110:S64-S65.

Alim, H., Moustafa, A., Youssef, E., Khan, Z., Raja, S., Nawras, A., and Javaid, T. Acute pancreatitis due to cephalexin: A case report and review of literature. American Journal of Gastroenterology 2017. (Supplement 1) S1518.

Alim, H., Moustafa, A., Youssef, E., Raja, S., Khan, Z., Sodeman, T., Nawras, A., and Javaid, T. Acute pancreatitis due to cephalexin: A case report and review of literature. American Journal of Gastroenterology 2017. (Supplement 1) S724-S725.

Alkinj, B., Gopalratnam, K., Kwon, J. S., and Winterbottom, C. Nivolumab: Unusual cause of severe type B lactic acidosis. American Journal of Respiratory and Critical Care Medicine 2018. Conference.

Anderson, E., Storage, T., and Park, W. A case of a young man presenting with cholestatic pancreatitis as an adverse reaction to lisinopril. American Journal of Gastroenterology 2016. 111:S557.

Anong, J., Litchfield, J., and Bajaj, K. Friend or foe, pain from my pain pills? A rare case of drug-induced acute pancreatitis. American Journal of Gastroenterology 2013. 108:S64-S65.

Aouinti, I., Kastalli, S., Hamza, I., Lakhal, M., Daghfous, R., and El, Aidli S. Interferon beta-1a-induced acute pancreatitis. Fundamental and Clinical Pharmacology 2012. 26:110.

Assanasen, C., Garcia-Frausto, E., and Rathjen, S. Multiple episodes of pancreatitis due to mercaptopurine and not asparaginase affecting therapy in pediatric leukemia. Pediatric Blood and Cancer 2012. 58 (7) 1069.

Azhari, H., Ma, C., and Kumar, P. Drug associated acute pancreatitis secondary to apixaban: A case report. Canadian Journal of Gastroenterology and Hepatology 2016.

Barkin, J. A., Nemeth, Z., Saluja, A. K., and Barkin, J. S. A systematic review of cannabis use and the development of acute pancreatitis. American Journal of Gastroenterology 2016. 111:S50-S51.

Barkin, J. A., Nemeth, Z., Saluja, A. K., and Barkin, J. S. Cannabis induced acute pancreatitis: A systematic review. Pancreas 2016. 45 (10) 1497.

Bataille, J., Markarian, M., Carlier, P., and Eftekhari, P. Acute pancreatitis reported with isotretinoin: Study in the French pharmacovigilance database. Fundamental and Clinical Pharmacology 2014. 28:50.

Bauters, T., Mondelaers, V., Robays, H., De, Wilde H., Benoit, Y., and De, Moerloose B. Methemoglobinemia and hemolytic anemia caused by rasburicase administration in a child with acute lymphoblastic Leukemia. Pediatric Blood and Cancer 2010. 55 (5) 864.

Ben, Salem F., Zaiem, A., Hamza, I., Zgolli, F., Aouinti, I., El, Aidli S., Daghfous, R., and Kastalli, S. Captopril induced acute pancreatitis associated to angioedema. Fundamental and Clinical Pharmacology 2018. (Supplement 1) 95.

Ben, Sassi M., Kastalli, S., Charfi, O., Lakhoua, G., El, Aidli S., Lakhal, M., Daghous, R., and Zaiem, A. Acute pancreatitis induced by metformin. Drug safety 2015. 38 (10) 996-997.

Bhalla, R., Lewis, M., and Bartel, M. Tumor necrosis factor alpha (TNFalpha) inhibitor class induced acute pancreatitis. American Journal of Gastroenterology 2014. 109:S312-S313.

Bharadwaj, M., Khan, Md, and Mohan, N. Single centre experience of 144 children with pancreatitis. Pancreatology: official journal of the International Association of Pancreatology (IAP) ..[et al.] 2011. 11:45.

Bloomgren, G., Dore, D., Patterson, R., Noel, R., Braun, D., and Seeger, J. Incidence of acute pancreatitis in exenatide initiators compared to other antidiabetic drug initiators: A retrospective, cohort study. Diabetes 2009. 58:no.

Bossous, P.-M. and Hanley, J. Protonix abuse: It hurts!. Journal of hospital medicine 2012. 7:S161.

Butt, L. A., Itidiare, M., and Cohen, Z. Rosuvastatin-induced multi-organ dysfunction syndrome (MODS). American Journal of Gastroenterology 2016. 111:S516.

Cabrera, R. and Caverzagie, K. Metronidazole-induced pancreatitis in a patient treated for acute diverticulitis. Journal of General Internal Medicine 2011. 26:S496.

Chahal, K. S. and Kraus, A. A rare association of acute pancreatitis with alendronate use. Journal of General Internal Medicine 2016. 31 (2 SUPPL. 1) S517-S518.

Charfi, O., Aouinti, I., Kaabi, W., Zaiem, A., Lakoua, G., Hamza, I., El, Aidli S., Daghfous, R., and Kastalli, S. Multivisceral impairment in a DRESS Syndrome. Fundamental and Clinical Pharmacology 2018. (Supplement 1) 93.

Charpentier, J., Raikhelkar, J., and Wassef, W. A preventable cause of acute pancreatitis: Olanzapine induced hypertriglyceridemia. American Journal of Gastroenterology 2010. 105:S210.

Chaudhari, D. J., Bhatheja, S., and Zakaria, W. Tigecycline: Cautionary tale. Journal of General Internal Medicine 2013. 28:S407.

Chebane, L., Bagheri, H., and Montastruc, J. L. Drug-induced pancreatitis: A study in the French pharmacovigilance database. Fundamental and Clinical Pharmacology 2013. 27:65-66.

Chhaparia, A., Hammami, M., and Schroeder, K. Eluxadoline induced pancreatitis: A case report. American Journal of Gastroenterology 2016. 111:S809.

Cinnor, B., Crossman, H., Kaplan, J., Mittal, C., Gerich, M. E., and Kao, D. J. First reported case of pembrolizumab-induced immune mediated hemorrhagic gastritis. Gastroenterology 2017. (5 Supplement 1) S891.

Cranswick, N. and Magrath, E. Pancreatitis in children: Drugs as an important cause. Journal of Paediatrics and Child Health 2010. 46:17.

Dafalla, M., Ganta, V., Hagahmed, N., Odonkor, W., and Nunlee-Bland, G. Canagliflozin and sitagliptin induced acute pancreatitis. Endocrine Practice 2018. (Supplement 1) 30.

Daifu, T., Kato, I., Ogata, H., Kamitori, T., Mikami, T., Umeda, K., Hiramatsu, H., Takahashi, H., and Adachi, S. Re-administration of all-trans retinoic acid (ATRA) after atra-induced acute pancreatitis in pediatric acute promyelocytic leukemia (APL). Pediatric Blood and Cancer 2017. (Supplement 4) S70.

Daoud, J., Dahal, K., and Wetz, R. Statin-Induced pancreatitis. Journal of hospital medicine 2010. 5:129.

Dedania, B. K., Zhang, H. C., and Miller, E. Everolimus-induced hypertriglyceridemia manifesting as acute pancreatitis. American Journal of Gastroenterology 2017. (Supplement 1) S718-S719.

Del Val, Antonana A., Ble, Caso M., Higon Ballester, M. D., and Ortuno Cortes, J. A. Lacosamide induced acute pancreatitis with positive rechallenge test. Pancreatology: official journal of the International Association of Pancreatology (IAP) ..[et al.] 2013. 13 (4 SUPPL. 1) e11.

Dickey, S. E., Hamilton, L. A., and Suda, K. J. Possible drug-induced pancreatitis in a complicated adolescent patient post-traumatic injury. Pharmacotherapy 2014. 34 (10) e288.

Dore, D. D., Hussein, M., Hoffman, C., Pelletier, E. M., Smith, D. B., and Seeger, J. D. A pooled analysis of cohort studies comparing risk of acute pancreatitis among initiators of exenatide and other antihyperglycemic drugs in two commercial health insurance claims databases. Pharmacoepidemiology and drug safety 2011. 20:S160-S161.

Duchene, B., Jaumally, B. A., and Gregoire, F. A case of tenofovir causing fanconi syndrome and pancreatitis. Chest 2017. (4 Supplement 1) A304.

Elouni, B., Vignier, N., Ben, Salem C., Zamy, M., Bouchaud, O., and Biour, M. Amiodarone-induced mild acute pancreatitis. Drug safety 2010. 33 (10) 906-907.

Enger, C., Gately, R., Niemcryk, S. J., Ming, E. E., and McAfee, A. T. Pharmacoepidemiology safety study of statin, fibrate, or statin-fibrate combination therapy. Pharmacoepidemiology and drug safety 2010. 19:S268.

Eross, B., Meczker, A., Miko, A., and Hegyi, P. 5-ASA induces mild acute pancreatitis: A case report. Pancreatology: official journal of the International Association of Pancreatology (IAP) ..[et al.] 2018. (4 Supplement 1) S17.

Eto, H., Kawabe, K., Miyahara, Y., Fukuda, H., Kasai, T., Muramatsu, S., and Ito, H. Drug-induced pancreatitis diagnosed by Mesalazine challenge test: Case report. Journal of Gastroenterology and Hepatology (Australia) 2016. 31:255.

Fathallah, N., Chemchik, H., Slim, R., Naija, W., Ben, Salem C., and Bouraoui, K. Fatal furosemide-induced acute pancreatitis with positive rechallenge. Fundamental and Clinical Pharmacology 2011. 25:38.

Fernandez, S. and Calvo, G. A case report: Pancreatitis in patient with crohn's disease. Basic and Clinical Pharmacology and Toxicology 2016. (Supplement 1) 35.

Ferreira, A. M., Santos, J., Ferreira, S., Campos, R. A., and Leite, A. L. Sertraline induced acute pancreatitis in a diabetic adolescent?. Pediatric diabetes 2017. (Supplement 25) 143.

Folz, H. Acute pancreatitis episode after dose increase of dulaglutide in a patient with a remote history of pancreatitis: A case report. JACCP Journal of the American College of Clinical Pharmacy 2018. (2) 342-343.

Gaiffe, A., Caissutti, J., Valnet-Rabier, M. B., and Davani, S. Acute pancreatitis and phosphodiesterase 5 inhibitors: A first case report. Fundamental and Clinical Pharmacology 2016. (Supplement 1) 52-53.

Garrido, J. P., Thakore, S., Zohra, F., and Islam, A. My heart was fixed but my tummy hurts! contrast induced pancreatitis following cardiac catheterization. Journal of Investigative Medicine 2015. 63 (2) 338.

Gatta, L., Corti, F., Giunta, S., Ceccarelli, G., and Scarpignato, C. Ethinylestradiol/levonorgestrel oral contraceptive and acute pancreatitis: A case series. Digestive and Liver Disease 2017. (Supplement 2) e155-e156.

Gino, M. and Mete, F. A rare case of acute pancreatitis. Italian Journal of Medicine 2015. 9:47.

Gorges, Rony, Ghalayini, Wael, and Zughaib, Marcel. A case of contrast-induced pancreatitis following cardiac catheterization. The Journal of invasive cardiology 2013. 25 (10) E203-E204.

Gubergrits, N. and Klochkov, O. Treatment of drug-induced pancreatitis in patients with pulmonary tuberculosis. Pancreatology: official journal of the International Association of Pancreatology (IAP) ..[et al.] 2013. 13 (3 SUPPL. 1) S33-S34.

Gubergrits, N., Lukashevich, G., Klochkov, A., and Byelyayeva, N. Effectiveness of staged antihomotoxical therapy of drug-induced pancreatitis caused by antituberculous (anti-TB) preparations. Pancreatology: official journal of the International Association of Pancreatology (IAP) ..[et al.] 2015. 15 (3 SUPPL. 1) S54.

Gurudath, G. S., Sharath Kote, G. S., Ramesh Kumar, T. S., Khan, M. J., Palanivelu, C., Sharma, N., Palve, S., Piyush, and Suganya. Valproic acid induced pancreatitis with hepatic infarct- A rare vascular complications. Indian Journal of Gastroenterology 2016. 35 (1 Supplement) A92.

Gweon, T.-G., Kim, J.-H., Kim, J.-I., and Park, S.-H. Acute pancreatitis secondary to ciprofloxacin therapy in patients with infectious colitis. Journal of gastroenterology and hepatology 2012. 27:376-377.

Hamza, I., Aouinti, I., Charfi, O., Zaiem, A., Ben, Salem F., Daghfous, R., El, Aidli S., and Kastalli, S. Interferon beta-1b-induced acute pancreatitis. Fundamental and Clinical Pharmacology 2017. (Supplement 1) 64.

Hasanin, M., Rasheed, K., and Baig, M. Doxycycline-induced pancreatitis. American Journal of Gastroenterology 2014. 109:S316.

Hiroi, N., Sue, M., Oda, K., Oka, Y., Shigemitsu, R., Iga, R., Yoshihara, A., Usui, S., Kuboki, K., and Yoshino, G. A case of steroid diabetes with severe acute pancreatitis after liraglutide and bezafibrate administration. Endocrine Reviews 2012. 33 (3 MeetingAbstracts) no.

Homenko, D. and Viveiros, K. Acute pancreatitis within hours of 6-mercaptopurine use. Inflammatory bowel diseases 2009. 15:S6-S7.

Huq, S. N., Ravi, B., and Terner, S. When rare becomes common: Acute pancreatitis in chronic marijuana use. Journal of General Internal Medicine 2016. 31 (2 SUPPL. 1) S791-S792.

Ilaiwy, A., Ramreddy, N., and George, R. Pancreatitis associated with a new haart. Journal of General Internal Medicine 2017. (2 Supplement 1) S558.

Issa, D., Kumar, V., Dessie, S., and Bou-Haidar, D. Acute pancreatitis secondary to the use of anabolic steroids. American Journal of Gastroenterology 2017. (Supplement 1) S690.

Itidiare, M., Rahman, A., Nathan, R. S., and Ashraf, S. Rosuvastatin-induced pancreatitis. American Journal of Gastroenterology 2015. 110:S106.

Iwamoto, S., Yin, A., Tan, S., and Swenson, S. L. Clomiphene-induced hypertriglyceridemia and acute pancreatitis. Journal of General Internal Medicine 2013. 28:S308-S309.

Jacob, J., Patel, K., Allen, P., and Sellars, M. Pancreatitis in a pediatric population-it's not just gallstones. Pediatric radiology 2011. 41:S336.

Jaglall, N., Daoud, J., Dahal, K., Seminara, D., and Wetz, R. Pancreatitis caused by a statin. American Journal of Gastroenterology 2010. 105:S216.

Jain, V., Sharma, D., Ramasamy, V., Anklesaria, A., and Goswami, G. A case of exenatide-induced recurrent pancreatitis despite withdrawl of drug therapy. American Journal of Gastroenterology 2014. 109:S291.

Jalal, T. and Ramchandani, H. Cannabis abuse: A rare cause of acute pancreatitis. Journal of General Internal Medicine 2013. 28:S304.

Jang, D. K. and Lee, J. K. Drug-induced acute pancreatitis: A hospital-based analysis. Journal of gastroenterology and hepatology 2017. (Supplement 3) 202.

Jinnur, P., Vasudevan, V., Ali, R., Arjomand, F., Tiwary, T., Vanam, V., and Abbas, Q. A rare case of acetaminophen induced acute pancreatitis, ARDS, and anion gap metabolic acidosis. Chest 2012. 142 (4 SUPPL. 1) no.

Joyau, C., Darnis, D., Hauet, Q., Mahe, J., Veyrac, G., and Jolliet, P. Complicated valproic acid-induced pancreatitis: A case report. Fundamental and Clinical Pharmacology 2013. 27:121.

Junga, Z. C., Shah, N., and Betteridge, J. A novel presentation of 6-mercaptopurine toxicity in a patient with IBD. American Journal of Gastroenterology 2015. 110:S287-S288.

Karkee, A., Sharma, A., Masood, U., Pattar, S., Grewal, S., Badar, Z., and Hess, M. Unusual case of pancreatitis with normal lipase and amylase. American Journal of Gastroenterology 2016. 111:S510-S511.

Khodasevich, D. and Tenner, S. Evidence shows few drugs cause acute pancreatitis. American Journal of Gastroenterology 2016. 111:S13.

Kim, J.-Y., Jang, D. K., Lee, J. K., and Lee, J. H. Causative agents of drug-induced pancreatitis: A national wide assessment. Journal of Gastroenterology and Hepatology (Australia) 2016. 31:255.

Kohli, D., Rashti, F., and Patel, M. Terbinafine-induced acute pancreatitis. American Journal of Gastroenterology 2012. 107:S326.

Korpaisarn, S. and Kirali, S. Liraglutide induced life-threatening pancreatitis. Endocrine Reviews 2015. 36:no.

Kourti, M., Hatzipantelis, E., Tragiannidis, A., Papageorgiou, T., and Athanassiadou, F. Successful treatment of L-asparaginase induced pancreatitis with octreotide in a child with acute lymphoblastic Leukemia. Haematologica 2010. 95:494.

Krishnan, S. K., Dandachi, D., Parikh, M., and Friedman, H. Well known, but least suspected-a case of furosemide-induced pancreatitis. Journal of General Internal Medicine 2013. 28:S421.

Kriska, M., Hudec, R., Sallstedt, L., and Hill, R. Oseltamivir and pancreatitis. Drug safety 2011. 34 (10) 915.

Kuttab, J. S., Veltre, D., Yi, P., Rangan, V., and Norton, L. E. Isoniazid, a rare cause of acute pancreatitis. Journal of General Internal Medicine 2012. 27:S444-S445.

Lee, E., Javid, G., Weinstein, B., and Lee, H. Metformin-associated acute hepatitis and pancreatitis. American Journal of Gastroenterology 2011. 106:S296.

Lee, P. H., Stockton, M. D., and Franks, A. S. Acute pancreatitis associated with liraglutide. Annals of Pharmacotherapy 2011. 45 (4) e22.

Lee, S. J., Cha, B. H., Lee, H. H., Jeon, W. J., and Kim, Y. N. A case of acute pancreatitis with pseudocyst induced by isoniazid. Journal of gastroenterology and hepatology 2011. 26:241.

Lightbourne, T., Najafian, N., and McCarty, T. R. Consideration for empagliflozin-associated pancreatitis. American Journal of Gastroenterology 2017. (Supplement 1) S710.

Lorenz, K., Abuazab, M., Sekulla, C., and Dralle, H. Calcitonin stimulation testing and the risk of pancreatitis. Langenbeck's archives of surgery 2014. 399 (8) 1087.

Marsille, F., Guy, C., Beyens, M., Mounier, G., Celarier, T., and Mismetti, P. Clopidogrel: Acute pancreatitis (AP), a case report. Fundamental and Clinical Pharmacology 2011. 25:60.

McAuliffe, A. and Shimanovsky, A. Severe acute pancreatitis in a patient with non small cell lung carcinoma on alectinib. Journal of General Internal Medicine 2018. (2 Supplement 1) 616.

McCarty, T. R. and Sack, J. Bupropion-associated, drug-induced pancreatitis in a patient with bulimia nervosa. American Journal of Gastroenterology 2016. 111:S591-S592.

Meftah, A., M'zah, D., Rahmoune, I., Filali, H., and Hakkou, F. Drug-induced acute pancreatitis: A report of four cases. Fundamental and Clinical Pharmacology 2012. 26:113.

Mezghani, M. A., Turki, M., Mhiri, A., Khrouf, M., Maaloul, I., Marrekchi, C., Hammami, S., Elleuch, E., Zghal, K., and Ben, Jemaa M. Association of an antituberculosis drug-induced fulminant hepatitis and an omeprazole drug-induced pancreatitis. Drug safety 2013. 36 (9) 937.

Mikityanskiy, Y., Ross, C., and Grendell, J. Clomiphene citrate-induced acute pancreatitis in a male without hypertriglyceridemia. American Journal of Gastroenterology 2016. 111:S510.

Moltenis, M., Pascual, M., Valnet-Rabier, M. B., and Davani, S. Massive hypertriglyceridemia during immunosuppressive therapy. Fundamental and Clinical Pharmacology 2015. 29:68.

Mostafa, A. M. T., Vearrier, D., and Greenberg, M. I. Asymptomatic lipase elevation in a patient with proton pump inhibitor overdose. Clinical toxicology 2018. (10) 1013.

Moy, B. and Kapila, N. Doxycycline-induced pancreatitis. American Journal of Gastroenterology 2014. 109:S300.

Muthukani, S., Prabhu, P. R., Suresh, P., Karthick, S. N., Rakesh, R. B., and Meenakshi-Sundaram, S. Beware of another a after alcohol: Anticonvulsantinduced pancreatitis. Annals of Indian Academy of Neurology 2014. 17:S224.

Nair, V., Gnanabakthan, N., and John, D. Docetaxel-induced acute pancreatitis. American Journal of Gastroenterology 2015. 110:S113-S114.

Napier, Sarah and Thomas, Matthew. 36 year old man presenting with pancreatitis and a history of recent commencement of Orlistat case report. Nutrition journal 2006. 5:19.

Niu, G. and Zhang, X. A case of drug-induced acute pancreatitis. Pancreatology: official journal of the International Association of Pancreatology (IAP) ..[et al.] 2016. 16:S46.

Olayode, A. and Kizer, R. Acute pancreatitis secondary to sorafenib use. American Journal of Gastroenterology 2013. 108:S262.

Oman, Z., Gu, L., and Bourdillon, M. A case of drug induced pancreatitis. Journal of Investigative Medicine 2018. (4) 828-829.

Ommurugan, B., Holla, S., Bairy, K., and Kavitha, S. Trimethoprim-sulfamethoxazole: A rare cause of drug induced. Indian Journal of Physiology and Pharmacology 2015. 59 (5 SUPPL. 1) 132.

Ormeci, A. R., Koca, T. G., Dereci, S., Koca, Y. S., and Akcam, M. An adolescent with abdominal pain taking isotretinoin for severe acne. Archives of disease in childhood 2017. (Supplement 2) A119-A120.

Orsi, D., Dudaie, R., and Dicpinigaitis, P. Acute pancreatitis associated with metformin toxicity. Critical care medicine 2010. 38:A271.

Patel, K., Pikas, E., and George, T. Drug-induced necrotizing pancreatitis with a focus on canagliflozin. American Journal of Gastroenterology 2016. 111:S557-S558.

Patel, K., Shah, S., and George, T. A cause of pancreatitis in a commonly used antibiotic: Metronidazole-induced pancreatitis. American Journal of Gastroenterology 2017. (Supplement 1) S1522.

Patel, N., Abraham, B., and Weatherly, J. The role of methotrexate in azathioprine and 6-mercaptopurine induced pancreatitis in inflammatory bowel disease patients: A case series. Inflammatory bowel diseases 2013. 19:S29.

Patel, V., Attia, K., Pattisapu, A., and Weiss, J. Nilotinib: A case of acute pancreatitis in a patient with chronic myeloid leukemia. American Journal of Gastroenterology 2015. 110:S56.

Quintanilla-Flores, D. L., Flores-Caballero, M. A., Rodriguez-Gutierrez, R., Tamez-Perez, H. E., and Gonzalez-Gonzalez, J. G. Acute pancreatitis and diabetic ketoacidosis following L-asparaginase/ prednisone therapy in acute lymphoblastic leukemia. Case Reports in Oncological Medicine 2014. 2014:no.

Rassameehiran, S., Mankongpaisarnrung, C., and Rakvit, A. An uncommon cause of drug-induced pancreatitis: Doxycycline. Journal of Investigative Medicine 2014. 62 (2) 456-457.

Robin, P., Bres, V., Porokhov, B., Hillaire-Buys, D., Petit, P., and Faillie, J. L. Focus on medical history in incretin-based drugs inducing pancreatitis in patients with type 2 diabetes mellitus. Fundamental and Clinical Pharmacology 2014. 28:51.

Rodriguez Jimenez, C. M., Sanchez-Colomer, M. G., Quintana, E. F., Fernandez Del Campo, C. B., and Del Mar Garcia, Saiz M. Pancreatitis by omeprazole. An adverse drug reaction to consider from a clinical perspective?. Basic and Clinical Pharmacology and Toxicology 2014. 115:35.

Rose, F. T., Oparaji, J.-A., Orabi, A., Howard, A. S., Okafor, D. C., Turner, R., Lowe, M. E., Ritchey, K. A., and Husain, S. Z. Risk factors for asparaginase associated pancreatitis: A systematic review. Pancreas 2016. 45 (10) 1535.

Rubio, Guindulain E., Valdivielso, Cortazar E., Bolado, Concejo F., Rodriguez, Gutierrez C., Prieto, Martinez C., Nantes, Castillejo O., and Urman Fernandez, J. M. Acute pancreatitis caused by azathioprine/mercaptopurine in patients with inflammatory bowel disease. Pancreatology: official journal of the International Association of Pancreatology (IAP) ..[et al.] 2013. 13 (4 SUPPL. 1) e14.

Ruellan, A. L., Veyrac, G., Joyau, C., Le, Vallier M., and Jolliet, P. Acute pancreatitis after morphine sulfate ingestion: Report of two cases. Drug safety 2014. 37 (10) 857.

Sapp, A., Nawaz, M., Jafri, R., Hamad, F., and Shilman, A. A rare case of medication-induced pancreatitis. American Journal of Gastroenterology 2014. 109:S100.

Sassier, M., Fedrizzi, S., Do, P., Gervais, R., and Coquerel, A. Case report of an acute pancreatitis with Doxycycline administered for cutaneous adverse event induced by Erlotinib. Fundamental and Clinical Pharmacology 2015. 29:50.

Selvi-Sabater, P., Rizo-Cerda, A. M., Sanchez-Martinez, I., Manresa-Ramon, N., Arribas-Diaz, B., Alonso-Dominguez, M. T., Gorostiza-Frias, I., Sanchez-Catalicio, M. M., Titos-Arcos, J. C., and Espuny-Miro, A. Possible telaprevir-induced pancreatitis. A case study. European Journal of Hospital Pharmacy 2014. 21:A108.

Servillas, E., Soliz, M., and Olson, J. C. Linezolid toxicity mimics septic shock in a subacute rehab patient. Journal of the American Geriatrics Society 2018. (Supplement 2) S26.

Sethi, S., Kutait, A., and Musleh, S. A rare case of isoniazid-induced pancreatitis. Pancreas 2009. 38 (8) 1045-1046.

Sevcikova, A., Zboril, V., Prokopova, L., and Novotny, I. Azathioprine and induction of acute pancreatitis in inflammatory bowel disease patients. Pancreatology: official journal of the International Association of Pancreatology (IAP) ..[et al.] 2009. 9 (4) 513-514.

Shuster, J. New antifungal as a potential cause of severe thrombocytopenia - Pancreatitis due to ACE inhibitor - Recurrent hepatitis due to cetirizine - Long-term cardiovascular risks associated with protease inhibitors - Pure red cell aplasia and epoetin therapy - Pure red cell aplasia redux: Interferon therapy - Excellent drug reaction reviews. Hospital Pharmacy 2004. 39 (12) 1148.

Soota, K., Telfah, M., Ramesh, N., Pereira, M., and Lingutla, D. Dipeptidyl peptidase-4 inhibitor-induced acute pancreatitis: A complication well documented but under recognized. American Journal of Gastroenterology 2013. 108:S262.

Stein, R. and Kelsen, J. The heterogeneous presentation of pancreatitis in pediatric inflammatory bowel disease. Journal of pediatric gastroenterology and nutrition 2016. 63:S85-S86.

Stone, J. and Puri, N. Mirtazapine induced pancreatitis with associated hypertriglyceridemia and diabetic ketoacidosis. Chest 2014. 145 (3 MEETING ABSTRACT) no.

Suryapranata, H. and De, Vries H. Pancreatitis associated with sulphasalazine. British medical journal 1986. 292 (6522) 732.

Suzuki, Y. A case of mitochondrial diabetes with tRNA Leu(UUR) mutation at position 3271 and autoimmune pancreatitis after SGLT2 inhibitor treatment. Journal of Diabetes Investigation 2016. 7:44.

Toprak, S. K., Ocal, S., Erismis, B., Yildirim, E., Altun, R., Karakus, S., Tek, I., and Topcuoglu, P. Acute pancreatitis following VAD chemotherapy combination consisting of vincristine, doxorubicin, and dexamethasone in a newly diagnosed multiple myeloma patient: A case report. Internet Journal of Oncology 2012. 8 (2) no.

Umar, J., Shah, R., Manocha, D., and Rawlins, S. Lamivudine-induced acute pacreatitis: A unique case. American Journal of Gastroenterology 2015. 110:S74.

Urbanek, K., Vinklerova, I., Hajdu, D., and Prochazka, V. Focused Conference Group: PW02 - Symposium on advances in gi pharmacology incidence, severity and aetiology of drug-induced acute pancreatitis. Basic and Clinical Pharmacology and Toxicology 2010. 107:627.

Virk, M., Anand, S., Changela, K., and Shah, S. Isolated acute pancreatitis in the absence of liver damage: Unreported complication of acetaminophen toxicity. American Journal of Gastroenterology 2012. 107:S325.

Vyas, N., Alkhawam, H., Sogomonian, R., Ching Companioni, R. A., Tiba, M., and Walfish, A. Pegaspargase induced severe pancreatitis. Friend or foe?. Journal of Investigative Medicine 2016. 64 (4) 942.

Wazir, T., Oliver, N., Corrigan, C., and Bell, A. L. Two case reports of acute pancreatitis associated with tocilizumab therapy for rheumatoid arthritis (RA). Irish Journal of Medical Science 2013. 182:S84-S85.

Weiss, M. Propofol-induced hypertriglyceridemia as a cause of stroke. Journal of Clinical Lipidology 2017. (3) 798-June.

Wenten, M., Gaebler, J., Hussein, M., Pelletier, E. M., Smith, D., Girase, P., Noel, R., Bloomgren, G., Braun, D., and Boies, L. A retrospective cohort study to assess the relative risk of acute pancreatitis among initiators of exenatide compared to initiators of other antidiabetic medication: A follow-up study. Diabetes 2010.:no.

West, W. A. and Uwaifo, G. I. Hemochromatosis and cystic fibrosis carriers identify risk for GLP-1 agonist associated pancreatitis. Diabetes 2018. (Supplement 1) A599.

Whittington, K. M. and Antolovic-Stanfel, N. Olanzapine-induced asymptomatic pancreatitis: A case report. Journal of pharmacy practice 2010. 23 (2) 186.

Williams, J. and Hernandez, L. Hepatitis and pancreatitis associated with the use of trimetropim sulfamethoxazole: A case report. American Journal of Gastroenterology 2013. 108:S326.

Wong, K. and Nabeel, M. A rare culprit in pancreatitis. American Journal of Gastroenterology 2015. 110:S64.

Wu, B. U. and Liu, I.-L. Risk of acute pancreatitis among patients taking commonly prescribed 'pancreatitis-associated' medications: A regional-population based study. Gastroenterology 2013. 144 (5 SUPPL. 1) S276.

Yadav, L., Tariq, T., Khalid, S., and Driscoll, R. Eluxadoline-induced recurrent pancreatitis in a young female without a gallbladder. American Journal of Gastroenterology 2017. (Supplement 1) S969.

Yakubov, S., Tin, K., Soe, E. P., Khodorskiy, D. O., Kadkhodayan, K., Tsirlin, Y., Mayer, I. E., and Rahmani, R. Glucagon-like peptide-1 receptor agonists, dipeptidyl peptidase-4 inhibitors, and risk of acute pancreatitis: A meta-analysis of randomized clinical trial and review of literature. American Journal of Gastroenterology 2015. 110:S10.

Yi, F. and Xia, B. Mesalazine induced acute pancreatitis in a patient with ulcerative colitis. Journal of gastroenterology and hepatology 2012. 27:160.

Zayouna, N., Dudar, A., Iskanderian, Z., Stawick, L., and Ahsan, S. Acute pancreatitis secondary to marijuana use. American Journal of Gastroenterology 2012. 107:S339.

Language other than English, French, or Spanish (n = 132)

Adachi, S., Akiyama, Y., Takimoto, T., Tanizawa, A., Kiriyama, Y., Kubota, Y., and Mikawa, H. [Aclarubicin-related pancreatitis in a child with AML]. [Rinsho ketsueki] The Japanese journal of clinical hematology 1988. 29 (3) 385-388.

Barros, H. M., Barros, E. J., and Carvalhaes, J. T. [Acute pancreatitis associated with the use of sodium valproate]. AMB: revista da Associacao Medica Brasileira 1986. 32 (1-2) 33-34.

Bartuzi, Z., Gawronska-Ukleja, E., Kuzminski, A., and Poplawski, C. Acute pancreatitis - Adverse effect of 5-aminosalicylic acid in a patient with ulcerative colitis. Gastroenterologia Polska 2005. 12 (4) 361-363.

Battillocchi, B., Salvio, A., Vermeil, V., Stefanini, S., D'Amore, L., Dandolo, R., and Negro, P. [Acute severe pancreatitis caused by anti-HIV drugs]. Annali italiani di chirurgia 2002. 73 (4) 439-3.

Battillocchi, Barbara, Diana, Massimo, Dandolo, Rossella, Stefanini, Silvano, D'Amore, Linda, and Negro, Paolo. [Drug-induced acute pancreatitis: a personal contribution]. Chirurgia italiana 2002. 54 (5) 605-612.

Becker, C., Hvalic, C., Delmore, G., Krahenbuhl, S., and Schlienger, R. [Recurrent acute pancreatitis during pravastatin-therapy]. Praxis 2006. 95 (4) 111-116.

Boros, G. and Horvath, L. [Prednisolone pancreatitis]. Orvosi hetilap 1966. 107 (48) 2283-2285.

Borys-Iwanicka, A., Jamer, T., and Iwanczak, B. Acute pancreatitis in two children treated with valproic acid due to epilepsy. Pediatria Polska 2016. 91 (6) 619-622.

Brinkmann, O. H., Hardinghaus, W., Wimmer, G., and Junge-Hulsing, G. [Acute intermittent porphyria with pancreatitis and myocardial damage due to oral contraceptives]. ZFA.Zeitschrift fur Allgemeinmedizin 1979. 55 (22) 1227-1233.

Bruusgaard-Mouritsen, Mads Emil, Leerhoy, Bonna, and Hansen, Mark Berner. [Acute pancreatitis after ingestion of ibuprofen in a 16-year-old boy]. Ugeskrift for laeger 2015. 177 (28) .

Ceuppens, A., Cool, M., Deboever, G., and Lambrecht, G. Azathioprine-induced acute pancreatitis. Tijdschrift voor Geneeskunde 2017. 73 (16) 973-976.

Chen, C. C. and Yeh, S. P. Fatal pancreatitis occurred in a patient with refractory CD30+ anaplastic large cell lymphoma after brentuximab vedotin treatment. Journal of Cancer Research and Practice 2017. 4 (1) 35-37.

Chen, Zaisheng and Li, Jian. [Experience in diagnosis and treatment of asparaginase-associated pancreatitis in children]. Zhonghua er ke za zhi = Chinese journal of pediatrics 2014. 52 (11) 854-858.

Chojnacki, C., Romanowski, M., and Wachowska-Kelly, P. Psychosomatic complications during treatment for ulcerative colitis. Przeglad Gastroenterologiczny 2012. 7 (1) 52-55.

Chung, B. H., Nam, H. S., Kwon, J. H., Im, S. H., Kim, J. W., Kim, S. J., Lee, S. Y., Kim, Y. K., and Park, S. H. A Case of Isoniazid Induced Acute Pancreatitis. Tuberculosis and Respiratory Diseases 2004. 56 (4) 411-414.

Czyzykowski, Rafal, Polowinczak-Przybylek, Joanna, Janiak, Anna, Herman, Jerzy, and Potemski, Piotr. Tamoxifen-induced acute pancreatitis - a case report. Przeglad menopauzalny = Menopause review 2014. 13 (1) 70-72.

de Jongh, F. E., Ottervanger, J. P., and Stuiver, P. C. [Acute pancreatitis caused by metronidazole]. Nederlands tijdschrift voor geneeskunde 1996. 140 (1) 37-38.

Eberhardt, H., Herterich, R., and Hofweber, K. Development of an acute pancreatitis and hapatopathy during amiodarone therapy. Monatsschrift fur Kinderheilkunde 2001. 149 (2) 147-149.

Eckardt, V. F., Kanzler, G., Rieder, H., and Ewe, K. [Pancreatitis associated with 5-aminosalicylic acid]. Deutsche medizinische Wochenschrift (1946) 1991. 116 (14) 540-542.

Eland, I. A., De Gooyer, D. J., Stokkers, P., Westerveld, B. D., Wilson, J. H. P., and Stricker, B. H. C. Acute pancreatitis attributed to use of mesalazine. Tijdschrift voor Geneeskunde 1998. 54 (6) 417-420.

Engelmann, M. D. M., Henriksen, S. D., and Tingsgaard, L. K. Fatal haemorrhagic pancreatitis associated with sodium valproate. Ugeskrift for laeger 1995. 157 (31) 4357-4358.

Engelmann, M. D., Henriksen, S. D., and Tingsgaard, L. K. [Fatal pancreatitis associated with valproate therapy]. Ugeskrift for laeger 1995. 157 (31) 4357-4358.

Faintuch, J., Chaib, E., Mott, C. B., Calache, J. E., and Machado, M. C. [Pancreatic complications of sulfasalazine]. Revista do Hospital das Clinicas 1986. 41 (2) 87-90.

Farhoud, S., Stephani, S. M., and Bromberg, S. H. [Acute pancreatitis due to intramural hematoma of the duodenum by the use of anticoagulants]. Arquivos de gastroenterologia 2001. 38 (1) 53-56.

Francobandiera, G., Rondalli, G., Telattin, P., Cattalini, N., Cocciolo, M., and Colli, A. Rare pancreatic side effects of clozapine: Two case reports. Rivista di Psichiatria 1999. 34 (5) 270-273.

Gabryelewicz, A., Bankowski, E., and Chmielewski, J. [Acute pancreatitis as a complication of long-term therapy with adrenal cortex hormones]. Polskie Archiwum Medycyny Wewnetrznej 1970. 44 (2) 173-176.

Glintborg, B. Pancreatitis in a patient with Crohn's disease treated with mesalazine and azathioprine. Ugeskrift for laeger 2000. 162 (34) 4553-4554.

Greif, J. and Erdmann, E. [Life-threatening interaction between azathioprine and allopurinol]. Der Internist 1985. 26 (11) 717-719.

Gubergrits, N. B., Lukashevich, G. M., and Zagorenko, Iu A. [Antibiotics in pancreatology: pros and contras]. Klinicheskaia meditsina 2006. 84 (2) 56-61.

Gul, M., Erdemir, E., Cander, B., Girisgin, S., Ergin, M., and Kocak, S. Case presentation of acute pancreatitis related to steroid therapy. Akademik Acil Tip Olgu Sunumlari Dergisi 2013. 4 (1) 35-37.

Guo, Rong, DU, Xin, Weng, Jian Yu, Deng, Cheng Xin, Wu, Sui Jing, and Luo, Cheng Wei. [Acute pancreatitis induced by cyclosporine a following allogeneic hematopoietic stem cell transplant]. Zhongguo shi yan xue ye xue za zhi 2009. 17 (2) 472-475.

Gyorgy, J. Incretin-based antidiabetic treatment and diseases of the pancreas (pancreatitis, pancreas carcinoma). Orvosi hetilap 2016. 157 (14) 523-528.

Gyorgy, J., Geza, S., and Gyorgy, B. [A case of lupus pancreatitis (?) in systemic lupus erythematosus]. Morphologiai es igazsagugyi orvosi szemle 1976. 16 (3) 221-224.

Hagi, T. T., Stirnimann, G., Stutz, A., and Lang, N. P. Akute Pankreatitis nach nicht chirurgischer Parodontaltherapie in Kombination mit systemischer Antibiotikatherapie. Swiss Dent J 2017. 127 (4) 315-323.

Hansen, L. F., Nordling, M. M., and Mortensen, H. B. Pancreatitis after MMR vaccination. Ugeskrift for laeger 2003. 165 (22) 2305-2306.

Hesselmann, J. [Acute pancreatitis from codeine preparations]. Deutsche medizinische Wochenschrift (1946) 1984. 109 (8) 317.

Hesselmann, J. Acute pancreatitis due to codeine-containing remedies. Deutsche Medizinische Wochenschrift 1984. 109 (8) 317.

Hisada, S., Shiratori, K., Shimizu, K., Hoshino, Y., Tsuchiya, N., Hayashi, N., and Mizoguchi, H. A case of L-asparaginase-induced severe acute pancreatitis. Japanese Journal of Gastroenterology 2001. 98 (12) 1374-1378.

Hsu, Yu Juei, Chen, Yeu Chin, Ho, Ching Liang, Kao, Woei Yau, and Chao, Tsu Yi. Diabetic ketoacidosis and persistent hyperglycemia as long-term complications of L-asparaginase-induced pancreatitis. Zhonghua yi xue za zhi = Chinese medical journal; Free China ed 2002. 65 (9) 441-445.

Imada, M. [Case of pancreatitis following cortico-steroid therapy of nephrotic syndrome]. Nihon Shonika Gakkai zasshi.Acta paediatrica Japonica 1966. 70 (2) 96-102.

Ishii, Y., Kojima, H., Ann, T., Ohura, H., Asada, K., and Uemura, M. Prednisolon-induced acute pancreatitis in an adult patient with idiopatic thrombocytopenic purpura. Japanese Journal of Gastroenterology 2003. 100 (4) 459-464.

Ishii, Y., Yoshikawa, M., Matsumoto, M., Kikuchi, E., Masui, K., Mizumoto, Y., Umemoto, N., Tsujinoue, H., Tamagawa, T., Kawata, M., Kawamoto, H., Ueda, S., Orihashi, T., Mitoro, A., and Fukui, H. A case of acute pancreatitis induced by interferon therapy for chronic hepatitis C. Japanese Journal of Gastroenterology 1997. 94 (2) 148-152.

Izumi, Y., Konishi, H., Mochizuki, T., Ishikura, H., Usui, T., Sawada, H., Miyake, T., and Uchino, H. [A case of acute lymphocytic leukemia complicated with multiple pancreatic pseudocysts probably caused by L-asparaginase]. [Rinsho ketsueki] The Japanese journal of clinical hematology 1984. 25 (1) 57-62.

Jakobovic, Jasminka, Butkovic, Diana, Popovic, Ljiljana, Skaric, Ivancica, and Mikecin, Lili. [Acute childhood pancreatitis caused by valproate]. Lijecnicki vjesnik 2009. 131 Suppl 3:8-10.

Jiang, Y., Yang, X., and Jiang, W. Valproic acid-induced recurrent pancreatitis: one case report. Chinese Journal of Neurology 2017. 50 (1) 52-53.

Kageyama, Y., Yamauchi, H., Nakayama, S., Terashi, K., Hirose, S., Komatsuzaki, O., and Fukuda, H. [A case of bromovalerylurea intoxication associated with acute hepatitis and pancreatitis]. Nihon Naika Gakkai zasshi.The Journal of the Japanese Society of Internal Medicine 1989. 78 (7) 970-971.

Kale, Koroglu B., Songur, Y., Ersoy, I. H., Koroglu, M., Akin, M., and Tamer, M. N. Sitagliptin and acute pancreatitis: Cases and literature review. Turkiye Klinikleri Journal of Medical Sciences 2012. 32 (3) 859-864.

Keino, Dai, Ohyama, Ryo, Ashikaga, Tomoko, Morimoto, Mizuho, Yamashita, Atsuki, Kondoh, Kensuke, and Kinoshita, Akitoshi. [Efficacy of chemotherapy combined with bortezomib for two cases of relapsed/refractory acute lymphoblastic leukemia]. [Rinsho ketsueki] The Japanese journal of clinical hematology 2014. 55 (3) 327-333.

Khristov, Kh, Marinov, D., Vasilev, Kh, and Todorova, M. [Acute steroid pancreatitis in childhood, report of a case]. Khirurgiia 1975. 28 (1) 44-48.

Kim, Da bin, Cho, Yoo Kyung, Song, Hyun Joo, and Song, Byung Cheol. [A case of acute pancreatitis and acute hepatitis caused by ingestion of Ceramium kondoi]. The Korean journal of gastroenterology = Taehan Sohwagi Hakhoe chi 2013. 62 (5) 306-309.

Kim, Kook Hyun, Kim, Tae Nyeun, and Jang, Byung Ik. [A case of acute pancreatitis caused by 5-aminosalicylic acid suppositories in a patient with ulcerative colitis]. The Korean journal of gastroenterology = Taehan Sohwagi Hakhoe chi 2007. 50 (6) 379-383.

Koch, C. A. and Wolkersdoerfer, G. [Hyperlipoproteineumia in HIV patients undergoing antiretroviral therapy: which risk is greater--pancreatitis or coronary disease?]. Deutsche medizinische Wochenschrift (1946) 2000. 125 (47) 1450.

Koch, Robert O., Graziadei, Ivo W., Zangerle, Robert, Romani, Nikolaus, Maier, Hans, and Vogel, Wolfgang. Acute hepatic failure and lactate acidosis associated with antiretroviral treatment for HIV. Wiener klinische Wochenschrift 2003. 115 (3-4) 135-140.

Kohlen, K., Haase, G., Fritzsche, C., and Drewelow, B. [Codeine-induced pancreatitis]. Deutsche medizinische Wochenschrift (1946) 2005. 130 (14) 878-879.

Kojima, K., Kumagai, K., Uchizono, M., Fujii, Y., Koike, T., Kusano, K., Watanabe, H., Murohisa, T., Tamano, M., and Hiraishi, H. A case of mesalazine-induced pancreatitis in intestinal Behcet's disease. Dokkyo Journal of Medical Sciences 2008. 35 (2) 121-125.

Kok, K. F. and de Vries, R. A. [Acute pancreatitis in a hepatitis C positive patient following treatment with peginterferon alfa-2b and ribavirin]. Nederlands tijdschrift voor geneeskunde 2006. 150 (12) 681-683.

Kovacs, E., Tornoczky, J., and Horgasz, J. Pancreatitis after glucocorticoid therapy. Zeitschrift fur die gesamte innere Medizin und ihre Grenzgebiete 1973. 28 (20) 629-633.

Kristensson, H. [Oxyphenbutazone induced pancreatitis--nonspecific glutamyl transpeptidase increase]. Lakartidningen 1982. 79 (39) 3463.

Kvande, K. T. and Madsen, S. [Selective serotonin uptake inhibitors and pancreatitis]. Tidsskrift for den Norske laegeforening: tidsskrift for praktisk medicin, ny raekke 2001. 121 (2) 177-178.

Lambertuccil, J. R., Mourao, Franca B., and De Melo, Queiroz E. Acute pancreatitis caused by meglumine antimoniate given for the treatment of visceral leishmaniasis. Revista da Sociedade Brasileira de Medicina Tropical 2004. 37 (1) 74-75.

Langers, A. M. and Jonkers, G. J. [Pancreatitis ascribed to the use of itraconazole]. Nederlands tijdschrift voor geneeskunde 2001. 145 (23) 1127-1128.

Lankisch, P. G. and Criee, C. P. [Acute pancreatitis during anticonvulsant therapy using sodium valproinate (ergenyl)]. Deutsche medizinische Wochenschrift (1946) 1980. 105 (25) 905.

Larsen, F. S., Clemmesen, J. O., and Hansen, B. A. [Hemorrhagic pancreatitis. A rare complication of paracetamol poisoning]. Ugeskrift for laeger 1995. 157 (7) 898-899.

Luippold, G. Acute pancreatitis acquired by the use of exenatide. Arzneimitteltherapie 2009. 27 (7-8) 248-249.

Luykx, J. J., Huygh, J., Daems, J., and Schoonheydt, K. Pancreatitis and thrombotic thrombocytopenic purpura caused by quetiapine-induced hypertriglyceridemia. Tijdschr Psychiatr 2018. 60 (8) 552-556.

Malbergier, Andre and de Oliveira Junior, Hercilio Pereira. [Sertraline and acute pancreatitis: a case-report]. Revista brasileira de psiquiatria (Sao Paulo, Brazil: 1999) 2004. 26 (1) 39-40.

Matsushita, M., Ebinuma, H., Fukuhara, S., Minami, K., Nakamoto, N., Funakoshi, S., Kanai, T., Saito, H., and Hibi, T. A case of acute pancreatitis induced by telaprevir in the anti-HCV treatment with peginterferon and ribavirin. Acta Hepatologica Japonica 2013. 54 (5) 340-346.

Mattos, M. S., Friedman, R. K., Da Silva Filho, I. L., and Oliveira-Neto, M. P. Pancreatitis: One of the adverse effects of antimonial therapy in leishmaniasis. Anais Brasileiros de Dermatologia 2000. 75 (1) 45-50.

Mikolasevic, I., Milic, S., Mijandrusic-Sincic, B., Licul, V., and Stimac, D. Cannabis-induced acute pancreatitis. Medicinski Glasnik 2013. 10 (2) 405-408.

Miyoshi, T., Ohnishi, A., and Yamaguchi, Y. [A case of motor neuropathy with pyramidal sign due to prolonged administration of high dose of pancuronium bromide (Myoblock)]. Rinsho shinkeigaku = Clinical neurology 1993. 33 (6) 620-624.

Momota, T., Ito, S., Kobayashi, K., Mimasu, S., and Kuniya, T. [A case of acute pancreatitis during administration of valproic acid]. No to hattatsu.Brain and development 1993. 25 (5) 453-458.

Montag, H. Azathioprine and its side effects. H+G Zeitschrift fur Hautkrankheiten 1984. 59 (23) 1600-1607.

Morhart, R. Juvenile systemic lupus erythematosus - A case report. Aktuelle Rheumatologie 2002. 27 (4) 198-200.

Morio, Reona, Imamura, Michio, Fukuhara, Takayuki, Kan, Hiromi, Fujino, Hatsue, Kawaoka, Tomokazu, Hiramatsu, Akira, Aikata, Hiroshi, Sasaki, Tamito, and Chayama, Kazuaki. [A case of chronic hepatitis C with pancreas divisum and acute pancreatitis during combination treatment with telaprevir/peginterferon/ribavirin]. Nihon Shokakibyo Gakkai zasshi = The Japanese journal of gastro-enterology 2014. 111 (10) 1997-2003.

Munhoz, R. P., dos Santos, M. L., and Hernandez-Fustes, O. J. [Fatal necro-hemorrhagic pancreatitis related to sodium valproate: case report]. Arquivos de neuro-psiquiatria 2001. 59 (3-B) 821-823.

Musella, S., Alfano, C., Ciardiello, A., and De' Longis, G. [Rare case of acute pancreatitis following an overdose of cortisone]. Rassegna internazionale di clinica e terapia 1971. 51 (7) 400-407.

Nagata, Kaori, Kihara, Yasuyuki, Eguchi, Ryoji, Nakamura, Hayato, Yoshikawa, Ichiro, and Otsuki, Makoto. [A case of octreotide acetate-induced acute pancreatitis]. Nihon Shokakibyo Gakkai zasshi = The Japanese journal of gastro-enterology 2007. 104 (11) 1652-1657.

Nagayama, A., Kora, Y., Iwaoka, Y., Kusano, T., Matsuo, K., Ozawa, T., and Yasumi, K. Acute necrotizing pancreatitis in a case of Vogt-Koyanagi-Harada disease during sytemic corticosteroid treatment. Japanese Journal of Clinical Ophthalmology 2004. 58 (1) 81-85.

Nishikawa, Jun, Kudo, Takahiko, Miyazaki, Takako, Mihara, Hiroshi, Ueda, Akira, Ando, Takayuki, Kajiura, Shinya, Fujinami, Haruka, Ogawa, Kohei, Hosokawa, Ayumu, and Sugiyama, Toshiro. [Two cases of thiopurine-induced acute pancreatitis in inflammatory bowel disease]. Nihon Shokakibyo Gakkai zasshi = The Japanese journal of gastro-enterology 2012. 109 (2) 224-230.

Okumura, H., Miyahara, K., Ito, K., Koshimura, O., and Hashimoto, K. [Review of literature and a case report of steroid pancreatitis]. Naika.Internal medicine 1969. 23 (5) 915-920.

Ozawa, Y., Inagaki, Y., Yonei, Y., Tsukada, N., Okawa, H., Kiryu, Y., and Oda, M. [An adult case of Reye like syndrome and acute pancreatitis associated with sodium valproate]. Nihon Shokakibyo Gakkai zasshi = The Japanese journal of gastro-enterology 1992. 89 (7) 1467-1470.

Pae, Y., Koyanagi, S., Sawayama, Y., Tani, Y., Hayashi, J., and Kashiwagi, S. [An AIDS case of probable pentamidine-induced diabetic ketoacidosis and severe acute pancreatitis]. Nihon Shokakibyo Gakkai zasshi = The Japanese journal of gastro-enterology 1996. 93 (3) 226-230.

Petersen, H. H. and Skovbjerg, H. [Acute pancreatitis--induced by 5-aminosalicylic acid or an extraintestinal manifestation of ulcerative colitis?]. Ugeskrift for laeger 1995. 157 (39) 5400-5401.

Piotrowska, A., Iwaszkiewicz, K., Sopylo, B., Arlukiewicz-Piwowar, M., Gadomski, A., Brzewski, M., and Kaminski, A. External drainage of pancreatic pseudocyst - Complication induced by L-asparaginase in oncologic pediatric patients. Przeglad Pediatryczny 2007. 37 (1) 148-150.

Piper, C. and Mathias, B. [Oral contraceptives: undesirable effects in internal medicine]. Medizinische Klinik (Munich, Germany: 1983) 1989. 84 (5) 227-235.

Pluhar, W. [A case of possible lovastatin-induced pancreatitis in concomitant Gilbert syndrome]. Wiener klinische Wochenschrift 1989. 101 (16) 551-554.

Rambaldi, M., Russo, R., Iaquinto, G., Agozzino, L., and De Cristofano, R. [Pancreatitis caused by drugs]. Recenti progressi in medicina 1977. 63 (4) 307-328.

Ranft, K. and Binder, H. H. [Recurrent acute pancreatitis in pregnancy and in treatment with ovulation inhibitors]. Medizinische Klinik (Munich, Germany: 1983) 1990. 85 (12) 715-718.

Reece, I. J., Spitz, L., and Rickwood, A. M. K. Steroid induced pancreatitis in a child complicated by abscess formation. Zeitschrift fur Kinderchirurgie 1980. 31 (2) 168-170.

Rodrigues, R., Gavarrete, D. D., Bernardi, E. A. T., and Munhoz, E. C. Pancreatitis in adult with acute lymphoblastic leukemia using L-asparaginase and simvastatin: Case report and systematic review. Medicina (Brazil) 2016. 49 (1) 90-94.

Rossi, A., Palombo, D., Capilupi, V., and Chiapasco, M. Acute pancreatitis induced by administration of acetaminophen plus codeine after a dental treatment: A case report with literature analysis. Dental Cadmos 2016. 84 (5) 304-312.

Sakakihara, Y. Valproate associated pancreatitis. Japanese Journal of Neuropsychopharmacology 1996. 18 (7) 533-536.

Sasaki, Yumihiko, Aoki, Sachiko, Aoki, Kota, Achiwa, Koichi, Yama, Tsuyoki, Kubota, Minoru, Ishikawa, Daisuke, Mizutani, Tetsuya, Kunii, Shin, Watanabe, Kazumasa, and Okumura, Akihiko. [Acute pancreatitis associated with the administration of ceftriaxone in an adult patient]. Nihon Shokakibyo Gakkai zasshi = The Japanese journal of gastro-enterology 2009. 106 (4) 569-575.

Schaefer, J. R., Steinmetz, A., Dugi, K., Ehlenz, K., von Wichert, P., and Kaffarnik, H. [Oral contraceptive-induced pancreatitis in the hyperchylomicronemia syndrome]. Deutsche medizinische Wochenschrift (1946) 1995. 120 (10) 325-328.

Schillaci, F., Stagnitti, F., and Malizia, A. Acute pancreatitis following the use of thiazide diuretic. A clinical contribution. Policlinico - Sezione Chirurgica 1983. 90 (1) 26-31.

Scholten, Joep G. F. and Buijs, Evert J. [Acute pancreatitis after propofol administration]. Nederlands tijdschrift voor geneeskunde 2014. 158:A7115.

Schworer, H. and Ramadori, G. [Acute pancreatitis--adverse effect of 5-aminosalicylic acid (mesalazine) in various galenic dosage forms]. Deutsche medizinische Wochenschrift (1946) 2000. 125 (44) 1328-1330.

Schworer, H. and Ramadori, G. Acute pancreatitis caused by 5-aminosalicylic acid (mesalazine) administered orally or by enema!!. Deutsche Medizinische Wochenschrift 2000. 125 (44) 1328-1330.

Senturk, Z. and Kerman, M. Acute postoperative pancreatitis. Turk Anesteziyoloji ve Reanimasyon 1999. 27 (4) 209-211.

Shimizu, A., Koyama, M., Miyazaki, K., Tagawa, S., Takase, K., Nakano, T., Tameda, Y., and Kosaka, Y. [A case report of drug induced hepatitis and pancreatitis]. Nihon Shokakibyo Gakkai zasshi = The Japanese journal of gastro-enterology 1997. 94 (5) 351-355.

Shin, Do Hyun, Lee, Kwang Hyuk, Kim, Chi Hoon, Kim, Kap Hyun, Park, Sung Hyun, Chang, Dong Kyung, Lee, Jong Kun, and Lee, Kyu Taek. [A case of inferior vena cava thrombosis and acute pancreatitis in a patient with ulcerative colitis]. The Korean journal of gastroenterology = Taehan Sohwagi Hakhoe chi 2010. 56 (4) 255-259.

Sikorska-Fic, Barbara, Stanczak, Elzbieta, Matysiak, Michal, and Kaminski, Andrzej. [Acute pancreatitis during chemotherapy of acute lymphoblastic leukaemia complicated with pseudocyst]. Medycyna wieku rozwojowego 2008. 12 (4 Pt 2) 1051-1055.

Sinkovic, Andreja, Koren, Metka, Skok, Pavel, and Potrc, Stojan. [Acute pancreatitis following azathioprine therapy of crohn's disease--a case report and the overview of the literature]. Acta medica Croatica: casopis Hravatske akademije medicinskih znanosti 2008. 62 (1) 81-84.

Son, Chang Nam, Lee, Hang Lak, Joo, Yong Won, Lee, Oh Young, Han, Dong Soo, Yoon, Byung Chul, Choi, Ho Soon, and Hahm, Joon Soo. [A case of acute pancreatitis induced by multiple drugs in a patient with ulcerative colitis]. The Korean journal of gastroenterology = Taehan Sohwagi Hakhoe chi 2008. 52 (3) 192-195.

Stojanovic, M., Zivanovic, D., Madic, J., Karadzic, D., Stanojevic, G., Jovanovic, M., Jeremic, Lj, and Stojanovic, M. [An acute pancreatitis in a child caused by Na-valproate]. Acta chirurgica Iugoslavica 2004. 51 (3) 125-127.

Svane, S. [Acute pancreatitis after treatment with sulindac]. Tidsskrift for den Norske laegeforening: tidsskrift for praktisk medicin, ny raekke 1992. 112 (11) 1453.

Svane, S. Sulindac-induced acute pancreatitis. Tidsskrift for den Norske Laegeforening 1992. 112 (11) 1453.

Tamura, Shinichi, Ishida, Hiroyuki, Fujiki, Atsushi, Yoshihara, Takao, Kondo, Osamu, Inoue, Masami, Kawa, Keisei, Kawabata, Kenji, and Imamura, Toshihiko. [Effective infliximab treatment for a recurrent type of acute intestinal graft-versus-host disease accompanied by steroid-induced depression]. [Rinsho ketsueki] The Japanese journal of clinical hematology 2012. 53 (3) 361-366.

Tazi, I., Rachid, M., Quessar, A., Harif, M., and Benchekroun, S. [Acute pancreatitis secondary to L-asparaginase (a case report)]. Eastern Mediterranean health journal = La revue de sante de la Mediterranee orientale = al-Majallah al-sihhiyah li-sharq al-mutawassit 2009. 15 (2) 475-479.

Trinchieri, V., Ferone, U., Monacelli, M., Isceri, L., Proietti, F., and Sorice, F. [Pancreatitis during treatment of leishmaniasis with n-methylglucamine antimoniate in a subject infected with HIV]. Rivista europea per le scienze mediche e farmacologiche = European review for medical and pharmacological sciences = Revue europeenne pour les sciences medicales et pharmacologiques 1996. 18 (2) 49-51.

Trinchieri, V., Ferone, U., Monacelli, M., Isceri, L., Proietti, F., and Sorice, F. Pancreatitis during treatment of Leishmaniosis in an HIV infected patient, with N-meglumine antinoniate. European Review for Medical and Pharmacological Sciences 1996. 18 (2) 49-51.

Tsuruoka, Kayori, Sekiya, Syuusuke, Yokoyama, Takeshi, Koitabashi, Kenichirou, Shimazaki, Minako, Sakurada, Tsutomu, Shirai, Sayuri, Yasuda, Takashi, and Kimura, Kenjirou. [MPO-ANCA related vasculitis complicating mucinous cystadenoma of the pancreas and severe acute pancreatitis after steroid pulse therapy: a case report]. Nihon Jinzo Gakkai shi 2008. 50 (7) 948-953.

Tuder, R. M. and Pinto, R. V. [Necro-hemorrhagic pancreatitis due to corticoids]. Revista do Hospital das Clinicas 1982. 37 (3) 152-154.

Tuder, R. M. and Pinto, R. V. Necrohemorrhagic pancreatitis due to corticosteroid therapy. Revista do Hospital das Clinicas de Faculdade de Medicina da Universidade de Sao Paulo 1982. 37 (3) 152-154.

Uccella, R., Morenghi, R., Ripamonti, P., and Depiesse, D. Acute diuretic induced pancreatitis. Rassegna internazionale di clinica e terapia 1982. 62 (13) 913-924.

Uchihara, M., Maekawa, S., Kobayashi, T., Fukuma, T., Sakai, H., Shaura, K., Sato, C., and Marumo, F. [A case of sulindac induced acute pancreatitis]. Nihon Shokakibyo Gakkai zasshi = The Japanese journal of gastro-enterology 1992. 89 (9) 2073-2076.

Uittenbogaart, Steven B. and Klemt-Kropp, Michael. [Mesalazine and sulphasalazine for Crohn's disease: few indications, severe adverse reactions]. Nederlands tijdschrift voor geneeskunde 2011. 155 (48) A3842.

Ujihara, M., Haruta, J.-I., Yamaguchi, T., Ito, H., Ito, T., Furukawa, D., Iwama, A., Uchiyama, I., and Kusakabe, A. A successful drainage with endoscopic cystgastrostomy for pancreatic pseudocyst after L-Asparaginase induced pancreatitis in a child with acute leukemia. Gastroenterological Endoscopy 2007. 49 (3) 358-364.

Urikova, S. Hyperlipidemia induced by quetiapine causing acute pancreatitis. Klinicka Farmakologie a Farmacie 2017. 31 (4) 29-31.

Uskent, N., Sezer, M., and Belen, G. Tamoxifen-induced severe acute pancreatitis after a short-term therapy: A case report. Turk Onkoloji Dergisi 2011. 26 (1) 26-28.

Versteegh, M. I., Vijverberg, P. L., and van Dijk, H. A. [Pancreas irritation caused by oxyphenbutazone (Tanderil)]. Nederlands tijdschrift voor geneeskunde 1983. 127 (42) 1924-1925.

Volz, M. S., Siegmund, B., and Hauser, W. [Efficacy, tolerability, and safety of cannabinoids in gastroenterology: A systematic review]. Schmerz (Berlin, Germany) 2016. 30 (1) 37-46.

von Boxberg, C., Breidenbach, K., Hohler, H., and Kobberling, J. [Undesired drug effects after taking chlormezanone (Muscle Trancopal) with lethal results]. Deutsche medizinische Wochenschrift (1946) 1998. 123 (28-29) 866-870.

Wang, Li xin, Fu, Wei guo, Guo, Da qiao, Xu, Xin, Chen, Bin, Jiang, Jun hao, Yang, Ju, Shi, Zhen yu, Zhu, Ting, Shi, Yun, Dong, Zhi hui, Tang, Xiao, Li, Wei miao, and Wang, Yu qi. [Analysis of contrast medium induced pancreatitis]. Zhonghua yi xue za zhi 2010. 90 (17) 1159-1161.

Wisskirchen, T. and Simon, H. Thyrotoxic crisis under amiodarone. Herz Kreislauf 1995. 27 (3) 98-101.

Wlazlowski, M., Celinska, W., Maciejka-Kapuscinska, L., Ploszynska, A., and Idczak, E. [Acute pancreatitis in children with acute lymphoblastic leukemia treated with L-asparaginase]. Polski tygodnik lekarski (Warsaw, Poland: 1960) 1994. 49 (12-13) 296-297.

Yamano, Takeshi, Yokote, Taiji, Akioka, Toshikazu, Hara, Satoshi, Oka, Tomoko, Tsuji, Motomu, and Hanafusa, Toshiaki. [Acute pancreatitis during the treatment of relapsed acute promyelocytic leukemia with As2O3]. [Rinsho ketsueki] The Japanese journal of clinical hematology 2006. 47 (1) 23-25.

Yamashina, T., Takeuchi, H., Kawanishi, J., Akiyama, S., Niitsu, Y., and Kamata, T. [A case of drug induced pancreatitis with erythrodermia due to administration of phenytoin]. Nihon Shokakibyo Gakkai zasshi = The Japanese journal of gastro-enterology 1991. 88 (5) 1269-1274.

Yang, C. M., Hsieh, Y. L., and Hwang, B. Acute pancreatitis in association with L-asparaginase therapy: report of one case. Zhonghua yi xue za zhi = Chinese medical journal; Free China ed 1993. 51 (1) 74-77.

Yasui, I., Shimokawa, T., Kasai, M., Yamada, H., Watanabe, E., Takeyama, H., and Satake, T. [A case of ALL complicated with acute pancreatitis and pancreatic pseudocyst caused by L-asparaginase ]. Gan to kagaku ryoho.Cancer & chemotherapy 1993. 20 (1) 149-152.

Yutsudo, Y., Hasegawa, Y., Takada, T., Yamada, H., Aoyama, N., Morita, S., and Kasuga, M. [A case of rheumatoid arthritis (RA) with drug-induced acute pancreatitis due to mizoribine]. Ryumachi.[Rheumatism] 1997. 37 (4) 564-567.

Zenker, M., Metzker, M., Wegener, E., and Heidemann, P. H. Valproate (VPA)-induced pancreatitis. Monatsschrift fur Kinderheilkunde 1995. 143 (9) 843-846.

Full text unavailable (n = 18)

Akiyama, T., Fuji, T., Kondoh, S., Adachi, K., Tanaka, S., Harima, K., Sasaki, T., Ohmura, R., Nakata, K., Noguchi, T., Amano, H., Aibe, T., and Takemoto, T. Two cases of drug-induced pancreatitis due to methyldopa and mephenamic acid. Gastroenterological Endoscopy 1988. 30 (4) 760-765.

Andrick, B. J., Capito, M. D., and DeRemer, C. E. Clozapine drug-induced pancreatitis of intermediate latency of onset confirmed by de-challenge and re-challenge. International journal of clinical pharmacology and therapeutics 2019. 57 (1) 37-40.

Basak, R. B., Malpani, V., Kakish, K., Padmanabhan, A., and Boeck, A. Valproic acid-induced pancreatitis in a physically and mentally challenged child. Journal of Pediatric Epilepsy 2012. 1 (1) 69-72.

Bilir, C., Engin, H., Ustun, H., and Ustundag, Y. Gemcitabine-cisplatin induced acute pancreatitis: A case report. HealthMED 2012. 6 (5) 1863-1864.

Bricard, C., Juret, C., Loungouala, M., and Lion, L. Acute pancreatitis caused by valproic acid: A case report. Pharmacien Hospitalier 2000. 35 (142) 29-31.

Butt, Adeel A. Fatal lactic acidosis and pancreatitis associated with ribavirin and didanosine therapy. The AIDS reader 2003. 13 (7) 344-348.

Catalan, I., Supervia, A., Saballs, P., and Blanco, M. L. [Acute pancreatitis due to amphotericin B in an HIV-positive patient]. Anales de medicina interna (Madrid, Spain: 1984) 1999. 16 (1) 47-48.

Chowdhary, M., Kabbani, A. A., and Chhabra, A. Canagliflozin-induced pancreatitis: A rare side effect of a new drug. Therapeutics and Clinical Risk Management 2015. 11:991-994.

De La Torre, Gutierrez S., Montero, Vazquez J., and Bello, Mimbrera G. Repeated pancreatitis and operated cholelithiasis in patients treated lengthily with valproate. Pancreatitis due to valproate?. Anales de Medicina Interna 1985. 2 (5) 240-243.

Kornaros, S. Valproic acid and pancreatitis: Which is the mechanism?. Archives of Gastroenterohepatology 2007. 26 (3-4) 97-99.

Laghate, V. D. and Gupta, S. B. Acute pancreatitis and diabetic ketoacidosis in non-diabetic person while on treatment with sodium valproate, chlorpromazine and haloperidol. The Journal of the Association of Physicians of India 2004. 52:257-258.

Nakamura, C. T., Wilkinson, R., and Woodruff, K. Pancreatitis and parotitis following therapy with L-asparaginase. International Pediatrics 1999. 14 (1) 25-27.

O'Donoghue, D. J. Acute pancreatitis due to nadolol-induced hypertriglyceridaemia. British Journal of Clinical Practice 1989. 43 (2) 74-75.

Parker, W. A. Estrogen-induced pancreatitis. Clinical pharmacy 1983. 2 (1) 75-79.

Schrogie, J. J., Holt, P., Hartley, R. C., and Bartholomew, L. G. "Histamine-induced" pancreatitis. Gastroenterology 1965. 49 (6) 672-675.

Shuster, J. Acute pancreatitis with isotretinoin: Adrenal insufficiency caused by azole antifungal - High-dose quetiapine and photopsia. Levamisole and leukoencephalopathy: Metabolic syndrome and clozapine - Increased ADRs to antimicrobials and anticonvulsants in AIDS patients. Unsuspected trouble with an OTC medication. Hospital Pharmacy 2006. 41 (12) 1152-1154.

Shuster, J. Methemoglobinemia as a result of excessive topical anesthesia; Severe MAOI hypertensive reaction unrecognized by ED personnel; Pulmonary toxicity secondary to procarbazine; Acute pancreatitis associated with propofol; Delirium caused by donepezil fatal aspergillus infection following short-term corticosteroid therapy. Hospital Pharmacy 2002. 37 (6) 589-593.

Yamane, C. Pancreatic necrosis in a boy with acute lymphoblastic leukemia on steroid therapy. Journal of Transportation Medicine 1979. 33 (2) 140-143.

Review of cases from literature (n = 7)

Chapman, S. A., Wacksman, G. P., and Patterson, B. D. Pancreatitis associated with valproic acid: a review of the literature. Pharmacotherapy 2001. 21 (12) 1549-1560.

Franks, Andrea S., Lee, Phillip H., and George, Christa M. Pancreatitis: a potential complication of liraglutide?. The Annals of pharmacotherapy 2012. 46 (11) 1547-1553.

Glueck, C. J., Lang, J., Hamer, T., and Tracy, T. Severe hypertriglyceridemia and pancreatitis when estrogen replacement therapy is given to hypertriglyceridemic women. The Journal of laboratory and clinical medicine 1994. 123 (1) 59-64.

Hauben, Manfred and Hung, Eric Y. Revisiting the reported signal of acute pancreatitis with rasburicase: an object lesson in pharmacovigilance. Therapeutic advances in drug safety 2016. 7 (3) 94-101.

Silva, Matthew A., Key, Sujin, Han, Edward, and Malloy, Michael J. Acute Pancreatitis Associated With Antipsychotic Medication: Evaluation of Clinical Features, Treatment, and Polypharmacy in a Series of Cases. Journal of clinical psychopharmacology 2016. 36 (2) 169-172.

Steinberg, W. M. and Lewis, J. H. Steroid-induced pancreatitis: Does it really exist?. Gastroenterology 1981. 81 (4) 799-808.

Yang, T.-L., Shen, M.-C., Yu, M.-L., Huang, Y.-B., and Chen, C.-Y. Acute pancreatitis in patients with type 2 diabetes mellitus treated with dipeptidyl peptidase-4 inhibitors. Journal of Food and Drug Analysis 2016. 24 (2) 450-454.

Other study design (n = 87)

. A look at rash and pancreatitis as side effects of antidiabetic drugs. Pharmaceutical Journal 2010. 284 (7602) 509-510.

Allen, R. J. and Coulter, D. L. Valproic acid induced pancreatitis in children. Pediatrics 1980. 65 (6) 1194-1195.

Andersen, V., Sonne, J., and Andersen, M. Spontaneous reports on drug-induced pancreatitis in Denmark from 1968 to 1999. European Journal of Clinical Pharmacology 2001. 57 (6-7) 517-521.

Anderson, Sarah L. and Trujillo, Jennifer M. Association of pancreatitis with glucagon-like peptide-1 agonist use. The Annals of pharmacotherapy 2010. 44 (5) 904-909.

Ansari, E., Talenti, D. A., Scopelliti, J. A., Saadat, J. M., and Zehr, B. D. Serum lipase and amylase ratio in acute alcoholic and nonalcoholic pancreatitis by using Dupont ACA discrete clinical analyzer. Digestive diseases and sciences 1996. 41 (9) 1823-1827.

Arriero, J. M. and Gil, J. [Acute pancreatitis, erythromycin and Mycoplasma pneumoniae infection]. Medicina clinica 1991. 96 (10) 394-395.

Badalov, Nison, Baradarian, Robin, Iswara, Kadirawel, Li, Jianjun, Steinberg, William, and Tenner, Scott. Drug-induced acute pancreatitis: an evidence-based review. Clinical gastroenterology and hepatology: the official clinical practice journal of the American Gastroenterological Association 2007. 5 (6) 648-644.

Bai, H. X., Ma, M. H., Orabi, A. I., Park, A., Latif, S. U., Bhandari, V., and Husain, S. Z. Novel characterization of drug-associated pancreatitis in children. Journal of pediatric gastroenterology and nutrition 2011. 53 (4) 423-428.

Barreto, Savio G., Tiong, Leong, and Williams, Randall. Drug-induced acute pancreatitis in a cohort of 328 patients. A single-centre experience from Australia. JOP: Journal of the pancreas 2011. 12 (6) 581-585.

Berend, K. Case 23-2013: A 54-year-old woman with metformin toxicity [7]. New England Journal of Medicine 2013. 369 (18) 1768.

Bolaman, Z., Yavasoglu, I., and Kadikoylu, G. Plasmapheresis in asparaginase-induced pancreatitis. Transfusion 2009. 49 (1) 185.

Bonacini, M. Pancreatic involvement in human immunodeficiency virus infection. Journal of clinical gastroenterology 1991. 13 (1) 58-64.

Buntain, W. L., Wood, J. B., and Woolley, M. M. Pancreatitis in childhood. Journal of pediatric surgery 1978. 13 (2) 143-149.

Busch, S. J., Hoffmann, P., Sahota, P., Johnson, R., Kothny, W., Meyer, F., and Foley, J. E. Studies in rodents with the dipeptidyl peptidase-4 inhibitor vildagliptin to evaluate possible drug-induced pancreatic histological changes that are predictive of pancreatitis and cancer development in man. Diabetes, obesity & metabolism 2013. 15 (1) 72-76.

Chapot, R., Rognon, C., Rudelli, A., and Ducou Le Pointe, H. [Iconographic rubric. A case of acute pancreatitis caused by asparaginase]. Archives Francaises de Pediatrie 1993. 50 (8) 705-707.

Cheng, Roger M. S., Mamdani, Muhammad, Jackevicius, Cynthia A., and Tu, Karen. Association between ACE inhibitors and acute pancreatitis in the elderly. The Annals of pharmacotherapy 2003. 37 (7-8) 994-998.

Cofini, M., Quadrozzi, F., Favoriti, P., Favoriti, M., and Cofini, G. Valproic acid-induced acute pancreatitis in pediatric age: case series and review of literature. Il Giornale di chirurgia 2015. 36 (4) 158-160.

Devlin, John W., Lau, Adah K., and Tanios, Maged A. Propofol-associated hypertriglyceridemia and pancreatitis in the intensive care unit: an analysis of frequency and risk factors. Pharmacotherapy 2005. 25 (10) 1348-1352.

Diemont, W. L. Is this reaction caused by this drug?. The Netherlands journal of medicine 2005. 63 (7) 242-243.

Drucker, D. J., Sherman, S. I., Bergenstal, R. M., and Buse, J. B. The safety of incretin-based therapies - Review of the scientific evidence. Journal of Clinical Endocrinology and Metabolism 2011. 96 (7) 2027-2031.

Drucker, D. J., Sherman, S. I., Gorelick, F. S., Bergenstal, R. M., Sherwin, R. S., and Buse, J. B. Incretin-based therapies for the treatment of type 2 diabetes: Evaluation of the risks and benefits. Diabetes care 2010. 33 (2) 428-433.

Elashoff, Michael, Matveyenko, Aleksey V., Gier, Belinda, Elashoff, Robert, and Butler, Peter C. Pancreatitis, pancreatic, and thyroid cancer with glucagon-like peptide-1-based therapies. Gastroenterology 2011. 141 (1) 150-156.

Engel, S. S., Williams-Herman, D. E., Golm, G. T., Clay, R. J., Machotka, S. V., Kaufman, K. D., and Goldstein, B. J. Sitagliptin: review of preclinical and clinical data regarding incidence of pancreatitis. International journal of clinical practice 2010. 64 (7) 984-990.

Faillie, Jean Luc, Babai, Samy, Crepin, Sabrina, Bres, Virginie, Laroche, Marie Laure, Le Louet, Herve, Petit, Pierre, Montastruc, Jean Louis, Hillaire-Buys, Dominique, and French Pharmacovigilance Center. Pancreatitis associated with the use of GLP-1 analogs and DPP-4 inhibitors: a case/non-case study from the French Pharmacovigilance Database. Acta diabetologica 2014. 51 (3) 491-497.

Filippatos, Theodosios D., Panagiotopoulou, Thalia V., and Elisaf, Moses S. Adverse Effects of GLP-1 Receptor Agonists. The review of diabetic studies: RDS 2014. 11 (3-4) 202-230.

Floyd, Andrea, Pedersen, Lars, Nielsen, Gunnar Lauge, Thorlacius-Ussing, Ole, and Sorensen, Henrik Toft. Risk of acute pancreatitis in users of azathioprine: a population-based case-control study. The American journal of gastroenterology 2003. 98 (6) 1305-1308.

Forsmark, Chris E. Incretins, Diabetes, Pancreatitis and Pancreatic Cancer: What the GI specialist needs to know. Pancreatology: official journal of the International Association of Pancreatology (IAP) ..[et al.] 2016. 16 (1) 10-13.

Friedman, G. D. and Selby, J. V. How often does metronidazole induce pancreatitis?. Gastroenterology 1990. 98 (6) 1702-1703.

Gerstner, Thorsten, Bell, Nellie, and Konig, Stephan. Oral valproic acid for epilepsy--long-term experience in therapy and side effects. Expert opinion on pharmacotherapy 2008. 9 (2) 285-292.

Ghatalia, Pooja, Morgan, Charity J., Choueiri, Toni K., Rocha, Pedro, Naik, Gurudatta, and Sonpavde, Guru. Pancreatitis with vascular endothelial growth factor receptor tyrosine kinase inhibitors. Critical reviews in oncology/hematology 2015. 94 (1) 136-145.

Giorda, C. B., Nada, E., Tartaglino, B., Marafetti, L., and Gnavi, R. A systematic review of acute pancreatitis as an adverse event of type 2 diabetes drugs: from hard facts to a balanced position. Diabetes, obesity & metabolism 2014. 16 (11) 1041-1047.

Giorda, Carlo B., Sacerdote, Carlotta, Nada, Elisa, Marafetti, Lisa, Baldi, Ileana, and Gnavi, Roberto. Incretin-based therapies and acute pancreatitis risk: a systematic review and meta-analysis of observational studies. Endocrine 2015. 48 (2) 461-471.

Gordon, I. J. Acute pancreatitis: A complication of beta-blockade. British medical journal 1982. 284 (6329) 1634.

Guo, Jeff J., Jang, Raymond, Louder, Anthony, and Cluxton, Robert J. Acute pancreatitis associated with different combination therapies in patients infected with human immunodeficiency virus. Pharmacotherapy 2005. 25 (8) 1044-1054.

Haber, C. J., Meltzer, S. J., Present, D. H., and Korelitz, B. I. Nature and course of pancreatitis caused by 6-mercaptopurine in the treatment of inflammatory bowel disease. Gastroenterology 1986. 91 (4) 982-986.

Harris AG, Caroli-Bosc FX, Demarquay JF, Hastier P, Delmont J. Acute pancreatitis in an octreotide-treated AIDS patient - Suggested alternative mechanisms [4]. Pancreas. 1995;11(3):318–9.

Haskell, C. M., Canellos, G. P., and Leventhal, B. G. L aaparaginase toxieity. Cancer research 1969. 29 (4) 974-975.

Heap, G. A., Singh, A., Bewshea, C., Weedon, M. N., Cole, A., Creed, T., Greig, E., Irving, P., Lindsay, J., Mawdsley, J., Mazhar, Z., Orchard, T., Reffitt, D., Holden, A., and Ahmad, T. Thiopurine induced pancreatitis in inflammatory bowel disease: Clinical features and genetic determinants. Gut 2014. 63:A2-A3.

Heap, G. A., Weedon, M. N., Bewshea, C. M., Singh, A., Chen, M., Satchwell, J. B., Vivian, J. P., So, K., Dubois, P. C., Andrews, J. M., Annese, V., Bampton, P., Barnardo, M., Bell, S., Cole, A., Connor, S. J., Creed, T., Cummings, F. R., D'Amato, M., Daneshmend, T. K., Fedorak, R. N., Florin, T. H., Gaya, D. R., Greig, E., Halfvarson, J., Hart, A., Irving, P. M., Jones, G., Karban, A., Lawrance, I. C., Lee, J. C., Lees, C., Lev-Tzion, R., Lindsay, J. O., Mansfield, J., Mawdsley, J., Mazhar, Z., Parkes, M., Parnell, K., Orchard, T. R., Radford-Smith, G., Russell, R. K., Reffitt, D., Satsangi, J., Silverberg, M. S., Sturniolo, G. C., Tremelling, M., Tsianos, E. V., Van Heel, D. A., Walsh, A., Watermeyer, G., Weersma, R. K., Zeissig, S., Rossjohn, J., Holden, A. L., and Ahmad, T. HLA-DQA1-HLA-DRB1 variants confer susceptibility to pancreatitis induced by thiopurine immunosuppressants. Nature Genetics 2014. 46 (10) 1131-1134.

Jensen, Troels M., Saha, Kishore, and Steinberg, William M. Is there a link between liraglutide and pancreatitis? A post hoc review of pooled and patient-level data from completed liraglutide type 2 diabetes clinical trials. Diabetes care 2015. 38 (6) 1058-1066.

Jick, H., Derby, L. E., Garcia Rodriguez, L. A., Jick, S. S., and Dean, A. D. Nonsteroidal antiinflammatory drugs and certain rare, serious adverse events: a cohort study. Pharmacotherapy 1993. 13 (3) 212-217.

Jordan, S. C. and Ament, M. E. Pancreatitis in children and adolescents. The Journal of pediatrics 1977. 91 (2) 211-216.

Kalkan, K. and Aksu, U. Rosuvastatin and risk of acute pancreatitis. International journal of cardiology 2015. 190 (1) 293.

Koller, E. A., Cross, J. T., Doraiswamy, P. M., and Malozowski, S. N. Pancreatitis associated with atypical antipsychotics: From the Food and Drug Administration's MedWatch surveillance system and published reports. Pharmacotherapy 2003. 23 (9 I) 1123-1130.

Ksiadzyna, Dorota. Drug-induced acute pancreatitis related to medications commonly used in gastroenterology. European journal of internal medicine 2011. 22 (1) 20-25.

Lacy, B. E. Emerging treatments in neurogastroenterology: eluxadoline - a new therapeutic option for diarrhea-predominant IBS. Neurogastroenterology and motility: the official journal of the European Gastrointestinal Motility Society 2016. 28 (1) 26-35.

Lambert, J. S., Seidlin, M., Valentine, F. T., Reichman, R. C., and Dolin, R. Didanosine: long-term follow-up of patients in a phase 1 study. Clinical infectious diseases: an official publication of the Infectious Diseases Society of America 1993. 16 Suppl 1:S40-S45.

Lankisch, P. G., Droge, M., and Gottesleben, F. Drug induced acute pancreatitis: incidence and severity. Gut 1995. 37 (4) 565-567.

Lautz, Timothy B., Chin, Anthony C., and Radhakrishnan, Jayant. Acute pancreatitis in children: spectrum of disease and predictors of severity. Journal of pediatric surgery 2011. 46 (6) 1144-1149.

Li, Ling, Shen, Jiantong, Bala, Malgorzata M., Busse, Jason W., Ebrahim, Shanil, Vandvik, Per Olav, Rios, Lorena P., Malaga, German, Wong, Evelyn, Sohani, Zahra, Guyatt, Gordon H., and Sun, Xin. Incretin treatment and risk of pancreatitis in patients with type 2 diabetes mellitus: systematic review and meta-analysis of randomised and non-randomised studies. BMJ (Clinical research ed.) 2014. 348:g2366.

Lieb II, J. G. and Forsmark, C. E. A 50-Year-Old Woman With Unexplained Recurrent Pancreatitis. Clinical Gastroenterology and Hepatology 2009. 7 (2) 141-144.

Ljung, Rickard, Ruck, Christian, Mattsson, Fredrik, Bexelius, Tomas Sjoberg, Lagergren, Jesper, and Lindblad, Mats. Selective serotonin reuptake inhibitors and the risk of acute pancreatitis: a Swedish population-based case-control study. Journal of clinical psychopharmacology 2012. 32 (3) 336-340.

Lucidi, Vincenzina, Alghisi, Federico, Dall'Oglio, Luigi, D'Apice, Maria Rosaria, Monti, Lidia, De Angelis, Paola, Gambardella, Stefano, Angioni, Adriano, and Novelli, Giuseppe. The etiology of acute recurrent pancreatitis in children: a challenge for pediatricians. Pancreas 2011. 40 (4) 517-521.

Mancano, M. ISMP adverse drug reactions-telaprevir-related dermatitis; Neuroleptic malignant syndrome with risperidone long-acting injection; Tigecycline-related pancreatitis; Venlafaxine-related psychosis; Neurologic adverse effects of ranolazine. Hospital Pharmacy 2013. 48 (5) 360-365.

Martinez, Esteban, Milinkovic, Ana, de Lazzari, Elisa, Ravasi, Giovanni, Blanco, Jose L., Larrousse, Maria, Mallolas, Josep, Garcia, Felipe, Miro, Jose M., and Gatell, Jose M. Pancreatic toxic effects associated with co-administration of didanosine and tenofovir in HIV-infected adults. Lancet (London, England) 2004. 364 (9428) 65-67.

Nachnani, J. S., Bulchandani, D. G., Nookala, A., Herndon, B., Molteni, A., Pandya, P., Taylor, R., Quinn, T., Weide, L., and Alba, L. M. Biochemical and histological effects of exendin-4 (exenatide) on the rat pancreas. Diabetologia 2010. 53 (1) 153-159.

Norgaard, M., Ratanajamit, C., Jacobsen, J., Skriver, M. V., Pedersen, L., and Sorensen, H. T. Metronidazole and risk of acute pancreatitis: a population-based case-control study. Alimentary pharmacology & therapeutics 2005. 21 (4) 415-420.

Oliveira, Natalia Mejias, Ferreira, Felipe Augusto Yamauti, Yonamine, Raquel Yumi, and Chehter, Ethel Zimberg. Antiretroviral drugs and acute pancreatitis in HIV/AIDS patients: is there any association? A literature review. Einstein (Sao Paulo, Brazil) 2014. 12 (1) 112-119.

Oskarsson, Viktor, Orsini, Nicola, Sadr-Azodi, Omid, and Wolk, Alicja. Postmenopausal hormone replacement therapy and risk of acute pancreatitis: a prospective cohort study. CMAJ: Canadian Medical Association journal = journal de l'Association medicale canadienne 2014. 186 (5) 338-344.

Pezzilli, R., Morselli-Labate, A. M., and Corinaldesi, R. NSAIDS and acute pancreatitis: A systematic review. Pharmaceuticals 2010. 3 (3) 558-571.

Pozniak, A. L. and Ahern, M. Azathioprine-induced pancreatitis. Arthritis and Rheumatism 1982. 25 (9) 1149.

Preiss, David, Tikkanen, Matti J., Welsh, Paul, Ford, Ian, Lovato, Laura C., Elam, Marshall B., LaRosa, John C., DeMicco, David A., Colhoun, Helen M., Goldenberg, Ilan, Murphy, Michael J., MacDonald, Thomas M., Pedersen, Terje R., Keech, Anthony C., Ridker, Paul M., Kjekshus, John, Sattar, Naveed, and McMurray, John J. V. Lipid-modifying therapies and risk of pancreatitis: a meta-analysis. JAMA 2012. 308 (8) 804-811.

Raja, R. A., Schmiegelow, K., and Frandsen, T. L. Asparaginase-associated pancreatitis in children. British journal of haematology 2012. 159 (1) 18-27.

Raja, Raheel A., Schmiegelow, Kjeld, Albertsen, Birgitte K., Prunsild, Kaie, Zeller, Bernward, Vaitkeviciene, Goda, Abrahamsson, Jonas, Heyman, Mats, Taskinen, Mervi, Harila-Saari, Arja, Kanerva, Jukka, Frandsen, Thomas L., and Nordic Society of Paediatric Haematology and Oncology (NOPHO) group. Asparaginase-associated pancreatitis in children with acute lymphoblastic leukaemia in the NOPHO ALL2008 protocol. British journal of haematology 2014. 165 (1) 126-133.

Romero, Y., Yebra, M., Lacoma, F., and Manzano, L. Metronidazole and pancreatitis. Clinical infectious diseases: an official publication of the Infectious Diseases Society of America 1992. 15 (4) 750-751.

Runzi, M. and Layer, P. Drug-associated pancreatitis: facts and fiction. Pancreas 1996. 13 (1) 100-109.

Rutten, J.-P., Poeze, M., and Dejong, C. H. C. Acute pancreatitis caused by excessive use of growth hormone in a 40-year-old man. Pancreas 2008. 36 (2) 217.

Sahu, S., Saika, S., Pai, S. K., and Advani, S. H. L-asparaginase (Leunase) induced pancreatitis in childhood acute lymphoblastic leukemia. Pediatric hematology and oncology 1998. 15 (6) 533-538.

Seok, Seung Hyeok, Cho, Wan Seob, Park, Jung Shin, Na, Yirang, Jang, Ahram, Kim, Hojoong, Cho, Yujin, Kim, Taesung, You, Ji Ran, Ko, Sanghoon, Kang, Byeong Cheol, Lee, Jong Kwon, Jeong, Jayoung, and Che, Jeong Hwan. Rat pancreatitis produced by 13-week administration of zinc oxide nanoparticles: biopersistence of nanoparticles and possible solutions. Journal of applied toxicology: JAT 2013. 33 (10) 1089-1096.

Singh, Sonal and Loke, Yoon K. Statins and pancreatitis: a systematic review of observational studies and spontaneous case reports. Drug safety 2006. 29 (12) 1123-1132.

Sorensen, H. T., Jacobsen, J., Norgaard, M., Pedersen, L., Johnsen, S. P., and Baron, J. A. Newer cyclo-oxygenase-2 selective inhibitors, other non-steroidal anti-inflammatory drugs and the risk of acute pancreatitis. Alimentary pharmacology & therapeutics 2006. 24 (1) 111-116.

Spigset, Olav, Hagg, Staffan, and Bate, Andrew. Hepatic injury and pancreatitis during treatment with serotonin reuptake inhibitors: data from the World Health Organization (WHO) database of adverse drug reactions. International Clinical Psychopharmacology 2003. 18 (3) 157-161.

Stocco, G., Lanzi, G., Yue, F., Giliani, S., Sasaki, K., Tommasini, A., Pelin, M., Martelossi, S., Ventura, A., and Decorti, G. Patients' induced pluripotent stem cells to model drug induced adverse events: A role in predicting thiopurine induced pancreatitis?. Current Drug Metabolism 2016. 17 (1) 91-98.

Stuecklin-Utsch, A., Hasan, C., Bode, U., and Fleischhack, G. Pancreatic toxicity after liposomal amphotericin B. Mycoses 2002. 45 (5-6) 170-173.

Sung, Hye Young, Kim, Jin Il, Lee, Hyun Jeong, Cho, Hyung Jun, Cheung, Dae Young, Kim, Sung Soo, Cho, Se Hyun, and Kim, Jae Kwang. Acute pancreatitis secondary to ciprofloxacin therapy in patients with infectious colitis. Gut and liver 2014. 8 (3) 265-270.

Thisted, H., Jacobsen, J., Munk, E. M., Norgaard, B., Friis, S., McLaughlin, J. K., Sorensen, H. T., and Johnsen, S. P. Statins and the risk of acute pancreatitis: a population-based case-control study. Alimentary pharmacology & therapeutics 2006. 23 (1) 185-190.

Thoeni, R. F., Fell, S. C., and Goldberg, H. I. CT detection of asymptomatic pancreatitis following ERCP. Gastrointestinal radiology 1990. 15 (4) 291-295.

Tiao, Mao Meng, Chuang, Jiin Haur, Ko, Sheung Fat, Kuo, Hsin Wei, Liang, Chi Di, and Chen, Chao Long. Pancreatitis in children: clinical analysis of 61 cases in southern Taiwan. Chang Gung medical journal 2002. 25 (3) 162-168.

Van Camp, J. M., Polley, T. Z., and Coran, A. G. Pancreatitis in children: Diagnosis and etiology in 57 patients. Pediatric Surgery International 1994. 9 (7) 492-497.

Viazis, Nikos, Rekoumis, George, Vlachogiannakos, John, and Avgerinos, Alec. Effect of octreotide and corticosteroids on human sphincter of oddi motility. Journal of gastroenterology and hepatology 2004. 19 (1) 116-117.

Vilar, Santiago, Harpaz, Rave, Santana, Lourdes, Uriarte, Eugenio, and Friedman, Carol. Enhancing adverse drug event detection in electronic health records using molecular structure similarity: application to pancreatitis. PloS one 2012. 7 (7) e41471.

Vinklerova, Ilona, Prochazka, Michal, Prochazka, Vlastimil, and Urbanek, Karel. Incidence, severity, and etiology of drug-induced acute pancreatitis. Digestive diseases and sciences 2010. 55 (10) 2977-2981.

Vogelzang, Nicholas J. Antiangiogenic agents, chemotherapy, and the treatment of metastatic transitional cell carcinoma. Journal of clinical oncology: official journal of the American Society of Clinical Oncology 2013. 31 (6) 670-675.

Wang, T., Wang, F., Gou, Z., Tang, H., Li, C., Shi, L., and Zhai, S. Using real-world data to evaluate the association of incretin-based therapies with risk of acute pancreatitis: a meta-analysis of 1,324,515 patients from observational studies. Diabetes, obesity & metabolism 2015. 17 (1) 32-41.

Weizman, Z. and Durie, P. R. Acute pancreatitis in childhood. The Journal of pediatrics 1988. 113 (1 Pt 1) 24-29.

Werlin, Steven L. and Fish, Daryl L. The spectrum of valproic acid-associated pancreatitis. Pediatrics 2006. 118 (4) 1660-1663.

Woodall, B. S. and DiGregorio, R. V. Comment: olanzapine-induced acute pancreatitis. The Annals of pharmacotherapy 2001. 35 (4) 506-508.

Non-DIP case report (n = 33)

Aljabri, K., Sirrs, S., and Nantel, S. Hypertriglyceridemia and hypercholesterolemia induced by L-asparaginase. Annals of Saudi Medicine 2003. 23 (3-4) 173-174.

Amouyal, C., Levy, P., Andreelli, F., and Hartemann, A. Acute pancreatitis with dipeptidyl peptidase-4 (DPP4) inhibitor or fulminant type 1 diabetes?. Diabetes & Metabolism 2018. 44 (1) 95-96.

Aurousseau, M. H., Levacher, S., Beneton, C., Blaise, M., and Pourriat, J. L. [Transient dysfibrinogenemia and thrombocytopenia associated with recurrent acute pancreatitis in the course of isotretinoin therapy]. La Revue de medecine interne 1995. 16 (8) 622-625.

Benbow, E. W. Simultaneous acute inflammation in entopic and ectopic pancreas. Journal of clinical pathology 1988. 41 (4) 430-434.

Bhatia, R., Shankar, S. K., and Tandon, P. N. Iatrogenic pancreatitis in a neurosurgical patient. Neurology India 1981. 29 (4) 199-202.

Bhavsar, B. and Bhatt, A. Steroid induced hyperlipidemic pancreatitis and new onset diabetes mellitus. Treatment with plasmapheresis. Journal of the Pancreas 2008. 9 (5) 664-665.

Bodemar, G. and Hjortswang, H. Octreotide-induced pancreatitis: an effect of increased contractility of Oddi sphincter. Lancet (London, England) 1996. 348 (9042) 1668-1669.

Brockner, J. and Boisen, E. Fatal multisystem toxicity after co-trimoxazole. Lancet (London, England) 1978. 1 (8068) 831.

Brown, N. M. and Strachan, J. W. Analgesia in acute pancreatitis. British medical journal 1984. 288 (6434) 1917.

El-Hussuna, Alaa, Arnesen, Regnar Boge, and Rosenberg, Jacob. Tramadol poisoning with hyperamylasemia. BMJ case reports 2010. 2010 .

Epelde, F., Boada, L., and Tost, J. Pancreatitis caused by loperamide overdose. The Annals of pharmacotherapy 1996. 30 (11) 1339.

Fathallah, N., Ben, Salem C., Slim, R., Kaabia, N., Letaief, A., and Bouraoui, K. Fatal allopurinol-induced hypersensitivity syndrome associated with pancreatic abnormalities. Journal of Clinical Rheumatology 2010. 16 (4) 170-171.

Gocho, N., Aoki, E., Okada, C., and Hirashima, T. Myxedema Coma Following the Administration of Gonadotropin-releasing Hormone Agonist Complicated by Acute Pancreatitis. Internal Medicine 11-1-2018. 57 (21) 3117-3122.

Harris, R. E. and Gibbs, C. E. Acute renal failure, acute cholecystitis and pancreatitis. Associated with twin gestation and cesarean section possible implication of tetracycline therapy. Virginia medical monthly 1965. 92 (10) 476-480.

Hiramatsu, K., Moriuchi, J., Arimori, S., Ide, A., Usui, T., Tanaka, K., Sato, T., and Osamura, Y. Acute pancreatitis and Cushing's disease. The Tokai journal of experimental and clinical medicine 1991. 16 (2) 153-156.

Jitkritsadakul, Onanong, Jagota, Priya, Petchrutchatachart, Sitthi, Sansopha, Lalana, Rerknimitr, Rungsun, and Bhidayasiri, Roongroj. Recurrent pancreatitis as a rare complication of duodenal levodopa infusion treatment. Movement disorders: official journal of the Movement Disorder Society 2013. 28 (9) 1308-1310.

Lattanzi, Lorenzo, Casamassima, Francesco, Brunetto, Maurizia, Tatulli, Alessandro, Longobardi, Antonio, Schiavi, Elisa, Danese, Alessandra, Litta, Antonella, Stange, Jonathan P., and Cassano, Giovanni B. Asymptomatic hyperamylasemia and hyperlipasemia associated with aripiprazole. Journal of clinical psychopharmacology 2009. 29 (5) 504-506.

Levy, P., Perniceni, T., and Gayet, B. [High-dose furosemide: a new cause of chronic pancreatitis?]. Gastroenterologie clinique et biologique 1997. 21 (12) 1004-1005.

Marcovici, I. and Marzano, D. Pregnancy-induced hypertension complicated by postpartum renal failure and pancreatitis: A case report. American Journal of Perinatology 2002. 19 (4) 177-179.

Mikhailidis, D., Ganotakis, E., Georgoulias, V., Vallance, D., and Winder, A. Tamoxifen-induced hypertriglyceridaemia. Oncology reports 1997. 4 (3) 625-628.

Ohshiro, Y., Tawata, M., and Takasu, N. Acute pancreatitis and exacerbation of hepatitis B following reduced dose of prednisolone [2]. QJM - Monthly Journal of the Association of Physicians 2003. 96 (11) 868-869.

Paleti, S., Yarlagadda, B., Gremida, A., Aziz, M., Hanson, J., and McCarthy, D. Colitis and Pancreatitis in a Patient with Systemic Lupus Erythematosus: Due to Disease or to Drug?. Digestive diseases and sciences 2018. 63 (9) 2206-2209.

Patel, Jayna S. and Scheiner, Edward D. Acute parotitis induced by trimethoprim/sulfamethoxazole. Ear, nose, & throat journal 2011. 90 (2) E22.

Ragucci, K. R. and Wells, B. J. Olanzapine-induced diabetic ketoacidosis. The Annals of pharmacotherapy 2001. 35 (12) 1556-1558.

Sakaguchi, T., Nakamura, S., Suzuki, S., Konno, H., Fujita, K., Suzuki, K., Ushiyama, T., Ishikawa, A., Harada, M., and Baba, S. Intracystic hemorrhage of pancreatic serous cystadenoma after renal transplantation: report of a case. Surgery today 2000. 30 (7) 667-669.

Selvi Sabater, P., Espuny Miro, A., Munoz Bertran, E. D., and Plaza Aniorte, J. [Possible telaprevir-induced pancreatitis. A case study]. Farmacia hospitalaria: organo oficial de expresion cientifica de la Sociedad Espanola de Farmacia Hospitalaria 2013. 37 (3) 269-270.

Solomon, F. A. J. and Feeney, W. J. Pseudomembranous tracheobronchitis, pancreatitis and enterocolitis: report of a case complicating antibiotic therapy of pneumonia and septicemia. Annals of Internal Medicine 1957. 47 (1) 161-172.

Sommer, M., Dieterich, A., Krause, C., Ruther, E., and Wiltfang, J. Subclinical pancreatitis related to mirtazapine - a case report. Pharmacopsychiatry 2001. 34 (4) 158-159.

Soto-Fernandez, S., Gonzalez-Carro, P., De Pedro-Esteban, A., Legaz-Huidobro, M. L., Perez-Roldan, F., Roncero Garcia-Escribano, O., Valbuena-Gonzalez, M., and Ruiz-Carrillo, F. [Infliximab-induced hepatitis in a patient with Crohn's disease]. Gastroenterologia y hepatologia 2006. 29 (5) 321-322.

Stefaniak, T., Glowacki, J., Dymecki, D., Lachinski, A., and Gruca, Z. Pancreatitis following heart transplantation: report of a case. Surg Today 2003. 33 (9) 693-697.

Tanvetyanon, Tawee and Stiff, Patrick J. Recurrent steroid-responsive pancreatitis associated with myelodysplastic syndrome and transformations. Leukemia & lymphoma 2005. 46 (1) 151-154.

Trancoso, V. N., Ramos, J. S., Barreiras, J., and Reis, B. Chronic pancreatitis after therapy with synthetic ACTH. American Journal of Gastroenterology 1984. 79 (10) 769-772.

Yegin, Ender Gunes, Eryuksel, Emel, Giral, Adnan, Ceyhan, Berrin, and Ozdogan, Osman Cavit. Decision-making dilemma in drug-induced acute pancreatitis. Journal of digestive diseases 2014. 15 (12) 694-697.

Drug name or dosage not reported (n = 171)

. Acute pancreatitis linked to isotretinoin. Prescrire international 2015. 24 (157) 46.

Aboulafia, D. M. Acute pancreatitis. A fatal complication of AIDS therapy. Journal of clinical gastroenterology 1997. 25 (4) 640-645.

Ahmad, Faheem Asem and Mahmud, Sajid. Acute pancreatitis following orlistat therapy: report of two cases. JOP: Journal of the pancreas 2010. 11 (1) 61-63.

Akhter, S., Krishnan, P., and Kaul, P. Tigecycline-Associated Acute Pancreatitis. American journal of therapeutics 2018. 25 (6) e749-e750.

Alabed, Yazan Z., Aghayev, Ayaz, Sakellis, Christopher, and Van den Abbeele, Annick D. Pancreatitis Secondary to Anti-Programmed Death Receptor 1 Immunotherapy Diagnosed by FDG PET/CT. Clinical nuclear medicine 2015. 40 (11) e528-e529.

Alvarez, O. A. and Zimmerman, G. Pegaspargase-induced pancreatitis. Medical and pediatric oncology 2000. 34 (3) 200-205.

Alvarez-Cienfuegos Suarez, J., Ardaiz San Martin, J., Colas Vicente, A., Jorge Cheverri, J., and Inchausti Teja, J. L. [Pancreatitis induced by steroids. Apropos of a case and review of the literature]. Revista Clinica Espanola 1980. 158 (1-2) 81-82.

Alvarez-Cienfuegos, Suarez J., Ardaiz San, Martin J., and Colas, Vicente A. Steroid induced pancreatitis. Revista Clinica Espanola 1980. 158 (1-2) 81-82.

Amellal, N., Amrani, L., Kabbaj, N., Chaoui, Z., and Amrani, N. Acute pancreatitis during ulcerative colitis: Uncommon complication of mesalazine. Journal Africain d'Hepato-Gastroenterologie 2010. 4 (4) 254-256.

Ances, I. G. and McClain, C. A. Acute pancreatitis following the use of thiazide in pregnancy. Southern medical journal 1971. 64 (3) 267-269.

Andersen, G. O. and Ritland, S. Life threatening intoxication with sodium valproate. Journal of toxicology.Clinical toxicology 1995. 33 (3) 279-284.

Aponte-Cipriani, S. L., Teplitz, C., and Yancovitz, S. Pancreatitis possibly related to 2'-3'-dideoxycytidine. Annals of Internal Medicine 1993. 119 (6) 539-540.

Asconape, J. J., Penry, J. K., Dreifuss, F. E., Riela, A., and Mirza, W. Valproate-associated pancreatitis. Epilepsia 1993. 34 (1) 177-183.

Audia, Pat, Feinfeld, Donald A., Dubrow, Alan, and Winchester, James F. Metformin-induced lactic acidosis and acute pancreatitis precipitated by diuretic, celecoxib, and candesartan-associated acute kidney dysfunction. Clinical toxicology (Philadelphia, Pa.) 2008. 46 (2) 164-166.

Begun, J. Positioning biologics-A case-based discussion: Ustekinumab. Journal of gastroenterology and hepatology 2018. 33 Suppl 3:16-17.

Belhassen Garcia, M., Geijo Martinez, F., Carpio Perez, A., Sanchez Martin, F., and Prieto Vicente, V. [Pancreatitis due to acetaminophen-codeine]. Anales de medicina interna (Madrid, Spain: 1984) 2006. 23 (8) 400-401.

Belhassen, Garcia M., Geijo, Martinez F., Carpio, Perez A., Sanchez, Martin F., and Prieto, Vicente, V. Pancreatitis due to acetaminophen-codeine [9]. Anales de Medicina Interna 2006. 23 (8) no.

Bergemann, N., Ehrig, C., Diebold, K., Mundt, C., and von Einsiedel, R. Asymptomatic pancreatitis associated with clozapine. Pharmacopsychiatry 1999. 32 (2) 78-80.

Bisschop, D., Germain, M. L., Munzer, M., and Trenque, T. Thioguanine, pancreatoxicity? [6]. Therapie 2001. 56 (1) 67-69.

Blasco-Perrin, H., Glaser, B., Pienkowski, M., Peron, J. M., and Payen, J. L. Gadolinium induced recurrent acute pancreatitis. Pancreatology: official journal of the International Association of Pancreatology (IAP) ..[et al.] 2013. 13 (1) 88-89.

Boehm, K. M. and Gunaga, S. Cimetidine-induced lactic acidosis and acute pancreatitis. Southern medical journal 2010. 103 (8) 849.

Boudreaux, J. P., Hayes, D. H., Mizrahi, S., Hussey, J., Regenstein, F., and Balart, L. Fulminant hepatic failure, hepatorenal syndrome, and necrotizing pancreatitis after minocycline hepatotoxicity. Transplantation proceedings 1993. 25 (2) 1873.

Bouget, J., Deugnier, Y., Camus, C., Thoreux, P. H., Letulzo, Y., Thomas, R., and Ramee, M. P. [Valproic acid: association of a fatal acute hepatitis and pancreatitis]. Annales de medecine interne 1990. 141 (5) 491-493.

Brazer, S. R. and Medoff, J. R. Sulfonamide-induced pancreatitis. Pancreas 1988. 3 (5) 583-586.

Brown, K. V., Khan, A. Z., and Paterson, I. M. Lisinopril-induced acute pancreatitis. Journal of the Royal Army Medical Corps 2007. 153 (3) 191-192.

Buie, Larry W., Moore, Joseph, and van Deventer, Hank. Successful use of octreotide as a chemoprotectant for prevention of PEG-asparaginase-induced pancreatitis. Pharmacotherapy 2014. 34 (8) e149-e151.

Butt, Waseem, Saadati, Hamid, and Saif, Muhammad Wasif. Oxaliplatin-induced pancreatitis: a case series. Anticancer research 2010. 30 (12) 5113-5115.

Carrillo-Jimenez, R. and Nurnberger, M. Celecoxib-induced acute pancreatitis and hepatitis: A case report [3]. Archives of internal medicine 2000. 160 (4) 553-554.

Charneau, J. and Mendler, M. [Acute necrotic pancreatitis and althiazide]. Gastroenterologie clinique et biologique 1997. 21 (2) 164-165.

Chen, C.-C. and Yeh, S.-P. Fatal pancreatitis occurred in a patient with refractory CD30+ anaplastic large cell lymphoma after brentuximab vedotin treatment. Journal of Cancer Research and Practice 2016.:no.

Chiewchengchol, D., Wananukul, S., and Noppakun, N. Pancreatic panniculitis caused by L-asparaginase induced acute pancreatitis in a child with acute lymphoblastic leukemia. Pediatric Dermatology 2009. 26 (1) 47-49.

Cobb, T. K. and Pierce, J. R. J. Acute pancreatitis associated with ketoprofen. Southern medical journal 1992. 85 (4) 430-431.

Collin, A., Clevenot, D., Moulin, P., Macabeo, C., and David, J.-S. Acute pancreatitis induced by olanzapine. Annales francaises d'anesthesie et de reanimation 2009. 28 (10) 907-909.

Connor, D. F. Severe acute necrotising pancreatitis caused by sodium valproate: a case report. Critical care and resuscitation: journal of the Australasian Academy of Critical Care Medicine 1999. 1 (4) 366-367.

Crawford, Mark W., Pehora, Carolyne, and Lopez, Alejandra V. Drug-induced acute pancreatitis in children receiving chemotherapy for acute leukemia: does propofol increase the risk?. Anesthesia and analgesia 2009. 109 (2) 379-381.

Cuenca, R., Cierco, P., San Jose, A., and Bosch, J. A. [Pyrazolone-induced agranulocytosis, consumption coagulopathy and acute pancreatitis]. Medicina clinica 1989. 92 (15) 596.

Dalal, J. J. and Digrajkar, A. Contrast induced acute pancreatitis following coronary intervention. IHJ Cardiovascular Case Reports (CVCR) 2017. 1 (2) 109-110.

de Tersant, M., Kwon, T., Macher, M. A., Maisin, A., Deschenes, G., and Niel, O. Hypernatremia and acute pancreatitis in chronic kidney disease: back to the salt mines. Questions. Pediatric Nephrology 2018. 33 (7) 1155-1156.

Dickey, Susan E., Mabry, William A., and Hamilton, Leslie A. Possible Sulfamethoxazole/Trimethoprim-Induced Pancreatitis in a Complicated Adolescent Patient Posttraumatic Injury. Journal of pharmacy practice 2015. 28 (4) 419-424.

Dinopoulos, A., Karapanou, O., Alexopoulou, E., Tzetis, M., Attilakos, A., and Fretzayas, A. VPA-induced recurrent pancreatitis in a cystic fibrosis carrier. European Journal of Paediatric Neurology 2011. 15 (5) 453-455.

Durrington, P. N. and Cairns, S. A. Acute pancreatitis: A complication of beta-blockade. British medical journal 1982. 284 (6321) 1016.

Etienne, Denzil and Reda, Yousef. Statins and their role in acute pancreatitis: Case report and literature review. World journal of gastrointestinal pharmacology and therapeutics 2014. 5 (3) 191-195.

Falcao de, Campos C. and de, Carvalho M. Riluzole-induced recurrent pancreatitis. Journal of Clinical Neuroscience 2017. 45:153-154.

Famularo, G., Pozzessere, C., Polchi, S., and De Simone, C. Acute pancreatitis after morphine administration. Italian journal of gastroenterology and hepatology 1999. 31 (6) 522-523.

Farrell, J. and Schmitz, P. G. Paracetamol-induced pancreatitis and fulminant hepatitis in a hemodialysis patient. Clinical nephrology 1997. 48 (2) 132-133.

Flores-Calderon, Judith, Exiga-Gonzalez, Emma, Moran-Villota, Segundo, Martin-Trejo, Jorge, and Yamamoto-Nagano, Alfonso. Acute pancreatitis in children with acute lymphoblastic leukemia treated with L-asparaginase. Journal of pediatric hematology/oncology 2009. 31 (10) 790-793.

Fontana, F. and Cappelli, G. Acute pancreatitis associated with everolimus after kidney transplantation: a case report. BMC Nephrology 2016. 17 (1) 1-4.

Ford, D. M., Portman, R. J., and Lum, G. M. Pancreatitis in children on chronic dialysis treated with valproic acid. Pediatric nephrology (Berlin, Germany) 1990. 4 (3) 259-261.

Franco, J. M., Vallabhajosyula, S., and Griffin, T. J. Quetiapine-induced hypertriglyceridaemia causing acute pancreatitis. BMJ case reports 2015. 2015:no.

Frippiat, F., Derue, G., Heller, F., Honore, P., Moreau, M., and Vandercam, B. Acute pancreatitis associated with severe lactic acidosis in human immunodeficiency virus-infected patients receiving triple therapy. Journal of Antimicrobial Chemotherapy 2000. 45 (3) 411-412.

Frossard, J. L., Felley, C., and Michetti, P. Recurrent acute pancreatitis and therapy for ulcerative colitis. Case reports in gastroenterology 2010. 4 (3) 304-306.

Fujinaga, S., Nishizaki, N., Hirano, D., Kanai, H., Suzuki, M., Ohtomo, Y., Kaneko, K., and Shimizu, T. Acute pancreatitis in a 2-year-old girl on peritoneal dialysis and using icodextrin solution. Clinical nephrology 2011. 75 (1) 89-90.

Gandhi, Mitul D., Evens, Andrew M., Fenske, Timothy S., Hamlin, Paul, Coiffier, Bertrand, Engert, Andreas, Moskowitz, Alison J., Ghosh, Nilanjan, Petrich, Adam M., Lomasney, Jon, Chadburn, Amy, Wood, Gary S., Salva, Katrin, Nardone, Beatrice, Trifilio, Steven M., Raisch, Dennis W., West, Dennis P., Gordon, Leo I., and Winter, Jane N. Pancreatitis in patients treated with brentuximab vedotin: a previously unrecognized serious adverse event. Blood 2014. 123 (18) 2895-2897.

Gershon, T. and Olshaker, J. S. Acute pancreatitis following lisinopril rechallenge. American Journal of Emergency Medicine 1998. 16 (5) 523-524.

Gerstner, Thorsten, Busing, Deike, Bell, Nellie, Longin, Elke, Kasper, Johannes Martin, Klostermann, Wolfgang, Hebing, Burkhard, Hanefeld, Folker, Eckel, Ulrich, Hoffmann, Reiner, Bettendorf, Ulrich, Weidner, Birgit, Wiemer-Kruel, Adelheid, Brockmann, Knut, Neumann, Fritz Wilhelm, Sandrieser, Thorsten, Wolff, Markus, and Konig, Stephan. Valproic acid-induced pancreatitis: 16 new cases and a review of the literature. Journal of gastroenterology 2007. 42 (1) 39-48.

Ghatak, R., Masso, L., Kapadia, D., and Kulairi, Z. I. Medication as a Cause of Acute Pancreatitis. Am J Case Rep 7-28-2017. 18:838-841.

Ghio, Lucio, Fornaro, Gaetano, and Rossi, Paola. Risperidone-induced hyperamylasemia, hyperlipasemia, and neuroleptic malignant syndrome: a case report. Journal of clinical psychopharmacology 2009. 29 (4) 391-392.

Goldstein, J., Laskin, D. A., and Ginsberg, G. H. Sulindac associated with pancreatitis. Annals of Internal Medicine 1980. 93 (1) 151.

Grahit, Vidosa, V, Aviles, Ciguela S., Ribas, Batllori A., and Juncadella, Garcia E. Acute pancreatitis due to lipid-lowering drugs. Atencion primaria 2005. 35 (8) 437-438.

Grandes Ibanez, Jesus, Ocampo Hermida, Antonio, Longueira Suarez, Rebeca, and Lourido Cebreiro, Tamara. [Recurrent pancreatitis in a human immunodeficiency virus-infected patient on antiretroviral therapy]. Medicina clinica 2010. 134 (11) 512.

Gupta, H., Bansal, R., Khanna, S., and Saxena, S. An unusual complication of bortezomib therapy: Acute pancreatitis. Indian Journal of Nephrology 2014. 24 (2) 135-136.

Halalsheh, H., Bazzeh, F., Alkayed, K., Salami, K., and Madanat, F. 6-Mercaptopurine-induced recurrent acute pancreatitis in children with acute lymphoblastic Leukemia/Lymphoma. Journal of pediatric hematology/oncology 2013. 35 (6) 470-472.

Hemphill, Michael T. and Jones, Kellie R. Tigecycline-induced acute pancreatitis in a cystic fibrosis patient: A case report and literature review. Journal of cystic fibrosis: official journal of the European Cystic Fibrosis Society 2016. 15 (1) e9-11.

Hoshino, T., Hatsumi, N., Takada, S., Sakura, T., and Miyawaki, S. All-trans-retinoic acid as a possible cause of acute pancreatitis even in the absence of hypertriglyceridemia. International Journal of Hematology 2008. 88 (1) 121-122.

Howaizi, M., Sbai-Idrissi, M. S., and Baillet, P. [Loperamide-induced acute pancreatitis]. Gastroenterologie clinique et biologique 2000. 24 (5) 589-591.

Isaacs, K. L. and Murphy, D. Pancreatitis after rectal administration of 5-aminosalicylic acid. Journal of clinical gastroenterology 1990. 12 (2) 198-199.

Iyer, Shridhar N., Tanenberg, Robert J., Mendez, Carlos E., West, R. Lee, and Drake, Almond J. Pancreatitis associated with incretin-based therapies. Diabetes care 2013. 36 (4) e49.

Jin, Chuan Fang and Sable, Robert. Isoniazid-induced acute hepatitis and acute pancreatitis in a patient during chemoprophylaxis. Journal of clinical gastroenterology 2002. 35 (1) 100-101.

Jomli, R., Nacef, F., and Douki, S. [Acute pancreatitis induced by valproic acid]. L'Encephale 2013. 39 (4) 292-295.

Kaplan, M. H. and Dreiling, D. A. Steroids revisited. II. Was cortisone responsible for the pancreatitis?. The American journal of gastroenterology 1977. 67 (2) 141-147.

Kawakubo, K., Hata, H., Kawakami, H., Kuwatani, M., Kawahata, S., Kubo, K., Imafuku, K., Kitamura, S., and Sakamoto, N. Pazopanib-induced severe acute pancreatitis. Case Reports in Oncology 2015. 8:356-358.

Kefeli, A., Akturk, A., Yeniova, A. O., and Basyigit, S. Ciprofloxacin induced pancreatitis: Has this condition been overlooked?. Acta gastro-enterologica Belgica 2016. 79 (1) 65-66.

Keskin, M., Songur, Y., and Isler, M. Clomiphene-induced acute pancreatitis without hypertriglyceridemia. American Journal of the Medical Sciences 2007. 333 (3) 194-196.

Keung, Y. K., Rizk, R., Wu, X. Y., and Cobos, E. Drug-induced hypertriglyceridemia with and without pancreatitis. Southern medical journal 1999. 92 (9) 912-914.

Kheda, M. F. and Szerlip, H. M. Two cases of iodixanol-induced pancreatitis. NDT Plus 2008. 1 (5) 296-299.

Kiraly, B. and Gunning, K. A case of pancreatitis associated with aripiprazole in the absence of hyperglycemia. Primary Care Companion to the Journal of Clinical Psychiatry 2008. 10 (6) 484-485.

Kitmacher, P., Zarski, J. P., Barnoud, D., Gressin, R., Guignier, M., Sotto, J. J., and Rachail, M. Acute fatty liver associated necrotical and hemorrhagic pancreatitis following asparaginase therapy. Annales de Gastroenterologie et d'Hepatologie 1991. 27 (6) 271-272.

Klatt, E. C. Pathology of pentamidine-induced pancreatitis. Archives of pathology & laboratory medicine 1992. 116 (2) 162-164.

Kose, M., Emet, S., Akplnar, T. S., Ilhan, M., Gok, A. F. K., Dadashov, M., and Tukek, T. An unexpected result of obesity treatment: Orlistat-related acute pancreatitis. Case reports in gastroenterology 2015. 9:152-155.

Ksouda, K., Maaloul, I., Affes, H., Lahieni, D., Sahnoun, Z., Zeghal, K. M., Ben, Jmeaa M., and Hammami, S. Acute pancreatitis associated with omperazole. Journal Africain d'Hepato-Gastroenterologie 2013. 7 (4) 214-216.

Kumar, V., Issa, D., Smallfield, G., and Bouhaidar, D. Acute pancreatitis secondary to the use of the anabolic steroid trenbolone acetate. Clinical toxicology (Philadelphia, Pa.) 2019. 57 (1) 60-62.

Labgaa, I., Uldry, E., Doerig, C., Schmidt, S., Demartines, N., and Halkic, N. Loperamide-induced recurrent acute pancreatitis. Clinics and research in hepatology and gastroenterology 2016. 40 (1) e13-e14.

Laguna, F., Soriano, V., and Gonzalez-Lahoz, J. M. Misdiagnosis of pancreatitis in patients receiving treatment with pentavalent antimonial agents [7]. Clinical Infectious Diseases 1994. 19 (5) 978-979.

Lai, Hsin Yi, Chen, Jeon Hor, Tsai, Po Pang, Ho, Mao Wang, and Shen, Wu Chung. Hepatic steatosis and pancreatitis associated with the use of stavudine in a patient with HIV infection. AJR.American journal of roentgenology 2004. 183 (6) 1605-1607.

Lal, H., Thakral, A., Suryakant, Naik, S., Munjal, S., Rai, P., Prasad, N., Gupta, A., and Sharma, R. K. Immunosuppression induced acute pancreatitis in renal transplant recipient - Imaging and interventional management. Indian Journal of Transplantation 2013. 7 (4) 120-123.

Lamelas, R. G., Chapchap, P., Magalhaes, A. C., Filho, J. O., Mendes, W. L., and de Camargo, B. Successful management of a child with asparaginase-induced hemorrhagic pancreatitis. Medical and pediatric oncology 1999. 32 (4) 316.

Law, Ryan, Vargo, John J., and Stevens, Tyler. Acute pancreatitis and aspiration pneumonia after administration of synthetic human secretin (with video). Gastrointestinal endoscopy 2011. 74 (5) 1166-1168.

Lazar, D., Rahim, U., and Ramee, E. Ophthalmic erythromycin chronologically linked to acute pancreatitis. Ochsner Journal 2013. 13 (3) 429-430.

Lederman, J. C. and Nawaz, H. Toxic interaction of didanosine and acetaminophen leading to severe hepatitis and pancreatitis: a case report and review of the literature. The American journal of gastroenterology 2001. 96 (12) 3474-3475.

Lee, J. and Goldberg, I. J. Hypertriglyceridemia-induced pancreatitis created by oral estrogen and in vitro fertilization ovulation induction. Journal of Clinical Lipidology 2008. 2 (1) 63-66.

Lee, J. S. O., Owshalimpur, D., and Schofield, C. Trimethoprim/sulfamethoxazole induced multiorgan dysfunction. BMJ case reports 2012.:no.

Leong, R. W. Positioning biological agents: Session two summary. Journal of gastroenterology and hepatology 2018. 33 Suppl 3:11, 2018 Sep. .

Levin, T. L., Berdon, W. E., Seigle, R. R., and Nash, M. A. Valproic-acid-associated pancreatitis and hepatic toxicity in children with endstage renal disease [1]. Pediatric radiology 1997. 27 (2) 192-193.

Lin, Hsuan Hwai, Hsu, Chin Hui, and Chao, You Chen. Tamoxifen-induced severe acute pancreatitis: a case report. Digestive diseases and sciences 2004. 49 (6) 997-999.

Lopez Almaraz, R., Garcia Saiz, Ma M., Montesdeoca Melian, A., and Requena Quesada, G. Ma. [Acute pancreatitis after anesthesia with propofol in a teenage boy treated with liposomal amphotericin B]. Anales de pediatria (Barcelona, Spain: 2003) 2004. 60 (5) 480-481.

Lu, Linghui, Lou, Yanni, and Tan, Huangying. Chemotherapy-induced fulminant acute pancreatitis in pancreatic carcinoma: A case report. Oncology letters 2014. 8 (3) 1143-1146.

Mahjoub, W., Jarboui, S., Moussa, M. B., Abdesselem, M. M., and Zaouche, A. Indomethacin-induced pancreatitis. A second case report [1]. Journal of the Pancreas 2006. 7 (3) 321-323.

Mallick, S. Metformin induced acute pancreatitis precipitated by renal failure. Postgraduate medical journal 2004. 80 (942) 239-240.

Marshall, S. R. Tigecycline-induced pancreatitis. Hospital Pharmacy 2009. 44 (3) 239-241.

Mattioni, Sarah, Zamy, Michele, Mechai, Frederic, Raynaud, Jean Jacques, Chabrol, Amelie, Aflalo, Vanessa, Biour, Michel, and Bouchaud, Olivier. Isoniazid-induced recurrent pancreatitis. JOP: Journal of the pancreas 2012. 13 (3) 314-316.

McIntire, D. R. and Bayne, D. B. Empagliflozin-Induced Pancreatitis. Annals of Pharmacotherapy 2018. 52 (11) 1158-1159.

Mehrotra, T. N., Dwivedi, K. K., Singh, M. M., Mittal, H. S., and Singh, V. S. Hyperlipidaemia and pancreatitis associated with oral contraceptive therapy. The Journal of the Association of Physicians of India 1975. 23 (2) 161-164.

Mehrotra, T. N., Mital, H. S., and Gupta, S. K. Contraceptive pills and acute pancreatitis. The Journal of the Association of Physicians of India 1981. 29 (6) 489-490.

Mete, D., Milon, A., Belon, G., and Gatina, J. H. [Acute pancreatitis and ketoprofen]. Gastroenterologie clinique et biologique 2001. 25 (6-7) 721-722.

Mungall, I. P. and Hague, R. V. Pancreatitis and the pill. Postgraduate medical journal 1975. 51 (602) 855-857.

Mungan, Z., Attila, T., Unal, Kabaoglu Z., and Vural, M. Vildagliptin-induced acute pancreatitis without enzyme elevation. The Turkish journal of gastroenterology: the official journal of Turkish Society of Gastroenterology 2017. 28 (6) 514-517.

Munoz Ruiz, A. I., Calvo Elipe, A., Guerrero Vega, E., Gorgojo Martinez, J. J., Vera Lopez, E., and Gilsanz Fernandez, C. [Pancreatitis and inappropriate ADH secretion syndrome associated with amiodarone]. Anales de medicina interna (Madrid, Spain: 1984) 1996. 13 (3) 125-126.

Murtaza, G., Khalid, M. F., and Mungo, N. A. Recurrent Pantoprazole-Associated Pancreatitis. American journal of therapeutics 2018. 25 (4) e492-e493.

Newman, C. E. and Ellis, D. J. Pancreatitis during combination chemotherapy. Clinical Oncology 1979. 5 (1) 83-84.

Nouri, M. and Martin, D. [Postoperative pancreatitis after non abdominal surgery]. Annales francaises d'anesthesie et de reanimation 2003. 22 (8) 730-732.

Ocal, Serkan, Korkmaz, Murat, Yildirim, Abdullah Emre, Altun, Reskan, Akbas, Enver, and Selcuk, Haldun. Lansoprazole-induced acute pancreatitis. The Turkish journal of gastroenterology: the official journal of Turkish Society of Gastroenterology 2014. 25 (5) 582-583.

Ogunseinde, B. A., Wimmers, E., Washington, B., Iyob, M., Cropper, T., and Callender, C. O. A case of tacrolimus (FK506)-induced pancreatitis and fatality 2 years postcadaveric renal transplant [12]. Transplantation 2003. 76 (2) 448.

O'Halloran, E., Hogan, A., and Mealy, K. Metronidazole-induced pancreatitis. HPB surgery: a world journal of hepatic, pancreatic and biliary surgery 2010. 2010:523468.

Okon, Emmanuel, Engell, Christian, van Manen, Robbert, and Brown, Jack. Tigecycline-related pancreatitis: a review of spontaneous adverse event reports. Pharmacotherapy 2013. 33 (1) 63-68.

Onder, A. H., Ozturk, B., Tatli, A. M., and Kayikcioglu, E. Acute pancreatitis linked to bevacizumab: A case report. Journal of Oncological Science 2018. 4 (2) 108-110.

Ouakaa-Kchaou, A., Gargouri, D., Elloumi, H., Kochlef, A., Romani, M., Kilani, A., Kharrat, J., and Ghorbel, A. Corticosteroid-induced acute pancreatitis. Tunisie Medicale 2010. 88 (2) 124.

Ouakaa-Kchaou, Asma, Gargouri, Dalila, Elloumi, Hela, Kochlef, Asma, Romani, Malika, Kilani, Afef, Kharrat, Jamel, and Ghorbel, Abdeljabbar. [Drug-induced pancreatitis associated with corticosteroids]. La Tunisie medicale 2010. 88 (2) 137.

Oztas, E., Akpinar, M. Y., and Kaplan, M. Ertapenem-induced acute pancreatitis in a cirrhotic patient. Ochsner Journal 2017. 17 (4) 305.

Pandey, Arvind S. and Surana, Ashish. Isoniazid-induced recurrent acute pancreatitis. Tropical doctor 2011. 41 (4) 249-250.

Patel, Mahendra K., Barvaliya, Manish J., Patel, Tejas K., and Tripathi, C. B. Stavudine induced acute necrotizing pancreatitis with tetany in a pediatric patient. Indian journal of pharmacology 2012. 44 (4) 523-525.

Patel, V., Pattisapu, A., Attia, K., and Weiss, J. Nilotinib-Induced Acute Pancreatitis in a Patient with Chronic Myeloid Leukemia. Case Reports in Gastroenterology 2017. 11 (2) 344-347.

Pelucio, M. T., Rothenhaus, T., Smith, M., and Ward, D. J. Fatal pancreatitis as a complication of therapy for HIV infection. The Journal of emergency medicine 1995. 13 (5) 633-637.

Perry, R. C., Cushing, H. E., Deeg, M. A., and Prince, M. J. Ritonavir, triglycerides, and pancreatitis. Clinical infectious diseases: an official publication of the Infectious Diseases Society of America 1999. 28 (1) 161-162.

Perry, W., Jenkins, M. V., and Stamp, T. C. B. Lysosomal enzymes and pancreatitis during rifampicin therapy. Lancet (London, England) 1979. 1 (8114) 492.

Pichon, V., Theissen, O., and Loeb, J. P. [Ketoacidotic coma, a way to discover severe acute pancreatitis in a non-diabetic patient treated with acitretin]. Annales francaises d'anesthesie et de reanimation 1996. 15 (8) 1230-1231.

Pickleman, J., Straus, F. H., and Paloyan, E. Pancreatitis associated with thiazide administration. A role for the parathyroid glands?. Archives of surgery (Chicago, Ill.: 1960) 1979. 114 (9) 1013-1016.

Plotka, A., Wziatek, A., Wachowiak, J., and Derwich, K. Successful Management of a Child With Drug-induced Necrotizing Pancreatitis During Acute Lymphoblastic Leukemia Therapy: A Case Report. Journal of pediatric hematology/oncology 2019. 41 (2) e125-e128.

Queizan, A., Hernandez, F., and Rivas, S. Pancreatic pseudocyst caused by valproic acid: Case report and review of the literature. European Journal of Pediatric Surgery 2003. 13 (1) 60-62.

Rassiat, Emmanuel, Michiels, Christophe, Jouve, Jean Louis, Sgro, Catherine, Faivre, Jean, and Hillon, Patrick. [Acute pancreatitis after clarithromycin and beta-methasone]. Gastroenterologie clinique et biologique 2003. 27 (1) 123.

Rominger, J. M., Gutierrez, J. G., Curtis, D., and Chey, W. Y. Methyldopa-induced pancreatitis. The American journal of digestive diseases 1978. 23 (8) 756-758.

Rosenberg, H. K. and Ortega, W. Hemorrhagic pancreatitis in a young child following valproic acid therapy. Clinical and ultrasonic assessment. Clinical pediatrics 1987. 26 (2) 98-101.

Saadati, H. and Saif, M. W. Sorafenib-induced acute pancreatitis. Journal of the Pancreas 2010. 11 (3) 283-284.

Salem, Jean F., Haydar, Ali, and Hallal, Ali. Inferior phrenic artery pseudoaneurysm complicating drug-induced acute pancreatitis. BMJ case reports 2014. 2014 .

Sammett, D., Greben, C., and Sayeed-Shah, U. Acute pancreatitis caused by penicillin. Digestive diseases and sciences 1998. 43 (8) 1778-1783.

Sarikaya, M., Taser, N., Dogan, Z., Ergu, B., Tezer, F. I., and Filik, L. Levetiracetam induced acute pancreatitis case in pregnancy. Canadian Journal of Neurological Sciences 2013. 40 (6) 896.

Sarner, L. and Fakoya, A. Acute onset lactic acidosis and pancreatitis in the third trimester of pregnancy in HIV-1 positive women taking antiretroviral medication. Sexually transmitted infections 2002. 78 (1) 58-59.

Sastry, J., Young, S., and Shaw, P. J. Acute pancreatitis due to tacrolimus in a case of allogeneic bone marrow transplantation [1]. Bone marrow transplantation 2004. 33 (8) 867-868.

Sato, Munehiro, Takamura, Masaaki, Sato, Yuichi, Yokoyama, Hisashi, Nozawa, Yujiro, Masui, Yukiko, Miida, Hiroshi, Hashimoto, Tsuyoshi, Ito, Masaaki, and Aoyagi, Yutaka. Drug-induced acute pancreatitis associated with 22-oxacalcitriol ointment for treatment of psoriasis. JOP: Journal of the pancreas 2009. 10 (3) 336-337.

Schwartz, M. S. and Cappell, M. S. Pentamidine-associated pancreatitis. Digestive diseases and sciences 1989. 34 (10) 1617-1620.

Sepulveda, S., Dermine, H., Mulot, A., Villard, M., and Haberer, J.-P. Acute pancreatitis after intravenous buprenorphine misuse in a heroin addict [1]. Annales francaises d'anesthesie et de reanimation 2004. 23 (6) 658-659.

Silbermintz, A., Krishnan, S., Banquet, A., and Markowitz, J. Granulomatous pneumonitis, sclerosing cholangitis, and pancreatitis in a child with Crohn disease: Response to infliximab. Journal of pediatric gastroenterology and nutrition 2006. 42 (3) 324-326.

Singh, Veerpal, Devata, Sumana, and Cheng, Yee C. Carboplatin and docetaxel-induced acute pancreatitis: brief report. International journal of clinical oncology 2010. 15 (6) 642-644.

Smoczynski, M., Marek, I., Dubowik, M., Rompa, G., and Pienkowska, J. Endoscopic treatment of pancreatic ascites. Gastroenterologia Polska 2005. 12 (1) 27-30.

Socinski, M. A. and Garnick, M. B. Acute pancreatitis associated with chemotherapy for germ cell tumors in two patients. Annals of Internal Medicine 1988. 108 (4) 567-568.

Solakoglu, Tevfik, Akar, Mustafa, Aktan Kosker, Tugba, Buyukasik, Semnur, and Ersoy, Osman. Is bortezomib a rare cause of acute pancreatitis?. JOP: Journal of the pancreas 2013. 14 (6) 682-683.

Solakoglu, Tevfik, Akyol, Pinar, Guney, Tekin, Dilek, Imdat, Atalay, Roni, Koseoglu, Huseyin, Akin, Ebru, Demirezer Bolat, Aylin, Buyukasik, Naciye Semnur, and Ersoy, Osman. Acute pancreatitis caused by bortezomib. Pancreatology: official journal of the International Association of Pancreatology (IAP) ..[et al.] 2013. 13 (2) 189-190.

Song, J., Kim, S. B., Kim, K. H., Kim, T. N., and Lee, K. H. A case report of motesanib-induced biliary sludge formation causing obstructive cholangitis with acute pancreatitis treated by endoscopic sphincterotomy. Medicine (United States) 2016. 95 (37) no.

Sparrow, M. Positioning biologics-A case-based discussion: Vedolizumab. Journal of gastroenterology and hepatology 2018. 33 Suppl 3:15, 2018 Sep. .

Spraker, Holly L., Spyridis, Georgios P., Pui, Ching Hon, and Howard, Scott C. Conservative management of pancreatic pseudocysts in children with acute lymphoblastic leukemia. Journal of pediatric hematology/oncology 2009. 31 (12) 957-959.

Stepani, P., Mezieres, P., Tossou, H., Delcenserie, R., Andrejak, M., and Bories, C. [Association of methyclothiazide-triamterene and acute pancreatitis: a case with positive reintroduction]. Gastroenterologie clinique et biologique 2000. 24 (10) 974-975.

Tadkal, P. S., Bhat, N., and Hande, M. Benzodiazepine induced acute pancreatitis. Journal of Clinical and Diagnostic Research 2018. 12 (6) OD06-OD07.

Tarik, A. and Mandour, O. Quetiapine induced hyperosmolar non-ketotic state and pancreatitis. Practical Diabetes International 2008. 25 (9) 358.

Tester, W., Forbes, W., and Leighton, J. Vinorelbine-induced pancreatitis: a case report. Journal of the National Cancer Institute 1997. 89 (21) 1631.

Tsai, L.-Y., Ma, W.-Y., Hsia, T.-L., Pei, D., and Su, C.-C. Sitagliptin-associated acute pancreatitis: A case report. Journal of Internal Medicine of Taiwan 2011. 22 (4) 278-282.

Tsao, C. Y. and Wright, F. S. Acute chemical pancreatitis associated with carbamazepine intoxication. Epilepsia 1993. 34 (1) 174-176.

Tsutsumi, Y., Ehira, N., Kanamori, H., Yamato, H., Obara, S., Tanaka, J., Asaka, M., Imamura, M., and Masauzi, N. Pancreatitis complications in a patient with myelodysplastic syndrome, who was treated with fluconazole. International journal of clinical practice 2004. 58 (8) 811.

Umar, J., Zayac, A., Masood, U., and Rawlins, S. Lamivudine-Associated Pancreatitis: Strongest Evidence to Date. American journal of therapeutics 2017. 24 (5) e636-e637.

Ungprasert, Patompong, Permpalung, Nitipong, Summachiwakij, Sarawut, and Manatsathit, Wuttiporn. A case of recurrent acute pancreatitis due to intra-articular corticosteroid injection. JOP: Journal of the pancreas 2014. 15 (2) 208-209.

Urban, M., Splaingard, M., and Werlin, S. L. Pancreatitis associated with remote traumatic brain injury in children. Child's nervous system: ChNS: official journal of the International Society for Pediatric Neurosurgery 1994. 10 (6) 388-391.

Varma, M. R., Mathew, S., Krishnadas, D., and Vinayakumar, K. R. Imatinib-induced pancreatitis. Indian journal of pharmacology 2010. 42 (1) 50-52.

Verma, R. Canagliflozin-associated acute pancreatitis. American journal of therapeutics 2016. 23 (3) e972-e973.

Wachira, John Kelly, Jensen, Cristina Hill, and Rhone, Kelly. Doxycycline-induced pancreatitis: a rare finding. South Dakota medicine: the journal of the South Dakota State Medical Association 2013. 66 (6) 227-229.

Wadood, Ali, Chesner, Robert, Mirza, Mehboob, and Zaman, Shafquat. Tamoxifen precipitation of familial hypertriglyceridaemia: a rare cause of acute pancreatitis. BMJ case reports 2016. 2016 .

Waele, B. D., Smitz, J., and Willems, G. Recurrent pancreatitis secondary to hypercalcemia following vitamin D poisoning. Pancreas 1989. 4 (3) 378-380.

Werlang, M. E., Lewis, M. D., and Bartel, M. J. Tumor necrosis factor alpha inhibitor-induced acute pancreatitis. ACG case reports journal 2017. 4 (18) .

Wingfield, T. W., Goodale, D. B., and Suljaga-Petchel, K. Pancreatitis after propofol administration: Is there a relationship? [1]. Anesthesiology 1996. 84 (1) 236-237.

Wu, Shu Fen, Chen, An Chyi, Peng, Ching Tien, and Wu, Kang Hsi. Octreotide therapy in asparaginase-associated pancreatitis in childhood acute lymphoblastic leukemia. Pediatric blood & cancer 2008. 51 (6) 824-825.

Xingwei, W., Gang, Z., Xiaoyan, Z., Hong, G., Lei, W., Xianlong, L., Lei, D., and Xin, Y. Antitumor drug-induced acute pancreatitis: Report of a special case. Journal of Medical Colleges of PLA 2010. 25 (6) 378-381.

Yang, C. C., Deng, J. F., and Lin, T. J. Pancytopenia, hyperglycemia, shock, coma, rhabdomyolysis, and pancreatitis associated with acetaminophen poisoning. Veterinary and human toxicology 2001. 43 (6) 344-348.

Yilmaz, M., Kinikoglu, O., Ceyla, B., Arslan, F., and Mert, A. Recurrent pancreatitis induced by metronidazole re-exposure and a review of the current literature. Acta gastro-enterologica Belgica 2016. 79 (3) 389-390.

Zerra, Patricia, Bergsagel, John, Keller, Frank G., Lew, Glen, and Pauly, Melinda. Maintenance Treatment With Low-Dose Mercaptopurine in Combination With Allopurinol in Children With Acute Lymphoblastic Leukemia and Mercaptopurine-Induced Pancreatitis. Pediatric blood & cancer 2016. 63 (4) 712-715.

Implicated substance not a drug (n = 32)

Adler, J. B., Mazzotta, S. A., and Barkin, J. S. Pancreatitis caused by measles, mumps, and rubella vaccine. Pancreas 1991. 6 (4) 489-490.

Barquero, Romero J., Redondo Lopez, J. M., Galeano, Diaz F., and Perez, Miranda M. Fatal acute pancreatitis in a patient who received an homeopathic treatment [3]. Medicina clinica 2004. 122 (8) 318-319.

Belze, Olivier Jr, Legras, Annick, Ehrmann, Stephan, Garot, Denis, and Perrotin, Dominique. Cannabis-induced acute pancreatitis. The American journal of emergency medicine 2011. 29 (1) 131-134.

Binet, Q., Dufour, I., Agneessens, E., Debongnie, J. C., Aouattah, T., Covas, A., Coche, J. C., and De, Koninck, X. The second case of a young man with L-arginine-induced acute pancreatitis. Clinical journal of gastroenterology 2018. 11 (5) 424-427.

Bruminhent, J., Carrera, P., Li, Z., Amankona, R., and Roberts, I. M. Acute pancreatitis with saw palmetto use: A case report. Journal of medical case reports 2011. 5:no.

Cerezo-Ruiz, Antonio, Lozano Rodriguez-Mancheno, Aquiles, Cortes-Rodriguez, Begona, and de Paula Rosa-Jimenez, Francisco. [Mild acute pancreatitis associated with cocaine consumption]. Gastroenterologia y hepatologia 2012. 35 (8) 610-611.

Chan, P. W. K. and Goh, A. Y. T. Life threatening pancreatitis following varicella vaccination: Cause, association or co-incidence?. Medical Journal of Malaysia 2000. 55 (4) 527-528.

Das, A., Chang, D., Biankin, A. V., and Merrett, N. D. Pancreatitis following human papillomavirus vaccination. Medical Journal of Australia 2008. 189 (3) 178.

Fatma, Houissa, Mouna, Bouraoui, Leila, Mouelhi, Radhouane, Debbeche, and Taoufik, Najjar. Cannabis: a rare cause of acute pancreatitis. Clinics and research in hepatology and gastroenterology 2013. 37 (1) e24-e25.

Feldman, G. and Zer, M. Infantile acute pancreatitis after mumps vaccination simulating an acute abdomen. Pediatric Surgery International 2000. 16 (7) 488-489.

Findikli, H. A., Bilge, Z., Aydin, H., Yuceer, M. M., Algin, A., and Aydin, B. The combination of acute pancreatitis and toxic hepatitis developing secondary to exposure to malathion: a case report. Acta gastro-enterologica Belgica 2018. 81 (2) 333-335.

Garcia Gavilan, Maria Del Carmen, Moreno Garcia, Antonio Miguel, Rosales Zabal, Jose Miguel, Navarro Jarabo, Jose Maria, and Sanchez Cantos, Andres. Case of drug-induced acute pancreatitis produced by horsetail infusions. Revista espanola de enfermedades digestivas: organo oficial de la Sociedad Espanola de Patologia Digestiva 2017. 109 .

Gatt, D. T. Pancreatitis following monovalent typhoid and cholera vaccinations. British Journal of Clinical Practice 1986. 40 (7) 300-301.

Grant, Paul and Gandhi, Pankaj. A case of cannabis-induced pancreatitis. JOP: Journal of the pancreas 2004. 5 (1) 41-43.

Hayat, Ashik, Thaneeru, Pavan, Priest, Pauline, and Wilson, Robin. Recurrent pancreatitis in an icodextrin-based peritoneal dialysis patient. Yet another case report. The New Zealand medical journal 2013. 126 (1377) 67-69.

Howaizi, Mehran, Chahine, Mouhamad, Haydar, Fadi, Jemaa, Yassine, and Lapoile, Emmanuel. Cannabis-induced recurrent acute pancreatitis. Acta gastro-enterologica Belgica 2012. 75 (4) 446-447.

Jibrin, Ismaila, Erinle, Ayodele, Saidi, Abdulfattah, and Aliyu, Zakari Y. Saw palmetto-induced pancreatitis. Southern medical journal 2006. 99 (6) 611-612.

Kayar, Yusuf, Eroglu, Hatice, Pamukcu, Ozgul, Cetin, Huseyin, Kocas, Onur, and Atci, Mustafa. Cannabinoid-induced acute pancreatitis. The Turkish journal of gastroenterology: the official journal of Turkish Society of Gastroenterology 2014. 25 (3) 335-336.

Lashner, B. A., Kirsner, J. B., and Hanauer, S. B. Acute pancreatitis associated with high-concentration lipid emulsion during total parenteral nutrition therapy for Crohn's disease. Gastroenterology 1986. 90 (4) 1039-1041.

Lesser, D. and Hillesheim, P. Pancreatitis in a woman taking an herbal supplement. Southern medical journal 2007. 100 (1) 59-60.

Lucas Moreno, J. M., Diez, Lorenzo P., Espin, Rios, I, Lopez, Martin A., Gonzalez, Sanchez E., and Contessotto, Spadetto C. Acute pancreatitis after measles, mumps and rubella vaccine. Anales Espanoles de Pediatria 1995. 42 (3) 211-212.

Nicholson, J. A., Smith, D., and Scott, M. H. Nicotine gum causing pancreatitis: A case report. Pancreas 2010. 39 (1) 116.

Pronisceva, V., Sebastian, J., Joseph, S., and Sharp, E. A case report on over-replacement of oral calcium supplements causing acute pancreatitis. Annals of the Royal College of Surgeons of England 2014. 96 (1) 94E-95E.

Ramis, Pedromingo M., Canete, Diaz A., Torres, Rubio J., and Magro, Rodriguez R. Pancreatitis in the young adult after vaccination for measles, mumps and German measles [1]. Atencion primaria 1993. 11 (2) 105.

Rizos, Evangelos, Liberopoulos, Evangelos, Kosta, Paraskevi, Efremidis, Stavros, and Elisaf, Moses. Carbofuran-induced acute pancreatitis. JOP: Journal of the pancreas 2004. 5 (1) 44-47.

Rubinstein, Sofia, Franjul, Rafael, Surana, Sikander, and Fogel, Joshua. Icodextrin-induced acute pancreatitis in a peritoneal dialysis patient: a case report and literature review. Clinical nephrology 2016. 86 (2016) (11) 283-286.

Saka, Mendane, Tuzun, Ahmet, Ates, Yuksel, Bagci, Sait, Karaeren, Necmettin, and Dagalp, Kemal. Acute pancreatitis possibly due to arginine use: a case report. The Turkish journal of gastroenterology: the official journal of Turkish Society of Gastroenterology 2004. 15 (1) 56-58.

Shlomovitz, Eran, Davies, Ward, Cairns, Ewa, Brintnell, William C., Goldszmidt, Mark, and Dresser, George K. Severe necrotizing pancreatitis following combined hepatitis A and B vaccination. CMAJ: Canadian Medical Association journal = journal de l'Association medicale canadienne 2007. 176 (3) 339-342.

Toovey, S. and Jamieson, A. Pancreatitis complicating adult immunisation with a combined mumps measles rubella vaccine. A case report and literature review. Travel Medicine and Infectious Disease 2003. 1 (3) 189-192.

Vazquez-Rodriguez, Sergio, Soto, Santiago, Fernandez, Estela, Baltar, Ruth, and Vazquez-Astray, Enrique. [Cocaine induced acute pancreatitis]. Gastroenterologia y hepatologia 2009. 32 (8) 588-589.

Wargo, K. A., Geveden, B. N., and McConnell, V. J. Cannabinoid-induced pancreatitis: A case series. Journal of the Pancreas 2007. 8 (5) 579-583.

Wargo, Kurt A., Allman, Elena, and Ibrahim, Farrah. A possible case of saw palmetto-induced pancreatitis. Southern medical journal 2010. 103 (7) 683-685.

No latency period reported (n = 36)

Alberti-Flor, J. J., Hernandez, M. E., Ferrer, J. P., Howell, S., and Jeffers, L. Fulminant liver failure and pancreatitis associated with the use of sulfamethoxazole-trimethoprim. The American journal of gastroenterology 1989. 84 (12) 1577-1579.

Barreda, Luis, Rosas, Johana, Milian, William, Valdivia, Duilio, and Targarona, Javier. [Sodium valproate as a cause of acute pancreatitis: a case report]. Revista de gastroenterologia del Peru: organo oficial de la Sociedad de Gastroenterologia del Peru 2006. 26 (3) 318-323.

Camfield, P. R., Bagnell, P., Camfield, C. S., and Tibbles, J. A. Pancreatitis due to valproic acid. Lancet (London, England) 1979. 1 (8127) 1198-1199.

Capolongo, Giovanna, Zacchia, Miriam, Pollastro, Rosa Maria, Radice, Leonardo, and Anastasio, Pietro. A case of valproic acid-induced acute pancreatitis in tuberous sclerosis coexisting with end-stage renal disease. Journal of nephrology 2013. 26 (2) 412-416.

Chase, H. S. J. and Mogan, G. R. Phenformin-associated pancreatitis. Annals of Internal Medicine 1977. 87 (3) 314-315.

Coulter, D. L. and Allen, R. J. Pancreatitis associated with valproic acid therapy for epilepsy. Annals of Neurology 1980. 7 (1) 92.

Dominguez Jimenez, Jose Luis, Bernal Blanco, Enrique, Marin Moreno, Miguel Alonso, and Puente Gutierrez, Juan Jesus. [Acute pancreatitis associated with levofloxacin]. Gastroenterologia y hepatologia 2009. 32 (4) 323-324.

Eckhauser, M. L., Dokler, M., and Imbembo, A. L. Diuretic-associated pancreatitis: a collective review and illustrative cases. The American journal of gastroenterology 1987. 82 (9) 865-870.

Farina Castro, R., Monzon Rubio, E., Ojeda Betancor, N., and Rodriguez-Perez, A. [Necro-hemorrhagic pancreatitis after prolonged propofol perfusion]. Revista espanola de anestesiologia y reanimacion 2002. 49 (10) 558-559.

Fimognari, F. L., Corsonello, A., Pastorell, R., and Antonelli-Inc. Metformin-induced pancreatitis [10]. Diabetes care 2006. 29 (5) 1183.

Fimognari, Filippo Luca, Corsonello, Andrea, Pastorell, Ruggero, and Antonelli-Inc. Metformin-induced pancreatitis: A possible adverse drug effect during acute renal failure. Diabetes care 2006. 29 (5) 1183.

Graeber, G. M., Marmor, B. M., Hendel, R. C., and Gregg, R. O. Pancreatitis and severe metabolic abnormalities due to phenformin therapy. Archives of surgery (Chicago, Ill.: 1960) 1976. 111 (9) 1014-1016.

Graeber, G. M., Marmor, B. M., Hendel, R. C., and Gregg, R. O. Pancreatitis and severe metabolic abnormalities in patients taking phenformin. Review of surgery 1976. 33 (6) 437-439.

Grimaud, J. C., Maillot, A., Bremondy, A., Thervet, L., and Salducci, J. [Must sulfapyridine always be accused? Apropos of a case of acute pancreatitis caused by mesalazine]. Gastroenterologie clinique et biologique 1989. 13 (4) 432.

Hagger, R., Brown, C., and Hurley, P. Olanzapine and pancreatitis. The British journal of psychiatry: the journal of mental science 2000. 177:567.

Haye, O. L. Piroxicam and pancreatitis. Annals of Internal Medicine 1986. 104 (6) 895.

Isley, W. L. and Oki, J. Estrogen-induced pancreatitis after discontinuation of concomitant medroxyprogesterone therapy. American Journal of Medicine 1997. 102 (4) 416-417.

Kahn, D. and Bourgeois, J. A. Acute pancreatitis and diabetic ketoacidosis in a schizophrenic patient taking olanzapine. Journal of clinical psychopharmacology 2007. 27 (4) 397-400.

Khan, B. A., Deel, C., and Hellman, R. N. Tumor lysis syndrome associated with reduced immunosuppression in a lung transplant recipient. Mayo Clinic proceedings 2006. 81 (10) 1397-1399.

Kitamura, S., Yanagi, T., Inamura, Y., Hata, H., Imafuku, K., Yoshino, K., and Shimizu, H. Pazopanib does not bring remarkable improvement in patients with angiosarcoma. Journal of Dermatology 2017. 44 (1) 64-67.

Leblanc, A., Leclercq, B., Nitenberg, G., Lasser, P., Couanet, D., Hartmann, O., and Lemerle, J. [Acute hemorrhagic pancreatitis caused by asparaginase. A case in a child with a favorable course]. Presse medicale (Paris, France: 1983) 1983. 12 (21) 1351-1353.

Levitan, A. A. Phenformin and pancreatitis. Annals of Internal Medicine 1973. 78 (2) 306-307.

Madsen, Kristian Roerbaek. Fatal hypertriglyceridaemia, acute pancreatitis and diabetic ketoacidosis possibly induced by quetiapine. BMJ case reports 2014. 2014 .

Meyers, D. H. Pancreatitis, panniculitis and methandienone. Australian and New Zealand journal of medicine 1982. 12 (3) 302.

Murphy, R. L., Noskin, G. A., and Ehrenpreis, E. D. Acute pancreatitis associated with aerosolized pentamidine. The American journal of medicine 1990. 88 (5N) 53N-56N.

Niederle, B., Bartos, V., Hrodek, O., and Hyniova, H. Acute pancreatitis after imuran in a patient with autoimmune haemolytic anaemia. Materia medica Polona.Polish journal of medicine and pharmacy 1978. 10 (1) 60-62.

Pinkston, R. and Walker, L. A. Multiorgan system failure caused by valproic acid toxicity. The American journal of emergency medicine 1997. 15 (5) 504-506.

Ryan, L. M., Hankwitz, P., and Banasiak, M. Phenformin and pancreatitis. Annals of Internal Medicine 1977. 87 (6) 796.

Sandhya, Lekshmi S., Malavika, S., Sidharthan, N., Kamath, G., and Vijayan, M. A case report on ganciclovir induced pancreatitis. Journal of Pharmaceutical Sciences and Research 2017. 9 (7) 1079-1080.

Singh, S., Nautiyal, A., and Dolan, J. G. Recurrent acute pancreatitis possibly induced by atorvastatin and rosuvastatin. Is statin induced pancreatitis a class effect?. Journal of the Pancreas 2004. 5 (6) 502-504.

Strunge, P. Letter: Frusemide-induced pancreatitis?. British medical journal 1975. 3 (5980) 434.

Stumpf, M. A. M., Kluthcovsky, A. C. G. C., Okamoto, J. M., Schrut, G. C. A., Cajoeiro, P. O., Chacra, A. P. M., and Bizeli, R. Acute pancreatitis secondary to oral contraceptive-induced hypertriglyceridemia: a case report. Gynecological Endocrinology 2018. 34 (11) 930-932.

Stuyt, P. M. J., Demacker, P. N. M., and Stalenhoef, A. F. H. Pancreatitis induced by oestrogen in a patient with type I hyperlipoproteinaemia. British medical journal 1986. 293 (6549) 734.

Suarez, Otero R., Espinoza, Lopez D., Ponce, Ponce A., and Carrillo, Ponce C. Pancreatitis probably induced by tigecycline: A report of a case. Medicina Interna de Mexico 2010. 26 (3) 273-275.

Thys, F., Schapira, M., Ghilain, J. M., Maisin, J. M., and Henrion, J. [Acute pancreatitis and fenfluramine]. Gastroenterologie clinique et biologique 1994. 18 (4) 385-386.

Ting, J. Y. S. Acute pancreatitis related to therapeutic dosing with colchicine: A case report. Journal of medical case reports 2007. 1:no.

Acute pancreatitis diagnosis not according to Atlanta criteria (n = 45)

Arjomand, H. and Kemp, D. G. Quinapril and pancreatitis. The American journal of gastroenterology 1999. 94 (1) 290-291.

Artac, M., Sari, R., Altunbas, H., and Karayalcin, U. Asymptomatic acute pancreatitis due to tamoxifen-induced severe hypertriglyceridemia in a patient with diabetes mellitus and breast cancer. Journal of chemotherapy (Florence, Italy) 2002. 14 (3) 309-311.

Baraibar, I., Quilez, A., Salas, D., Roman, M., Rolfo, C., Perez-Gracia, J. L., and Gil-Bazo, I. Pazopanib-induced asymptomatic radiological acute pancreatitis: A case report. Molecular and Clinical Oncology 2017. 6 (5) 651-654.

Borgia, M. C., Celestini, A., Caravella, P., and Catalano, C. Angiotensin-converting-enzyme inhibitor administration must be monitored for serum amylase and lipase in order to prevent an acute pancreatitis: a case report. Angiology 2001. 52 (9) 645-647.

Brett, A. S. and Shaw, S. V. Simultaneous pancreatitis and hepatitis associated with trimethoprim-sulfamethoxazole. The American journal of gastroenterology 1999. 94 (1) 267-268.

Cabooter, M., Elewaut, A., and Barbier, F. Salicylate-induced pancreatitis. Gastroenterology 1981. 80 (1) 214.

Capurso, G., Archibugi, L., Tessieri, L., Petrone, M. C., Laghi, A., and Arcidiacono, P. G. Focal immune-related pancreatitis occurring after treatment with programmed cell death 1 inhibitors: a distinct form of autoimmune pancreatitis?. European Journal of Cancer 2018. 95:123-126.

Chevalier, X., Awada, H., Baetz, A., and Amor, B. Danazol induced pancreatitis and hepatitis. Clinical rheumatology 1990. 9 (2) 239-241.

Chiba, M., Horie, Y., Ishida, H., Arakawa, H., and Masamune, O. A case of salicylazosulfapyridine (Salazopyrin)-induced acute pancreatitis with positive lymphocyte stimulation test (LST). Gastroenterologia Japonica 1987. 22 (2) 228-233.

Colls, B. M. and George, P. M. Severe hypertriglyceridaemia and hypercholesterolaemia associated with tamoxifen use. Clinical oncology (Royal College of Radiologists (Great Britain)) 1998. 10 (4) 270-271.

Coodley, E., Derasse, J., and Carver, J. Phenformin and pancreatitis. Annals of Internal Medicine 1973. 78 (2) 307-308.

Detlefs, R. L. Drug-induced pancreatitis presenting as subcutaneous fat necrosis. Journal of the American Academy of Dermatology 1985. 13 (2 Pt 1) 305-307.

Durrani, S. R. and Kelly, J. T. Pancreatitis as a complication of aspirin desensitization for aspirin-exacerbated respiratory disease. Journal of Allergy and Clinical Immunology 2013. 131 (1) 244-246.

Elouni, B., Salem, C. B., Zamy, M., Sakhri, J., Bouraoui, K., and Biour, M. Bortezomib-induced acute pancreatitis. Journal of the Pancreas 2010. 11 (3) 275-276.

Frankenburg, F. R. and Kando, J. Eosinophilia, clozapine, and pancreatitis. Lancet (London, England) 1992. 340 (8813) 251.

Fullerton, F., McPhillips, M., Edelman, K., and Riccio, M. Acute pancreatitis in association with clozapine. New Trends in Experimental and Clinical Psychiatry 1994. 10 (3) 149-151.

Garlipp, P., Rosenthal, O., Haltenhof, H., and Machleidt, W. The development of a clinical syndrome of asymptomatic pancreatitis and eosinophilia after treatment with clozapine in schizophrenia: implications for clinical care, recognition and management. Journal of psychopharmacology (Oxford, England) 2002. 16 (4) 399-400.

Gerstner, Thorsten, Bauer, Marc Oliver, Longin, Elke, Bell, Nellie, and Koenig, Stephan A. Reversible hepatotoxicity, pancreatitis, coagulation disorder and simultaneous bone marrow suppression with valproate in a 2-year-old girl. Seizure 2007. 16 (6) 554-556.

Greene, Jeffery P. An adolescent with abdominal pain taking isotretinoin for severe acne. Southern medical journal 2006. 99 (9) 992-994.

Grosse, Pascal, Rusch, Lars, and Schmitz, Bettina. Pancreatitis complicating treatment with intravenous valproic acid. Journal of Neurology 2002. 249 (4) 484-485.

Hoff, P. M., Valero, V., Holmes, F. A., Whealin, H., Hudis, C., and Hortobagyi, G. N. Paclitaxel-induced pancreatitis: a case report. Journal of the National Cancer Institute 1997. 89 (1) 91-93.

Johnson, Cynda Ann. Estrogen-related pancreatis. Menopause (New York, N.Y.) 2003. 10 (5) 488-488.

Kim, Soo Ryang, Imoto, Susumu, Mita, Keiji, Taniguchi, Miyuki, Sasase, Noriko, Muramatsu, Akira, Kudo, Masatoshi, Kitai, Satoshi, El-Shamy, Ahmed, Hotta, Hak, and Hayashi, Yoshitake. Pegylated interferon plus ribavirin combination therapy for chronic hepatitis C with high viral load of serum hepatitis C virus RNA, genotype 1b, discontinued on attaining sustained virological response at week 16 after onset of acute pancreatitis. Digestion 2009. 79 (1) 36-39.

Leseur, A., Locher, C., Kraemer, A., and Bouvet, F. Codeine-acetaminophen combination-induced acute pancreatitis. A case report. JEUR 2006. 19 (1) 37-38.

Liu, J. W. Birth control pills and pancreatitis. Maryland state medical journal 1982. 31 (2) 66-67.

Manfredini, R., Bariani, L., Chierici, F., Tassi, A., Rizzioli, E., Notarstefano, P., Risichella, I. S., Cecilia, O. L., Mirizio, A. M., Franceschini, F., Fersini, C., and Gallerani, M. Acute pancreatitis associated with mesalamine: A case report and review of the literature. Advances in therapy 1996. 13 (4) 216-219.

Media Villa Garcia, J. D., Lopez-Gomez, M., Bianchi Llave, J. L., and Mateas Ruiz, F. [Acute pancreatitis secondary to intravenous administration of erythromycin]. Anales de medicina interna (Madrid, Spain: 1984) 1996. 13 (2) 99-100.

Mileusnic, Darinka, Donoghue, Edmund R., and Lifschultz, Barry D. Pathological case of the month: sudden death in a child as a result of pancreatitis during valproic acid therapy. Pediatric pathology & molecular medicine 2002. 21 (5) 477-484.

Morimoto, T., Hirai, K., Fukumura, A., Takakura, H., Koike, T., and Shimizu, T. Early diagnosis of asparaginase-associated pancreatitis based on elevated serum elastase-1 levels: Case reports. Biomedical Reports 2013. 1 (4) 651-653.

Pascual Velasco, F. [Acute pancreatitis induced by erythromycin]. Medicina clinica 1990. 95 (10) 397.

Pedro-Botet, J., Miralles, R., Coll, J., and Rubies-Prat, J. Captopril versus enalapril: cough versus pancreatitis. DICP: the annals of pharmacotherapy 1990. 24 (4) 438-439.

Possidente, C. J., Rogers, F. B., Osler, T. M., and Smith, T. A. Elevated pancreatic enzymes after extended propofol therapy. Pharmacotherapy 1998. 18 (3) 653-655.

Raderer, M., Kornek, G., and Scheithauer, W. Re: Vinorelbine-induced pancreatitis: a case report. Journal of the National Cancer Institute 1998. 90 (4) 329.

Ramsay, L. E., Wakefield, V. A., and Harris, E. E. Methyldopa-induced chronic pancreatitis. The Practitioner 1982. 226 (1368) 1166-1169.

Ruggiero, S., DI Nardo, R., Polimeno, T., Rossi, F., and Capuano, A. Ceftriaxone-induced pancreatitis in a pediatric patient: case report. Journal of chemotherapy (Florence, Italy) 2010. 22 (1) 63-65.

Schrier, R. W. and Bulger, R. J. Steroid-induced pancreatitis. JAMA 1965. 194 (5) 564-565.

Scotto Di, Fazano C., Messica, O., Quennesson, S., Quennesson, E. R., Inaoui, R., Vergne, P., Bonnet, C., Bertin, P., and Treves, R. Two new cases of glucocorticoid-induced pancreatitis [1]. Revue du Rhumatisme (English Edition) 1999. 66 (4) 235.

Separovic, R., Pavlovic, M., Silovski, T., Silovski, H., and Vuger, A. T. Uncommon side effects of sunitinib therapy in a patient with metastatic renal cell cancer: Case report. Acta clinica Croatica 2018. 57 (3) 577-580.

Shuster, J. Clarifying adverse drug event terminology; thiazolidinedione-induced congestive heart failure; riluzole-induced neutropenia; possible pancreatitis linked to quetiapine use; clopidogrel-induced thrombotic, thrombocytopenic purpura. Hospital Pharmacy 2004. 39 (7) 624.

Soman, M. and Swenson, C. A possible case of carbamazepine-induced pancreatitis. Drug intelligence & clinical pharmacy 1985. 19 (12) 925-927.

Tilkemeier, P. and Thompson, P. D. Acute pancreatitis possibly related to enalapril. The New England journal of medicine 1988. 318 (19) 1275-1276.

Torres, D., Parrinello, G., Trapanese, C., and Licata, G. Sudden severe abdominal pain after a single low dose of paracetamol/codein in a cholecystectomized patient: Learning from a case report. American journal of therapeutics 2010. 17 (4) e133-e134.

Tsutsumi, Yutaka, Kanamori, Hiroe, Mashiko, Shinobu, Yamato, Hiroaki, Ibata, Makoto, Ehira, Nobuyuki, Kawamura, Takahito, Umehara, Shintaro, Obara, Shinji, Mori, Akio, Ogura, Nobutaka, Tanaka, Junji, Asaka, Masahiro, Imamura, Masahiro, and Masauzi, Nobuo. Leukoencephalopathy with cerebral hemorrhage following acute pancreatitis due to tacrolimus in a case of allogeneic peripheral blood stem cell transplantation. Leukemia & lymphoma 2006. 47 (5) 943-947.

Warren, S. E., Mitas II, J. A., and Swerdlin, A. H. R. Pancreatitis due to methyldopa: Case report. Military Medicine 1980. 145 (6) 399-400.

Zylberberg, H., Zylberberg, L., Hagege, H., Havard, S., Gorin, I., and Chousterman, M. Probable G-CSF-induced hepatitis and pancreatitis in an HIV-seropositive patient. Journal of hepatology 1995. 22 (5) 596-597.

Acute pancreatitis diagnostic criteria not reported (n = 5)

Famularo, G., Morviducci, L., and Gasbarrone, L. Pancreatitis in incretin-based therapies. Clinics and research in hepatology and gastroenterology 2015. 39 (3) e37-e38.

Gardillou, L., Cvitkovic, F., Floiras, J.-L., Briere, M., and Turpin, F. Acute pancreatitis following cytotoxic therapy based of doxorubicin and vinorelbin: About a case report. Journal de Pharmacie Clinique 1999. 18 (4) 292-294.

Llorente, S., Gimeno, L., Navarro, M. J., Moreno, S., and Rodriguez-Girones, M. Therapy of visceral leishmaniasis in renal transplant recipients intolerant to pentavalent antimonials. Transplantation 2000. 70 (5) 800-801.

Naguy, A. and Elsori, D. Quetiapine-induced pancreatitis in a case of juvenile bipolar disorder. Asia Pac Psychiatry 2018. 10 (3) e12316, 2018.

Weaver, G. A. Do antihypertensive agents cause chronic pancreatitis?. Journal of clinical gastroenterology 1987. 9 (1) 8-11.

Excluded for other reasons (n = 81)

All but one of these exclusions were due to DIP being attributed to or suspected of being caused by a combination of drugs, or the authors being unable to isolate the causative drug amongst many that the patient was taking. The remaining case was excluded because the patient had an acute systemic syndrome of which acute pancreatitis was one manifestation (i.e., Drug Rash with Eosinophilia and Systemic Symptoms (DRESS) syndrome), and the AP may have been secondary to systemic dysfunction.

Abou Chacra, L., Ghosn, M., Ghayad, E., and Honein, K. A case of pancreatitis associated with all-trans-retinoic acid therapy in acute promyelocytic leukemia [2]. Hematology Journal 2001. 2 (6) 406-407.

Abraham, M., Mitchell, J., Simsovits, D., and Gasperino, J. Hypertriglyceridemic Pancreatitis Caused by the Oral Contraceptive Agent Estrostep. Journal of Intensive Care Medicine 2015. 30 (5) 303-307.

Alajaj, A. and Elrishi, M. A. Acute pancreatitis associated with saxagliptin treatment presented by metabolic acidosis. Practical Diabetes 2016. 33 (5) 158-158a.

Alonso-Alonso, M., Rodriguez Prada, J. Ignacio, and Gomez-Alonso, J. [Pancreatitis coincident with valproate and olanzapine use]. Neurologia (Barcelona, Spain) 2006. 21 (1) 47-48.

Ando, Kenji, Kim, Soo Ryang, Imoto, Susumu, Nakajima, Taisuke, Mita, Keiji, Fukuda, Katsumi, Taniguchi, Miyuki, Sasase, Noriko, Muramatsu, Akira, Matsuoka, Toshiyuki, Kudo, Masatoshi, and Hayashi, Yoshitake. Acute Pancreatitis Associated with Pegylated Interferon and Ribavirin Treatment of Chronic Hepatitis C, Genotype 1b with High Viral Load. Case reports in gastroenterology 2009. 3 (3) 372-376.

Antonopoulos, Stavros, Mikros, Sotiris, Kokkoris, Stelios, Protopsaltis, John, Filioti, Konstantina, Karamanolis, Dimitrios, and Giannoulis, Grigorios. A case of acute pancreatitis possibly associated with combined salicylate and simvastatin treatment. JOP: Journal of the pancreas 2005. 6 (3) 264-268.

Batson, O. A. and Branda, R. F. Acute pancreatitis complicating therapy of Hodgkin disease: a case report. American Journal of Hematology 1990. 33 (1) 78-79.

Beamish, N., Schwarer, A. P., Watson, A. M., Roberts, S., O'Brien, P., and Morgan, B. Acute pancreatitis complicating a bone marrow harvest. Bone marrow transplantation 1997. 19 (5) 525-526.

Bedrossian, S. and Vahid, B. A case of fatal necrotizing pancreatitis: Complication of hydrochlorothiazide and lisinopril therapy. Digestive diseases and sciences 2007. 52 (2) 558-560.

Ben Kridis, W., Khanfir, A., and Frikha, M. Acute pancreatitis induced by anticancer chemotherapy. Acta clinica Belgica 2013. 68 (4) 309-310.

Blanchard, Jennifer N., Wohlfeiler, Michael, Canas, Albert, King, Kevin, and Lonergan, J. Tyler. Pancreatitis with didanosine and tenofovir disoproxil fumarate [corrected]. Clinical infectious diseases: an official publication of the Infectious Diseases Society of America 2003. 37 (5) e57-e62.

Bracamonte, Jesse D., Underhill, Mike, and Sarmiento, Paul. Acute pancreatitis associated with lisinopril and olanzapine. American journal of health-system pharmacy: AJHP: official journal of the American Society of Health-System Pharmacists 2010. 67 (3) 214-216.

Bui, S. K., O'brien, J. M., and Cunningham Jr, E. T. Purtscher retinopathy following drug-induced pancreatitis in an HIV-positive patient. Retina 2001. 21 (5) 542-545.

Callens, S., De, Schacht C., Huyst, V., and Colebunders, R. Pancreatitis in an HIV-infected person on a tenofovir, didanosine and stavudine containing highly active antiretroviral treatment. Journal of Infection 2003. 47 (2) 188-189.

Cao, C. L., Duan, P. Y., Zhang, W. J., Li, L., Qu, F. Z., Sun, B., and Wang, G. Acute pancreatitis induced by etoposide-lobaplatin combination chemotherapy used for the treatment of lung cancer: A case report and literature review. Medicine (United States) 2017. 96 (29) e7601, 2017.

Casassus-Builhe, Didier, Rey, Phillippe, and Carrere, Christophe. [Association of paracetamol and codeine, a rare cause of acute drug-induced pancreatitis]. Presse medicale (Paris, France: 1983) 2004. 33 (8) 536.

Ceneli, O., Sucak, G. T., Yagci, M., Karakan, T., Isik, S., and Haznedar, R. Acute pancreatitis complicating treatment of granulocytic sarcoma; cytosine arabinoside induced? A case report. HAEMA 2005. 8 (2) 297-300.

Chapman, Scott J. R., Woolley, Ian J., Visvanathan, Kumar, and Korman, Tony M. Acute pancreatitis caused by tipranavir/ritonavir-induced hypertriglyceridaemia. AIDS (London, England) 2007. 21 (4) 532-533.

Chapoy, P., Laplane, D., Monfort, G., Alessandrini, P., and Carcassonne, M. [Acute non-traumatic pancreatitis in childhood. Report of 9 cases (author's transl)]. Chirurgie pediatrique 1980. 21 (5) 313-320.

Chaudhari, Swati, Park, James, Anand, Bhupinderjit S., Pimstone, Neville R., Dieterich, Douglas T., Batash, Steven, and Bini, Edmund J. Acute pancreatitis associated with interferon and ribavirin therapy in patients with chronic hepatitis C. Digestive diseases and sciences 2004. 49 (6) 1000-1006.

Chou, Jen Wei, Cheng, Ken Sheng, and Huang, Chih Wen. Sorafenib-induced Acute Pancreatitis: A Case Report and Review of the Literature. Internal medicine (Tokyo, Japan) 2016. 55 (6) 623-627.

Colle, I., Peeters, P., Reynaert, H., and Urbain, D. Enalapril/hydrochlorothiazide-induced angioneurotic edema and acute necrotizing pancreatitis. European journal of internal medicine 2000. 11 (1) 45-47.

da Silva, Jane, Giroldi, Simone Batisti, de Oliveira Basso, Franciani, Antunes, Georgia Nunes, Borba, Luis Augusto, and de Lima, Cassio Renato Montenegro. Acute pancreatitis during interferon-alpha and ribavirin treatment for hepatitis C. BMJ case reports 2009. 2009 .

Fisher, A. A. and Bassett, M. L. Acute pancreatitis associated with angiotensin II receptor antagonists. Annals of Pharmacotherapy 2002. 36 (12) 1883-1886.

Garg, R., Hussey, C., and Ibrahim, S. Pancreatitis associated with the use of sitagliptin and orlistat combination: A case report. Diabetic Medicine 2010. 27 (4) 485-486.

Gropper, Debra and Jackson, Cherry W. Pancreatitis associated with quetiapine use. Journal of clinical psychopharmacology 2004. 24 (3) 343-345.

Gupta, A., Marrs, T., Urquhart, D., Clarke, S., Rosenthal, M., and Bush, A. Fungal pleural effusion secondary to a rare cause of pancreatic pseudocyst. Pediatric Pulmonology 2009. 44 (6) 616-618.

Hallberg, P., Hallberg, E., and Amini, H. Acute pancreatitis following medical abortion: Case report. BMC Women's Health 2004. 4:no.

Hamrahian, M., Fulop, T., Mollaee, M., Lopez-Ruiz, A., and Juncos, L. A. Recurrent acute pancreatitis in a patient on peritoneal dialysis using 7.5% icodextrin. Peritoneal dialysis international: journal of the International Society for Peritoneal Dialysis 2012. 32 (5) 568-570.

Hastier, P., Demarquay, J. F., Maes, B., Caroli-Bosc, F. X., Dumas, R., Delmont, J., and Chichmanian, R. M. Acute pancreatitis induced by codeine-acetaminophen association: a case report with positive rechallenge. Pancreas 1996. 13 (3) 324-326.

Hatzipantelis, E., Pana, Z. D., Pavlou, E., Balakou, E., Tsotoulidou, V., Papageorgiou, T., Tragiannidis, A., and Athanassiadou, F. Epileptic seizures after octreotide administration in a 6.5-year-old female with ALL and L-asparaginase associated pancreatitis: a possible drug interaction. Klinische Padiatrie 2011. 223 (6) 360-363.

Hsieh, Cheng Yang and Chen, Chih Hung. Rhabdomyolysis and pancreatitis associated with coadministration of danazol 600 mg/d and lovastatin 40 mg/d. Clinical therapeutics 2008. 30 (7) 1330-1335.

Iyer, Shridhar N., Drake, Almond J., West, R. Lee, Mendez, Carlos E., and Tanenberg, Robert J. Case report of acute necrotizing pancreatitis associated with combination treatment of sitagliptin and exenatide. Endocrine practice: official journal of the American College of Endocrinology and the American Association of Clinical Endocrinologists 2012. 18 (1) e10-e13.

Jadresic, D. Acute pancreatitis associated with dual vigabatrin and lamotrigine therapy. Seizure 1994. 3 (4) 319.

Jain, R. and Ramanan, S. V. Iatrogenic pancreatitis. A fatal complication in the induction therapy for acute lymphocytic leukemia. Archives of internal medicine 1978. 138 (11) 1726.

Jeffries, J. J. and Masson, J. Pancreatitis following overdose with amoxapine and procyclidine. Canadian journal of psychiatry.Revue canadienne de psychiatrie 1985. 30 (7) 546-547.

Jones, Kellie L. and Valero, Vicente. Capecitabine-induced pancreatitis. Pharmacotherapy 2003. 23 (8) 1076-1078.

Jost, R., Stey, C., and Salomon, F. Fatal drug-induced pancreatitis in HIV. Lancet (London, England) 1993. 341 (8857) 1412.

Kabbaj, N., Sentissi, S., Guedira, M. M., Mohammadi, M., Benaissa, A., and Amrani, N. [Acute pancreatitis during treatment for chronic viral hepatitis C]. Gastroenterologie clinique et biologique 2008. 32 (3) 232-233.

Kanbay, Mehmet, Sekuk, Haldun, Yilmaz, Ugur, Gur, Gurden, and Boyacioglu, Sedat. Acute pancreatitis associated with combined lisinopril and atorvastatin therapy. Digestive diseases (Basel, Switzerland) 2005. 23 (1) 92-94.

Kirian, Margaret A., Higginson, Robert T., and Fulco, Patricia Pecora. Acute onset of pancreatitis with concomitant use of tenofovir and didanosine. The Annals of pharmacotherapy 2004. 38 (10) 1660-1663.

Kolk, A., Horneff, G., Wilgenbus, K. K., Wahn, V., and Gerharz, C. D. Acute lethal necrotising pancreatitis in childhood systemic lupus erythematosus - Possible toxicity of immunosuppressive therapy. Clinical and experimental rheumatology 1995. 13 (3) 399-403.

Kunjathaya, Purnima, Ramaswami, Pradeep Kakkadasam, Krishnamurthy, Anupama Nagar, and Bhat, Naresh. Acute necrotizing pancreatitis associated with vildagliptin. JOP: Journal of the pancreas 2013. 14 (1) 81-84.

Liu, B. A., Knowles, S. R., Cohen, L. B., Werb, M. R., and Shear, N. H. Pancreatic insufficiency due to antituberculous therapy. The Annals of pharmacotherapy 1997. 31 (6) 724-726.

Locher, Christophe, Lambare, Benedicte, Fischer, Daniel, and Labayle, Denis. [Acute pancreatitis induced by codeine-acetaminophen association: report of two cases]. Gastroenterologie clinique et biologique 2003. 27 (1) 124-125.

Longhurst, H. J. and Pinching, A. J. Drug Points: pancreatitis associated with hydroxyurea in combination with didanosine. BMJ (Clinical research ed.) 2001. 322 (7278) 81.

Mascarello, M., Papa, G., Arne, Z. M., and Luzzati, R. Acute necrotizing pancreatitis related to tigecycline. Journal of Antimicrobial Chemotherapy 2012. 67 (5) 1296-1297.

McDonald, Kevin B., Garber, Bryan G., and Perreault, Marc M. Pancreatitis associated with simvastatin plus fenofibrate. The Annals of pharmacotherapy 2002. 36 (2) 275-279.

Mennecier, D., Thiolet, C., Bredin, C., Potier, V., Vergeau, B., and Farret, O. [Acute pancreatitis after treatment by levofloxacin and methylprednisolone]. Gastroenterologie clinique et biologique 2001. 25 (10) 921-922.

Mills, K. M., Johnson, D. M., Middlebrooks, M., and Burton, G. V. Possible drug-associated pancreatitis after paclitaxel-cremophor administration. Pharmacotherapy 2000. 20 (1) 95-97.

Miltiadous, G., Anthopoulou, A., and Elisaf, M. Acute pancreatitis possibly associated with combined salicylate and atorvastatin therapy. Journal of the Pancreas 2003. 4 (1) 20-21.

Ocal, Serkan, Selcuk, Haldun, Korkmaz, Murat, Unal, Hakan, and Yilmaz, Ugur. Acute pancreatitis following doxycycline and ornidazole coadministration. JOP: Journal of the pancreas 2010. 11 (6) 614-616.

Okayasu, Hiroaki, Shinozaki, Takahiro, Osone, Akira, Ozeki, Yuji, and Shimoda, Kazutaka. Development of acute pancreatitis caused by sodium valproate in a patient with bipolar disorder on hemodialysis for chronic renal failure: a case report. BMC psychiatry 2014. 14:93.

Palacios, Rosario, Santos, Jesus, Ruiz, Josefa, and Marquez, Manuel. [Pancreatitis, triglycerides and highly-active antiretroviral therapy]. Medicina clinica 2002. 118 (2) 77-78.

Palau, E., Audibert, L., and Ribera, J. M. Acute pancreatitis during antituberculous treatment. Gastroenterologia y hepatologia 1982. 5 (10) 567-568.

Perego, Elisa, Scaini, Alberto, Romano, Fabrizio, Franciosi, Claudio, and Uggeri, Franco. Estrogen-induced severe acute pancreatitis in a male. JOP: Journal of the pancreas 2004. 5 (5) 353-356.

Pezzilli, R., Billi, P., Melandri, R., Broccoli, P. L., and Fontana, G. Anticonvulsant-induced chronic pancreatitis. A case report. The Italian journal of gastroenterology 1992. 24 (5) 245-246.

Puckett, J. B., Butler, W. M., and McFarland, J. A. Pancreatitis and cancer chemotherapy. Annals of Internal Medicine 1982. 97 (3) 453.

Rashid, J., Starer, P. J., and Javaid, S. Pancreatitis and diabetic ketoacidosis with quetiapine use. Psychiatry 2009. 6 (5) 34-37.

Rawson, R. V., Robbins, E., Kapoor, R., Scolyer, R. A., and Long, G. V. Recurrent bowel obstruction: unusual presentation of pembrolizumab-induced pancreatitis in annular pancreas. European Journal of Cancer 2017. 82:167-170.

Renkes, P. and Trechot, P. Acetaminophen-codeine combination induced acute pancreatitis. Pancreas 1998. 16 (4) 556-561.

Renkes, P., Petitpain, N., Cosserat, F., Bangratz, S., and Trechot, P. Can roxithromycin and betamethasone induce acute pancreatitis? A case report. Journal of the Pancreas 2003. 4 (5) 184-186.

Roquin, Guillaume, Peres, Marine, Lerolle, Nicolas, Dib, Nina, Mercat, Alain, Croue, Anne, and Augusto, Jean Francois. First report of lamotrigine-induced drug rash with eosinophilia and systemic symptoms syndrome with pancreatitis. The Annals of pharmacotherapy 2010. 44 (12) 1998-2000.

Routy, J. P., Smith, G. H., Blank, D. W., and Gilfix, B. M. Plasmapheresis in the treatment of an acute pancreatitis due to protease inhibitor-induced hypertriglyceridemia. Journal of clinical apheresis 2001. 16 (3) 157-159.

Ruinemans, G. M. F., Balemans, C., Mattijssen, V., Wiersma-van Tilburg, A. J., and Smit, H. J. M. Fatal necrotizing pancreatitis during combined treatment with erlotinib and sunitinib. Lung Cancer 2010. 70 (3) 364-365.

Ruman, Jane, Brenner, Steven, and Sauer, Mark V. Severe hypertriglyceridemia and pancreatitis following hormone replacement prior to cryothaw transfer. Journal of assisted reproduction and genetics 2002. 19 (2) 94-97.

Sahni, H., Kirti, K., and Palshetkar, R. Right flank pain as the only symptom for acute pancreatitis in a patient on treatment for HIV infection. Internet Journal of Medical Update 2016. 11 (1) 16-18.

Salvador, V. B., Singh, M., Witek, P., and Peress, G. Cyclophosphamide and doxorubicin-induced acute pancreatitis in a patient with breast cancer. British Journal of Medical Practitioners 2014. 7 (3) no.

Shintani, D., Yoshida, H., Imai, Y., and Fujiwara, K. Acute pancreatitis induced by paclitaxel and carboplatin therapy in an ovarian cancer patient. European Journal of Gynaecological Oncology 2016. 37 (2) 286-287.

Sorodoc, L., Lionte, C., Bologa, C., Petris, O., Sorodoc, V., and Buga, C. Acute pancreatitis after nifedipine and acetaminophen poisoning - Case report. Central European Journal of Medicine 2009. 4 (4) 527-531.

Suzuki, Mitsuyoshi, Takata, Oto, Sakaguchi, Sachi, Fujimura, Junya, Saito, Masahiro, and Shimizu, Toshiaki. Retherapy using L-asparaginase with octreotide in a patient recovering from L-asparaginase-induced pancreatitis. Experimental hematology 2008. 36 (3) 253-254.

Tarin, F., Camps, C., Berrocal, A., and Vicent, J. M. Pancreatitis following cisplatin and vindesin. Revista Espanola de Enfermedades Digestivas 1994. 85 (3) 224-225.

Thompson, Amelia B., Wynn, Bridget A., Akerele, O., Rostad, A., Anderson, Evan J., Camacho-Gonzalez, Andres F., Spearman, Paul, and Chakraborty, Rana. Acute pancreatitis associated with dolutegravir and lamivudine/abacavir administration. AIDS (London, England) 2015. 29 (3) 390-392.

Torello, J., Ibanez, L., Marques, L., and Viladomiu, L. [Acute recurrent pancreatitis caused by estrogens]. Medicina clinica 1989. 93 (6) 238-239.

Tosun, Emine, Oksuzoglu, Berna, and Topaloglu, Oya. Relationship between acute pancreatitis and ACE inhibitors. Acta cardiologica 2004. 59 (5) 571-572.

Trenque-Tessereau, Marie Gilles, Picot, Catherine, Herment, Nathalie, and Trenque, Thierry C. Combined estradiol/gestodene and acute pancreatitis. The Annals of pharmacotherapy 2005. 39 (11) 1953-1954.

Vassallo, P., Green, N., and Courtney, E. Hypercalcemia secondary to excessive self-medication with antacids causing acute pancreatitis: a case report. Croatian Medical Journal 2-28-2019. 60 (1) 42-45.

Vucicevic, Z., Degoricija, V., Alfirevic, Z., and Vukicevic-Badouin, D. Fatal hyponatremia and other metabolic disturbances associated with psychotropic drug polypharmacy. International Journal of Clinical Pharmacology and Therapeutics 2007. 45 (5) 289-292.

Willert, J. R., Dahl, G. V., and Marina, N. M. Letter to the editor: Recurrent mercaptopurine-induced acute pancreatitis: A rare complication of chemotherapy for acute lymphoblastic leukemia in children [3]. Medical and pediatric oncology 2002. 38 (1) 73-74.

Yanar, Fatih, Agcaoglu, Orhan, Sarici, Inanc S., Ozcinar, Beyza, Gok, Ali F. K., Gunay, Kayihan, and Ertekin, Cemalettin. Clinical challenges in drug induced pancreatitis: Presentation of two cases and review of the literature. International journal of surgery case reports 2013. 4 (8) 708-710.

Yucel, Hanifi and Warmerdam, Laurence V. Capecitabine-induced pancreatitis. Journal of oncology pharmacy practice: official publication of the International Society of Oncology Pharmacy Practitioners 2010. 16 (2) 133-134.
