# Supplementary material for: Drug induced pancreatitis: A systematic review of case reports to determine potential drug associations
Source: PLoS One. 2020 Apr 17;15(4):e0231883. doi: 10.1371/journal.pone.0231883 (PMC7164626; doi:10.1371/journal.pone.0231883)
Supplement: S5 Text — (DOCX) [file pone.0231883.s005.docx]

S5 TEXT: Detailed Summary Table – drugs associated with drug-induced pancreatitis

For the citations of case reports associated with each drug, please see Table 4 in the main manuscript. Citations within the table (e.g., for causality assessment tools, can be found after the table.

| **Drug** | **Number of cases (number meeting drug class criteria)** | **Number of overdose/ suicide cases (%)** | **Latency: consistency (%); predominant latency category** | **Re-challenge: positive cases (%); negative cases (%)** | **Causality assessment: number of cases; highest assigned likelihood (tool(s) used to assign highest likelihood)** | **Primary illness associated with acute pancreatitis: number of cases^a^ (%)**  **Other comments regarding risk factors.** | **Other drugs excluded as a cause of DIP:**  **Yes (%);**  **No (%);**  **Unclear (%)** |
| --- | --- | --- | --- | --- | --- | --- | --- |
| **Class Ia** | | | | | | |  |
| 5-aminosalicylic acid (mesalamine) | 31 (6) | 0 (0%) | 76.7; 1–30 days | 11 (35%);  0 (0%) | 7; Probable (Begaud [1], Eland [2], Karch and Lasagna [3], Naranjo [4]) | Chronic bowel^b^: 27 (87%)  Hepatic dysfunction^c^: 2 (6%)  Not reported: 4 (13%) | Yes: 25 (81%);  No: 2 (6%);  Unclear: 4 (13%) |
| Mercaptopurine (6-MP) | 6 (2) | 0 (0%) | 83; 1–30 days | 4 (67%);  0 (0%) | 0 | Chronic bowel: 5 (83%)  Methotrexate discontinued 30 days prior to AP in one case due to hepatotoxicity | Yes: 5 (83%);  No: 0 (0%);  Unclear: 1 (17%) |
| Acetaminophen | 9 (1) | 7 (78%) | 44.4; 1–30 days | 3 (33%);  0 (0%) | 1; Likely (Naranjo [4]) | Cholecystectomy: 2 (22%)  RA: 1 (11%) | Yes: 9 (100%);  No: 0 (0%);  Unclear: 0 (0%) |
| All-trans retinoic acid | 3 (1) | 0 (0%) | 66.7; 1–30 days | 1 (33%);  1 (33%) | 0 | Hypercholesterolemia: 1 (33%) (after control of hypercholesterolemia, ATRA was reinitiated without recurrence of AP) | Yes: 2 (67%);  No: 1 (33%);  Unclear: 0 (0%) |
| Azathioprine | 29 (1) | 0 (0%) | 83; 1–30 days | 9 (31%);  0 (0%) | 5; Definite (Eland [2]) | Chronic bowel: 19 (66%)  Immune disorder^d^: 5 (17%)  Hepatic dysfunction: 3 (10%)  Renal dysfunction: 1 (3%)  In one case, azathioprine was used to treat autoimmune pancreatitis. | Yes: 20 (69%);  No: 1 (3%);  Unclear: 8 (28%) |
| Azodisalicylate/ olsalazine | 3 (1) | 0 (0%) | 66.7; 1–30 days | 2 (67%);  0 (0%) | 1; Probable (Eland [2]) | Chronic bowel: 2 (67%) | Yes: 2 (67%);  No: 0 (0%);  Unclear: 1 (33%) |
| Bezafibrate | 1 | 0 (0%) | NA; 1–30 days | 1 (100%);  0 (0%) | 0 | Hypercholesterolemia: 1 (100%) | Yes: 1 (100%);  No: 0 (0%);  Unclear: 0 (0%) |
| Captopril | 3 (1) | 0 (0%) | 66.7; >30 days | 2 (67%);  0 (0%) | 1; Possible (Eland [2]) | Diabetes: 1 (33%)  RA: 1 (33%) | Yes: 1 (33%);  No: 1 (33%);  Unclear: 1 (33%) |
| Carbimazole | 2 (1) | 0 (0%) | 100; 1–30 days | 1 (50%);  0 (0%) | 0 | None | Yes: 2 (100%);  No: 0 (0%);  Unclear: 0 (0%) |
| Cimetidine | 7 (1) | 0 (0%) | 85.7; 1–30 days | 4 (57%);  0 (0%) | 1; Definite (Eland [2]) | Cholecystectomy: 1 (14%)  Diabetes with renal dysfunction: 1 (14%)  Recent trauma: 1 (14%) | Yes: 5 (71%);  No: 1 (14%);  Unclear: 1 (14%) |
| Codeine | 5 (1) | 0 (0%) | 100; <24 hours | 3 (60%);  0 (0%) | 0 | Previous cholecystectomy: 5 (100%) | Yes: 4 (80%);  No: 1 (20%);  Unclear: 0 (0%) |
| Dapsone | 3 (1) | 0 (0%) | 66.7; >30 days | 1 (33%);  0 (0%) | 2; Probable (Naranjo [4]) | Immune disorder: 3 (100%) | Yes: 3 (100%);  No: 0 (0%);  Unclear: 0 (0%) |
| Erythromycin | 12 (1) | 4 (33%) | 58.3; <24 hours | 1 (8%);  0 (0%) | 0 | Diabetes: 1 (8%)  Hypercholesterolemia: 1 (8%)  Duodenal ulcer in a heavy drinker and smoker: 1 (8%) | Yes: 9 (75%);  No: 1 (8%)  Unclear: 2 (16%) |
| Fluvastatin | 1 (1) | 0 (0%) | NA; >30 days | 1 (100%);  0 (0%) | 0 | Hypercholesterolemia: 1 (100%) | Yes: 1 (100%);  No: 0 (0%);  Unclear: 0 (0%) |
| Furosemide | 4 (1) | 0 (0%) | 75; 1–30 days | 3 (75%);  0 (0%) | 2; Probable (Naranjo [4]) | Dyslipidemia: 1 (25%)  Hyperlipidemia and previous pancreatitis due to sulfonamide: 1 (25%) | Yes: 4 (100%);  No: 0 (0%);  Unclear: 0 (0%) |
| Interferon alpha | 12 (2) | 0 (0%) | 58.3; 1–30 days | 3 (25%);  1 (8%) | 6; Definite (Eland [2], Naranjo [4]) | Cholecystectomy: 1 (8%)  Hepatic dysfunction: 7 (58%) | Yes: 7 (58%);  No: 3 (25%);  Unclear: 2 (17%) |
| Isoniazid | 10 (3) | 0 (0%) | 80; 1–30 days | 5 (50%);  0 (0%) | 0 | Cholecystectomy: 1 (10%)  SLE and renal dysfunction: 1 (10%)  Renal dysfunction^e^: 2 (20%) | Yes: 9 (90%);  No: 1 (10%);  Unclear: 0 (0%) |
| L-asparaginase | 34 (1) | 0 (0%) | 88; 1–30 days | 3 (9%);  1 (3%) | 12; Probable (Delcenserie 2001 [5]) | Hepatic dysfunction: 2 (6%)  At least one case died of multi-organ failure, with AP diagnosed at autopsy. | Yes: 9 (28%);  No: 8 (25%);  Unclear: 15 (47%) |
| Lisinopril | 6 (1) | 0 (0%) | 66.7; >30 days | 1 (17%);  0 (0%) | 2; Probable (FDA algorithm (cited in Maliekal and Drake [6], Naranjo [4]) | Cholecystectomy: 1 (17%)  Diabetes: 3 (50%)  Familial hypertriglyceridemia: 1 (17%) | Yes: 4 (67%);  No: 1 (17%);  Unclear: 1 (17%) |
| Metformin | 4 (1) | 1 (25%) | 50; <24 hours | 1 (25%);  0 (0%) | 0 | Diabetes: 2 (50%) | Yes: 2 (50%);  No: 2 (50%);  Unclear: 0 (0%) |
| Methimazole | 6 (3) | 0 (0%) | 83; 1–30 days | 3 (60%);  0 (0%) | 3; Definite (Karch and Lasagna [3], Mallory and Kern [7]) | None | Yes: 6 (100%);  No: 0 (0%);  Unclear: 0 (0%) |
| Methylprednisolone | 6 (1) | 0 (0%) | 83; 1–30 days | 2 (33%);  0 (0%) | 1; Probable (Naranjo [4]) | Cholecystectomy: 1 (17%)  Immune disorder: 3 (50%)  Hepatic dysfunction: 1 (17%)  Hyperlipidemia: 1 (17%)  Undiagnosed diabetes: 1 (17%) | Yes: 5 (83%);  No: 0 (0%);  Unclear: 1 (17%) |
| Metronidazole | 11 (2) | 0 (0%) | 81.8; 1–30 days | 4 (36%);  0 (0%) | 2; Definite (Eland [2]) | Chronic bowel: 2 (18%)  HIV/AIDS: 1 (9%)  Renal dysfunction: 1 (9%) | Yes: 9 (75%);  No: 1 (8%);  Unclear: 2 (17%) |
| Nitrofurantoin | 3 (1) | 0 (0%) | 100; 1–30 days | 2 (67%);  0 (0%) | 0 | Diabetes and pancreatic carcinoma: 1 (33%) | Yes: 3 (100%);  No: 0 (0%);  Unclear: 0 (0%) |
| Orlistat | 1 | 0 (0%) | NA; 1–30 days | 1 (100%);  0 (0%) | 0 | None | Yes: 1 (100%);  No: 0 (0%);  Unclear: 0 (0%) |
| Piroxicam | 1 | 0 (0%) | NA; 1–30 days | 1 (100%);  0 (0%) | 1; Probable (Delcenserie 2001 [5]) | None | Yes: 1 (100%);  No: 0 (0%);  Unclear: 0 (0%) |
| Pravastatin | 2 (1) | 0 (0%) | 0; 1–30 days | 1 (50%);  0 (0%) | 0 | Cholecystectomy: 1 (50%)  Diabetes and hyperlipidemia: 1 (50%)  Hypercholesterolemia: 1 (50%) | Yes: 1 (50%);  No: 1 (50%);  Unclear: 0 (0%) |
| Prednisone | 8 (1) | 0 (0%) | 50; 1–30 and >30 days | 2 (25%);  0 (0%) | 0 | Chronic bowel: 2 (25%)  HIV/AIDS: 1 (13%)  Immune disorder: 5 (63%)  Renal dysfunction: 1 (13%) | Yes: 2 (25%);  No: 6 (75%);  Unclear: 0 (0%) |
| Premarin | 2 (1) | 0 (0%) | 0; 1–30 days | 1 (50%);  0 (0%) | 0 | Diabetes: 1 (50%) | Yes: 1 (50%);  No: 1 (50%);  Unclear: 0 (0%) |
| Procainamide | 1 | 0 (0%) | NA; >30 days | 1 (100%);  0 (0%) | 0 | None | Yes: 1 (100%);  No: 0 (0%);  Unclear: 0 (0%) |
| Pyritinol | 1 | 0 (0%) | NA; >30 days | 1 (100%);  0 (0%) | 0 | None | Yes: 1 (100%);  No: 0 (0%);  Unclear: 0 (0%) |
| Ramipril | 4 (1) | 0 (0%) | 50; 1–30 and >30 days | 1 (25%);  0 (0%) | 1; Probable (Karch and Lasagna [3]) | SLE: 1 (25%)  Renal dysfunction: 1 (25%) | Yes: 3 (75%);  No: 0 (0%);  Unclear: 1 (25%) |
| Ranitidine | 1 | 0 (0%) | NA; >30 days | 1 (100%);  0 (0%) | 0 | Chronic bowel: 1 (100%) | Yes: 1 (100%);  No: 0 (0%);  Unclear: 0 (0%) |
| Rosuvastatin | 1 | 0 (0%) | NA; 1–30 days | 1 (100%);  0 (0%) | 1; Probable (Naranjo [4]) | Hyperlipidemia: 1 (100%) | Yes: 1 (100%);  No: 0 (0%);  Unclear: 0 (0%) |
| Simvastatin | 6 (2) | 0 (0%) | 66.7; >30 days | 2 (33%);  0 (0%) | 0 | Diabetes and hyperlipidemia: 1 (17%)  Hypercholesterolemia: 4 (67%) | Yes: 6 (83%);  No: 0 (0%);  Unclear: 1 (17%) |
| Sorafenib | 7 (1) | 0 (0%) | 86; 1–30 days | 1 (14%);  0 (0%) | 2; Probable (Naranjo [4]) | Cholecystectomy: 1 (14%)  Diabetes and renal dysfunction: 1 (20%)  Hepatic dysfunction: 2 (29%)  Renal dysfunction: 4 (80%) | Yes: 6 (86%);  No: 0 (0%);  Unclear: 1 (14%) |
| Sulindac | 9 (3) | 0 (0%) | 55.6; >30 days | 7 (78%);  0 (0%) | 1; Probable (Eland [2]) | Diabetes and hyperlipoproteinemia: 1 (11%)  Immune disorder: 2 (22%) | Yes: 7 (78%);  No: 0 (0%);  Unclear: 2 (22%) |
| Tamoxifen | 6 (1) | 0 (0%) | 83; >30 days | 2 (33%);  0 (0%) | 0 | Diabetes: 3 (60%)  Hypertriglyceridemia (controlled): 1 (20%)  Hyperlipidemia (sub-clinical?): 1 (20%) | Yes: 6 (100%);  No: 0 (0%);  Unclear: 0 (0%) |
| Telaprevir | 1 | 0 (0%) | NA; 1–30 days | 1 (100%);  0 (0%) | 0 | Hepatic dysfunction: 1 (100%) | Yes: 1 (100%);  No: 0 (0%);  Unclear: 0 (0%) |
| Tetracycline | 5 (1) | 0 (0%) | 60; 1–30 days | 2 (40%);  0 (0%) | 1; Possible (Eland [2]) | None | Yes: 4 (80%);  No: 0 (0%);  Unclear: 1 (20%) |
| Thalidomide | 1 | 0 (0%) | NA; >30 days | 1 (100%);  0 (0%) | 1; Probable (Mallory and Kern [7]) | GVHD: 1 (100%) | Yes: 1 (100%);  No: 0 (0%);  Unclear: 0 (0%) |
| Trimethoprim-sulfamethoxazole | 9 (2) | 0 (0%) | 78; 1–30 days | 4 (44%);  0 (0%) | 2; Definite (Karch and Lasagna [3], Naranjo [4]) | Cholecystectomy: 1 (11%)  Chronic bowel: 1 (11%)  HIV/AIDS: 4 (44%)  Hepatic dysfunction: 2 (22%) | Yes: 8 (89%);  No: 1 (11%);  Unclear: 0 (0%) |
| Vemurafenib | 1 | 0 (0%) | NA; 1–30 days | 1 (100%);  0 (0%) | 1; Definite (Naranjo [4]) | None | Yes: 1 (100%);  No: 0 (0%);  Unclear: 0 (0%) |
| Valproic acid | 56 (3) | 1 (2%) | 86; >30 days | 8 (14%);  1 (2%) | 1; Plausible (Kramer [8]) | Cholecystectomy: 1 (2%)  Diabetes: 1 (2%)  Renal dysfunction: 2 (4%)  Sepsis: 1 (2%)  Chicken pox exposure: 1 (2%) | Yes: 45 (80%);  No: 3 (5%);  Unclear: 8 (14%) |
| **Class Ib** | | | | | | | |
| Amiodarone | 3 (1) | 0 (0%) | 100; 1–30 days | 1 (33%);  0 (0%) | 1; Probable (Naranjo [4]) | Cholecystectomy: 1 (33%) | Yes: 2 (67%);  No: 1 (33%);  Unclear: 0 (0%) |
| Ampicillin | 1 | 0 (0%) | NA; 1–30 days | 1 (100%);  0 (0%) | 0 | None | Yes: 1 (100%);  No: 0 (0%);  Unclear: 0 (0%) |
| Antilymphocyte globulin | 1 | 0 (0%) | NA; 1–30 days | 1 (100%);  0 (0%) | 0 | Renal dysfunction: 1 (100%) | Yes: 1 (100%);  No: 0 (0%);  Unclear: 0 (0%) |
| Carbamazepine | 5 (2) | 1 (20%) | 60; 1–30 days | 2 (40%);  0 (0%) | 1; Probable (Naranjo [4]) | HIV/AIDS: 1 (20%)  Naranjo “probable” case was admitted for gallstone-induced AP 6 months later, causing authors to acknowledge that drug may not have been the initial cause. | Yes: 3 (60%);  No: 2 (40%);  Unclear: 0 (0%) |
| Ciprofloxacin | 1 | 0 (0%) | NA; 1–30 days | 1 (100%);  0 (0%) | 0 | None | Yes: 1 (100%);  No: 0 (0%);  Unclear: 0 (0%) |
| Clomiphene | 2 (2) | 0 (0%) | 100; >30 days | 2 (100%);  0 (0%) | 0 | Diabetes and hypertriglyceridemia: 1 (50%) | Yes: 2 (100%);  No: 0 (0%);  Unclear: 0 (0%) |
| Clothiapine | 1 | 0 (0%) | NA; 1–30 days | 1 (100%);  0 (0%) | 0 | None | Yes: 1 (100%);  No: 0 (0%);  Unclear: 0 (0%) |
| Clozapine | 9 (2) | 1 (11%) | 77.8; 1–30 days | 2 (22%);  1 (11%) | 1; Possible (Mallory and Kern [7]) | Diabetes with slowly rising triglyceride levels over 3 years of clozapine therapy: 1 (11%) | Yes: 6 (67%);  No: 2 (22%);  Unclear: 1 (11%) |
| Cytarabine | 4 (2) | 0 (0%) | 100; 1–30 days | 2 (50%);  0 (0%) | 0 | None | Yes: 2 (50%);  No: 2 (50%);  Unclear: 0 (0%) |
| Dexamethasone | 1 | 0 (0%) | NA; 1–30 days | 1 (100%);  0 (0%) | 0 | “Numerous severe medical problems” but none believed to be the cause of AP: 1 (100%) | Yes: 0 (0%);  No: 0 (0%);  Unclear: 1 (100%) |
| Didanosine | 8 (2) | 0 (0%) | 75; >30 days | 2 (25%);  0 (0%) | 1; Possible (Eland [2]) | HIV/AIDS: 8 (100%)  At least one patient had multiple severe manifestations of HIV, progressing to death. | Yes: 0 (0%);  No: 5 (63%);  Unclear: 3 (37%) |
| Diphenoxylate+atropine | 1 | 0 (0%) | NA; <24 hours | 1 (100%);  0 (0%) | 0 | Cholecystectomy: 1 (100%) | Yes: 0 (0%);  No: 1 (100%);  Unclear: 0 (0%) |
| Eluxadoline | 2 (1) | 0 (0%) | 0; <24 hours and 1–30 days | 1 (50%);  0 (0%) | 0 | Cholecystectomy: 2 (100%)  Chronic bowel: 2 (100%) | Yes: 1 (50%)  No: 0 (0%)  Unclear: 1 (50%) |
| Enalapril | 10 (1) | 0 (0%) | 80; >30 days | 2 (20%);  0 (0%) | 3; Probable (Begaud [1], Eland [2], Karch and Lasagna [3]) | Renal dysfunction: 1 (10%)—renal insufficiency and multi-drug therapy may have caused toxicity in this patient. | Yes: 7 (70%);  No: 0 (0%);  Unclear: 3 (30%) |
| Everolimus | 2 (1) | 0 (0%) | 100; >30 days | 1 (50%);  0 (0%) | 0 | Cholecystectomy: 1 (50%)  Dyslipidemia: 1 (50%) | Yes: 1 (50%);  No: 1 (50%);  Unclear: 0 (0%) |
| Growth Hormone | 2 (1) | 0 (0%) | 100; 1–30 days | 1 (50%);  0 (0%) | 0 | None | Yes: 1 (50%);  No: 1 (50%);  Unclear: 0 (0%) |
| Hydrochlorothiazide | 2 (1) | 0 (0%) | 100; 1–30 days | 1 (50%);  0 (0%) | 0 | Previous pancreatitis: 1 (50%)  One case may have been idiopathic as it was mild and improved over 24 hours. | Yes: 0 (0%);  No: 2 (100%);  Unclear: 0 (0%) |
| Hydrocortisone | 1 | 0 (0%) | NA; 1–30 days | 1 (100%);  0 (0%) | 0 | Chronic bowel: 1 (100%) | Yes: 1 (100%);  No: 0 (0%);  Unclear: 0 (0%) |
| Ifosfamide | 4 (2) | 0 (0%) | 100; 1–30 days | 2 (50%);  0 (0%) | 0 | Renal dysfunction: 1 (25%) | Yes: 2 (50%);  No: 2 (50%);  Unclear: 0 (0%) |
| Indalpine | 1 | 0 (0%) | NA; >30 days | 1 (100%);  0 (0%) | 1; Probable (Dangoumau [9] ) | None | Yes: 0 (0%);  No: 0 (0%);  Unclear: 1 (100%) |
| Lamivudine | 3 (1) | 0 (0%) | 66.7; 1–30 days | 1 (33%);  0 (0%) | 1; Possible (Eland [2]) | Hepatic dysfunction: 2 (67%)  HIV/AIDS: 1 (33%) | Yes: 2 (67%);  No: 0 (0%);  Unclear: 1 (33%) |
| Losartan | 3 (2) | 0 (0%) | 100; 1–30 days | 2 (67%);  0 (0%) | 1; Probable (Karch and Lasagna [3]) | Diabetes and renal dysfunction: 1 (33%) | Yes: 2 (67%);  No: 1 (33%);  Unclear: 0 (0%) |
| Mefenamic acid | 3 (2) | 0 (0%) | 66.7; 1–30 days | 2 (67%);  0 (0%) | 0 | None | Yes: 3 (100%);  No: 0 (0%);  Unclear: 0 (0%) |
| Meglumine antimoniate | 5 (1) | 0 (0%) | 100; 1–30 days | 1 (20%);  0 (0%) | 0 | HIV/AIDS with Hepatic dysfunction: 1 (20%) | Yes: 5 (100%);  No: 0 (0%);  Unclear: 0 (0%) |
| Methyldopa | 3 (3) | 0 (0%) | 100; 1–30 days | 3 (100%);  0 (0%) | 0 | None | Yes: 1 (33%);  No: 0 (0%);  Unclear: 2 (67%) |
| Mirtazapine | 4 (2) | 0 (0%) | 75; >30 days | 2 (50%);  0 (0%) | 1; Probable (Naranjo [4]) | None | Yes: 2 (50%);  No: 1 (25%);  Unclear: 1 (25%) |
| Nelfinavir | 1 | 0 (0%) | NA; >30 days | 1 (100%);  0 (0%) | 0 | Cholecystectomy: 1 (100%)  HIV/AIDS: 1 (100%)  Didanosine also administered and not discussed as a potential cause. | Yes: 0 (0%);  No: 1 (100%);  Unclear: 0 (0%) |
| Octreotide | 6 (1) | 0 (0%) | 50; <24 hours and >30 days | 1 (17%);  0 (0%) | 0 | HIV/AIDS: 2 (33%) | Yes: 3 (50%);  No: 0 (0%);  Unclear: 3 (50%) |
| Omeprazole | 1 | 0 (0%) | NA; >30 days | 1 (100%);  0 (0%) | 1; Definite (Mallory and Kern [7]) | Cholecystectomy: 1 (100%)  Hyperlipidemia and renal dysfunction: 1 (100%) | Yes: 1 (100%);  No: 0 (0%);  Unclear: 0 (0%) |
| Oral contraceptive | 3 (2) | 0 (0%) | 66.7; >30 days | 2 (67%);  0 (0%) | 0 | None | Yes: 3 (100%);  No: 0 (0%);  Unclear: 0 (0%) |
| Oxyphenbutazone | 2 (1) | 0 (0%) | 100; 1–30 days | 1 (50%);  0 (0%) | 1; Definite (Eland [2]) | None | Yes: 1 (50%);  No: 0 (0%);  Unclear: 1 (50%) |
| Paclitaxel | 4 (1) | 0 (0%) | 75; 1–30 days | 1 (25%);  0 (0%) | 1; Definite (Naranjo [4]) | At least one patient had multi-organ sepsis and failure over course of AP treatment. | Yes: 3 (75%);  No: 1 (25%);  Unclear: 0 (0%) |
| Paromomycin | 1 | 0 (0%) | NA; 1–30 days | 1 (100%);  0 (0%) | 0 | HIV/AIDS: 1 (100%)  Previous exposure to didanosine/ octreotide/ pentamidine, alcoholism, or Cryptosporidium may have inflamed pancreas prior to paromomycin administration. Weak association. | Yes: 1 (100%);  No: 0 (0%);  Unclear: 0 (0%) |
| Pentamidine | 20 (2) | 0 (0%) | 80; 1–30 days | 2 (10%);  0 (0%) | 1; Probable (Naranjo [4]) | HIV/AIDS: 19 (95%)  Hepatic dysfunction: 1 (5%)  Renal dysfunction: 2 (10%)  At least one case died of multi-organ failure and multiple systemic infections.  Previous exposure to didanosine may have predisposed to AP in at least one case. | Yes: 14 (70%);  No: 3 (15%);  Unclear: 3 (15%) |
| Perindopril | 2 (1) | 0 (0%) | 100; 1–30 days | 1 (50%);  0 (0%) | 1; Probable (Naranjo [4]) | Cholecystectomy: 1 (50%)  Diabetes: 2 (100%) | Yes: 2 (100%);  No: 0 (0%);  Unclear: 0 (0%) |
| Prednisolone | 4 (1) | 0 (0%) | 75; 1–30 days | 1 (25%);  0 (0%) | 0 | Chronic bowel: 3 (75)  Bullous pemphigoid: 1 (25%) | Yes: 1 (25%);  No: 0 (0%);  Unclear: 3 (75%) |
| Propofol | 12 (1) | 0 (0%) | 58; 1–30 days | 2 (17%);  0 (0%) | 1; Probable (Naranjo [4]) | Cholecystectomy: 1 (8%) | Yes: 4 (33%);  No: 5 (42%);  Unclear: 3 (25%) |
| Quetiapine | 2 (1) | 0 (0%) | 100; >30 days | 1 (50%);  0 (0%) | 1; Probable (Naranjo [4]) | Previous valproic acid-induced AP: 1 (50%) | Yes: 1 (50%);  No: 1 (50%);  Unclear: 0 (0%) |
| Rifampicin | 1 | 0 (0%) | NA; >30 days | 1 (100%);  0 (0%) | 0 | None | Yes: 1 (100%);  No: 0 (0%);  Unclear: 0 (0%) |
| Risperidone | 4 (1) | 0 (0%) | 75; 1–30 days | 1 (25%);  0 (0%) | 0 | Hypercholesterolemia: 1 (25%) | Yes: 1 (50%);  No: 0 (0%);  Unclear: 1 (50%) |
| Salazopyrine | 1 | 0 (0%) | NA; <24 hours | 1 (100%);  0 (0%) | 0 | Chronic bowel: 1 (100%) | Yes: 1 (100%);  No: 0 (0%);  Unclear: 0 (0%) |
| Saxagliptin | 1 | 0 (0%) | NA; >30 days | 1 (100%);  0 (0%) | 0 | Diabetes: 1 (100%) | Yes: 1 (100%);  No: 0 (0%);  Unclear: 0 (0%) |
| Stibogluconate | 18 (7) | 1 (6%) | 100; 1–30 days | 7 (39%);  0 (0%) | 0 | HIV/AIDS: 6 (33%)  Renal dysfunction: 2 (11%)  Multi-organ failure leading to death occurred in at least one case. | Yes: 6 (33%);  No: 4 (22%);  Unclear: 8 (44%) |
| Sulfasalazine | 8 (3) | 0 (0%) | 75; 1–30 days | 3 (38%);  0 (0%) | 0 | Chronic bowel: 8 (100%) | Yes: 6 (75%);  No: 2 (25%);  Unclear: 0 (0%) |
| Tigecycline | 10 (1) | 0 (0%) | 100; 1–30 days | 2 (20%);  0 (0%) | 4; Probable (Mallory and Kern [7], Naranjo [4]) | Cholecystectomy: 1 (11%)  Chronic bowel with RA: 1 (11%)  Renal dysfunction: 3 (30%)  Hyperlipidemia: 1 (11%)  Authors state population receiving tigecycline for multi-drug resistant organisms have numerous underlying conditions and may have an altered catabolism, making them more susceptible to the pancreatotoxic effects of tigecycline. | Yes: 7 (70%);  No: 3 (30%);  Unclear: 0 (0%) |
| Valsartan | 1 | 0 (0%) | NA; >30 days | 1 (100%);  0 (0%) | 0 | Cholecystectomy: 1 (100%)  Pancreatitis due to gallstones and possibly drug 6 weeks prior: 1 (100%) | Yes: 1 (100%);  No: 0 (0%);  Unclear: 0 (0%) |
| Voriconazole | 1 | 0 (0%) | NA; 1–30 days | 1 (100%);  0 (0%) | 0 | None | Yes: 1 (100%);  No: 0 (0%);  Unclear: 0 (0%) |
| **Class Ic** | | | | | | | |
| Adefovir dipivoxil | 1 | 0 (0%) | NA; 1–30 days | 0 (0%);  0 (0%) | 0 | Cholecystectomy: 1 (100%)  Hepatic dysfunction: 1 (100%) | Yes: 1 (100%);  No: 0 (0%);  Unclear: 0 (0%) |
| Amoxicillin + clavulanic acid | 2 (1) | 0 (0%) | 0; 1–30 days and >30 days | 0 (0%);  0 (0%) | 0 | None | Yes: 2 (100%)  No: 0 (0%)  Unclear: 0 (0%) |
| Artesunate | 1 | 0 (0%) | 1–30 days | 0 (0%);  0 (0%) | 1; Probable (Naranjo [4]) | Malaria: 1 (100%) | Yes: 1 (100%)  No: 0 (0%)  Unclear: 0 (0%) |
| Atorvastatin | 3 (1) | 0 (0%) | 66.7; >30 days | 0 (0%);  0 (0%) | 2; Probable, C-2 (Naranjo [4], WHO [10]) | Hyperlipidemia: 2 (67%)  Hypercholesterolemia: 1 (33%) | Yes: 3 (100%);  No: 0 (0%);  Unclear: 0 (0%) |
| Axitinib | 2 (1) | 0 (0%) | 100; >30 days | 0 (0%);  0 (0%) | 0 | Renal dysfunction: 2 (100%) | Yes: 2 (100%);  No: 0 (0%);  Unclear: 0 (0%) |
| Boceprevir | 1 | 0 (0%) | NA; >30 days | 0 (0%);  0 (0%) | 1, Probable (Naranjo [4]) | Cholecystectomy: 1 (100%)  Hepatic dysfunction: 1 (100%) | Yes: 1 (100%);  No: 0 (0%);  Unclear: 0 (0%) |
| Bortezomib | 2 (1) | 0 (0%) | 100; 1–30 days | 0 (0%);  0 (0%) | 1; Probable (Naranjo [4]) | None | Yes: 1 (100%);  No: 0 (0%);  Unclear: 0 (0%) |
| Canaglifozin | 2 (1) | 0 (0%) | 0; 1–30 days and >30 days | 0 (0%);  0 (0%) | 0 | Diabetes: 2 (100%)  Renal dysfunction: 1 (50%) | Yes: 1 (50%);  No: 0 (0%);  Unclear: 1 (50%) |
| Candesartan | 1 | 0 (0%) | NA; >30 days | 0 (0%);  0 (0%) | 0 | Renal dysfunction: 1 (100%) | Yes: 1 (100%);  No: 0 (0%);  Unclear: 0 (0%) |
| Celecoxib | 4 (3) | 0 (0%) | 75; 1–30 days | 0 (0%);  0 (0%) | 1; Probable (Delcenserie 2001 [5]) | Cholecystectomy: 1 (25%)  Renal dysfunction: 1 (25%) | Yes: 2 (50%);  No: 1 (25%);  Unclear: 1 (25%) |
| Clarithromycin | 5 (2) | 0 (0%) | 80; 1–30 days | 0 (0%);  0 (0%) | 3; Probable (Naranjo [4]) | One morbidly ill patient experienced progressive organ dysfunction, shock, hypotension, and death. AP was diagnosed at autopsy. | Yes: 4 (80%);  No: 0 (0%);  Unclear: 1 (20%) |
| Danazol | 1 | 0 (0%) | NA; >30 days | 0 (0%);  0 (0%) | 0 | None | Yes: 1 (100%);  No: 0 (0%);  Unclear: 0 (0%) |
| Dexfenfluramine | 1 | 0 (0%) | NA; 1–30 days | 0 (0%);  0 (0%) | 0 | None | Yes: 1 (100%);  No: 0 (0%);  Unclear: 0 (0%) |
| Diclofenac | 2 (1) | 0 (0%) | 100; 1–30 days | 0 (0%);  0 (0%) | 0 | Hyperlipidemia: 1 (50%) | Yes: 2 (100%)  No: 0 (0%)  Unclear: 0 (0%) |
| Diethylstilbestrol | 1 | 0 (0%) | NA; >30 days | 0 (0%);  0 (0%) | 0 | Metastases due to prostate cancer could not be ruled out: 1 (100%) | Yes: 1 (100%);  No: 0 (0%);  Unclear: 0 (0%) |
| Dilantin | 1 | 0 (0%) | NA; 1–30 days | 0 (0%);  0 (0%) | 1; Definite (Mallory and Kern [7]) | None | Yes: 1 (100%);  No: 0 (0%);  Unclear: 0 (0%) |
| Dimethyl fumarate | 1 | 0 (0%) | NA; 1–30 days | 0 (0%);  0 (0%) | 1; Probable (Mallory and Kern [7]) | Cholecystectomy: 1 (100%) | Yes: 1 (100%);  No: 0 (0%);  Unclear: 0 (0%) |
| Doxycycline | 4 (1) | 0 (0%) | 100; 1–30 days | 0 (0%);  0 (0%) | 3; Probable (Eland [2], Naranjo [4]) | Cholecystectomy: 2 (50%) | Yes: 3 (75%);  No: 0 (0%);  Unclear: 1 (25%) |
| Ezetimibe | 1 | 0 (0%) | NA; 1–30 days | 0 (0%);  0 (0%) | 0 | Diabetes: 1 (100%) | Yes: 1 (100%);  No: 0 (0%);  Unclear: 0 (0%) |
| Finasteride | 1 | 0 (0%) | NA; >30 days | 0 (0%);  0 (0%) | 0 | None | Yes: 1 (100%);  No: 0 (0%);  Unclear: 0 (0%) |
| Flurbiprofen | 1 | 0 (0%) | NA; <24 hours | 0 (0%);  0 (0%) | 1; Highly probable (Naranjo [4]) | Previous pancreatitis induced by ketoprofen: 1 (100%) | Yes: 1 (100%);  No: 0 (0%);  Unclear: 0 (0%) |
| Gadolinium | 3 (2) | 0 (0%) | 100; <24 hours | 0 (0%);  0 (0%) | 0 | Drug may have exacerbated a gallstone-related pancreatitis in one patient | Yes: 2 (67%);  No: 0 (0%);  Unclear: 1 (33%) |
| Glicazide | 1 | 0 (0%) | NA; >30 days | 0 (0%);  0 (0%) | 0 | Diabetes: 1 (100%)  Episode occurred 3 years after starting medication and no re-challenge was performed—may be idiopathic. | Yes: 1 (100%);  No: 0 (0%);  Unclear: 0 (0%) |
| Glimepiride | 1 | 0 (0%) | NA; 1–30 days | 0 (0%);  0 (0%) | 1; Probable (Delcenserie 2001 [5]) | Diabetes: 1 (100%) | Yes: 1 (100%);  No: 0 (0%);  Unclear: 0 (0%) |
| Ibuprofen | 3 (2) | 2 (67%) | 66.7; <24 hours | 0 (0%);  0 (0%) | 2; Probable (Eland [2], Mallory and Kern [7]) | Cholecystectomy: 1 (33%) | Yes: 1 (50%);  No: 0 (0%);  Unclear: 1 (50%) |
| Interferon beta | 1 | 0 (0%) | NA; >30 days | 0 (0%);  0 (0%) | 0 | None | Yes: 1 (100%);  No: 0 (0%);  Unclear: 0 (0%) |
| Indomethacin | 2 (2) | 0 (0%) | 0; 1–30 and >30 days | 0 (0%);  0 (0%) | 0 | None | Yes: 2 (100%);  No: 0 (0%);  Unclear: 0 (0%) |
| Irbesartan | 1 | 0 (0%) | NA; 1–30 days | 0 (0%);  0 (0%) | 1; Probable (Naranjo [4]) | None | Yes: 1 (100%);  No: 0 (0%);  Unclear: 0 (0%) |
| Itraconazole | 2 (1) | 1 (50%) | 0; 1–30 and >30 days | 0 (0%);  0 (0%) | 0 | Diabetes and hyperlipidemia: 1 (50%) | Yes: 1 (50%);  No: 1 (50%);  Unclear: 0 (0%) |
| Ixazomib | 1 | 0 (0%) | 1–30 days | 0 (0%);  0 (0%) | 1; Likely (Karch and Lasagna [3]) | Cholecystectomy: 1 (100%)  Renal dysfunction: 1 (100%) | Yes: 1 (100%)  No: 0 (0%)  Unclear: 0 (0%) |
| Ketoprofen | 2 (1) | 0 (0%) | 100; 1–30 days | 0 (0%);  0 (0%) | 0 | Cholecystectomy: 1 (50%) | Yes: 2 (100%);  No: 0 (0%);  Unclear: 0 (0%) |
| Ketorolac | 2 (1) | 0 (0%) | 0; <24 hours and 1–30 days | 0 (0%);  0 (0%) | 1; Probable (Naranjo [4]) | Diabetes: 1 (50%) | Yes: 2 (100%);  No: 0 (0%);  Unclear: 0 (0%) |
| Lanreotide | 2 (1) | 0 (0%) | 0; <24 hours and 1–30 days | 0 (0%);  0 (0%) | 0 | None | Yes: 1 (50%);  No: 0 (0%);  Unclear: 1 (50%) |
| Lenvatinib | 1 | 0 (0%) | >30 days | 0 (0%);  0 (0%) | 0 | Renal dysfunction: 1 (100%) | Yes: 1 (100%)  No: 0 (0%)  Unclear: 0 (0%) |
| Liraglutide | 5 (2) | 0 (0%) | 100; >30 days | 0 (0%);  0 (0%) | 4; Probable (Begaud [1], Naranjo [4]) | Diabetes: 5 (100%)  Renal dysfunction: 2 (40%)  Hyperlipidemia: 2 (40%)  Valproic acid-induced AP previously: 1 (20%) | Yes: 4 (80%);  No: 1 (20%);  Unclear: 0 (0%) |
| Meprobamate | 1 | 1 (100%) | NA; <24 hours | 0 (0%);  0 (0%) | 1; Probable (Naranjo [4]) | None | Yes: 1 (100%);  No: 0 (0%);  Unclear: 0 (0%) |
| Metolazone | 2 (1) | 0 (0%) | 100; 1–30 days | 0 (0%);  0 (0%) | 0 | Cholecystectomy: 1 (50%)  Diabetes: 2 (100%)  Renal dysfunction: 1 (50%) | Yes: 2 (100%);  No: 0 (0%);  Unclear: 0 (0%) |
| Minocycline | 5 (2) | 0 (0%) | 80; >30 days | 0 (0%);  0 (0%) | 2, Doubtful and probable (Begaud [1]) | Cystic fibrosis class V mutation: 2 (50%)  Hypercholesterolemia: 1 (25%) | Yes: 4 (80%);  No: 1 (20%);  Unclear: 0 (0%) |
| Naltrexone | 2 (1) | 0 (0%) | 0; 1–30 days and >30 days | 0 (0%);  0 (0%) | 0 | None | Yes: 1 (50%);  No: 0 (0%);  Unclear: 1 (50%) |
| Naproxen | 3 (3) | 1 (33%) | 66.7; <24 hours | 0 (0%);  0 (0%) | 0 | None | Yes: 3 (100%);  No: 0 (0%);  Unclear: 0 (0%) |
| Nilotinib | 3 (2) | 0 (0%) | 66.7; 1–30 days | 0 (0%);  0 (0%) | 1; Possible (Naranjo [4]) | Hypercholesterolemia: 1 (33%)  Hyperhistaminemia was not ruled out in one CML case. | Yes: 3 (100%);  No: 0 (0%);  Unclear: 0 (0%) |
| Olanzapine | 10 (1) | 0 (0%) | 90; >30 days | 0 (0%);  0 (0%) | 3; Probable (Naranjo [4]) | Cholecystectomy: 1 (10%)  Diabetes and hyperlipidemia: 1 (10%)  Valproic acid may have increased the risk of AP in one patient. | Yes: 7 (70%);  No: 2 (20%);  Unclear: 1 (10%) |
| Pantoprazole | 1 | 0 (0%) | NA; 1–30 days | 0 (0%);  0 (0%) | 1; Probable (Naranjo [4]) | None | Yes: 1 (100%);  No: 0 (0%);  Unclear: 0 (0%) |
| Propylthiouracil | 1 | 0 (0%) | NA; 1–30 days | 0 (0%);  0 (0%) | 1; Probable (Delcenserie 1992 [11]) | None | Yes: 1 (100%);  No: 0 (0%);  Unclear: 0 (0%) |
| Riluzole | 3 (2) | 0 (0%) | 100; >30 days | 0 (0%);  0 (0%) | 2; Probable (Naranjo [4]) | Cholecystectomy: 1 (33%)  Hyperlipidemia: 1 (33%)  In one case, the authors stated that they could not prove DIP due to multiple other issues (ALS, etc.). | Yes: 2 (67%);  No: 1 (33%);  Unclear: 0 (0%) |
| Rofecoxib | 2 (2) | 0 (0%) | 0; <24 hours and 1–30 days | 0 (0%);  0 (0%) | 0 | Hepatic dysfunction: 2 (100%) | Yes: 2 (100%);  No: 0 (0%);  Unclear: 0 (0%) |
| Secnidazole | 1 | 0 (0%) | NA; 1–30 days | 0 (0%);  0 (0%) | 0 | None | Yes: 1 (100%);  No: 0 (0%);  Unclear: 0 (0%) |
| Sirolimus | 1 | 0 (0%) | NA; 1–30 days | 0 (0%);  0 (0%) | 0 | Renal dysfunction: 1 (100%) | Yes: 1 (100%);  No: 0 (0%);  Unclear: 0 (0%) |
| Theophylline | 1 | 1 (100%) | NA; <24 hours | 0 (0%);  0 (0%) | 0 | None | Yes: 1 (100%);  No: 0 (0%);  Unclear: 0 (0%) |
| Tiaprofenic acid | 1 | 0 (0%) | NA; 1–30 days | 0 (0%);  0 (0%) | 0 | None | Yes: 1 (100%);  No: 0 (0%);  Unclear: 0 (0%) |
| Tinidazole | 1 | 0 (0%) | NA; 1–30 days | 0 (0%);  0 (0%) | 1; Probable (Naranjo [4]) | None | Yes: 1 (100%);  No: 0 (0%);  Unclear: 0 (0%) |
| Vedolizumab | 1 | 0 (0%) | <24 hours | 0 (0%);  0 (0%) | 0 | Chronic bowel: 1 (100%) | Yes: 1 (100%)  No: 0 (0%)  Unclear: 0 (0%) |
| Vildagliptin | 2 (1) | 0 (0%) | 0; 1–30 and >30 days | 0 (0%);  0 (0%) | 0 | Cholecystectomy: 1 (50%)  Diabetes: 2 (100%) | Yes: 2 (100%);  No: 0 (0%);  Unclear: 0 (0%) |
| **Class II** | | | | | | | |
| Ceftriaxone | 5 | 0 (0%) | 100; 1–30 days | 0 (0%);  0 (0%) | 0 | Chronic bowel: 1 (25%)  Renal dysfunction: 2 (50%)  RA: 1 (25%) | Yes: 4 (80%);  No: 0 (0%);  Unclear: 1 (20%) |
| Clofibrate | 2 | 0 (0%) | 100; >30 days | 0 (0%);  0 (0%) | 0 | Hyperlipidemia: 2 (100%)  Biliary problems: 1 (50%) | Yes: 0 (0%);  No: 0 (0%);  Unclear: 2 (100%) |
| Exenatide | 3 | 0 (0%) | 100; 1–30 days | 0 (0%);  0 (0%) | 1; Probable (Naranjo [4]) | Cholecystectomy: 2 (66%)  Diabetes: 3 (100%)  Hyperlipidemia: 2 (67%)  At least one patient had numerous health issues as well as diabetes (coronary artery disease, RA, obesity, etc.) | Yes: 2 (67%);  No: 0 (0%);  Unclear: 1 (33%) |
| Isotretinoin | 3 | 0 (0%) | 100; >30 days | 0 (0%);  0 (0%) | 0 | Cholecystectomy: 1 (33%)  Hypercholesterolemia: 1 (33%) | Yes: 2 (67%);  No: 1 (33%);  Unclear: 0 (0%) |
| Levetiracetam | 2 | 0 (0%) | 100; 1–30 days | 0 (0%);  0 (0%) | 1; Probable (Naranjo [4]) | Cholecystectomy: 1 (50%) | Yes: 2 (100%);  No: 0 (0%);  Unclear: 0 (0%) |
| Sitagliptin | 3 | 0 (0%) | 100; >30 days | 0 (0%);  0 (0%) | 0 | Renal dysfunction: 1 (33%)  Diabetes: 1 (33%) | Yes: 2 (67%)  No: 1 (33%)  Unclear: 0 (0%) |
| **Class III** | | | | | | | |
| Acetylsalicylic acid | 3 | 1 (33%) | 66.7; 1–30 days | 0 (0%);  0 (0%) | 0 | None | Yes: 1 (33%);  No: 2 (67%);  Unclear: 0 (0%) |
| Gold | 4 | 0 (0%) | 75; 1–30 days | 0 (0%);  0 (0%) | 0 | Immune disorder: 4 (100%) | Yes: 4 (100%);  No: 0 (0%);  Unclear: 0 (0%) |
| Nivolumab | 2 | 0 (0%) | 0; <24 hours and 1–30 days | 0 (0%);  0 (0%) | 0 | None | Yes: 1 (50%)  No: 0 (0%)  Unclear: 1 (50%) |
| Ondansetron | 2 | 0 (0%) | 0; <24 hours and 1–30 days | 0 (0%);  0 (0%) | 0 | None | Yes: 1 (50%);  No: 1 (50%);  Unclear: 0 (0%) |
| Tacrolimus | 2 | 0 (0%) | 0; 1–30 and >30 days | 0 (0%);  1 (50%) | 1; Probable (Mallory and Kern [7]) | None | Yes: 2 (100%);  No: 0 (0%);  Unclear: 0 (0%) |
| **Class IV** | | | | | | | |
| Ado-Trastuzumab emtansine | 1 | No | 1–30 days | No | Probable (Naranjo [4]) | None | Yes |
| Albiglutide | 1 | No | 1–30 days | No | Probable (Naranjo [4]) | Cholecystectomy  Diabetes  Hepatic dysfunction | Unclear |
| Alendronate | 1 | No | 1–30 days | No | Possible (Eland [2]) | None | Unclear |
| Amineptine | 1 | No | 1–30 days | No | No | None | Yes |
| Benazepril | 1 | No | <24 hours | No | No | Diabetes | Yes |
| Brentuximab vedotin | 1 | No | 1–30 days | 0 (0%);  1 (100%) | Probable (Naranjo [4]) | None | Yes |
| Calcium carbonate | 1 | Yes | 1–30 days | No | Possible (Naranjo [4]) | Hyperlipidemia  Renal dysfunction | Yes |
| Capecitabine | 1 | No | >30 days | No | No | Diabetes  Hepatic dysfunction | Yes |
| Chlorthalidone | 1 | No | >30 days | No | No | None | Yes |
| Ciprofibrate | 1 | No | 1–30 days | No | Possible (Eland [2]) | Hyperlipidemia | Unclear |
| Cisplatin | 1 | No | >30 days | No | No | None | Yes |
| Clomipramine | 1 | Yes | <24 hours | No | No | None | Yes |
| Clonidine | 1 | No | 1–30 days | No | No | None | No |
| Demeclocycline | 1 | No | 1–30 days | No | No | None | Yes |
| Doxylamine succinate | 1 | Yes | 1–30 days | No | No | Renal dysfunction | Yes |
| Ertapenem | 1 | No | 1–30 days | No | Probable (Naranjo [4]) | Peritonitis | Yes |
| Estramustine phosphate | 1 | No | 1–30 days | No | No | Diabetes | Yes |
| Famcyclovir | 1 | No | 1–30 days | No | No | Hepatic dysfunction  Renal dysfunction | Yes |
| Gatifloxacin | 1 | No | 1–30 days | No | No | None | Yes |
| Gemfibrozil | 1 | No | >30 days | No | No | Hyperlipidemia | Yes |
| Granisetron | 1 | No | 1–30 days | No | No | None | Unclear |
| Interleukin-2 | 1 | No | 1–30 days | No | No | None | No |
| Lacosamide | 1 | No | >30 days | No | No | Necrotizing acute pancreatitis one month prior to episode | Yes |
| Lamotrigine | 1 | Yes | <24 hours | No | No | None | Yes |
| Linagliptin | 1 | No | >30 days | No | No | Diabetes | Yes |
| Linezolid | 1 | No | >30 days | No | Probable (Naranjo [4]) | Chronic bowel  Peritonitis | Yes |
| Lixisenatide | 1 | No | >30 days | No | No | Diabetes | Unclear |
| Loperamide | 1 | Yes | <24 hours | No | No | None | No |
| Lovastatin | 1 | No | >30 days | No | Probable or possible (Naranjo [4]) | Hyperlipidemia | No |
| Maprotiline | 1 | No | 1–30 days | No | Probable (Eland [2]) | None | Unclear |
| Methandrostenol | 1 | No | 1–30 days | No | No | Hepatic dysfunction | No |
| Micafungin | 1 | No | 1–30 days | No | Probable (Naranjo [4]) | History of gastrectomy due to gastric cancer and idiopathic thrombocytopenic purpura | No |
| Miltefosine | 1 | No | 1–30 days | No | Probable, probable/likely, or possible (Naranjo [4], WHO [10]) | Visceral leishmaniasis | Yes |
| Mizoribine | 1 | No | 1–30 days | No | No | Diabetes  Hepatic dysfunction  Renal dysfunction  Autoimmune disease | No |
| Montelukast | 1 | No | >30 days | No | Probable (Naranjo [4]) | None | Yes |
| Mycophenolate mofetil | 1 | No | 1–30 days | No | No | Renal dysfunction | Unclear |
| Nifuroxazide | 1 | No | <24 hours | No | No | None | Unclear |
| Norfloxacin | 1 | No | 1–30 days | No | No | Malaria | No |
| Pazopanib | 1 | No | >30 days | No | No | Renal dysfunction | Yes |
| PEG-based bowel cleanser | 1 | No | <24 hours | No | No | Cholecystectomy  Previous hemicolectomy for chronic constipation | No |
| Phenformin | 1 | No | 1–30 days | No | No | Diabetes | Unclear |
| Phenolpthalien | 1 | No | 1–30 days | No | No | Chronic constipation | Yes |
| Pregabalin | 1 | No | 1–30 days | No | No | Diabetes | Yes |
| Procetofene | 1 | No | >30 days | No | No | Hyperlipidemia | Unclear |
| Rasburicase | 1 | No | 1–30 days | No | No | None | Unclear |
| Rifamipin | 1 | No | 1–30 days | No | No | None | Yes |
| Ritonavir | 1 | No | >30 days | No | No | HIV | Yes |
| Roxithromycin | 1 | No | 1–30 days | No | No | None | No |
| Stavudine | 1 | No | >30 days | No | No | HIV | Yes |
| Sunitinib | 1 | No | >30 days | No | No | Renal dysfunction | Unclear |
| Tacalcitol | 1 | No | >30 days | No | No | Autoimmune disease | Yes |
| Telmisartan | 1 | Yes | <24 hours | No | Probable (Naranjo [4]) | Hepatic dysfunction | Unclear |
| Tocilizumab | 1 | No | 1–30 days | No | Probable (WHO [10]) | Autoimmune disease | Yes |
| Ursodeoxycholic acid | 1 | No | >30 days | No | No | Cholecystectomy  Hepatic dysfunction | Yes |
| Venlafaxine | 1 | No | >30 days | No | No | None | Yes |
| Zidovudine | 1 | No | >30 days | No | No | HIV/AIDS | Yes |
| Ziprasidone | 1 | No | 1–30 days | No | No | None | Yes |
| ^a^ Cases may have been counted more than once, if they had multiple conditions that may have been associated with acute pancreatitis  ^b^ Chronic bowel = Crohn’s disease, inflammatory bowel disease, or ulcerative colitis  ^c^ Hepatitis = any viral hepatitis, cirrhosis, cholangitis, liver transplant, Alagille’s Syndrome, signs of ascites and/or jaundice  ^d^ Immune disorder = Rheumatoid arthritis, systemic lupus erythematosus, glomerulonephritis, bullous pemphigoid, autoimmune pancreatitis, or optic neuritis  ^e^ Renal dysfunction = end-stage renal disease, acute or chronic renal disease/insufficiency/failure, glomerulonephritis/lupus nephritis, IgA nephropathy, nephrotic syndrome, renal transplant +/- rejection, renal carcinoma, nephrolithiasis, other renal insufficiency, drug nephrotoxicity, dialysis treatment, membranous glomerulopathy, metastatic involvement of kidney, nephrectomy, or pyelonephritis  AIDS = acquired immune deficiency syndrome; ALS = amyotropic lateral sclerosis; AP = acute pancreatitis; CML = chronic myelogenous leukemia; DIP = drug-induced pancreatitis; GVHD = graft-versus-host disease; HIV = human immunodeficiency virus; NA = not applicable; RA = rheumatoid arthritis; SLE = systemic lupus erythematosus | | | | | | | |

**References for Detailed Summary Table**

1. Bégaud B, Evreux JC, Jouglard J, Lagier G. [Imputation of the unexpected or toxic effects of drugs. Actualization of the method used in France]. Therapie. 1985;40: 111–118.

2. Eland IA, van Puijenbroek EP, Sturkenboom MJ, Wilson JH, Stricker BH. Drug-associated acute pancreatitis: twenty-one years of spontaneous reporting in The Netherlands. Am J Gastroenterol. 1999;94: 2417–2422.

3. Karch FE, Lasagna L. Toward the operational identification of adverse drug reactions. Clin Pharmacol Ther. 1977;21: 247–254.

4. Naranjo CA, Busto U, Sellers EM, Sandor P, Ruiz I, Roberts EA, et al. A method for estimating the probability of adverse drug reactions. Clin Pharmacol Ther. 1981;30: 239–245.

5. Delcenserie R. [What are the criteria for imputation of drug-induced pancreatitis?]. Gastroenterol Clin Biol. 2001;25: 1S18-1S21.

6. Maliekal J, Drake CF. Acute pancreatitis associated with the use of lisinopril. Ann Pharmacother. 1993;27: 1465–1466.

7. Mallory A, Kern F. Drug-induced pancreatitis: a critical review. Gastroenterology. 1980;78: 813–820.

8. Kramer MS, Leventhal JM, Hutchinson TA, Feinstein AR. An algorithm for the operational assessment of adverse drug reactions. I. Background, description, and instructions for use. JAMA. 1979;242: 623–632.

9. Dangoumau J, Evreux JC, Jouglard J. [Method for determination of undesirable effects of drugs]. Therapie. 1978;33: 373–381.

10. The Uppsala Monitoring Centre. The use of the WHO-UMC system for standardised case causality assessment. Available: http://www.who.int/medicines/areas/quality_safety/safety_efficacy/WHOcausality_assessment.pdf

11. Delcenserie R, Grange JD, Laugier R, Bernades P. [Definition and criteria of imputability of drug-induced acute pancreatitis]. Gastroenterol Clin Biol. 1992;16: 761–763.
