# Supplementary material for: Drug induced pancreatitis: A systematic review of case reports to determine potential drug associations
Source: PLoS One. 2020 Apr 17;15(4):e0231883. doi: 10.1371/journal.pone.0231883 (PMC7164626; doi:10.1371/journal.pone.0231883)
Supplement: S6 Text — (DOCX) [file pone.0231883.s006.docx]

S6 text: Sensitivity analysis - Positive imaging findings required for a diagnosis of DIP

**Drugs associated with DIP in case reports with positive imaging findings (n = 429)**

| **Class Ia**  **(n = 31 drugs)** | **Class Ib**  **(n = 33 drugs)** | **Class Ic**  **(n = 43 drugs)** | **Class II**  **(n = 5 drugs)** | **Class III**  **(n = 6 drugs)** | **Class IV**  **(n = 42 drugs)** |
| --- | --- | --- | --- | --- | --- |
| 5-ASA | 6-MP | Adefovir dipivoxil | Ceftriaxone | Acetylsalicylic acid | Ado-Trastuzumab emtansine (T-DM1) (Kadcyla) |
| Acetaminophen | Antilymphocyte globulin (ALG) | Amiodarone | Exenatide | Carbamazepine | Amineptine |
| Azathioprine | ATRA | Amoxicillin/clavulinic acid | Isotretinoin | Gold | Benazepril |
| Captopril | Cimetidine | Artesunate | Sitagliptin | Nivolumab | Bortezomib |
| Carbimazole | Ciprofloxacin | Atorvastatin | Stibogluconate | Pentamidine | Brentuximab Vedotin |
| Codeine | Clomiphene | Axitinib |  | Tacrolimus | Calcium carbonate |
| Dapsone | Clothiapine | Boceprevir |  |  | Capecitabine |
| Fluvastatin | Clozapine | Canagliflozin |  |  | Doxylamine succinate |
| Furosemide | Cytarabine | Celecoxib |  |  | Ertapenem |
| Isoniazid | Didanosine | Clarithromycin |  |  | Estramustine phosphate |
| L-asparaginase | Enalapril | Danazol |  |  | Gemfibrozil |
| Lisinopril | Everolimus | Dexfenfluramine |  |  | Granisetron |
| Metformin | Growth Hormone | Diclofenac |  |  | IL-2 |
| Methimazole | Hydrochlorothiazide | Diethylstilbestrol |  |  | Lacosamide |
| Methylprednisolone | Hydrocortisone | Dilantin |  |  | Levetiracetam |
| Metronidazole | IFN alpha | Doxycycline |  |  | Linagliptin |
| Nitrofurantoin | Ifosfamide | Erythromycin |  |  | Linezolid |
| Piroxicam | Indalpine | Finasteride |  |  | Lixisenatide |
| Pravastatin | Lamivudine | Flurbiprofen |  |  | Methandrostenolone |
| Premarin | Mefenamic acid | Gadolinium |  |  | Metolazone |
| Pyritinol | Meglumine antimoniate | Glimepiride |  |  | Micafungin |
| Ramipril | Mirtazapine | Ibuprofen |  |  | Miltefosine |
| Rosuvastatin | Nelfinavir | IFN beta |  |  | Mizoribine |
| Simvastatin | Octreotide | Indomethacin |  |  | Montelukast |
| Sorafenib | Omeprazole | Ketoprofen |  |  | Mycophenolate mofetil |
| Tamoxifen | Perindopril | Ketorolac tromethamine |  |  | Naltrexone |
| Telaprevir | Prednisolone | Lanreotide |  |  | Nifuroxazide |
| Tetracycline | Propofol | Lenvatinib |  |  | Norfloxacin |
| Tigecycline | Quetiapine | Liraglutide |  |  | Oxyphenbutazone |
| TMS | Saxagliptin | Meprobamate |  |  | Prednisone |
| VPA | Sulfasalazine | Minocycline |  |  | Pregabalin |
|  | Thalidomide | Nilotinib |  |  | Rifampin |
|  | Voriconazole | Olanzapine |  |  | Risperidone |
|  |  | Olsalazine |  |  | Ritonavir |
|  |  | Paclitaxel |  |  | Roxithromycin |
|  |  | Pantoprazole |  |  | Sulindac |
|  |  | Riluzole |  |  | Sunitinib |
|  |  | Rofecoxib |  |  | Tacalcitol |
|  |  | Secnidazole |  |  | Tocilizumab |
|  |  | Sirolimus |  |  | Ursodeoxycholic Acid |
|  |  | Theophylline |  |  | Venlafaxine |
|  |  | Vedolizumab |  |  | Ziprasidone |
|  |  | Vildagliptin |  |  |  |

**Drugs no longer associated with DIP, when positive imaging was required for the diagnosis of AP**

- Albiglutide
- Alendronate
- Ampicillin
- Bezafibrate
- Candesartan
- Chlorthalidone
- Ciprofibrate
- Cisplatin
- Clofibrate
- Clomipramine
- Clonidine
- Demeclocycline
- Dexamethasone
- Dimethyl fumarate
- Diphenoxylate with atropine
- Eluxadoline
- Ezetimibe
- Famcyclovir
- Gatifloxacin
- Glicazide
- Irbesartan
- Itraconazole
- Ixazomib
- Lamotrigine
- Loperamide
- Losartan
- Lovastatin
- Maprotiline
- Methyldopa
- Metronidazole
- Naproxen
- Ondansetron
- Oral contraceptive
- Orlistat
- Paromomycin
- Pazopanib
- PEG bowel cleanser
- Phenformin
- Phenolpthalien
- Procainamide
- Procetofene
- Propylthiouracil
- Ranitidine
- Rasburicase
- Rifampicin
- Salazopyrine
- Stavudine
- Telmisartan
- Tiaprofenic acid
- Tinidazole
- Valsartan
- Vemurafenib
- Zidovudine
